# dbVOR: a database system for importing pedigree, phenotype and genotype data and exporting selected subsets

**DOI:** 10.1186/s12859-015-0505-4

**Published:** 2015-03-18

**Authors:** Robert V Baron, Yvette P Conley, Michael B Gorin, Daniel E Weeks

**Affiliations:** Department of Human Genetics, Graduate School of Public Health, University of Pittsburgh, PittsburghPennsylvania, 15261 USA; Department of Health Promotion and Development, School of Nursing, University of Pittsburgh, Pittsburgh, Pennsylvania, 15261 USA; Department of Ophthalmology, David Geffen School of Medicine, Stein Eye Institute, University of California Los Angeles, Los Angeles, California, 90095 USA; Department of Biostatistics, Graduate School of Public Health, University of Pittsburgh, Pittsburgh, Pennsylvania, 15261 USA

**Keywords:** Association studies, Database, Genotypes, Genome-wide association studies, Linkage, Mega2, MySQL, Phenotypes, PLINK, Python

## Abstract

**Background:**

When studying the genetics of a human trait, we typically have to manage both genome-wide and targeted genotype data. There can be overlap of both people and markers from different genotyping experiments; the overlap can introduce several kinds of problems. Most times the overlapping genotypes are the same, but sometimes they are different. Occasionally, the lab will return genotypes using a different allele labeling scheme (for example 1/2 vs A/C). Sometimes, the genotype for a person/marker index is unreliable or missing. Further, over time some markers are merged and bad samples are re-run under a different sample name. We need a consistent picture of the subset of data we have chosen to work with even though there might possibly be conflicting measurements from multiple data sources.

**Results:**

We have developed the **dbVOR** database, which is designed to hold data efficiently for both genome-wide and targeted experiments. The data are indexed for fast retrieval by person and marker. In addition, we store pedigree and phenotype data for our subjects. The **dbVOR** database allows us to select subsets of the data by several different criteria and to merge their results into a coherent and consistent whole. Data may be filtered by: family, person, trait value, markers, chromosomes, and chromosome ranges. The results can be presented in columnar, Mega2, or PLINK format.

**Conclusions:**

**dbVOR** serves our needs well. It is freely available from https://watson.hgen.pitt.edu/register. Documentation for **dbVOR** can be found at https://watson.hgen.pitt.edu/register/docs/dbvor.html.

**Electronic supplementary material:**

The online version of this article (doi:10.1186/s12859-015-0505-4) contains supplementary material, which is available to authorized users.

## Background

Genetic studies of complex human diseases typically involve multiple iterations of genetic marker generation. For example, a set of individuals may first be assayed with a genome-wide chip that measures genotypes at hundreds of thousands of markers. Then a subset of interesting signals may be followed up by custom genotyping using a different technology. If there are technical problems, yet another round of custom genotyping using a third technology might be carried out on a subset of the samples. Managing and reconciling these multiple sources of data, often with different sample IDs, allele labels, and marker names, is best managed using a database system.

While several database systems have been developed for managing genetic data[1-7], when we tried some of these, we found that some relied on commercial database systems that were so complicated that they required a database administrator to routinely maintain and apply regular security updates. Others did not scale well as the numbers of markers genotyped per experiment rapidly increased. Some were written with sophisticated web-based interfaces, that, while elegant, were difficult to extend and customize as needed. Therefore, we set out to build our own database system, based on open-source MySQL, driven by command-line programs written in Python. We named our database system “dbVOR”, after Vör, the inquiring Norse goddess of wisdom from whom nothing can be concealed.

## Overview

**Figure 1:**
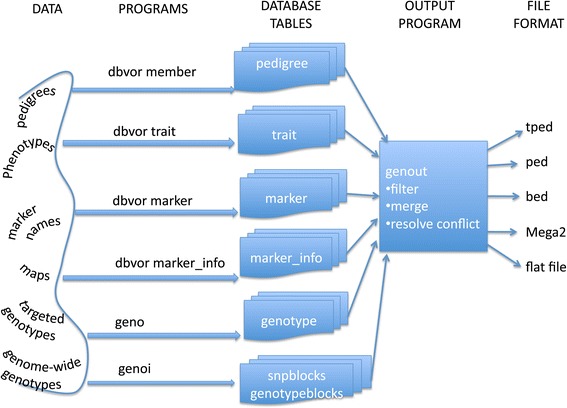
dbVOR usage: raw data is loaded into database tables by one of several programs. The **genout** program accesses the database tables, filters and merges the data and outputs genotypes in one of several common formats.

We have created a series of programs and a database called, **dbVOR**, to store, manage, and retrieve genetic data. Figure 1 illustrates the overall operations of **dbVOR**. The raw data flows into the database tables using one of several programs. Finally, the **genout** program can be used to filter and merge data to be output in one of several file formats.

The rest of this document is organized as follows: in the Implementation section, we first describe **dbVOR**’s ancillary database tables used to store pedigree, trait, and marker data. Then we show the tables used for storing genotype data: one for targeted experiments and the other set for genome-wide experiments. The key difference between these is that for genome-wide data we store multiple genotypes per record, in blocks. We next discuss the programs that are used to enter data into the aforementioned tables. A subsequent program is presented that extracts data from the database tables. The next few sections are intended to give a feel for details of the implementation: configuration files, log files, note storage, and database commit policy. The Example section illustrates the format and use of the targeted and genome-wide genotype tables. It also illustrates how multiple conflicting genotypes for the same person and marker coming from multiple or even the same experiment get resolved. The Data statistics section show how conflicting genotype information is summarized for user review. The Results section compares the efficiency of the genome-wide multiple genotypes per record representation to that of the simpler single genotype per record representation. The performance is also illustrated in the Discussion section via comparisons with existing systems. Finally, the Appendix shows the full database schema.

We maintain the following typographic conventions: All programs and subcommands are shown in a **bold** typeface. The names of database tables are shown in ***bold italic***.

## Implementation

### Database tables

**dbVOR** stores information about the collected genetic data in several tables. We will explain the important aspects of each table. For completeness, the entire schema is presented in the Appendix. This section describes the auxiliary tables while the next section describes the tables that store the genotype data.

The ***samples*** table stores plate, well, and miscellaneous study-related information about a sample, relates the sample identifier to a subject, and specifies the person from whom the sample was taken. If multiple samples exist for the same person, a preferred sample may be indicated, causing the other samples to be ignored.

The ***members*** table stores a pedigree and person name for a subject; it also stores the parent identifiers and sex of the subject (By design, all tables have a unique ID field for connecting data from different tables together). An auxiliary table, ***member_aliases***, provides alternate names for members and supports multiple differently named samples from the same person.

The ***traits*** table provides values for specified phenotypic traits for each subject. Not all subjects need to have known values for all traits. A glue table, ***traitmeta***, links trait name strings with trait IDs and contains a type (e.g. quantitative or qualitative) for each trait. The type is used to validate the trait values, and to ensure proper formatting on output.

The ***markers*** table associates a marker name string with an ID. It is used to check the input marker names for typographical errors in that marker names appearing in other contexts are verified against the names in the ***markers*** table. There is also a ***marker_aliases*** table that provides alternate names for ***markers*** to be used when marker names change over time.

The ***marker_info*** table contains the chromosome number and base pair position as well as the genetic positions (male, female, and average) of each marker. Each record is tagged with the build number from which the positions were derived.

Occasionally allele data coming back from our collaborators will be labeled differently from our other data. For example, a genotype might be designated as 1/2 when another genotyping platform might designate the genotype as A/G. The ***allele_map*** table can specify a remapping from one set of allele labels to another. Entries also contain an experiment ID key and a marker ID key.

The individual tables described so far are relatively small even for the larger data collections.

The ***genotypes*** table holds the genotype data for member-marker pairs. In addition to a member key and a marker key, each record contains a technology ID key and an experiment ID key. These two IDs come from the ***technologies*** table and the ***experiments*** table, respectively, which map name strings to IDs. Technology is used to label the technology used to generate the marker data (e.g., Illumina or Affymetrix) while Experiment describes a specific instance or batch of genotyping data generated using a particular technology.

The ***genotypes*** table provides an effective way to manage small volumes of data, thousands of members with several thousands markers, and gives adequate performance on moderately powered machines. But this strategy is not effective for large-scale studies with hundreds of thousands or millions of markers; this is illustrated in the Example section. We have a novel data representation for large numbers of markers. Rather than store a single marker’s genotype information per record, we store all the genotypes for a contiguous run of markers on a chromosome in the ***genotypeblocks*** table and use multiple blocks for each chromosome; this is also illustrated in the Example section. An auxiliary table, ***snpblocks***, is necessary to map a marker to the corresponding chromosome, block, and offset. This strategy is designed to efficiently read all the data on a chromosome or the data from ranges of markers on a chromosome; this is usually how data are read from the database. To be clear, the efficiency is for writing and reading records for experiments involving genome-wide large-scale data from the database; not for storage efficiency.

### Data entry programs

Data are entered into all but the genotype tables using the command line program, **dbvor**. It is immediately followed by a subcommand, for example **marker_info**. This option loads the ***marker_info*** table. The list of subcommands is indicated in Table 1.

**Table 1:** **Some of the programs that populate the database tables**

**Program name**	**Table**	**Behavior**
**dbvor createdb**	all tables defined	create a database, create auser and load schemadefinitions
**dbvor newmarker**	***markers***	define new markers
**dbvor missingmarkerinfo**	***markers, marker_info***	list markers without map info for the named build
**dbvor allelemap**	***allele_map***	store genotype remapping for a marker/experiment
**dbvor marker_info**	***marker_info***	store genetic and physical position data for a specific map build
**dbvor markeralias**	***marker_aliases***	store alternate names for markers
**dbvor gmimarker**	***marker_info***	store marker mapinformation presented inGenetic Map Interpolator(GMI) format [8]
**dbvor gmimarkeralias**	***marker_aliases***	store marker aliasing data presented in GMI format
**dbvor sample**	***samples***	store sample data andassociate it with thecorresponding members
**dbvor trait**	***traits, traitmeta***	store trait values formembers
**dbvor member**	***members***	store pedigree information
**dbvor memberalias**	***member_aliases***	store alternate names for members
**dbvor create_experiment**	***experiments***	create entry in ***experiment*** table associating ID with experiment string
**dbvor set_experiment_date**	***experiments***	set date field ofspecified experiment inthe ***experiment*** table
**dbvor delete_experiment**	***experiments***	delete all genotype data for the specified experiment
**dbvor set_active**	***genotypes***	set the active flag, to turn on/off specific data

To initially set up a project, one would first use the **dbvor** subcommand **createdb**, which creates a data schema in the database and loads the **dbVOR** table definitions into it. This subcommand also adds users to the database and grants them the appropriate privileges to use the data schema. There is a complimentary subcommand to undo these actions.

After initializing the **dbVOR** tables, then one would use the **dbvor** program to move data from flat files into the database (Table 1). The subprograms inherit from a hierarchy of Python classes that support the standard operations of *insert*, *update*, *compare* and *delete*, all of which work in a similar way across each utility. A typical utility, **dbvor marker*_*info**, stores map information for collections of markers. The flat file containing the input data is referenced from the configuration file, as described in the ‘Configuration files’ section below. Additional information in the configuration file indicates which column contains the *markername*, *chromosome*, *base pair position*, *average genetic position*, *female genetic position* and *male genetic position*. All columns except for *marker name* are optional. The configuration file must also specify the map build. Typing the command “**dbvor marker*_*info <configuration file>**” will parse data files and test for correctness. Adding the **commit** flag will insert the data into the database if there are no errors. Running the utility a second time with the **commit** flag but with no change to the underlying flat input file will make no changes to the database. You can extend the flat file with new rows. Now, redoing the **commit** operation will add *just the new entries* to the database. Thus in the end, the database will have the same information as the extended flat file. The flat file data may be revised (corrected) from what was originally in the database. Processing the revised flat file with the **compare** flag will show those entries where the flat file and database differ. The **commit** flag only adds new information into the database; it will not change existing records. It will just print a warning. The **replace** flag together with **commit** will copy any changed records and new records into the database. For each changed record, the program first removes the old record and then adds a new record. For some usages, this may violate database consistency checks in which case the **update** flag can be used instead of **replace**. For example, it you wanted to change the parents of a pedigree member whose database identifiers had been used in other tables (say ***trait*** or ***genotypes***), database integrity checks would not let you remove the member record from the database. But you can **update** the member record. Finally, the **delete** flag will remove all the records in the flat file from the database.

Similar utilities (Table 1) are available to add markers, pedigrees, traits, aliases, etc. They all use the same set of flags, which behave as described above.

The **geno** program (Table 2) enters data into the ***Genotypes*** table. It accepts genotype data in several formats: alleles in separate columns, allele pairs in a column, all genotypes for a person on one line, etc. If errors are detected, the data are rolled back out of the database. For example, each marker name used by **geno** must already be in the marker table, thus ensuring internal consistency of marker information within the database. The main reason the **geno** program is separate from the utilities accessed through the **dbvor** program is that there are a number of automated tests performed and statistics compiled as the genotype data are entered into the database. In particular, a warning will be raised for person/markers that are already in the database with a different genotype value for a given marker name, technology, and experiment. We do allow multiple genotype entries for the same member-marker if they have different technologies/experiments.

**Table 2:** **Programs that add genotype data to the database**

**Program name**	**Table**	**Behavior**
**geno**	***genotypes***	populate ***Genotypes*** table
**genoi**	***genotypeblocks, snpblocks, marker_info***	populate ***Genotypeblocks***, ***snpblocks***, and ***marker_info*** tables from Illumina data
**genoa**	***genotypeblocks, snpblocks, marker_info***	populate ***Genotypeblocks***, ***snpblocks***, and ***marker_info*** tables from Affymetrix data
**genok**	***genotypeblocks, snpblocks, marker_info, members, samples, traits***	populate ***Genotypeblocks***, ***snpblocks***, ***marker_info***, ***members***, ***samples***, and ***traits*** tables from PLINK binary format data

Illumina genome-wide marker data are often contained in many files, with one sample per file, complemented with an additional file which maps the chip assay ids to markers. (There are several ways to make the mapping file, such as look up the specified chromosome/position in a current build or do a BLAST look up of the specified flanking sequences). All these flat files contain simple columnar data. The **genoi** program (Table 2) inserts Illumina data by reading the marker mapping file and the collection of sample files. The marker data are inserted into the ***genotypeblocks*** table in the database and the relevant columns from the marker mapping data are inserted into the ***marker*_*info*** table as well as the ***snpblocks*** table.

Affymetrix data could, in principle, be represented the same way as Illumina data with one sample per file, but Affymetrix data are typically presented as a varying number of samples per file, along with a file of marker map information. The **genoa** program (Table 2) handles reading this type of data.

PLINK [9] uses a handful of flat files to efficiently hold genome-wide data. It is common to use PLINK binary format for processed genome-wide study data. One flat file, defines the markers, another file defines the pedigrees, and a final file in a compressed (binary) format defines the genotypes for the markers and pedigrees. The flat files contain simple columnar data. The **genok** program (Table 2) inserts the marker info into the ***marker*** table, the ***markerinfo*** table, as well as the ***snpblocks*** table. The pedigree data go into the ***members*** table, ***samples*** table, and ***traits*** table. Finally, the binary file is parsed to populate the ***genotypeblocks*** table.

The dbVOR code modules for processing genome-wide data consist of a base class that handles processing and storing the genotype and marker data and a derived class responsible for fetching the genotype data from the input files. This structure makes it possible to extend the processing to new input data formats with minimal revision of **dbVOR**.

### Data extraction

The **genout** program extracts genotype and phenotype data from the database. The extracted data may be formatted as simple columnar tables separated by tabs, commas or white space. In addition, the data may be extracted in Mega2 [10] format or most of the PLINK [9] formats (including binary formats), facilitating further analysis.

The extracted data can be filtered by including specific pedigrees and persons and/or excluding specific pedigrees and persons. As the filtering criteria might remove people needed to specify complete pedigree structures, the pool of selected individuals may be increased to include all members of any previously selected pedigree. Traits that should appear in the output can be listed and the population can be filtered by requiring members to have specific values for specified traits.

Unreliable measurements can be removed from the output. Markers of a given experiment may be filtered out via a list in the configuration file (described below) used by the extraction program, **genout**. In addition, ranges of markers on a chromosome, possibly associated with some particular individuals, can similarly be filtered out. Alternatively, filtering decisions can be recorded in the database by setting the ’active’ flag appropriately. This mechanism allows the user to tag data already in the database as not to be used.

Alternatively, a filter may be used to select a list of desired markers. Or all the markers on one or more chromosomes can be selected. In addition, sub-ranges of each chromosome, indicated in base pairs or genetic distance, can be requested. If any marker has no observations for all the subjects selected, it can be dropped from the output.

Since the database contains experiment and technology keys, the markers can be restricted to only those coming from particular experiments and/or technologies. In addition, the subjects can be restricted to those having markers from a specific experiment and/or technology.

### Configuration files

All the dbVOR programs are run from the command line. Most input programs read one or more files of data and load them into the appropriate database tables. Additional configuration information is needed for these programs. To avoid having numerous command line switches and flags, a configuration file is usually used (supplementing any parameters supplied on the command line). This technique makes it easy to repeat a previous run, as well as to supply many parameters and lists among the arguments: markers, persons, etc.

### Log files

Error reports and status information are written into a log file during each run. The log file incorporates the date and time of the run to create a unique file name (The log file name can be changed through the configuration file). After a run, it can be useful to review the log file to investigate errors, find problems, and read complete reports. Each log file is divided into several sections. The first few lines of each section are displayed on the screen during the program run to indicate to the user the kinds of activities and/or problems that are occurring. If the normal program operation does not present enough status information, one can add a verbose flag (and even a debug flag) to write more details of the internal operations of the program to the log file.

### Note storage

Most **dbVOR** tables contain a time stamp to record when each record was changed. But a time stamp is insufficient for documenting what changed and why. Our preferred way to keep notes is to record changes and their reasons to a (text) "note file". We provide a ***notes*** table as part of **dbVOR** that contains fields for notes and data as well as keywords and comments for easy retrieval. Thus the venerable “note file” and the related data can become a permanent part of the database. We typically would expect to store the log file associated with a **commit** of data to the database; there is a flag that can be added to any program to create a note in the database containing the log file of a **commit** as well as user-supplied notes.

### Computing resources

All the **dbVOR** programs are written in Python version 2.6. The database used is MySQL. In our customary usage, both the database and the **dbVOR** programs run on the same machine. Python and MySQL are available for many different platforms. Thus **dbVOR** should be very portable, though we developed it on Macintoshes running OS X. Version 1.11 of **dbVOR** is available in Additional file 1, along with a companion program needed for full functionality: version 1.4 of the Genetic Map Interpolator (GMI) [8] as Additional file 2; for updated versions of these, please check the **dbVOR** home page (https://watson.hgen.pitt.edu/register).

It should be possible to replace MySQL with a different choice of database. **dbVOR** should be portable, because its SQL commands are exclusively constructed in Python - there are no stored procedures used in **dbVOR**. The SQL Table definitions (Data Definition Language) would need to be adjusted to the new database syntax and a Python interface package to the database would also be required. The SQL used for data manipulation should be simple enough to be supported by most database engines.

### Database commit/Rollback policy

We want clarify our use of the **commit** flag described earlier; it is part of a protocol to ensure that no incorrect data is stored into the database. Each program that prepares data for insertion into the database checks many details, but it does not actually put data in the database unless the **commit** flag is given. Further, if any errors happen while inserting the data when a **commit** was requested, all the data are rolled back. One must then fix all errors that were found and run the program again.

## Example

Given the database design and programs described above, we now will show, using a simple example, how genotypes are pulled from the database. As a sample can be genotyped more than once at a given marker, we illustrate **dbVOR**’s support for handling and resolving any disagreements among the repeated genotypes.

### Extracting genotypes

**Figure 2:**
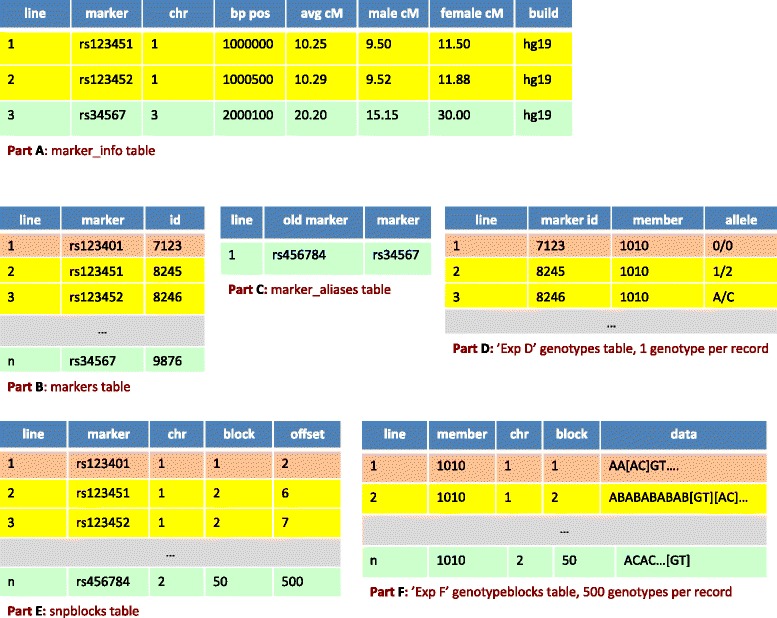
An example of the marker look up process. The tan lines show a request for a specific marker. Next, the yellow lines show a request for markers by chromosome range. Finally, the green lines illustrate how the marker name and position can change. The 0/0 genotype in line 1 of Part D represents the missing genotype. The [ ] brackets in Part F point out the 6th and 7th genotype on line 2 and 500th genotype on line n; the brackets are not part of the genotype data.

**Figure 3:**
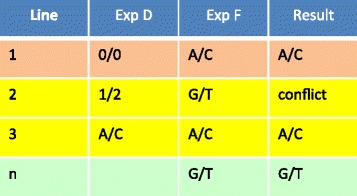
Possible conflicts in genotypes, and their resolution, arising from two experiments, Exp D and Exp F, covering the same subject and marker. “0/0” represents an unknown genotype.

Our **dbVOR** database design has two major tables: the ***genotypes*** table for a small number of markers and the ***genotypeblocks*** table for genome-wide scale data. Each row of the ***genotypeblocks*** table contains multiple genotypes; the example portrayed in Figure 2 Part F has 500 genotypes per record. We now illustrate how genotypes are fetched, both from the ***genotypes*** table and from the ***genotypeblocks*** table. This is presented in Figure 2. The tan lines show a request for a specific marker. Genotypes for rs123401 (***markers*** table, Figure 2 Part B line 1) are selected from experiment ‘Exp D’ in the ***genotypes*** table (Part D line 1) and experiment ‘Exp F’ in ***genotypeblocks*** table (Part F, line 1). (To pull data from the ***genotypeblocks*** table, we needed to look up rs123401’s block and offset indices in the ***snpblo*cks** table (Part E, line 1)). This selection pulls 0/0 from Exp D in the ***genotypes*** table (Part D line 1) and A/C from Exp F in ***genotypeblocks*** table (Part F line 1), as summarized in Line 1 of Figure 3. Note that the example tables in Figure 2 are simplifications and in the full dbVOR system, the experiment is attached to the row information.

Next, the yellow lines show a request for markers by chromosome range. Suppose we request markers on chromosome 1 between positions 10000000 and 20000000. The selected markers are shown in yellow in the ***marker_info*** table (Figure 2 Part A lines 1 & 2) - these represent markers rs123451 and rs123452 (***marker*** table, Part B lines 2 & 3). Retrieving genotypes for these markers from Exp F requires one select from ***genotypeblocks*** table; genotypes for both markers are stored in the same record (Part F line 2). But retrieving genotypes from Exp D requires multiple selects from the ***genotypes*** table (Part D, lines 2 & 3). This selection pulls 1/2 and A/C from the ***genotypes*** table (Part D) and G/T and A/C from the ***genotypeblocks*** table (Part F), as summarized in Lines 2 and 3 of Figure 3.

Finally, for the green lines the marker name and position have changed over time: rs456784 was on chromosome 2 when first imported into the ***snpblocks*** table (Figure 2 Part E, line n); now it is on chromosome 3 (***marker_info*** table, Part A line 3). Furthermore, rs456784 is now aliased to rs34567 in the ***marker_alias*** table (Part C, line 1). This marker was requested by either name or by asking for chromosome 3 (in its entirety or by an appropriately chosen range). A genotype at this marker is not available in ***genotypes*** table (Part D line 1), but is G/T from the ***genotypeblocks*** table (Part F, line n) of Figure 3.

### Genotype conflicts

If data are fetched from more than one experiment, some markers may have more than one measured genotype for a given individual. It does not matter if the experiments are all targeted or all genome-wide or if we have some of each. Whenever there are multiple measurements for the same person and marker, a conflict may arise. Note also that a single experiment may have two or more measurements for the same marker and person. Any conflicts must ultimately be resolved prior to outputting the data for further analysis. If all the known measurements concur with each other and the rest are unknown, the common measured value is output. This is illustrated in Figure 3 lines 1 and 3 (Creation of this table was explained above, based on Figure 2). If the genotype measurements conflict (Figure 3 line 2), we offer several mechanisms to resolve the conflict. If some experiments are deemed to be more trustworthy than others, a trust level can be specified for the experiment or marker. Alternatively, some poor quality experiments, though still stored in the database, can be marked as “not to be used” under normal circumstances. If after applying the trust metric, there are still multiple conflicting genotypes for a single member/marker, **dbVOR** will record the conflict in its log and output a missing genotype into the output file. A similar trust/ignore mechanism can be used to select which sample to use when a sample has been run on more than one plate (and/or duplicated as a control).

### Data statistics

As the data pass from the **dbVOR** database to the output files, information is accumulated by the **genout** program so that summary statistics can be produced. For example, the allele and genotype frequencies for each marker can be displayed. In addition, all the member/marker measurements that show more than one measurement are recorded for further analysis and to display potential conflicts. However, for a large scale studies with thousands of people and millions of markers, statistical record keeping can, in our experience, exhaust the resources of the machine. Under these circumstances, we suggest that the user gather statistics for one chromosome at a time, as this is more manageable. Alternatively, a flag is available to request that no statistics be gathered by **genout**and that one person’s worth of data be processed at a time, written out, deallocated from memory, and so forth.

When statistics are requested, then for every marker genotype that is measured by more than one experiment for a given person, **dbVOR** shows a matrix indicating the agreement of the experiment results for each experiment pair. For example, consider the following agreement matrix for a marker at two experiments: exp1 and exp2 (Table 3). From the first column, we observe that the second experiment did not measure 130 people that were successfully genotyped in the first experiment and in the first row we find two people that were not typed in the first experiment but were in the second experiment. We also see that the two experiments mostly agree on the other measurements except for three people: one person at **[1/1, 1/2]** meaning exp1’s genotype was **1/1**while exp2’s was **1/2** and two people at **[1/2, 1/1]**meaning exp1’s genotype was **1/2**for two people while exp2’s was **1/1**. We would output a missing genotype **0/0** for these 3 cases.

**Table 3:** **Example agreement matrix between two experiments, exp1 and exp2**

**exp1** ***↓*** **/exp2 →**	**0/0**	**1/1**	**1/2**	**2/2**
**0/0**	2	1	1	0
**1/1**	19	194	1	0
**1/2**	58	2	549	0
**2/2**	53	0	0	534

The genotypes from the first experiment label the rows and those from the second label the columns.

In Table 4, the agreement matrix informs us that these two experiments used different labeling schemes for reporting the genotypes. The automatic mechanism would see a conflict and would output a value of **0/0** for all these data. If supported by the laboratory records, the ***allele****_****map*** table can be used to resolve this difficulty by remapping allele **1** to **A**, and allele **2** to **C** for the marker in exp3.

**Table 4:** **Example agreement matrix between two experiments, exp3 and exp4**

**exp3** ***↓*** **/exp4 →**	**0/0**	**A/A**	**A/C**	**C/C**
**0/0**	0	0	0	0
**1/1**	0	190	0	0
**1/2**	0	0	504	0
**2/2**	0	0	0	359

The genotypes from the first experiment label the rows and those from the second label the columns.

## Results

Here we discuss some timing data based on a real data set, as well as compare **dbVOR** to other previously published database software.

### Performance

To measure performance, we used an Illumina HumanExome-12v1 exome chip data set where 1,058 samples were genotyped at 247,519 markers by the Center for Inherited Disease Research. These data were generated in accordance with the Declaration of Helsinki as part of our Genetics of Age-related Macular Degeneration study, with the approval of the UCLA and University of Pittsburgh Institutional Review Boards. Our performance measurements were performed on an iMac with a 3.33 GHz Intel Core 2 Duo processor having 8 GB memory. First, we used a small subset of our Illumina exome chip data with 25,143 markers measured on 1,058 samples contained in 1,058 files. This is about 10% of the available markers; these markers were chosen randomly from the 247,519 markers available. Loading these data into the ***Genotypeblocks*** table required 255 seconds (most of this time was consumed in reading and parsing the files.). The ***Genotypeblocks*** table has 66,654 blocks (i.e. rows); 63 blocks per sample (times 1058 samples equals 66,654). For each sample, chromosome 1 occupies 5 blocks, chromosomes 2, 6, and 11 each occupy 4 blocks, chromosomes 3, 4, 5, 7, 9, 12, 16, 17, and 19 each occupy 3 blocks, chromosomes 8, 10, 14, 15, 20, 22, and 23 each occupy 2 blocks and the remaining chromosomes (13, 18, 21, 24, and 26) each occupy just 1 block. All these data were output into a single file similar to PLINK PED format (using **genout**); and read into an empty ***Genotypes*** table. The populated ***Genotypes*** table contained 26,591,772 records. (This number is a bit smaller than 25143 markers * 1058 samples because missing genotypes are not stored in the database in this representation.) These records are 399 times the records of the ***Genotypeblocks*** table. Loading the data took 1,535 seconds. This is six times slower but this number does not tell the whole story. The time spent doing the database insert for the ***Genotypeblocks*** table was 30 seconds while the time for the ***Genotypes*** table was 1,284 seconds, a factor of 40 slower. In addition, as the ***Genotypes*** table is 399 times larger, this slows down database operations such as select and delete (As an example, using “delete” to remove all the entries in the genotypes table [and doing the requisite database book keeping to allow rollback] took over 30 minutes).

Extracting data from the database was faster when pulling from the ***Genotypeblocks*** table than from the ***Genotypes*** table. Fetching all the genotype data into PLINK bed format using the ***Genotypeblocks*** table took 268 seconds while fetching from the ***Genotypes*** table took 685 seconds. Both these timings are for fetching the data while keeping no statistics on the results.

The full Illumina exome chip data set has 247,519 markers for 1,058 samples. Loading this into the ***Genotypeblocks*** table took 2,245 seconds (0.62 hours). If loading the ***Genotypes*** table is 6 times slower, it would take 13,470 seconds (3.74 hours) to load the same data. Scaling the load times shown earlier, we predict that the ***Genotypeblocks*** load for a data set with 1,000 samples and 1,000,000 markers should take 2 hours and 40 minutes, while ***Genotypes*** should take 16 hours (This comparison assumes that the database performance scales linearly with the number of records – which is not true). Extracting all the markers for 1,058 members from the ***Genotypeblocks*** table into PLINK BED format took 3,305 sec, while extracting just the markers from chromosome 1 (25,175 markers) required 267 seconds.

We also timed **dbVOR** using the publicly-available Illumina HapMap data (Gene Expression Omnibus accession numbers GSE17205 (CEU), GSE17206 (CHB+JPT), and GSE17207 (YRI)) containing 620,932 markers for 73, 75, and 77 samples, respectively [11,12]. It took 132 seconds to load the marker names and positions into the ***markers*** table, ***marker_info*** table, and ***snpblocks*** table. After that, it took 436 seconds to load the 73 CEU samples, 374 seconds to load the 75 CHB+JPT samples, and 364 seconds to load the YRI samples. All these load times should be about the same because they read the **snpblocks** table and then use it to create the genotype blocks for the about the same number of samples. But the time to load the first of the HapMap data sets (CEU) is 62 seconds longer than the rest because, being first, it has to pull the **snpblocks** table into the database server cache whereas when loading the next two datasets the table is read from the server cache.

### Scaling

Currently, each record in the ***Genotypeblocks*** table holds exactly one 500 genotype block and some additional fields. This appears optimal for the data we currently load into dbVOR. We varied the number of genotypes per block from 50 to 5,000 and evaluated the impact on performance and space. The last block for each chromosome is usually not completely full of genotypes, nonetheless space is always reserved for a full block. The extra space is wasted. Our Illumina exome chip data with 247,519 markers has a total of 1,379 samples (stored as one file per sample) but we had pedigree data for only 1,058 individuals. We prefer to use all 1,379 samples for the following experiments. If we scale the previous load time of 2,245 seconds for 1,058 samples by 1379/1058, we would have predicted a load time of 2,926 seconds; we measured 2,901 seconds.

**Figure 4:**
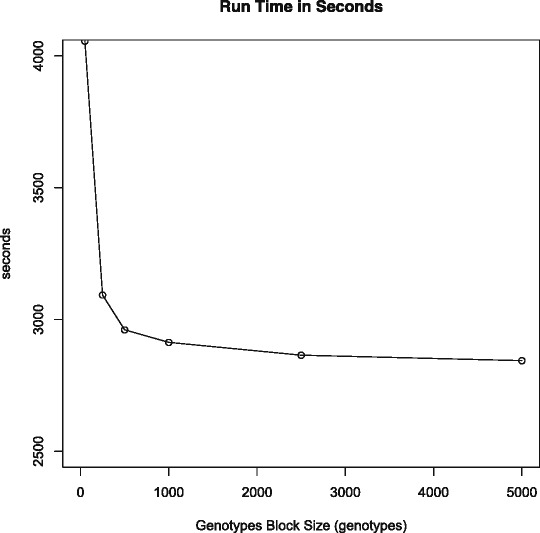
Time to load the *Genotypeblocks* table with 247,519 markers for 1,379 samples as a function of number of genotypes in a block.

Figure 4 shows that time to read the files and populate the database as we vary the genotype block size is relatively flat for more than 500 genotypes per block, but exponentially worse for smaller block sizes. Most of the load time comes from parsing the data file, not inserting the data into the database.

**Figure 5:**
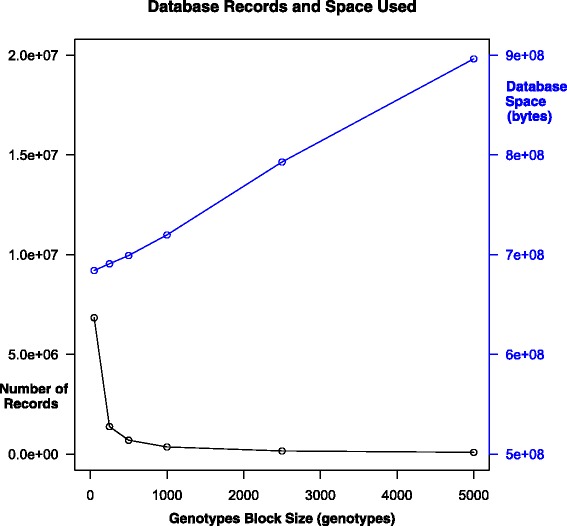
Number of records in the *Genotypeblocks* table and total space usage in bytes for alleles as a function of the number of genotypes per block, as computed on an example data set of 1,379 samples typed at 247,519 markers.

Figure 5 shows a similar looking curve for the number of records in the ***Genotypeblocks*** table. Also, as the number of genotypes per block rises, the larger blocks are not filled up completely since more of the chromosomes will not have enough markers to need a complete block. The unused space is wasted; at 5,000 genotypes per block, the database is over 45 times larger than its minimal size.

## Discussion

A number of database systems for handling human genetics data have been proposed, starting as early as 1988 when the “Human Genetics Database Management System” was developed; this database system handled not only marker data on pedigrees, but also clinical and laboratory data [1]. Before embarking on writing our own database software, we explored other options. As a small research group with limited funds, the capable Integrated Genotyping System [3] was not an option for us because we were not prepared to set up and maintain a Windows server machine. However, we did have a Sun Solaris machine, and so explored using GeneLink [2]. GeneLink was originally developed using Sybase SQL server, but we chose to get the Oracle version working, as we had a site license for Oracle. While we got it working, we found that keeping Oracle secure and updated was a major undertaking, requiring the skills of an experienced database system administrator. We failed at our attempts to extend GeneLink to handle non-integer pedigree IDs - changing the pedigree ID type in the database tables was simple, but adjusting the sophisticated web interface to accommodate this change was beyond our skill set. These experiences led us to focus on creating a database system, modeled on GeneLink, that used open source free components, was easy to install and maintain, and has a simple interface, enhancing the ability to subsequently modify it as needed.

In passing, we note that PLINK [9] is a formidable data analysis tool and it provides at least as much filtering capabilities as **dbVOR**. PLINK can do a variety of tasks - it can recode allele labels, flip strands, update individual information, zero out a set of genotypes, and merge sets of genotypes. However, PLINK only supports flat files; it has no database capabilities. Further, it cannot easily manage collections of experiments to process. Merging data sets with PLINK requires that both data sets use the same sets of person IDs and allele labels, while **dbVOR** allows one to resolve these issues via the use of appropriate aliases. And, having been written primarily for data sets of unrelated individuals, when working with family data it can be challenging with PLINK to avoid filtering out ungenotyped individuals required to maintain family structures. While our final merged and cleaned data sets are often analyzed with PLINK, in our experience **dbVOR** greatly facilitates the process of preparing a single PLINK-ready data set.

We have compared **dbVOR** to SNPpy, a recently developed database system [6,7]. Both SNPpy and **dbVOR** are database systems that store and retrieve genotype information from experiments. Both systems access their database in Python and by a command line interface. They both deal with the large amount of marker data in current genome-wise studies, but in different ways. SNPpy more heavily uses database features and uses C++ code embedded in Python to improve database performance. In addition, SNPpy can take advantage of multiple cores, when available. However, SNPpy supports only one phenotype and no parental information. **dbVOR** is specifically designed for linkage studies containing families, and so stores parent data. It also stores marker genetic positions (as well as base pair positions); in addition, it stores map information for multiple builds. **dbVOR** stores all the data as it was originally sourced from flat files, Illumina files, or Affymetrix files. But it allows for later marker aliasing, person name aliasing, and allele value remapping. It provides statistics to show patterns/anomalies in the data without judging them as errors.

On data extraction, **dbVOR** allows poor data to be marked and ignored. It resolves multiple measurements of the same person/marker in an automated but customizable way. **dbVOR** can filter subsets of the database via simple lists and ranges and does not required the construction of complicated SQL expressions (as is necessary for SNPpy). Further, filtering can be constrained to keep the family structures intact. **dbVOR** reports data out in the less compact text-based PLINK formats like SNPpy does, as well as in the compact binary PLINK format. It also can output Mega2 files [10]. **dbVOR**’s data representation is particularly efficient for fetching all the markers from a given chromosome or chromosome range.

Table 5 compares the Illumina HapMap loading times with the corresponding times for SNPpy. Recall, these data contain 620,932 markers and 73, 77 and 75 samples for CEU, YRI, and CHB+JPT, respectively. We use an iMac with a 3.33 GHz Intel Core 2 Duo processor having 8 GB memory. The **dbVOR** configuration files used in these comparisons are provided in Additional file 3; the process for developing the timing data is described in detail in Additional file 4. The **dbVOR** calculation is done with the genotypes stored in the genome-wide block representation. The SNPpy calculation is done with genotypes stored in the shard representation with multiprocessing activity set to one processor (*j*=1).

**Table 5:** **Performance comparison: average time (in seconds) to insert all the Illumina HapMap data into the database and generate a PLINK “ped” file using dbVOR and SNPpy**

**Task**	**Cold Database**		**Hot Database**	
	**dbVOR**	**SNPpy**	**dbVOR**	**SNPpy**
load all HapMap data	1311	1053	1302	1038
select all 620,932 marker	1333	586	1317	478
genotypes; write to ped file				
select chromosome 1	117	188	98	111
genotypes; write to ped file				
select chromosome 1 genotypes	28	235	8	112
between bp 500,000 to 1,000,000;				
write to ped file				

The timings under Cold Database are for performing a task just after the database server is started. The Hot Database timing are for repeating the task a second (or third) time. The Cold numbers are averaged over three runs and the Hot numbers are averaged over six runs.

Regarding the average loading times seen in the first line of Table 5, SNPpy is faster than **dbVOR**. Similarly, when selecting and writing out all the genotypes, SNPpy is much faster than **dbVOR**. However, when smaller portions of the data are selected, **dbVOR** is faster than SNPpy. The ‘cold’ timings indicate performance right after the database has been started, while the ‘hot’ timings indicate the effect of repeating a task that has previously been carried out. Both systems show improvements in ‘hot’ mode when reading a chromosome or less; presumably because data are cached in the server and so do not have to be fetched from the database files

One might question why our database system contains two different subsystems for storing genotypes: the ***Genotypes*** table is used for storing small volumes of data, while the ***Genotypesblocks*** table approach is used for genome-wide large-scale genotype data. One reason is historical: we developed the ***Genotypes*** table approach first for targeted experiments. The other reason is that in our experience, in a given research project, it is common to start with genome-wide large-scale data and then follow-up with much smaller scale targeted follow-up data. The genome-wide representation is more efficient for reading and writing large-scale genotype data; however, it is less space efficient for targeted data (as storing small-scale data in a large block structure would waste space). The ***Genotypes*** table representation is just the opposite; it is efficient where the genome-wide representation is not and vice versa. The genome-wide representation for reading and writing uses fewer database records. The database must maintain indices, consistency constraints and atomicity of operations for each record. With fewer records, there is less total time overhead.

Finally, it is important to note that efficiency is context dependent: the genome-wide representation is inefficient in the case when one is selecting a small number of markers, each in a separate block. Such a selection would, when using the ***Genotypeblocks*** representation, require extraction and handling of a lot of unneeded marker data, as each block containing a selected marker would need to be pulled in its entirety from the database.

## Conclusions

Our **dbVOR** database provides useful solutions to data management problems commonly encountered during an ongoing study of the genetics of a complex trait. In such a study, oftentimes genome-wide data are first generated, and then later regions of interest are followed up via targeted custom genotyping using a different technology. Differing technologies may return different allele labels for a given marker. Genotyping experiments may partially fail, and so need to be redone. Clinical data may be presented with identifiers that differ from the identifiers used in the laboratory. Accordingly, **dbVOR** was designed to handle data from multiple sources with possibly conflicting measurements as well as conflicting codings when the underlying data are actually the same. It was designed to process not only targeted experiments but also genome-wide experiments and to merge their results into a coherent whole. **dbVOR** allows you to select subsets of the data by several different criteria and to output the results in tabular, Mega2, or PLINK format.

We now enumerate some of the strengths of our **dbVOR** database:

- **dbVOR** was designed to be easy to install, maintain, and modify. It is purposely lean, requiring just two software components (Python and MySQL), and it is open source.
- Our **dbVOR** database handles both genome-wide scale data, as well as targeted follow-up data of limited scale. While the genome-wide data are stored in a special way that maximizes efficiency, our database is capable of merging the two types of data together during export.
- **dbVOR** handles not only unrelated individuals, but also handles family data. It can keep family structures intact after filtering.
- Our database is capable of handling multiple different phenotypes, both categorical and quantitative, and provides summary statistics.
- **dbVOR** is designed to handle the reconciliation of overlapping genotype data from different experiments. In the process, it provides feedback to the user in the form of agreement matrices, clearly showing how many repeated genotypes agreed with each other and how many did not.
- Our database can resolve multiple ID systems, alternate marker names, and differing marker allele labels.
- **dbVOR** retains all original data while providing the ability to selectively turn off untrusted portions of the data.
- Our database supports flexible and powerful filtering during export. For example, specific pedigrees or persons can be excluded or included, and markers can be selected by name, chromosomal position, or chromosomal range.
- **dbVOR** supports the storage of multiple builds of map information, as well as supports genetic maps.
- Our database uses a command-line interface, augmented with flexible configuration files.
- **dbVOR** has the ability to do a test run of requested actions before committing such changes to the database. This permits iterative improvement to the choice of configuration options.
- **dbVOR** provides a system for storing and retrieving notes. The data analyst can use these notes to document data cleaning decisions and data manipulation choices.
- **dbVOR** supports Illumina, Affymetrix, and flat file input, largely by letting the user choose columns from the input files. It also supports input in PLINK binary ’bed’ format.

As with all software projects, **dbVOR** is a work in progress, and so there are a number of possible improvements that could be made in the future. These include:

- **dbVOR** could be extended to use multiple cores in parallel when available. Such an extension should enable the speed up of some currently slow operations.
- It might be useful to add support for data constraint checking, mainly for phenotype data.
- **dbVOR** could be extended to provide easy control of database user privileges, permitting us to supplement administrator roles with more limited reader and specialized roles.
- Our database could be extended to store and manage intensity data generated by genome-wide chips.
- While **dbVOR** can currently handle imputed genotypes that have been called, it could be extended to store and manage uncalled imputed genotypes which are represented by a trio of posterior genotype probabilities.

## Availability

Current versions of our dbVOR and GMI programs are attached as additional files. Updated stable releases will be available, after a simple registration, at our software registration site https://watson.hgen.pitt.edu/register. Our current working version of dbVOR can be found at the dbVOR BitBucket repository: https://bitbucket.org/dweeks/dbvor/, where no registration is required.

## Availability and requirements

**Project name:dbVORProject home page:**https://watson.hgen.pitt.edu/register**Operating system(s):** Platform independent**Programming language:** Python 2.6.x, 2.7.x**Other requirements:** MySQL, Python MySQL module**License:** GNU GPL 3**Any restrictions to use by non-academics:** None beyond those in the GNU GPL 3 license

## Appendix

### Data schema

**Figure 6:**
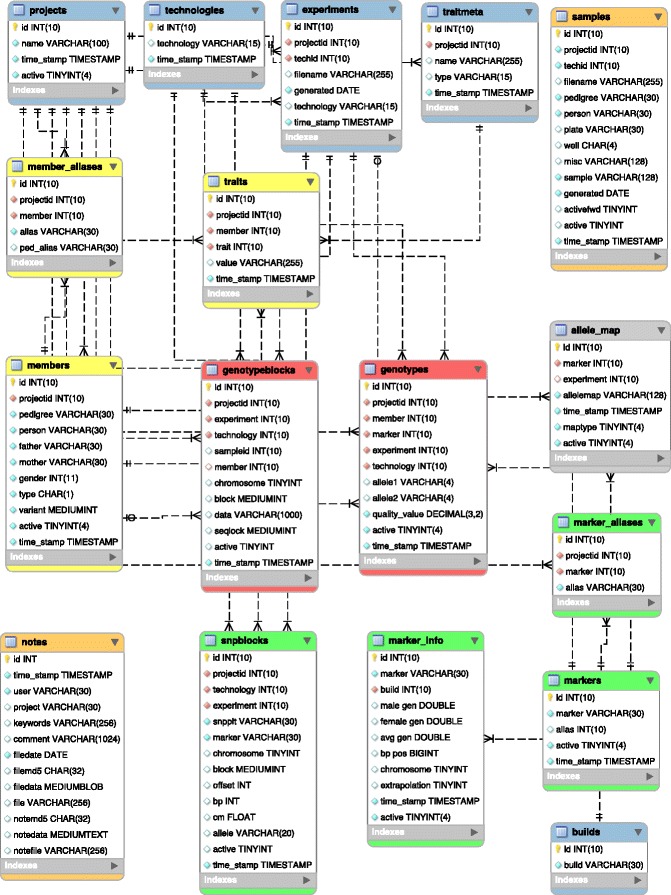
The dbVOR schema. The blue tables (*projects*, *technologies*, *experiments*, *traitmeta*, and *builds*) map a string name to a database identifier and occasionally supply additional data. The yellow tables (***members***, ***member_aliases*** *,* and ***traits***) contain pedigree and phenotype data. The red tables (***genotypes*** and ***genotypeblocks***) contain the genotype data. Each record in ***genotypes*** contains the data for a member and single marker, while a record in ***genotypeblocks*** contains a block of data for a member and several hundred contiguous markers. The green table (***snpblocks***) provides an index into ***genotypeblocks***, specifying where a particular marker is in a block of contiguous markers. The green tables (***markers***, ***marker_aliases***, and ***marker_info***) map a marker name to a database identifier and supply map position information. The ***allele_map*** table optionally remaps the allele names for a specified experiment and marker to different values. The ***samples*** table stores information about samples, the most important being the mapping from the sample identifier to the pedigree and person of the subject. The ***notes*** table provides a facility for documenting decisions made as the data are cleaned and processed.

Figure 6 is an enhanced entity-relationship diagram illustrating the layout of dbVOR’s database tables, as drawn by MySQL Workbench using Crow’s Foot Notation. Here the boxes represent tables, the lines between the boxes represent relationships between the tables, and cardinality and modality of these relationships is represented by the shapes at the ends of the lines. However, the lines are not useful in this static presentation. But if MySQL Workbench were interactively presenting this schema, hovering over table fields and arrows would clearly highlight foreign key dependencies. Each field is annotated by a key symbol or diamond symbol whose meanings are defined in Table 6.

**Table 6:** **Definitions of the field-specific symbols used in Figure** 6

**Symbol**	**Meaning**
Small key	Primary key
Red diamond	Foreign key
Filled diamond	Not null

## Additional files

Additional file 1
**Contains the entire dbVOR package, version 1.11.**

Additional file 2
**Contains the entire Genetic Map Interpolator (GMI) package, version 1.4.**

Additional file 3
**Contains the configuration files and some data files for dbVOR vs SNPpy performance comparisons.**

Additional file 4
**Contains additional documentation regarding the details of how we carried out the performance measurements.**

## Abbreviations

GMI: Genetic Map Interpolator
ID: Identifier
CEPH: Centre d’Etude du Polymorphisme Humain
CEU: CEPH (Utah residents with ancestry from northern and western Europe)
CHB: Han Chinese in Beijing, China
JPT: Japanese in Tokyo, Japan
YRI: Yoruba in Ibadan, Nigeria

## References

[1] Seuchter SA, Skolnick MH (1988). HGDBMS: a human genetics database management system. Comput Biomed Res.

[2] Gillanders EM, Masiello A, Gildea D, Umayam L, Duggal P, Jones MP (2004). GeneLink: a database to facilitate genetic studies of complex traits. BMC Genomics.

[3] Fiddy S, Cattermole D, Xie D, Duan XY, Mott R (2006). An integrated system for genetic analysis. BMC Bioinf.

[4] Yeung JM, Sham PC, Chan AS, Cherny SS (2008). OpenADAM: an open source genome-wide association data management system for Affymetrix SNP arrays. BMC Genomics.

[5] Fong C, Ko DC, Wasnick M, Radey M, Miller SI, Brittnacher M (2010). GWAS analyzer: integrating genotype, phenotype and public annotation data for genome-wide association study analysis. Bioinformatics.

[6] Mitha F, Herodotou H, Borisov N, Jiang C, Yoder J, Owzar K (2011). SNPpy–database management for SNP data from genome wide association studies. PLoS One.

[7] Mitha F (2013). Managing large, SNP datasets with SNPpy. Methods Mol Biol.

[8] Mukhopadhyay N, Tang X, Weeks DE. Genetic Map Interpolator. Paper presented at the annual meeting of the American Society of Human Genetics. Washington D.C. 2010. http://www.ashg.org/2010meeting/abstracts/fulltext/f22529.htm. Accessed 27 Feb 2015.

[9] Purcell S, Neale B, Todd-Brown K, Thomas L, Ferreira MAR, Bender D (2007). PLINK: a tool set for whole-genome association and population-based linkage analyses. Am J Hum Genet.

[10] Mukhopadhyay N, Almasy L, Schroeder M, Mulvihill WP (2005). Weeks DE. Mega2: data-handling for facilitating genetic linkage and association analyses. Bioinformatics.

[11] International HapMap Consortium (2005). A haplotype map of the human genome. Nature.

[12] International HapMap Consortium, Frazer KA, Ballinger DG, Cox DR, Hinds DA, Stuve LL (2007). A second generation human haplotype map of over 3.1 million SNPs. Nature.

