# Supplementary material for: dbVOR: a database system for importing pedigree, phenotype and genotype data and exporting selected subsets
Source: BMC Bioinformatics. 2015 Mar 18;16(1):91. doi: 10.1186/s12859-015-0505-4 (PMC4407391; doi:10.1186/s12859-015-0505-4)
Supplement: Additional file 4 — Contains additional documentation regarding the details of how we carried out the performance measurements. [file 12859_2015_505_MOESM4_ESM.pdf]

dbVOR: Performance Measurement Details

Supplemental Information for the paper:
"dbVOR: A database system for importing pedigree, phenotype and genotype data and exporting selected subsets"

January 30, 2015

Robert V. Baron[1], Yvette C. Conley[2], Michael B. Gorin[3], Daniel E. Weeks[1,4]

[1]Department of Human Genetics, Graduate School of Public Health, University of Pittsburgh, Pittsburgh, Pennsylvania, 15261, USA

[2]Department of Health Promotion and Development, School of Nursing, University of Pittsburgh, Pittsburgh, Pennsylvania, 15261, USA

[3]Department of Ophthalmology, David Geffen School of Medicine, University of California Los Angeles, Los Angeles, California, USA; Jules Stein Eye Institute, Los Angeles, California, 90095, USA

[4]Department of Biostatistics, Graduate School of Public Health, University of Pittsburgh, Pittsburgh, Pennsylvania, 15261, USA

Email: Robert V. Baron - rvb5@pitt.edu, Yvette C. Conley - yconley@pitt.edu, Michael B. Gorin - gorin@jsei.ucla.edu, Daniel E. Weeks - weeks@pitt.edu

1 Setup for Performance Measurements

The data we used and reported on in the Results section of our paper came from an Illumina HumanExome-12v1 exome chip data set of 1,058 samples genotyped at 247,519 markers by the Center for Inherited Disease Research. The data were generated as part of our Genetics of Age-related Macular Degeneration study and we are not able to freely share these data because of privacy concerns. However, while examining the SNPpy program for our **dbVOR** paper, we discovered an available Illumina data set that we also used for timing and can share. We want to thank the SNPpy author, Faheem Mitha, for finding this data set. These data are available from the ftp.ncbi.nih.gov site corresponding to Gene Expression Omnibus accession numbers: GSE17205 (CEU), GSE17206 (CHB+JPT), and GSE17207 (YRI). We give instructions below how to download and process these data to generate the performance results. We hope that these instructions are clear.

Overview

First, you should create a directory to store the data that we will untar and/or fetch for these measurements. The name does not matter.

We provide a .tgz file, Hapmap.tgz, that contains the three phenotype files described below in the **Family Data** section as well as **dbVOR** configuration files. The Hapmap and Plate data are rather large; you will need to use the **wget** commands described below to fetch them.

"Change directory" to the new directory and copy the Hapmap.tgz file to it. To unzip the Hapmap.tgz file, type:

```
tar zxvf Hapmap.tgz
```

HapMap Data

Fetch

Here we will show you how to fetch the data and later we will show how to use it for **dbVOR** and for SNPpy. First, fetch GSE17205, GSE17206 and GSE17207. In the new directory type:

```
wget -c ftp://ftp.ncbi.nih.gov/pub/geo/DATA/supplementary/series/GSE17205/GSE17205_Human610-Quadv1_CEU_Final_Call_Report.csv.gz
wget -c ftp://ftp.ncbi.nih.gov/pub/geo/DATA/supplementary/series/GSE17206/GSE17206_Human610-Quadv1_CHB+JPT_Final_Call_Report.csv.gz
wget -c ftp://ftp.ncbi.nih.gov/pub/geo/DATA/supplementary/series/GSE17207/GSE17207_Human610-Quadv1_YRI_Final_Call_Report.csv.gz
```

(**wget** is a generally available utility for fetching web pages. All the text after **wget** should be on a single line.)

checksum and size

The files' md5 checksums should be:

```
9654f79def058020b7e2c41ce8e891a1 GSE17205_Human610-Quadv1_CEU_Final_Call_Report.csv.gz
6846fcce6fb8940e840121c7d26e76e4 GSE17206_Human610-Quadv1_CHB+JPT_Final_Call_Report.csv.gz
a97ab3f7b043de603603b9faba76a260 GSE17207_Human610-Quadv1_YRI_Final_Call_Report.csv.gz
```

The compressed file sizes are almost 1 Gigabyte:

```
967,509,110 GSE17205_Human610-Quadv1_CEU_Final_Call_Report.csv.gz
992,630,807 GSE17206_Human610-Quadv1_CHB+JPT_Final_Call_Report.csv.gz
1,022,864,215 GSE17207_Human610-Quadv1_YRI_Final_Call_Report.csv.gz
```

unzip

We need to "unzip" these files for subsequent processing. So we will type:

```
gunzip *.gz
```

These uncompressed files have a format that is reminiscent of PLINK lgen format with additional frequency and scoring information on each line. The header reads:

```
SNP Name,Sample ID,Allele1 - Forward,Allele2 - Forward,GC Score,X,Y,X Raw,Y Raw,Log R Ratio,B Allele Freq
```

Plate Data

An annotation file, Human610-Quadv1_B.csv, is available from Illumina by typing (on one line):

```
wget -c --ftp-user=guest --ftp-password=illumina ftp://ftp.illumina.com//Whole%20Genome%20Genotyping%20Files/
Archived_Human_Products/Human610-Quad_v1_product_files/Human610-Quadv1_B%20files/Human610-Quadv1_B.csv
```

Family Data

The SNPpy distribution provides phenotypes in its directory data/Hapmap610/Pheno in the three files: ceupheno.csv, chbjptpheno.csv and yripheno.csv. They are included in our Hapmap.tgz file with the permission of Faheem Mitha, the author of SNPpy. We will use these phenotype files for SNPpy and for **dbVOR**. Each file looks like a PLINK .fam file with an additional 'race' column.

2 dbVOR processing

A few conventions are necessary to setup **dbVOR**. We will call the database **hapmap**, the user will be **watson** with password **crick**. The project will be "**project**", the technology will be **Illumina** and the experiments will be **CEU**, **YRI** and **CHBJPT**. Note also that the **dbVOR** manual is available online at `https://watson.hgen.pitt.edu/register/docs/dbVORtutorial.html`.

Start MySQL

Assume that MySQL is loaded into ***/usr/local/mysql***. We will be using the InnoDB database engine. So copy the file, ***/usr/local/mysql/support-files/my-innodb-heavy-4G.cnf*** to ***/etc/my.cnf***. Then to start the database server become root, and type:

```
mysqld_safe -D --user=_mysql &
```

_mysql should be replaced with the user id that the database server should run under if it is not **_mysql**.

Create Samples file

dbVOR requires a description of the samples being analyzed; in particular it needs lines with the fields:

```
subject_id,active_value,pedigree,person,plate,well,misc,sample_id
```

we do not have all of this information available. So we will leave the plate, well, and misc fields empty; they would be necessary for data cleaning. In addition, we do not have a true subject id or sample id, but we do observe that the person field is unique. So we will use the person field repeatedly. The person and pedigree link the sample to the subject. The sample_id is the lab name of the sample that will be found in the data received from the lab. The subject_id is a simple familiar name of the person that would be more important if the person field were not unique. There can be multiple samples for some subjects. We will make a simulated sample file by taking lines in the ceupheno.csv file, for example:

```
4,NA18500,3,2,1,0,YRI
```

and using the first two fields to generate a corresponding entry for the sample file, ceusamples.csv:

```
NA18500,1,4,NA18500,,,,NA18500
```

Create Database

First, to create the database **hapmap** that will be owned by the user **watson** with the password **crick**, we will run

```
dbvor create --sqlpath=whereIputdbVOR/sql HapmapCreateDB.cfg
```

where "*whereIputdbVOR*" should be replaced with the path where **dbVOR** was installed. You can find out more in the **dbVOR documentation**. The contents of the .cfg file should be obvious; see the **dbVOR manual** for further explanation. You will be prompted for the database root password and later for a personal database password; the latter should be set to **crick**.

Add Members

Next, we will load a set of members. (We will illustrate this and later concepts for CEU, but the file formats and hence most of the data in the .cfg files will be the same for each data set. Note: ceuMembers.cfg references the ceupheno.csv file, while the yriMembers.cfg file references yripheno.csv, etc.) The ceupheno.csv file is a standard .fam file with an extra column. (The yripheno.csv and chbjptpheno.csv have the same format as ceupheno.csv.) We need the pedigree, person and sex columns. We would like the father and mother information but as we currently do not understand the semantics of the values used, so we will ignore them. To setup a .cfg file, we supply: **input**, **sep**, **pedigree**, **person** and **gender** arguments with the values: ceupheno.csv, ",", 1, 2, and 5. Specifying a separator, **sep**, is necessary because the file does not use white space between values. This file is supplied as ceuMembers.cfg in the Hapmap.tgz file; so we type:

```
dbvor Member ceuMembers.cfg -v --commit
```

The command should be repeated for the other data sets:

```
dbvor Member yriMembers.cfg -v --commit
dbvor Member chbjptMembers.cfg -v --commit
```

Typically after experiments are established, the members do not change so we do not expect to run this command again. (Of course, we can if we need to add new members.)

Add Samples

The ceuSamples.cfg file specifies where the input samples file is and that it is in "csv" format. The samples are loaded by typing:

```
dbvor Sample ceuSamples.cfg -v --commit
```

The command should be repeated for the other data sets:

```
dbvor Sample yriSamples.cfg -v --commit
dbvor Sample chbjptSamples.cfg -v --commit
```

Add Markers

We are almost ready to load data. But we need to describe the markers on the plate. These data are in the Human610-Quadv1_B.csv file. We read the header which contains:

```
IlmnID,Name,IlmnStrand,SNP,AddressA_ID,AlleleA_ProbeSeq,AddressB_ID,AlleleB_ProbeSeq,GenomeBuild,Chr,MapInfo,
Ploidy,Species,Source,SourceVersion,SourceStrand,SourceSeq,TopGenomicSeq,BeadSetID,Intensity_Only,
Exp_Clusters,CNV_Probe
```

and select out the **Name**, (column 2), the **Chr**(omosome) [column 10], the **MapInfo** [base pair position] (column 11) and the **SNP** [alleles] (column 4). We enter these keyword-values into ceuMarkers.cfg, and indicate the file is "csv" format. In further examining the file, we observed that the mapping information came from build 36; thus we add the argument "build" with the value GB36. In addition, we must supply the arguments **make_snps**, and **snp_db_option** the latter with the value True and the former with no value to the ceuMarkers.cfg file.

Finally, there are extra data in the Human610-Quadv1_B.csv file that dbVOR does not process. In the current file, there are 7 lines before the header line that must be removed; in addition there are 23 lines starting with a line '>"–control–" at the end of the file that are spurious and also must be removed. We will create a new file without these extra data, Human610-Quadv1_BB.csv and use it for dbVOR. Now we type:

```
genoi ceuMarkers.cfg -v --commit
```

We run this command only once, not for each data set, because it describes the plate technology which is the same for all three data sets.

Add Data

Now it is time to load the data. We now look at the data file header:

```
SNP Name,Sample ID,Allele1 - Forward,Allele2 - Forward,GC Score,X,Y,X Raw,Y Raw,Log R Ratio,B Allele Freq
```

For the configuration file, we need the **name** [SNP Name] (column 1), the **sample** [Sample ID] (column 2), **allele1** [Allele1 - Forward] (column 3), **allele2** [Allele2 - Forward] (column 4) and **call** [GC Score] (column 5). We also need some boiler plate to indicate the "**inputdir**" (.), the field separator [**sep**] (","), the data file name (**input**) and an indicator that there are multiple samples in the input file (**multi**). In addition, we indicate that the markers come from the database and that the results are stored in the database: **snp_db_input** and **db_output**. We put all these arguments in a configuration file and then type:

```
genoi ceuData.cfg -v --commit
```

The command should be repeated for the other data sets:

```
genoi yriData.cfg -v --commit
genoi chbjptData.cfg -v --commit
```

The yriData.cfg and chbjptData.cfg files are provided. Note: For any **dbVOR** program run, we print out the start time, end time, and duration of the execution. Of course, you may prefer using the Unix **time** command for timing.

Extract data

We will fetch all the genotypes, the genotypes for chromosome 1 and the genotypes for chromosome 1 between base pair 500,000 and 1,000,000. The basic configuration file will supply:

argument	value
**project**	project
**experiments**	CEU
**experiments**	YRI
**experiments**	CHBJPT
**dir**	.
**type**	ped
**person_order**	1

You will also need to supply a unique **log** file name and **output** as a stem for any generated files. We provide several sample configuration files: out1.cfg, out2.cfg, out3.cfg, and out4.cfg. The first is an exception and uses **type** tped with no person_order. The rest generate the standard PLINK ped file and map file. out2.cfg generates the files for all the genotypes. out3.cfg adds the argument **chromosome** value **1** to filter for just chromosome 1. out4.cfg uses the argument **chrrange** value **1:500000..1000000** to fetch the subrange of chromosome 1. The program **genout** generates genotype output; you type:

```
genout out1.cfg
genout out2.cfg
genout out3.cfg
genout out4.cfg
```

3 SNPpy processing

Fetch SNPpy

SNPpy is described in:

Mitha F, Herodotou H, Borisov N, Jiang C, Yoder J, Owzar K. (2011) SNPpy - Database Management for SNP Data from Genome Wide Association Studies. PLoS ONE 6(10): e24982. doi:10.1371/journal.pone.0024982 `http://www.ncbi.nlm.nih.gov/pubmed/22039405`

The SNPpy source code can be obtained from its Bitbucket repository `https://bitbucket.org/faheem/snppy/` by using the Mercurial source code management system (`http://mercurial.selenic.com/`), and executing the command

```
hg clone http://bitbucket.org/faheem/snppy
```

This will create a directory named, **snppy**, in the current directory that contains the SNPpy sources.

Fetch Prerequisites

The file docs/MANUAL in the **snppy** directory explains how to load the prerequisites for SNPpy, how to load the example data and how to run SNPpy. I will give you my experiences as to how to create SNPpy and get its prerequisites, but I have only done this for a Mac and doubt the exact instructions would be the helpful for a different architecture. After several missteps, I finally settled on the MacOS "brew" package system and the python pip installation program to provided functioning prerequisite programs for SNPpy.

(If brew is not on your Mac, check out `http://brew.sh/`.) After brew is installed:

For boost, run:

```
brew install boost --c++11 --with-icu --with-python --without-single --without-static
```

For scons, run:

brew install scons

For postgres, run:

```
brew install postgresql
```

I use python 2.6. (I do not know if python 3.x would work.) Unfortunately, python 2.x does not include the package installer program, pip. This must be downloaded from python.org, and is called get-pip.py. Now we proceed with the python installations, so

To install pip, run:

```
python get-pip.py
```

Then for SQLAlchemy, run:

```
pip install sqlalchemy
```

For ConfigObj, run:

```
pip install configobj
```

For Psycopg, run:

```
pip install psycopg2
```

For (optional) Numpy, run:

```
pip install numpy
```

Massaging Data

For SNPpy, you must use a conversion program from the SNPpy distribution on the fetched Hapmap data, *convert_illumina_hapmap.py*. This removes uninteresting fields and inverts the data files into a PLINK tped file with a header. When I ran it on my hardware I got the timing numbers indicated below. *convert_illumina_hapmap.py* is located in the **snppy** directory tree; run it by typing (each command is one one line):

```
python convert_illumina_hapmap.py GSE17205_Human610-Quadv1_CEU_Final_Call_Report.csv
GSE17205_Human610-Quadv1_CEU_Final_Call_Report.converted.csv
python convert_illumina_hapmap.py GSE17206_Human610-Quadv1_CHB+JPT_Final_Call_Report.csv
GSE17206_Human610-Quadv1_CHB+JPT_Final_Call_Report.converted.csv
python convert_illumina_hapmap.py GSE17207_Human610-Quadv1_YRI_Final_Call_Report.csv
GSE17207_Human610-Quadv1_YRI_Final_Call_Report.converted.csv
```

The program reports execution times as, respectively:

convert took 287.850 s ~ 4 min and 47.9 sec

convert took 289.715 s ~ 4 min and 49.7 sec

convert took 292.283 s ~ 4 min and 52.3 sec

SNPpy preliminaries

The above converted Hapmap data collections should be moved to the data/Hapmap610 directory in the **snppy** directory. The original Human610-Quadv1_B.csv file should also be placed in the same directory

In the ***default_conf*** file, all file paths that contain "data/snppy/Hapmap610" must be modified to read "data/Hapmap610" instead. In addition, you might want to create a **user_conf** with your **dbuser** and **password** entries.

The first attempt to run load_dataset.py (below), will compile some C++ code. For this to work correctly on the Mac with brew, two changes have to be made. In the file SConstruct (in the installation directory), there is a line that defines "LIBS"; one library in that list is "boost_python". That Library must be changed to "boost_python-mt" instead ,viz.

```
LIBS=["python"+pyversion(), "m", "boost_python-mt"],
```

Secondly, the dynamic library must be called genocpp.so vs genocpp.dylib for python on the Mac. The simplest way to do this is to create a symbolic link, viz:

```
ln -s genocpp.dylib genocpp.so
```

Start Postgres

/usr/local/Cellar/postgresql/9.3.4/NOTES, explains how to start postgres. Typing

```
postgres -D /usr/local/var/postgres
```

will suffice.

Running SNPpy

Following the docs/MANUAL instructions, you might try to create a database to make sure the data load will work (run from the installation directory, **snppy**).

```
python init_db.py test illumina
```

The commands to load the data are:

```
python load_dataset.py -sy ceu test -a forward
python load_dataset.py -sy yri test -a forward
python load_dataset.py -sy chbjpt test -a forward
```

The SNPpy MANUAL explains what these commands do.

SNPpy data extraction

We will fetch all the genotypes, the genotypes for chromosome 1 and the genotypes for chromosome 1 between base pair 500,000 and 1,000,000. The first set is generated simply by running the commands below. (The -s indicates that test contains shard data.):

```
python make_output.py -s ped ceu yri chbjpt test -a forward
python make_output.py -s map ceu yri chbjpt test -a forward
```

To request genotypes for only chromosome 1, the **default_conf** file should be edited. Under the grouping for ceu (labeled [[ceu]] in the file), add the line

```
anno_filter = "chromosome = 1"
```

Also add this same line to the yri and chbjpt groupings. Then repeat the commands:

```
python make_output.py -s ped ceu yri chbjpt test -a forward
python make_output.py -s map ceu yri chbjpt test -a forward
```

But first, you must delete the output of the previous command. So type:

```
rm test.ceu_shard-yri_shard-chbjpt_shard.forward.ped
```

To request the genotypes for only base pairs between 500,000 and 1,000,000 on chromosome 1 a similar edit is necessary. The filter line in the **default_conf** file should now read:

```
anno_filter = "chromosome = 1 and location between 500000 and 1000000"
```

all three lines should be updated. Afterwards type:

```
python make_output.py -s ped ceu yri chbjpt test -a forward
python make_output.py -s map ceu yri chbjpt test -a forward
```

Again, you must delete the output of the previous command. So type:

```
rm test.ceu_shard-yri_shard-chbjpt_shard.forward.ped
```

4 Hardware and Experimental Setup

All experiments were performed on an iMac with a 3.33 GHz Intel Core 2 Duo processor having 8 GB memory running OS X 10.8 (Mavericks). To minimize variance in the measurements, the backup and the indexing services were disabled. Furthermore, the display was not used for login; rather, all activities were performed via a **ssh** shell. A typical run was: 1. reboot the machine, 2. start the database server, 3. perform an activity, 4. perform the activity again, and 5. perform the activity one last time. This template completely describes the select tests. For data insertion, each activity (step 3, 4, and 5) consisted of deleting the data schema from the server and then loading the three data sets. (The server is never reinitialized after a reboot.)

The **dbVOR** calculations are done with the genotypes stored in the genome-wide block representation. The SNPpy calculations are done with genotypes stored in the shard representation with multiprocessing activity set to one processor (j=1). NOTE: The configuration file shown under **dbVOR** for generating a tped PLINK file is not available for shard format data.

5 Performance Results

Table 1 provides more detailed performance results than the summary results provided in the main paper. The CEU, YRI, and CHB+JPT test data sets contain 73, 77 and 75 samples, respectively with 620,932 markers per sample. Note that here we use the times printed by the programs themselves, which do not measure the complete execution time; in our measurements, they are too short by about half a second.

Regarding the average loading times seen in the first section of Table 1, SNPpy is faster than **dbVOR**. Notice that for **dbVOR**, the time to insert each dataset should be rather similar. The first insert spends about 88 seconds reading the ***snpblocks*** information from the database. Each subsequent insert also reads the same information but it is cached in the database server and takes only about 9 seconds. By contrast, the SNPpy timings for insert appear to take progressively more time as each dataset inserted.

When selecting and writing out all the genotypes, SNPpy is much faster than **dbVOR** (Table 1). However, when smaller portions of the data are selected, **dbVOR** is faster than SNPpy.

The 'cold' timings indicate performance right after the database has been started, while the 'hot' timings indicate the effect of repeating a task that has previously been carried out. Both systems show improvements in 'hot' mode when reading a chromosome or less; presumably because data are cached in the server and so do not have to be fetched from the database files. Note that, for data selection, the hot database numbers might not be terribly realistic, but looking at the cold database numbers, it appears as though the **dbVOR** approach to representing genome-wide data in blocks yields a performance advantage.

Table 1: Performance comparison: average time (in seconds) to insert all the HapMap data into the database and generate a PLINK ped file using **dbVOR** and SNPpy. The timings under Cold Database are for performing a task just after the machine is booted and the database server is started. The Hot Database timing are for repeating the task a second (or third) time. The Cold numbers are averaged over three runs and the Hot numbers are averaged over six runs.

**Task**	**Cold Database**		**Hot Database**	
	**dbVOR**	**SNPpy**	**dbVOR**	**SNPpy**
insert 620,932 markers	132	36[a]	132	37[a]
insert CEU genotypes: 73 samples	436	285[b]	435	284[b]
insert YRI genotypes: 77 samples	379	306[b]	371	299[b]
insert CHB+JPT genotypes: 75 samples	364	345[b]	364	345[b]
Total time to insert all HapMap data	**1311**	**1053**[c]	**1302**	**1038**[c]
select all 620,932 marker genotypes; write to ped file	1333	586	1317	478
select chromosome 1 genotypes; write to ped file	117	188	98	111
select chromosome 1 genotypes between bp 500,000 to 1,000,000; write to ped file	28	235	8	112

[a]Time to make the marker annotation table.
[b]SNPpy time after subtracting the time to load the marker annotation table.
[c]This is total time to load all three data sets; SNPpy loads the marker annotation three times, once for each data set.
