# Supplementary material for: dbVOR: a database system for importing pedigree, phenotype and genotype data and exporting selected subsets
Source: BMC Bioinformatics. 2015 Mar 18;16(1):91. doi: 10.1186/s12859-015-0505-4 (PMC4407391; doi:10.1186/s12859-015-0505-4)
Supplement: Additional file 1 — Contains the entire dbVOR package, version 1.11. [file 12859_2015_505_MOESM1_ESM.zip › 1652824117132674_add1.tgz!/dbvor.1.11/doc/Book/dbVORtutorial.pdf]

dbVOR: A database system for importing pedigree, phenotype and genotype data and exporting selected subsets

Robert V. Baron and Daniel E. Weeks

February 16, 2015

Department of Human Genetics, University of Pittsburgh

Copyright © 2010-2015 by the University of Pittsburgh

Current Programmer: *Robert V. Baron*

Note: *The page* `https://watson.hgen.pitt.edu/register/docs/dbvor.html` *contains links to this document in PDF as well as in HTML format and it also contains links in other dbVOR specific materials.*

Contents

1 Introduction **12**

1.1 Organization of this Document 12

1.2 Motivation 12

1.3 Prerequisites 14

1.4 Download and Install dbVOR 14

1.4.1 Install prerequisites 14

1.4.2 Download and decompress dbVOR 15

1.4.3 Installation of dbVOR as a Python application 15

1.4.4 Note and record the path to the 'sql' folder for later use 15

1.4.5 Create a dbVOR database 15

1.4.6 Add information into the database 16

1.5 Citing dbVOR 16

1.6 Support, bug reports, and feedback 16

1.6.1 dbVOR Google Group 16

1.6.2 Bug reports 16

1.7 Contact information 17

1.8 Acknowledgments 17

2 Tutorial **18**

2.1 Introduction 18

2.2 Overview of dbvor commands 18

2.3 Creating a database 20
2.3.1 Using the .dbvorrc configuration file 21
2.4 Populating the database 22
2.4.1 Adding Pedigree Data using dbvor member 24
2.4.2 Adding Trait Data using dbvor trait 29
2.4.3 Adding targeted marker data using geno 31
2.4.4 Retrieving and exporting data using genout 33
2.4.5 Adding Marker Position Data using GMI 34
2.4.6 More GMI Marker Position Data 37
2.4.7 Manually adding Marker Position Data and Aliasing Data 38
2.5 Adding Additional Data 39
2.5.1 Adding more Pedigree Data 39
2.5.2 Adding additional marker data using geno 40
2.5.3 Merged Markers / Marker Aliases 41
2.5.4 Member Aliases 41
2.5.5 Another trait 44
2.5.6 Merging Experiments 44
2.5.6.1 Dry run 44
2.5.6.2 Verify Run 47
2.5.6.3 Verbose Verify Run 50
2.5.6.4 Commit 52
2.5.6.5 More Pesky Details 52
2.5.7 Problems during Merging Markers 53
2.5.7.1 Lets look at a block of output. 55
2.5.7.2 Using the concordance matrix 56
2.5.7.3 Resolvable Disagreements 57
2.5.7.4 Unresolved Disagreements 59
2.5.7.5 Final commit 59

2.5.8 Coda 60

2.5.8.1 Restricting Pedigree and Persons 60

2.5.8.2 Splitting up the configuration file 61

2.5.8.3 Changing the log file name 61

2.5.8.4 Less log output 61

2.6 genout: Data Extraction 61

2.6.1 Data Summary 61

2.6.2 Controlling Output Format 62

2.6.3 Resolving Data Conflicts 63

2.6.3.1 Setting “active” flag 63

2.6.4 A Selection Example 65

2.6.5 Selector 68

2.6.5.1 Select by marker, chromosome, etc. 69

2.6.5.2 Select by member 69

2.6.5.3 Select by trait 70

2.6.5.4 Select by 70

2.6.6 Filtering Experiment Data 71

2.6.7 Further Output Customization 74

2.6.8 Modified operations for genout 76

2.6.9 genout Statistics 76

2.7 Adding genome-wide data 77

2.7.1 Genome-wide HapMap Example 78

2.7.1.1 Family Data 79

2.7.1.2 Plate Data 79

2.7.1.3 Hapmap Data 79

2.7.1.3.1 checksum and size 79

2.7.1.3.2 unzip 80

2.7.1.3.3 Summary 80

2.7.2 Genome-wide Prerequisites 80
2.7.2.1 Create a database 80
2.7.2.2 Add Members for the HapMap Example 81
2.7.3 Genome-wide Samples 82
2.7.3.1 Create Samples for the HapMap Example 83
2.7.3.2 Add Samples for the HapMap Example 84
2.7.4 genoi: Simple genome-wide data (Illumina) 85
2.7.4.1 Simple GWAS Markers 85
2.7.4.1.1 Add Markers for the HapMap Example 86
2.7.4.2 Simple GWAS Data 88
2.7.4.2.1 Add Data for the HapMap Example 89
2.7.5 genoa: Genome-wide Data (Affymetrix) 90
2.7.5.1 GWAS Samples Revisited – Affymetrix samples 91
2.7.5.2 Affymetrix GWAS Markers 91
2.7.5.3 Affymetrix GWAS Data 92
2.7.6 genok: PLINK binary (bed) data files 94
2.7.6.1 Processing Family Data using dbvor plink 95
2.7.6.2 PLINK genome-wide Markers 97
2.7.6.3 PLINK bed genome-wide genotype data 98
2.7.6.4 Adding additional genome-wide data 99
2.7.7 Deleting and reentering genome-wide data 100
2.7.7.1 dbvor DeleteBlockSample 100
2.7.7.2 dbvor DeleteBlockMember 101
2.7.7.3 dbvor DeleteBlockAllSamples 101
2.7.7.4 dbvor DeleteSnpBlockMap 101
2.7.7.5 dbvor ShowSamples 102

3 Manual **103**

3.1 Introduction 103

3.2 General Arguments 103

3.2.1 Required Arguments 105

3.2.2 Common Arguments 105

3.3 dbVOR Commands 109

3.3.1 dbvor active 109

3.3.1.1 NAME 109

3.3.1.2 SYNOPSIS 109

3.3.1.3 ARGUMENTS 109

3.3.2 dbvor allelemap 111

3.3.2.1 NAME 111

3.3.2.2 SYNOPSIS 111

3.3.2.3 ARGUMENTS 111

3.3.3 dbvor createdb 113

3.3.3.1 NAME 113

3.3.3.2 SYNOPSIS 113

3.3.3.3 ARGUMENTS 113

3.3.3.4 USAGE 114

3.3.4 dbvor create_experiment 115

3.3.4.1 NAME 115

3.3.4.2 SYNOPSIS 115

3.3.4.3 ARGUMENTS 115

3.3.5 dbvor delete_experiment 116

3.3.5.1 NAME 116

3.3.5.2 SYNOPSIS 116

3.3.5.3 ARGUMENTS 116

3.3.6 dbvor gmimarker 117

3.3.6.1 NAME 117
3.3.6.2 SYNOPSIS 117
3.3.6.3 ARGUMENTS 117
3.3.7 dbvor gmimarkeralias 118
3.3.7.1 NAME 118
3.3.7.2 SYNOPSIS 118
3.3.7.3 ARGUMENTS 118
3.3.8 dbvor markeralias 119
3.3.8.1 NAME 119
3.3.8.2 SYNOPSIS 119
3.3.8.3 ARGUMENTS 119
3.3.9 dbvor markerinfo 120
3.3.9.1 NAME 120
3.3.9.2 SYNOPSIS 120
3.3.9.3 ARGUMENTS 120
3.3.10 dbvor missingmarkerinfo 122
3.3.10.1 NAME 122
3.3.10.2 SYNOPSIS 122
3.3.10.3 ARGUMENTS 122
3.3.11 dbvor member 123
3.3.11.1 NAME 123
3.3.11.2 SYNOPSIS 123
3.3.11.3 ARGUMENTS 123
3.3.11.4 USAGE 124
3.3.12 dbvor memberalias 125
3.3.12.1 NAME 125
3.3.12.2 SYNOPSIS 125
3.3.12.3 ARGUMENTS 125

3.3.13 dbvor newmarker 126
3.3.13.1 NAME 126
3.3.13.2 SYNOPSIS 126
3.3.13.3 ARGUMENTS 126
3.3.14 dbvor plink 127
3.3.14.1 NAME 127
3.3.14.2 SYNOPSIS 127
3.3.14.3 ARGUMENTS 127
3.3.14.4 ADDITIONAL TRAIT ARGUMENTS 128
3.3.15 dbvor plinksample 129
3.3.15.1 NAME 129
3.3.15.2 SYNOPSIS 129
3.3.15.3 ARGUMENTS 129
3.3.16 dbvor sample 130
3.3.16.1 NAME 130
3.3.16.2 SYNOPSIS 130
3.3.16.3 ARGUMENTS 130
3.3.16.3.1 sample file format 130
3.3.17 dbvor set_experiment_date 132
3.3.17.1 NAME 132
3.3.17.2 SYNOPSIS 132
3.3.17.3 ARGUMENTS 132
3.3.18 dbvor trait 133
3.3.18.1 NAME 133
3.3.18.2 SYNOPSIS 133
3.3.18.3 ARGUMENTS 133
3.3.19 dbvor DeleteBlockAllSamples 135
3.3.19.1 NAME 135

3.3.19.2 SYNOPSIS 135
3.3.19.3 ARGUMENTS 135
3.3.20 dbvor DeleteBlockMember 136
3.3.20.1 NAME 136
3.3.20.2 SYNOPSIS 136
3.3.20.3 ARGUMENTS 136
3.3.21 dbvor DeleteBlockSample 137
3.3.21.1 NAME 137
3.3.21.2 SYNOPSIS 137
3.3.21.3 ARGUMENTS 137
3.3.22 dbvor DeleteSnpBlockMap 138
3.3.22.1 NAME 138
3.3.22.2 SYNOPSIS 138
3.3.22.3 ARGUMENTS 138
3.3.23 dbvor ShowSamples 139
3.3.23.1 NAME 139
3.3.23.2 SYNOPSIS 139
3.3.23.3 ARGUMENTS 139
3.4 Genotype Entry 140
3.4.1 Geno 140
3.4.1.1 NAME 140
3.4.1.2 SYNOPSIS 140
3.4.1.3 ARGUMENTS 140
3.4.1.4 Deprecated Arguments 144
3.4.2 Genoi 145
3.4.2.1 NAME 145
3.4.2.2 SYNOPSIS 145
3.4.2.3 ARGUMENTS 145

3.4.2.3.1 make-snps marker data 145
3.4.2.3.2 genotype data 147
3.4.3 Genok 150
3.4.3.1 NAME 150
3.4.3.2 SYNOPSIS 150
3.4.3.3 ARGUMENTS 150
3.4.3.3.1 make-snps marker data 150
3.4.3.3.2 genotype data 151
3.4.4 Genoa 152
3.4.4.1 NAME 152
3.4.4.2 SYNOPSIS 152
3.4.4.3 ARGUMENTS 152
3.4.4.3.1 make-snps marker data 153
3.4.4.3.2 genotype data 155
3.5 Data extraction using genout 158
3.5.1 NAME 158
3.5.2 SYNOPSIS 158
3.5.3 ARGUMENTS 158
3.5.3.1 Formatting Arguments 158
3.5.3.2 Type values 160
3.5.3.3 Heterozygote values 161
3.5.3.4 Filters 161
3.5.3.4.1 Select by marker, chromosome, etc. 161
3.5.3.4.2 Select by member 162
3.5.3.4.3 Select by trait 164
3.5.3.4.4 Select by technology and experiment 164
3.5.3.4.5 Filtering Experiment Data 165
3.6 Miscellaneous Commands 167

3.6.1 Genote 168
3.6.1.1 NAME 168
3.6.1.2 SYNOPSIS 168
3.6.1.3 ARGUMENTS 168
3.6.1.3.1 Insert mode 168
3.6.1.3.2 Automatic insertion mode 169
3.6.1.3.3 Fetch mode 170
3.6.1.3.4 Administrative 171
3.7 License agreement 172
3.7.1 GNU General Public License Version 3 for dbVOR 172

4 Index 188

A MySQL mini-tutorial 191

Chapter 1

Introduction

1.1 Organization of this Document

After this Introduction, this document is divided into two complementary parts: a Tutorial in Chapter 2, and a Manual in Chapter 3. We recommend reading the dbVOR Tutorial first to better understand its use and to see a typical work flow. Section 1.4 explains how to install the dbVOR package. Appendix A presents some of the operational details using a MySQL database.

The web page `https://watson.hgen.pitt.edu/register/docs/dbvor.html` *contains links to this document in PDF as well as in HTML format and it also contains links to other dbVOR specific materials.*

1.2 Motivation

Genetic studies of complex human diseases typically involve multiple iterations of genetic marker generation. For example, a set of individuals may first be assayed with a genome-wide chip that measures genotypes at hundreds of thousands of markers. Then a subset of interesting signals may be followed up by custom genotyping using a different technology. If there are technical problems, yet another round of custom genotyping using a third technology might be carried out on a subset of the samples. There can be overlap of both people and markers from different genotyping experiments; the overlap can introduce several kinds of problems. Most times the overlapping genotypes are the same, but sometimes they are different. Occasionally, the lab will return genotypes using a different allele labeling scheme

(for example 1/2 vs A/C). Sometimes, the genotype for a person/marker index is unreliable or missing. Further, over time some markers are merged and bad samples are re-run under a different sample name.

Managing and reconciling these multiple sources of data, often with different sample IDs, allele labels, and marker names, is best managed using a database system. We need a consistent picture of the subset of data we have chosen to work with even though there might possibly be conflicting measurements from multiple data sources. The dbVOR system was initially developed to import pedigree, phenotype and genotype data into a database. It is designed to be robust and handle the varieties of problems that occur with typical lab.

Once the data are in the database, they can be extracted using a number of selection criterion:

- chosen pedigrees
- chosen pedigree/persons
- chosen markers
- chosen chromosomes
- chosen ranges on chromosomes
- chosen experiments
- chosen technologies

and formatted to produced output in several styles:

- simple tabular listings
- Mega2
- PLINK: lgen, ped, bed

The current implementation of dbVOR is coded in the Python language version 2.x. The data are stored in a MySQL database. dbVOR was developed on a Mac computer running OS X 10.6 (aka Snow Leopard). Since Python and MySQL are supported on many platforms, it is expected that dbVOR will work on any platform where these two environments are available.

1.3 Prerequisites

We will assume that you have downloaded and installed:

Python	Python is one of several popular interpreted object oriented languages. See `http://www.python.org/download/` You must use Python version 2.6 or 2.7. We have not transitioned all of dbVOR to the Python 3.x series yet.
MySQL	MySQL is a popular open source database package. See `http://www.mysql.com/downloads/mysql`. We assume that you have created a database and also established a root password for the database.
MySQLdb	This Python package allows access to the MySQL database from Python. The simplest way to install this package is to use the Python "pip" installer, viz.<br>`pip install MySQL-python`<br>If necessary, you can load the installer from `https://pip.pypa.io/en/latest/installing.html`.<br>For more information about the MySQLdb software, see `http://sourceforge.net/projects/mysql-python/`, click download and read the README in the "doc" directory.
GMI	This program fetches position and chromosome information for SNPs. See `https://watson.hgen.pitt.edu/register/`. Click "New Users" or "Registered Users" then request to download GMI.

1.4 Download and Install dbVOR

dbVOR can be obtained, after registration, from `https://watson.hgen.pitt.edu/register/` as a .tar.gz file. A more recent but potentially less stable version of dbVOR can be cloned from `https://bitbucket.org/dweeks/dbvor`. If anonymity is desired, note that dbVOR can be obtained from the bitbucket site without any registration.

1.4.1 Install prerequisites

First install all of the prerequisites as listed in above Section 1.3.

1.4.2 Download and decompress dbVOR

Go to `https://watson.hgen.pitt.edu/register/` and download the **dbvor.1.11.tgz** archive file. After downloading the file, you should extract this file into an empty directory:

tar xvzf dbvor.1.11.tgz

This will create a dbVOR source tree in the current directory in a folder named **dbvor.1.11**.

Several directories will be created. The **bin** directory contains the dbVOR Python programs. The **doc** directory contains useful information. **doc/Book** contains this document in **pdf** and **html**. And **doc/Book/data** contains all the data files refereed to in the tutorial below. The **sql** directory contains specialized database code for defining the tables in the database.

1.4.3 Installation of dbVOR as a Python application

To install dbVOR as a Python module with binaries into the customary locations, you will likely need root access, as 'regular' users may not have adequate permissions to complete the installation. If you have root access, dbVOR can be installed by changing to the "*dbvor.1.11*" directory and typing:

sudo python setup.py install

This will perform a normal egg/wheel installation of dbVOR as a Python module and install the binaries into a standard place (such as /usr/local/bin).

1.4.4 Note and record the path to the 'sql' folder for later use

You will need to make a note of the path to the **sql** directory in the **dbvor.1.11** folder for later use. We will refer to it as SQLPATH and use it in section 2.3 of the tutorial. To do this, cd into the **sql** directory, type 'pwd' at the Unix prompt, and record the output.

1.4.5 Create a dbVOR database

Now you will need to create a dbVOR database, initializing the tables within which your data will be stored. To do this, use the **dbvor createdb** command (Section 3.3.3) by following the instructions in Section 2.3 of the Tutorial chapter 2.

1.4.6 Add information into the database

For detailed instructions regarding how to add information into the database, please see Chapter 2. Tables 2.1 and 2.2 give an overview of some of the programs that are used to populate the database tables. Note that there are two ways of storing data in dbVOR: the first is best for storing targeted small-scale data, while the second is best for storing genome-wide scale data. The Tutorial in Chapter 2 initially focuses on how to enter small-scale data, while Section 2.7 describes how to enter and store genome-wide data.

1.5 Citing dbVOR

If you use dbVOR as part of a published work, please remember to reference dbVOR. You may reference it by citing the following:

> Baron RV, Conley YC, Gorin MB, Weeks DE (2015)
> dbVOR: A database system for importing pedigree, phenotype and genotype data and exporting selected subsets.
> Submitted.

as well as citing the web site and the version that you used. For example, the web site citation for version 1.11 should be:

> Baron RV, Conley YC, Gorin MB, Weeks DE (2015) dbVOR (Version 1.11).
> `https://watson.hgen.pitt.edu`

1.6 Support, bug reports, and feedback

1.6.1 dbVOR Google Group

dbVOR users are invited to participate in our dbVOR Google Group at `https://groups.google.com/d/forum/dbvor`. Support questions should be posted there.

1.6.2 Bug reports

If you encounter a bug in dbVOR, please send us a detailed bug report, including the following information:

- Program Name: dbVOR
- Program Version: [dbVOR prints out a version number when you first start it up.]
- Platform: [Windows, Macintosh, Linux, etc.]
- Description:
- Example files: [If you can, please send us example files that will allow us to try to re-create the bug on our own computers.]

Please send your bug reports and feedback to: Daniel E. Weeks at weeks@pitt.edu

1.7 Contact information


```
Daniel E. Weeks, Ph.D.
Professor of Human Genetics and Biostatistics
Department of Human Genetics
Graduate School of Public Health
University of Pittsburgh
Crabtree Hall, Room A303
130 DeSoto Street
Pittsburgh, PA 15261
USA

1 412 624-5388
FAX: 1 412 624-3020
E-mail: weeks@pitt.edu
Web page: https://watson.hgen.pitt.edu
```


1.8 Acknowledgments

This work was supported by the National Institutes of Health grants R01 GM076667 (P.I. Daniel E. Weeks), R01 EY009859 (P.I. Michael B. Gorin), and American Recovery and Reinvestment Act supplement R01 EY009859-14S1 (P.I. Michael B. Gorin), and the University of Pittsburgh.

Chapter 2

Tutorial

2.1 Introduction

This chapter is a tutorial. The tutorial works through a small toy example to illustrate the features of dbVOR. Some of the features of most of the dbVOR commands are shown. The next chapter 3 explains all the options and all the commands in excruciating detail.

There are two ways to store genotype data within the database. The first stores one genotype per record, and is appropriate for small-scale data sets such as those obtained when typing a modest number of markers in a targeted follow-up experiment. The second stores genotypes in blocks, and so is appropriate for genome-wide data sets with large numbers of markers. The tutorial first works through a small-scale data set before describing how to enter in genome-wide scale data in Section 2.7. Note that even if you only have a genome-wide data set, to fully understand how **dbVOR** works, it still would be helpful to take the time to work through the tutorial.

2.2 Overview of dbvor commands

Tables 2.1 and 2.2 give an overview of some of the programs that are used to populate the database tables. The following tutorial will provide a detailed guide tour of how to use many of these commands.

Table 2.1: Some of the programs that populate the database tables.

**Program Name**	**Table**	**Behavior**
**dbvor createdb**	all tables defined	create a database, create a user and load schema definitions
**dbvor newmarker**	*markers*	define new markers
**dbvor missingmarkerinfo**	*markers, marker_info*	list markers without map info for the named build
**dbvor allelemap**	*allele_map*	store genotype remapping for a marker/experiment
**dbvor marker_info**	*marker_info*	store genetic and physical position data for a map build
**dbvor markeralias**	*marker_aliases*	store alternate name for a marker
**dbvor gmimarker**	*marker_info*	store marker map information presented in Genetic Map Interpolator (GMI) format
**dbvor gmimarkeralias**	*marker_aliases*	store marker aliasing data presented in GMI format
**dbvor sample**	*samples*	store sample data and associate it with a member
**dbvor trait**	*traits, traitmeta*	store trait values for members
**dbvor member**	*members*	store pedigree information
**dbvor plink**	*members, samples, traits and traitmeta*	store pedigree and sample information and optionally, trait and traitmeta data
**dbvor memberalias**	*member_aliases*	store alternate name for a member
**dbvor create_experiment**	*experiments*	create entry in *experiment* table associating ID with experiment string
**dbvor set_experiment_date**	*experiments*	set date field of specified experiment in the *experiment* table
**dbvor delete_experiment**	*experiments*	delete all genotype data for the specified experiment
**dbvor set_active**	*genotypes*	set the active flag, to turn on/off specific data
**dbvor DeleteBlockAllSamples**	*genotypeblocks, samples*	delete all gwas data for the specified experiment
**dbvor DeleteBlockMember**	*genotypeblocks, samples*	delete all gwas data for the specified person in all experiments
**dbvor DeleteBlockSample**	*genotypeblocks, samples*	delete all gwas data for the specified samples in the given experiment
**dbvor DeleteSnpBlockMap**	*snpblocks*	delete all gwas marker mapping data for the specified

Table 2.2: Programs that add genotype data to the database.

Program Name	Table	Behavior
**geno**	***genotypes***	populate ***Genotypes*** table
**genoi**	***genotypeblocks***, ***snpblocks***, ***marker_info***	populate ***Genotypeblocks***, ***snpblocks***, and ***marker_info*** tables from Illumina data
**genoa**	***genotypeblocks***, ***snpblocks***, ***marker_info***	populate ***Genotypeblocks***, ***snpblocks***, and ***marker_info*** tables from Affymetrix data
**genok**	***genotypeblocks***, ***snpblocks***, ***marker_info***	populate ***Genotypeblocks***, ***snpblocks***, and ***marker_info*** tables from PLINK binary data

2.3 Creating a database

We must start by creating a MySQL database[1] with several tables and by creating a MySQL user who can access that database. Note that a MySQL user is defined in the MySQL database environment, and can differ from your Unix user name. We will assume that:

the database server machine is	**localhost**
the database name is	**exampledb**
your MySQL user name is	**example**
the "root" name for the server database is	**root**
the SQL table definitions are in the specified directory	**SQLPATH**

dbVOR saves you from the nitty gritty details of typing MySQL commands to create this database. (Section 1.4.4 explains how to define SQLPATH. It is simply the path to the SQL code that was included in the dbVOR installation. You only need to know it this once for **dbvor createdb**.)

You simply type[2]:

> **dbvor createdb --db=***exampledb* **--host=***localhost* **--user=***example* **--root=***root* **--sqlpath=<SQLPATH>**

[1]Technically, the database has already been created and we are creating what MySQL calls a data schema. But it is more common to call the schemas databases so we will stick with that convention.

[2]I will use the verb "run" or "type" (as I have here) to indicate that all the text on the subsequent indented line(s) should be typed on the computer command line. All the text should be typed on a single line even though there might be several indented lines. When you type them, these indented lines should be separated by at least one space.

where <SQLPATH> is replaced by the complete path to the directory containing the SQL table definitions (See Section 1.4.4 for details). This is the path to the **sql** directory of the **dbvor.100** folder.

(You can create as many additional databases[3] as you need; one is adequate for this exercise.)

You will have to provide most of this information: the database, the server, the user (and password), to each dbVOR program that you run so it can find this database. We have a simple mechanism to avoid having to type these details to every program.

2.3.1 Using the .dbvorrc configuration file

The **dbvor** program is used to create and populate most tables in the database (all except for the genotype table). The program is always followed immediately by the name of a sub-command: in this case **createdb**[4]. All the sub-commands used here (and in other dbVOR programs) are supplied with arguments. Typically, arguments are introduced with two "-" characters followed by the argument name. The value of the argument, if necessary, comes next, either introduced as part of the same text separated with an "=" or as the next (separate) argument[5].[6]

All commands need arguments, for example the database parameters we discussed earlier, and some commands need quite a few more arguments. It would be tedious and error prone to type the arguments on each command line. Thus, the dbVOR programs allow you to specify the arguments in a configuration file, in addition to on the command line. You would typically specify the configuration file name on the command line and possibly supply some additional arguments. These additional command line arguments can supplement or override the configuration file data.

Some information (the database parameters) is needed and hence repeated for every command. To eliminate the need for cut and paste repetition in each configuration file, **dbVOR** searches for a specially named configuration file in your *home* directory, ***.dbvorrc***[7]. This file is processed before any other arguments are seen; so is a good place to store common/con-

[3]schemas

[4]The subcommand name is not case sensitive; **createDB** will work just as well.

[5]Thus, the following command is equivalent to what was just shown: **dbvor createdb --db** ***exampledb*** **--host** ***localhost*** **--user** ***example*** **--root** ***root*** **--sqlpath SQLPATH**

[6]Note: Some arguments do not need values and some arguments only have a single "-".

[7]On Unix and Mac OS X, there is a long standing tradition that files that begin with "." are considered hidden. The normal file listing utility, **ls**, does not show these files by default. You must add the **-a** flag to **ls** to get information about hidden files.

stant arguments. ***.dbvorrc*** can contain the value for any argument, but if the argument is encountered later in a configuration file or on the command line, the later value is used.

I typically only include the **host, user,** and **password** in ***.dbvorrc***. I supply the database and some related parameters in each configuration file. This is mainly because I work with several different databases and experiments at a time. NOTE: The permissions for the ***.dbvor*** file should allow only the owner to read the file since it contains a password.

To simplify the use of this tutorial, **dbvor createdb** creates a ***.dbvorrc,*** but only if it does not exist. It will contain the **host**, **user**, and **password** as expected but in addition a few more arguments that make sense if you only are working with one database: **db**, **dir**, and **project. db** is the database (illustrated previously). **dir** is the directory that contains the information to be loaded into the database; by default, the current working directory is used. Finally, **project** is the project name; for now this should be the literal value, ***project***.[8] If you define another database, you will most certainly change these latter parameters. And, most likely, you will just add the parameters to any new configuration file that you create rather than changing the ***.dbvorrc*** back and forth.

Note: The .***dbvorrc*** file is created with permissions that make it readable only by the file's owner so the password is NOT exposed to everyone. The owner is the person who is running the **dbvor createdb** program.

With **dbvor createdb** invoke as above and using "secret" as the password, **.dbvorrc** looks like:

```
#The 3 lines below are included to make it easy to transfer other .cfg files and data to
#other users
host:       localhost
user:       example
password:   secret

#The lines below are added for convenience;  They should be really specified in
#individual .cfg files , not here
db:         exampledb
dir:        .
project:    project
```

2.4 Populating the database

All the example data and configuration files that are discussed in this tutorial are supplied in the ***data*** directory that is provided with this tutorial. You should run the change directory

[8] The implementation of **project** is currently being revised.

command, **cd**, viz.

cd doc/Book/data

to switch to that directory before proceeding with these exercises.

We will work on a very simple example to introduce the features of dbVOR. There are several different files. The data are totally fabricated and bear no relation to any existing study data. The first file we will work with, *dataset1.txt*, is shown below:

```
pedigree    person  father  mother  sex trait   rs7418435-1 rs7418435-2
1           1       0       0       1   0       0           0
1           2       0       0       2   2       A           G
1           3       1       2       1   2       A           A
1           4       1       2       2   1       G           A
2           101     0       0       1   0       A           G
2           201     0       0       2   2       G           A
2           301     101     201     1   2       A           A
2           401     101     201     2   1       G           A
2           501     101     201     2   2       G           G
```

The file has 8 columns that are separated by white space; you can indicate an explicit separator if required. The header line on this and subsequent files is required. The first 5 columns are family data, followed by one column for a trait value and then the two columns for the two alleles of SNP rs7418435. For the latter two columns, we make a header by appending "-1" and then "-2" to the SNP name, rs7418435, viz. rs7418435-1 and rs7418435-2. Rather than have one file with these data, dbVOR can also accept the pedigree in one file, the traits in another and the markers in still others.

The pedigree diagrams for the above two families are:

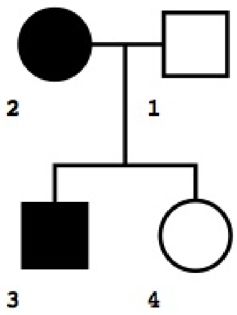

pedigree 1

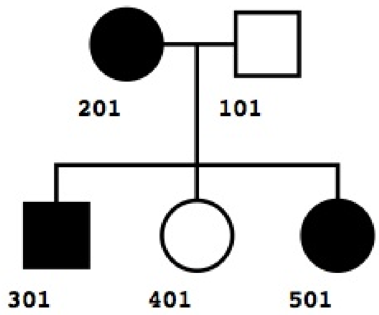

pedigree 2

2.4.1 Adding Pedigree Data using dbvor member

The data from the *dataset1.txt* file will populate several tables in the database. The first table contains pedigree data. The command, **dbvor member**, will load the pedigree data. First move into the **doc/Book/data** directory. Then let us type on the command line:

dbvor member ***example.ds1.ped.cfg***

example.ds1.ped.cfg is an example of a configuration file that we discussed earlier[9]. For **dbvor member**, we need to specify the pedigree information:

**--input**	the input file, *dataset1.txt*
**--pedigree**	the column number of the pedigree field
**--person**	the column number of the person field
**--father**	the column number of the father field
**--mother**	the column number of the mother field
**--gender**	the column number of the gender field

By convention, we label the first column of the input file: column 1 and subsequent columns are assigned consecutive integer values. There are many more arguments available.[10] [11]. Further, there are several arguments that are needed for almost every dbVOR command.

**--dir**	directory of **--input** file and also used to store logs, etc
**--project**	project name
**--db**	data schema to hold MySQL database tables
**--host**	MySQL database server machine
**--user**	user name for database access
**--password**	password for database access for above user

Most of these arguments were needed for **dbvor createdb** and all of these arguments were written into the ***.dbvorrc*** files. So it is unnecessary to repeat them in the configuration file shown below.

The ***example.ds1.ped.cfg***[12] file will look like:

```
input:     dataset1.txt
pedigree:  1
person:    2
```

[9]dbVOR assumes that the first unmarked (no **--**) argument is a configuration file. It could be preceded with a **--config** argument but that is unnecessary.

[10]If you want to see all the arguments, type **dbvor member --help**. (You can add **--help** to any command.)

[11]By default, the inter-column separator is white space. If the data have blank fields, a white space separator will confuse matters. The argument **--sep** lets you specify an explicit separator such as tab or comma.

[12]Note: In a configuration file, the arguments are typed without the initial "--", but with a trailing ":".

```
    father:    3
    mother:    4
    gender:    5
```

When you run **dbvor member *example.ds1.ped.cfg***, you see (of course the dates and times will be different for you)

```
Config: parsed ~/.dbvorrc for config options
START 08/15/2011 16:04:37, dbVOR version 0.55

cmd: /Users/me/bin/dbvor example.ds1.ped.cfg
cwd: /Users/me/dbVOR/doc/Book/data

              config: 'example.ds1.ped.cfg'
                host: 'localhost'
                user: 'example'
                  db: 'exampledb'
                 dir: './'
             project: 'project'
               input: 'dataset1.txt'
            pedigree: 1
              person: 2
              father: 3
              mother: 4
              gender: 5

SUCCESS: dbVOR version 0.55

END 08/15/2011 16:04:37, DURATION 0.057 sec
```

but what happened. The listing shows you the start time, end time and duration of the run. It echoes the command typed and the directory the command is running in. It summarizes the arguments that are present in the configuration file (merged with the ~/.dbvorrc configuration file). A ***log*** file contains all the information that is printed out between the START and END and can contain additional details. All this information is always generated during dbVOR program execution. The listing does not show arguments that are not changed from their default value. The default ***log*** file name is *<config file prefix>_ <date>.log.txt*, in this case *example.ds1.ped_20110815_16_04_37.log.txt*.

The pronouncement SUCCESS: towards the end of the printout indicates that there were no errors during processing (as opposed to a FAILURE: notice.) But what really happened? With no other "special" arguments specified, a dbVOR program just verifies that both the configuration file and also that the input file(s) are well formed. If you type on the command line:

> **dbvor member** ***example.ds1.ped.cfg --verbose***

you will see a printout of the parsed file data. The first time you try any of the commands presented below, you might want to add the **--verbose** keyword to see additional details. (You can also add the flag argument, ***--debug***, to see extra debugging details. This information tends to be dbVOR internals oriented and is more suitable for a developer rather than a user of the dbVOR programs.)

We observe the following protocol for inserting data into the database. First, we type the simple command (as above) to make sure the data are parsable. Then we type the command with the additional argument **--compare** viz.:

> **dbvor member** ***example.ds1.ped.cfg --compare***

to check whether the data are already in the database. This is obviously not necessary here, but in the general case, we might have entered some data and do not remember that we did. Someone else may have entered the data or some of the data. We may be curious whether the data file, in this case *dataset1.txt*, is actually what was used to populate the database. Finally, we might have changed some fields (parentage or sex) for some people in the file, but are not sure if the changes made their way into the database.

When we are happy that the data will go into the database correctly (and have fixed any problems along the way), we type the command with the argument **--commit** viz.:

> **dbvor member** ***example.ds1.ped.cfg --commit***

You might want to rerun

> **dbvor member** ***example.ds1.ped.cfg --compare -v***

to verify that the data are in the database and to see what the print out looks like when data are already present. Note: To make typing easier, the single letter argument **-v**, can be used in place of **--verbose (** and **-d** in place of **--debug**). A comparison entry looks like:

```
Line          2: pedigree:person 1:1
1)  "1:1" has the same value as database:
     father:'0', mother:'0', gender:1
```

The first line of the entry shows the line number from the input file and the person:pedigree value. The second line repeats the person:pedigree value and indicates whether the entry matches or does not match the database entry. It then shows a (or both for non-match) records in database format. The fields are: father, mother, and gender

Suppose we had changed the gender of pedigree 2 person 501 to 1 in *dataset1.txt* after running the initial **--commit**. A compare would now show this entry:

```
Line         10: pedigree:person 2:501
1)  "2:501" has a different value than database:
  new    father:'101', mother:'201', gender:1 vs
   db    father:'101', mother:'201', gender:2
```

For the **dbvor** sub-commands, ***--commit*** will only enter into the database records that are not already there. If you rerun

dbvor member ***example.ds1.ped.cfg --commit***

After you make the above gender change in *dataset1.txt* you will see the warning:

```
Line         10: pedigree:person 2:501
1)  IGNORING NEW VALUE
1)    new value = project id:1, father:'101', mother:'201', gender:1, active:1
  database value = project id:1, father:'101', mother:'201', gender:2, active
```

To force the database to be updated you must supply the ***--replace*** argument, viz. run:

dbvor member ***example.ds1.ped.cfg --commit --replace***

The convention of requiring ***--replace*** to replace record values applies to all sub-commands of **dbvor**[13]. Of course, it is also possible to type:

dbvor member ***example.ds1.ped.cfg --commit --delete***

to remove the entry entirely and then start again. (The actions: ***-compare***, ***-commit***, ***-delete***, and ***--replace*** are accepted by all of the sub-commands of the **dbvor** program.)

[13]NOTE: In some occasional cases where database constraints get in the way, you have to use the argument ***--update*** instead of ***--replace***. The individual sub-command will indicate when this is necessary.

2.4.2 Adding Trait Data using dbvor trait

The trait data come from the same file as the pedigree data, *dataset1.txt*. The command to enter the data is:

dbvor trait ***example.ds1.trait.cfg***

The necessary trait configuration data are:

**--input**	the input file, *dataset1.txt*
**--pedigree**	the column number of the pedigree field
**--person**	the column number of the person field
**--columns**	the column number of a trait field
**--types**	the type of the corresponding column

(**--columns** and **--types** illustrate a new kind of argument, that can be repeated as many times as necessary. Consider a file that lists several traits per person.) Since the trait data in *dataset1.txt* appear to be an affective trait, we use type **A**. (**dbVOR** will validate that the values are 0, 1 or 2.) (Other possibilities for --**types** include: **T** for number.) The name of the trait is taken from the header of the corresponding trait column in the **--input** file. If that is not descriptive enough, we can use the **--titles** argument (repeatable like **--columns**) to tailor the name.

The ***example.ds1.trait.cfg*** file looks like:

```
input:     dataset1.txt
pedigree:  1
person:    2
columns:   6
types:     A
```

Note: If there were several **--columns** of traits (say 6, 7, and 8), all of the configuration file lines below are equivalent. (This is always the case for any argument that accepts multiple values.)

```
columns:   6, 7, 8
         or
```

```
columns:  6
columns:  7
columns:  8
          or
columns:  6, 7
columns:  8
```

With this configuration file, we run the command variants:

dbvor trait ***example.ds1.trait.cfg***

dbvor trait ***example.ds1.trait.cfg --compare***

dbvor trait ***example.ds1.trait.cfg --commit***

to ultimately enter the data into the trait table. We leave as an exercise to the reader to run the commands. Do notice a few things. First, **--columns** and **--types** values are printed in the **log** output enclosed in brackets, to indicate that these arguments have a list of values.

```
columns: [6]
  types: ['A']
```

Also at the end of the log, we see:

```
===== Starting the "trait1" subsection
trait "trait1" type A col 6 (db id 4)
           value    count
             '0'        2
             '1'        2
             '2'        5
trait1: min 0.0, max 2.0, avg 1.33333333333, count 9, NA's 0, ""'s 0
```

We compute summary information as each dbVOR program runs and later print the results. Here, we show the three legal values for “trait1” and the count of each occurrence.

You could imagine for other type traits with many samples, the range of possible values could get quite large; you would not want to see all the values scrolling down the screen. Each potentially large run of data output is called a *subsection*; it is introduced in the output with “=====” and has a name. If the number of entries (not necessarily lines) printed in the subsection exceeds 10, output to the screen will be stopped and a summary line eventually printed indicating the true number of entries generated. *NOTE: All the entries will be available in the* ***log*** *file.*

2.4.3 Adding targeted marker data using geno

We call the SNP data associated with some markers for some people an *experiment*. A study will have one or more experiments. As data is generated, it should be captured in a file and the file can then be loaded into the database. The allele measurements are made using a *technology* such as TaqMan, iPLEX, etc. We record the technology for later data selection.

In our example, the first round of SNP data are also in the dataset1.txt file. The command to process marker data is new, **geno**. We are going to use the command:

geno *example.ds1.snp.cfg*

We need to set:

**--pedigree**	the column number of the pedigree field
**--person**	the column number of the person field
**--marker**	the column number of the first marker field
**--type**	description of how the markers are arranged in the file
**--technology**	name of the technology used
**--experiment**	name of the experiment, viz. dataset1.txt

,

In an experiment file, each column starting with the **--marker** column specifies alleles. The *dataset1.txt* experiment file is in "wide" format (**--type**=**wide**). **Wide** format indicates that there is one allele per column and that the column headers contain the marker name followed by a "-1" or a "-2" depending whether this is the first or second allele for the marker. In this example, all columns from the marker column 7, viz. 7 and 8 specify alleles in **wide** format. If there were more columns, the next pair would be for the next marker, etc. (For future reference, (**--type**=half) indicates that there is one column labeled with the snp name that contains both alleles.)

The ***example.ds1.snp.cfg*** file looks like:

```
pedigree:    1
person:      2
marker:      7
type:        wide
technology:  exampletech1
experiment:  dataset1.txt
```

With this configuration file, we want to run the command

geno *example.ds1.snp.cfg*

to ultimately enter the data into the genotype table. But the command fails. Recall that at the end of any command print out, there is indication whether the command succeeded or failed.

geno *example.ds1.snp.cfg* shows:

FAILURE: 2 Errors, dbVOR version 0.55

The particulars of the error was reported as[14]: For now, we only care about the first 3 observations in the log and have replaced the last three entries with "...". We will discuss them later.

```
The following markers are not in the marker (or alias) table:
'rs7418435'

 Missing markers are stored in  ./example.ds1.snp_MissingMarkers.txt.


To accept them, type:
    dbvor newmarker --dir=. --project=project
      --input=./example.ds1.snp_MissingMarkers.txt
      --db=exampledb --commit


...
```

This error might just indicate a data entry typographical error that we will have to go back to the input, *dataset1.txt*, to fix. But in this case, it merely indicates that the particular marker, *rs7418435*, has not been encountered before. The text in the error message indicates how to tell dbVOR to accept a list of markers as valid. You should follow the suggestion and type on the command line:

dbvor newmarker --dir=. --project=project

--input=./example.ds1.snp_MissingMarkers.txt --db=exampledb --commit

[14]In this display (and later displays), we occasionally split single lines into several lines to make the presentation more readable.

The **--db=exampledb** argument as well as the **--dir** and **--project** might not be necessary because they can be supplied in the ***.dbvorrc*** file. Of course, the value may not be in the ***.dbvorrc*** or may have been overwritten. **dbvor** can not tell when it is generating these lines so it is safer to place the text here. (The **--host**, **--user**, and **--password** might also have been overwritten, in which case they also need to be on the command line (above). But this is much less likely, so the "hint" does not show them.)

After the above command is run dbVOR will be able to run any of the following commands as expected:

> **geno** ***example.ds1.snp.cfg***
>
> **geno** ***example.ds1.snp.cfg --compare***
>
> **geno** ***example.ds1.snp.cfg --commit***

For each marker, the log will contain a count for each allele seen:

```
Counts for each marker field
rs7418435            '0':     2,    'A':     9,    'G':     7,
```

You can imagine that not having chromosome and position information for the markers will limited the functionality of dbVOR. That will be addressed after the next section. But now let's see the results of our work so far.

2.4.4 Retrieving and exporting data using genout

The **genout** program retrieves and exports data from the database. A simple invocation would be:

> **genout** ***example.ds1.out.cfg***

The configuration file, ***example.ds1.out.cfg,*** looks like:

```
type:        half
output:      example.ds1.out
marker:      rs7418435
```

The explanation of the argument values is:

**--type**	indicates the output format desired
**--output**	indicates the stem of the output file
**--marker**	indicates the marker requested

I am sure you can guess: **wide** will produce a file that looks exactly (well almost) like *dataset1.txt* and **long** a file like **plink**[15] *lgen* format. You can also ask for **mega2**[16] format output and most of the **plink** formats including the binary ones[17]. A table listing all of the possible **type** values can be found in Section 3.5.3.2.

Some formats (viz. **mega2, plink**) produce several output files with **--output** as the stem. For a simple **--type** like *half,* the output file name is the value of **--output** argument with ***.txt*** appended. The file, *example.ds1.out.txt* is shown below:

#PEDIGR	PERSON	FATHER	MOTHER	SEX	trait1	rs7418435
1	1	0	0	1	NA	00
1	2	0	0	2	2	AG
1	3	1	2	1	2	AA
1	4	1	2	2	1	AG
2	101	0	0	1	NA	AG
2	201	0	0	2	2	AG
2	301	101	201	1	2	AA
2	401	101	201	2	1	AG
2	501	101	201	2	2	GG

There are a few things to note about the output. The **trait1** values of 0 are reported as NA and the heterozygous alleles are listed in alphabetical order. These are default behaviors and can be changed.

2.4.5 Adding Marker Position Data using GMI

Ultimately, we will want to extract data from the database by chromosome, or position range (absolute or genetic), rather than exclusively by marker names. For a given marker, you can imagine supplying:

[15] `http://pngu.mgh.harvard.edu/~purcell/plink/index.shtml`
[16] `https://watson/hgen.pitt.edu/docs/mega2_html/mega2.html`,
`https://watson.hgen.pitt.edu/docs/mega2_html/Mega2_Documentation.pdf`
[17] Type: **genout --help** and look at the legal values for **--type**.

**marker**	marker name
**build**	build from which position info is obtained
**chromosome**	chromosome number or identifier
**position**	position of marker on the chromosome
**average**	average genetic position of marker
**male**	genetic position of marker for males
**female**	genetic position of marker for females

If you have these data available, you can use the **dbvor markerinfo** program to load these data (or a subset) into the marker_info table in dbVOR. This program and a related one will be explained in the next section.

There is a simpler alternative than hunting down all this information. The **gmi.pl**[18] program will get the data for you. **gmi.pl** acquires this information from the latest Ensembl[19] build.

Recall when we originally ran:

geno *example.ds1.snp.cfg*

the complete output was:

```
The following markers are not in the marker (or alias) table:
'rs7418435'

Missing markers are stored in ./example.ds1.snp_MissingMarkers.txt.
To accept them, type:
    dbvor newmarker --dir=. --project=project
     --input=./example.ds1.snp_MissingMarkers.txt --db=exampledb --commit

To look up the marker positions, type:
            gmi.pl ./example.ds1.snp_MissingMarkers.txt
        --outfile ./example.ds1.snp_gmi_out

To put them in the database, type the TWO lines below (correct
the arguments if necessary):
            dbvor gmimarkerALIAS --dir=. --project=project
        --input=./example.ds1.snp_gmi_out_log.txt
```

[18] https://watson.hgen.pitt.edu/register/doc/gmi.html
[19] http://www.ensembl.org

```
--db=exampledb --commit

   dbvor gmimarker --dir=. --project=project
--input=./example.ds1.snp_gmi_out_annot.txt
--build=<marker_build_version> --db=exampledb --commit
```

The instructions supplied above list three commands, along with their required arguments. Note that these commands are stored in the log file, so you can copy them from there if needed later. The appropriate value for the <marker_build_version> build identifier may be determined by examining the header line of the ***example.ds1.snp_ gmi_ out_ annot.txt*** file (see below for details).

The first command runs GMI to look up the marker positions; it has already pre-determined the required arguments for **gmi.pl**. You should type:

gmi.pl ./example.ds1.snp_MissingMarkers.txt --outfile ./example.ds1.snp_gmi_out

gmi.pl solves two problems. First, *rs7418435* is no longer the current name for that SNP at position 60116260 on chromosome 1; in reference assembly build 36.3 dbSNP, it has been merged with *rs1073516*. When you run **dbvor gmimarkerALIAS**[20]

dbvor gmimarkerALIAS --dir=. --project=project

--input=./example.ds1.snp_gmi_out_log.txt

--db=exampledb --commit

it will read the **gmi.pl** log file, look for the merged marker section and add it to the database. (If you then rerun **genout**, you will see that the marker column heading changed. dbVOR will always report the most recent marker name in output, but any of the earlier marker names that merged to the same name can be used for selecting markers in input.)

Second, **dbvor gmimarker** is a variant of **dbvor marker_info** that knows how to read the **gmi.pl** output files. to gather genetic position information. So when you run:

dbvor gmimarker --dir=. --project=project

--input=*./example.ds1.snp_ gmi_ out_ annot.txt*

--build=ensembl63 --db=exampledb --commit

[20] I mentioned earlier that the case of the subcommand to **dbvor** did not matter.

it will populate the marker_info table in the database.

NOTE: Looking at the last line of the code hint, you are expected to replace **--build=<*marker_build_ve*** with a concrete build identifier, viz. **--build=ensembl63** or **--build=37.2** or **--build=hg19**, etc. The appropriate value for the build identifier may be determined by examining the header line of the ***example.ds1.snp_gmi_out_annot.txt*** file.

NOTE: From the discussion at the end of section 2.4.3, the arguments **--dir**, **--project**, and **--db** might not be necessary.

NOTE: Finally, if you did not download and install **gmi.pl**, the results of running:

> **gmi.pl ./example.ds1.snp_MissingMarkers.txt --outfile ./example.ds1.snp_gmi_out**

are included in the tutorial data files for your convenience. So you can run **dbvor gmimarkerALIAS** and **dbvor gmimarker**.

2.4.6 More GMI Marker Position Data

At the end of section 2.4.3, we ran the **dbvor newmarker** command that added the new marker names to the database, though it did not add any marker info data. If we run

> **geno *example.ds1.snp.cfg***

again, it will no longer give us a listed of missing markers nor the sample code for adding marker data to the database that we discussed in the previous section. Suppose we somehow lost this sample code. Or suppose that Ensembl has come out with a new build and we want to add this build's marker info data to the database.

The **dbvor missingmarkerinfo** command is designed to peek at the list of valid markers and the marker_info table and print out a list of markers that need additional description and the sample code to get it. The program requires a single argument (which is optional):

**--build**	the name of the build we are inquiring about

With no build argument, **dbvor missingmarkerinfo** will print out the entire list of valid markers into a file named, *MissingMarkerInfo.txt.* And it will print out the sample code for running **gmi.pl, dbvor gmimarker** and **dbvor gmimarkerALIAS** with the argument file.,*MissingMarkerInfo.txt.* (I am not going to show the code here. You pretty much can guess what it looks like from the previous section; just the **--input** argument changed.) So

this is the simple way to get a list of markers and code hints to collect the marker data for the next genome build.

If you supply a **--build** argument, **dbvor missingmarkerinfo** will find the list of markers for which there is no marker data in the marker_info table for the given build. It will place the list in the file *MissingMarkerInfo_ <build name>.txt* and proceed to generate all the code hints to get marker data for this build. For a previously unknown build name, this process will give pretty much the same results as that with no **--build** specified, except the file name is slightly different.

If the specified build was already entered into the marker_info table, the listing that is produced is the list of markers that are missing from the table for this build. There are lots of reasons that you might not have a complete set of marker data for the build, especially if you choose the technique of the next section and supply the data by hand. The *MissingMarkerInfo_ <build name>.txt* will show the markers that are missing any marker data for the specified build and the program will suggest code to fix it.

If the set of marker data for the build is complete, the **dbvor missingmarkerinfo** will print a message and not generate its customary output.

2.4.7 Manually adding Marker Position Data and Aliasing Data

We introduced the **dbvor markerinfo** earlier. It takes a configuration file with the arguments:

**--input**	file containing position information about the markers
**--marker**	column number of the marker name
**--chromosome**	column number of the chromosome name
**--average**	column number of the average genetic distance (cM)
**--male**	column number of the male genetic distance
**--female**	column number of female genetic distance
**--position**	column number of the base pair position on the chromosome

I do not have any sample **--input** to show you. Of course, you could just use the **gmi.pl** program output, but that seems wrong.

If you are supplying this data yourself, you will also have to determine which SNPS have been merged together. (Obviously **gmi.pl** has to make this determination too.) *rs1073516* is the merged name for the SNP we looked at before, *rs7418435*. If you are supplying your

data via **dbvor markerinfo**, you must also supply information to indicate which markers have been merged, preferably sooner than later. The program to do this is:

dbvor markeralias

The **markeralias** sub-command is a bit atypical. If there is no **--input** specified, then the arguments **--old** and **--new** are the marker names that are to be merged. Otherwise, they indicate the columns in the **--input** file that contains the pairs.

These arguments can be specified on the command line or in a configuration file.

**--input**	the input file, *dataset1.txt*
**--old**	the column number of the original marker
**--new**	the column number of the merged marker

We could just type:

dbvor markeralias --old=*rs7418435* **--new**=*rs1073516*

If we had not used **dbvor gmimarkerALIAS**.

2.5 Adding Additional Data

We will proceed by adding more data to our example: some more people, some more traits, and some more markers.

2.5.1 Adding more Pedigree Data

The pedigree3.txt file is a pretty simple file. You should expect that the previous configuration file, *example.ds1.ped.cfg*, is almost exactly what you need for these data. You just have to change the **--input** argument. I will let you make this change and run **dbvor member** yourself. The correct configuration file is supplied with the tutorial. This file uses M/F vs 1/2 for sex, but that is automatically handled by dbVOR.

```
pedigree person father mother sex
3        1      0      0      M
3        2      0      0      F
3        3      1      2      M
3        4      1      2      F
```

This new pedigree would be diagrammed as:

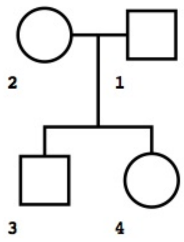

pedigree 3

When you run:

dbvor member ***example.ds2.ped.cfg*** **--compare -v**

you will see only information for pedigree 3 – nothing for pedigree 1 or 2. The comparison function uses the **--input** file as a key for what pedigrees to show. If you wanted to see all the pedigree information, you would have to append the first few columns of *dataset1.txt* and *pedigree3.txt*. If you then use this file as **--input**, you will see the full comparison.

2.5.2 Adding additional marker data using geno

The data in dataset2.txt introduce some more marker data for each person.

```
pedigree  person    rs72921657-1  rs72921657-2   rs1073516-1  rs1073516-2
1         1         0             0              0            0
1         2         C             A              0            0
1         3         A             A              0            0
1         4         A             C              0            0
2         2_101     0             0              G            A
2         2_201     C             C              A            G
2         2_301     C             A              A            A
2         2_401     C             A              A            G
2         2_501     C             C              G            G
```

3	1	A	A	G	A
3	2	C	C	G	G
3	3	A	C	A	G
3	4	0	0	A	A

The format is rather similar to *dataset1.txt* except we do not have pedigree or trait data in this file. (But the information is available in alternate files.) So *example.ds2.snp.cfg* is pretty similar to *example.ds1.snp.cfg* except that the **--marker** column is 3 and the **--input** file is now *dataset2.txt*. I have also assumed that the **--technology** for this set of experiments is different from the previous set.

2.5.3 Merged Markers / Marker Aliases

Now we try to add the additional marker data by running geno:

geno *example.ds2.snp.cfg*

Hopefully, we are informed that SNPS *rs72921657* "are not in the marker (or alias) table". At this point, we review sections 2.4.3 and 2.4.5 and then run the commands **"dbvor newmarker ..."**, **"gmi.pl ..."** and finally **"dbvor gmimarker ..."** hopefully by cutting and pasting the commands that were printed into the log.

BUT, we might be informed that SNPS *rs72921657* and *rs1073516* are unknown and must be registered with dbVOR. We stated earlier that *rs1073516* is the merged name for the SNP *rs7418435*. And we explained that, **dbvor gmimarkerALIAS** or **dbvor markeralias** could be used to register the association. It would appear that something failed and you should go back and check that step. Afterward, try re-running:

geno *example.ds2.snp.cfg*

2.5.4 Member Aliases

We are finally ready to ingest the markers from *dataset2.txt*, by typing:

geno *example.ds2.snp.cfg*

but we discover another (our last) problem.

```
 1 // geno example.ds2.snp.cfg
 2 // Config: parsed ~/.dbvorrc for config options
 3 // START 09/02/2011 15:53:57, dbVOR version 0.55
...
 4 // ===== Starting the "genotypewide" subsection
 5 // Line        6: pedigree:person 2:2_101
 6 // 1) ERROR: pedigree:person 2:2_101 is not in the MEMBER table.
 7 // Line        7: pedigree:person 2:2_201
 8 // 1) ERROR: pedigree:person 2:2_201 is not in the MEMBER table.
...
 9 // ===== Starting the "member_missing" subsection
10 // 5 pedigree:persons are not in the member table or alias table:
11 // Exclusion file was written to ./example.ds2.snp.MissingMembers.txt
12 // pedigree person
13 //        2   2_101
14 //        2   2_201
15 //        2   2_301
16 //        2   2_401
17 //        2   2_501
...
18 // FAILURE: 5 Errors, dbVOR version 0.55
19 // END 09/02/2011 15:53:57, DURATION 0.057 sec
```

We have prefixed a line number followed by " // " to the start of each line and have omitted some of the output which is indicated by ellipses marks in the printout. The problem is rather simple and summarized on lines 9 through 17. (Lines 5&6 show a single error report. The 1) at the start of line 6 indicates that this is the first error report for the above (line 5) line.) For whatever reason, the lab has reported different names for the people of pedigree 2. This can be a typographical error in their files. Or it could be a valid name for a different sample belonging to each person. The reason does not matter. We use the same type of mechanism that we just used for marker aliases. And a similar command:

dbvor memberalias

This stores a mapping of new name to the old name. (It might be worth noting that, internally, dbVOR treats these aliases slightly differently than *marker aliases.* In the case of *marker aliases*, the new name is considered the correct name, we just did not know it at the

time of data entry. In the case of *member aliases*, the new name is considered an alternate name for the correct name. In both cases, you can use the old name or the new name to refer same marker or same person.)

The **dbvor memberalias** command is invoked similarly to the **dbvor markeralias** command. Type:

dbvor memberalias *dataset2.alias.cfg*

The configuration file is similar to that for the marker configurations, but the old entries and new entries contain two items: both the pedigree and the person. Here is *dataset2.alias.cfg*:

**--input**	the input file, *dataset2.alias.txt*
**--oldpedigree**	the column number of the original pedigree name
**--oldperson**	the column number of the original person name
**--newpedigree**	the column number of the alias pedigree name
**--newperson**	the column number of the alias person name

The simplest input file, *dataset2.alias.txt*, would look like:

```
2    101    2    2_101
2    201    2    2_201
2    301    2    2_301
2    401    2    2_401
2    501    2    2_501
```

It was pretty easy to see how the person name was changed in the new data. Our lab will often concatenate a second sample number onto the name/number of the original sample for the same person, viz. 101/701[21]; sort of sample 701 for person 101. But clearly there is no standard way to guess the mapping of a new sample name but to go to the study protocol and see what was done.

Finally, run

dbvor memberalias *dataset2.alias.cfg* --commit

We could now run the **geno** command, but let's take care of one more detail first.

[21] We never did say that pedigree values or person values had to be integers; they do not. This allows a person to be 101/701 or E123 or whatever is convenient for your usage.

2.5.5 Another trait

We have one more trait to add in file *trait2.txt*, but we could not do this until we had accounted for the member aliasing:

```
pedigree    person    trait2
1           1         0
1           2         2
1           3         1
1           4         1
2           2_101     0
2           2_201     2
2           2_301     2
2           2_401     2
2           2_501     2
3           1         1
3           2         2
3           3         1
3           4         0
```

The configuration file is very similar to ***example.ds1.trait.cfg*** from section 2.4.2. We will leave it to you to create an appropriate configuration file and then run **dbvor trait** using it (See Section 2.4.2). You should get the summary statistics shown below:

```
===== Starting the "trait2" subsection
trait "trait2" type A col 3 (db id 3)
        value  count
          '2'      6
          '1'      4
          '0'      3
trait2: min 0.0, max 2.0, avg 1.23076923077, count 13, NA's 0, ""'s 0
```

2.5.6 Merging Experiments

2.5.6.1 Dry run

Now, please, once more, type:

geno ***example.ds2.snp.cfg***

Recall that invoked as above **geno** just reports on the data in the file to be processed. There are no errors reported. Let's look at the results. I have added line numbers down the left side of the printout for easier reference and have split an occasional long line in the middle of the line.

```
 1 \\ project: project, id 1
 2 \\ technology: exampletech2, id 0
 3 \\ experiment: "dataset2.txt", id 0
 4 \\
 5 \\ file "./dataset2.txt"
 6 \\
 7 \\ ===== Starting the "genotypewide" subsection
 8 \\
 9 \\ genotypes 26
10 \\
11 \\ 13 members included; 0 members excluded
12 \\
13 \\ ===== Starting the "includedpp" subsection
14 \\
15 \\ 13 pedigree:persons included:
16 \\ pedigree person
17 \\        1      1
18 \\        1      2
19 \\        1      3
20 \\        1      4
21 \\        2  2_101
22 \\        2  2_201
23 \\        2  2_301
24 \\        2  2_401
25 \\        2  2_501
26 \\        3      1
27 \\
28 \\ ===== The text above shows the first 10 records from a total of
          13 records in the log file for the "includedpp" subsection
29 \\
```

```
30 \\
31 \\ ===== Starting the "member_alias" subsection
32 \\
33 \\ 5 pedigree:persons were found in the member_alias table:
34 \\    alias   alias            original original
35 \\ pedigree person member_id pedigree person
36 \\        2   2_101         5        2    101
37 \\        2   2_201         6        2    201
38 \\        2   2_301         7        2    301
39 \\        2   2_401         8        2    401
40 \\        2   2_501         9        2    501
41 \\
42 \\ ===== Starting the "marker_contents" subsection
43 \\
44 \\ Counts for each marker field
45 \\ rs1073516          '0':    8,   'A':    9,   'G':    9,
46 \\ rs72921657         '0':    6,   'A':    9,   'C':   11,
47 \\
48 \\
49 \\ SUCCESS: dbVOR version 0.55
50 \\
51 \\ END 09/06/2011 11:36:00, DURATION 0.056 sec
```

I have not included the configuration information regurgitation; you have seen this before. Let me make a few observations about the log report:

**lines**	**observations**
2, 3	Because “dry run” does not use the database, an id of 0 is returned.
9	26 == 13 people times two markers per person
11, 15-26	There are 13 members that will be updated; they are listed. All members are updated and none are excluded
28	Subsection print out are limited to 10 entries on the screen. Look at the log file to see all the entries.
31-40	Show the aliased members and who they really are.
44-46	Show statistics for each marker. For each allele encountered, show the number of times it appeared

2.5.6.2 Verify Run

The above run of **geno** showed the data and the configuration file could be processed successfully. It does not say anything about what would happen when these data are added to the database. This may not be an issue for your particular study; perhaps there is only one measurement for the markers for each person. (But there are [almost] always controls that introduce redundancy/conflict.) A more helpful way to run the program is:

geno ***example.ds2.snp.cfg*** **--verify**

The **--verify** argument requests that the new data be "merged"[22] into the database data and any issues reported. All the listing details shown previously are also included in this next printout so we will not repeat them. The additional information is provided. The fact that the data (from *dataset1.txt*) were earlier entered into the database with old name for the marker is understood by dbVOR; thus processing will see multiple allele pairs present for some markers.

```
\\ ===== Starting the "marker_allconcordance" subsection
\\ Summary by marker:
\\ ===== "marker_allconcordance" record 1
\\
\\                                              missing or
\\ marker snp name      dup missing concordant concordant discordant
\\      1 rs1073516       9       1          5          3
0
\\      1 rs1073516  VS: exp 0 (cnt 9) & exp 1 (cnt 9): below 9;
\\     exp 0 == dataset2.txt,   exp 1 == dataset1.txt
\\                                               00   AA   AG   GG
\\                                          00    1    1    2    0
\\                                          AA    0    1    0    0
\\                                          AG    0    0    3    0
\\                                          GG    0    0    0    1
\\ diagonal        5, rest      0
\\ transpose       3, rest      2
\\
\\ Conflicts by members: end
```

[22] The merge is a gedanken experiment and only happens in the context of the program execution. No new data are inserted into the database without a **--commit**.

```
18 \\
19 \\ genotypes 26, present 9, match 6, zero 3, conflict 0, not present 17
20 \\
21 \\
22 \\ SUCCESS: dbVOR version 0.55
```

It is best to first look at line 19. It is a summary of the processing.

**lines**	**field**	**explanation**
19	genotypes	The total number of genotypes in the new input 13 people times two markers per person (26)
19	present	The number of markers that are already in the database. These were entered via *dataset1.txt* (9 people times 1 marker). Call this set the overlap.
19	match	The number of markers in the overlap that are identical in the database and new data.
19	zero	The number of markers in the overlap that were not measured in either the database or in the new data (but only measured in one). Thus there is one useful data value for the marker.
19	conflicts	The number of markers that have different allele pairs in the database versus the new data. The database after a **commit** will have both values. But by default, when data is pulled from the database with **genout**, the allele value of 0/0 will be supplied, indicating no measurement.
19	not present	The number of new entries in the input data file not already in the database.

The start of the listing shows the concordance of each marker and shows a concordance matrix for each pair of experiments in which the marker has been measured.

lines	field	explanation
5, 6	marker	Internal database id for marker
5, 6	snp name	marker name for the marker
5, 6	dup	number of persons for this marker that have more than one measurement
5, 6	missing	number of persons for this marker that have only zero (not measured) entries
5, 6	concordant	number of persons with the same allele pairs for this marker
5, 6	missing or concordant	number of persons with the same or zero (not measured) values for this marker
5, 6	discordant	number of persons that have conflicting measurements for this marker

A concordance matrix is generated for each pair of experiments. It has a two line heading illustrated below:

lines	field	explanation
7, 8	marker	Internal database id for marker
7, 8	snp name	marker name for the marker
7, 8	exp 0 (cnt #)	The count of measurements for this marker (i.e. persons) from experiment 0. The experiment name is shown on the second line.
7, 8	exp 1 (cnt #)	The count of measurements for this marker (i.e. persons) from experiment 1. The experiment name is shown on the second line.
7, 8	below #	The number of entries in the concordance matrix

The rows of the concordance matrix come from experiment 0 (*dataset2.txt* [in this case]) and the columns come from experiment 1 (*dataset1.txt*). The dimensions are labeled by the allele pairs and include an extra value "00" used to represent the unmeasured allele value. Its pretty easy to see that there are 5 elements on the diagonal – representing agreement between allele values from each experiment. Off diagonal elements would indicate disagreement. The "00" row shows there are a total of four non measured values from experiment 0 that are measured in experiment 1. The ("00", "00") position shows that there is one person that has no measurement for this marker in either experiment.

Finally looking just at the measured entries (not "00"), two summary lines (lines 14 & 15) indicate the number of elements along the diagonal vs the rest of the matrix and the transpose vs the rest of the matrix.

2.5.6.3 Verbose Verify Run

It might be that the summary data is insufficient and you want the complete details for each person and marker when there are multiple measurements. Adding the **-v** flag to the above command does this. Still more data are added to the output and (only the new data) are shown below. There are three new sections, the first to report on concordance for all measurements, the second to report on concordance for valid (non-zero) measurements and the third to report on conflicts.

geno *example.ds2.snp.cfg* --verify -v

```
\\ ===== Starting the "concordance" subsection
\\ Concordance across all multiple observed genotypes:
\\ pedigree  person  snp name   genotypes
\\        1       1  rs1073516
                                    [[2, '0/0 project/exampletech2/dataset2.txt@1'],
                                         '0/0 project/exampletech1/dataset1.txt@1']]
\\        2   2_101  rs1073516
                                    [[2, 'A/G project/exampletech2/dataset2.txt@1'],
                                         'A/G project/exampletech1/dataset1.txt@1']]
\\        2   2_201  rs1073516
                                    [[2, 'A/G project/exampletech2/dataset2.txt@1'],
                                         'A/G project/exampletech1/dataset1.txt@1']]
\\        2   2_301  rs1073516
                                    [[2, 'A/A project/exampletech2/dataset2.txt@1'],
                                         'A/A project/exampletech1/dataset1.txt@1']]
\\        2   2_401  rs1073516
                                    [[2, 'A/G project/exampletech2/dataset2.txt@1'],
                                         'A/G project/exampletech1/dataset1.txt@1']]
\\        2   2_501  rs1073516
                                    [[2, 'G/G project/exampletech2/dataset2.txt@1'],
                                         'G/G project/exampletech1/dataset1.txt@1']]
\\ Concordance count = 6
\\
\\ ===== Starting the "concordmissing" subsection
\\ Missing in some but concordance otherwise:
\\ pedigree  person  snp name   genotypes
```

```
15 \\          1       2  rs1073516
                                  [[1, '0/0 project/exampletech2/dataset2.txt@1']],
                                   [1, 'A/G project/exampletech1/dataset1.txt@1']]
16 \\          1       3  rs1073516
                                  [[1, '0/0 project/exampletech2/dataset2.txt@1']],
                                   [1, 'A/A project/exampletech1/dataset1.txt@1']]
17 \\          1       4  rs1073516
                                  [[1, '0/0 project/exampletech2/dataset2.txt@1']],
                                   [1, 'A/G project/exampletech1/dataset1.txt@1']]
18 \\ Missing in some but concordance otherwise count = 3
19 \\
20 \\ ===== Starting the "discordance" subsection
21 \\ Discordant genotypes:
22 \\ pedigree  person  snp name    genotypes
23 \\ Discordant genotypes count = 0
24 \\
```

The new sub-sections are grouped on several lines:

lines	observations
2-10	concordance report details
12-18	concordance report details for measured (non-zero) values only
20-23	discordance report details

The lines in each section are interpreted as:

lines	field	explanation
	pedigree	pedigree being displayed
	person	person being displayed
	snp name	marker being displayed
	genotypes	A list of lists: The inner list has a count of the number of entries followed by the allele data for the next "count" measurements. The allele data are: "letters" indicated as "x/y", followed by the project, the technology, and the experiment separated by "/" and then an "@" followed by the active value. All items in the inner list have the same alleles, but different technology or experiments.

You can use **-d** instead of **-v** (**debug** versus **verbose**) to get a more database centric view of the above data. Note: The active values for an entry will be explained in a later section (2.6.3).

2.5.6.4 Commit

Its finally time to add the data to the database:

> **geno** ***example.ds2.snp.cfg*** **--commit**

just does the commit and reports (at the end of some summary data)

```
Commiting Changes to the Database.
```

2.5.6.5 More Pesky Details

If you type:

> **geno** ***example.ds2.snp.cfg*** **--verify --v**

you will get a very confusing output. **--verify** compares the new data in a file with all the existing data in the database. So if we run **--verify** after a **--commit**, we proposing to add the data again to the database which would cause a duplication of all the measurements that were previously entered.

The option **--compare** is what is needed here. **--compare** will only examine data that is already in the database. It uses the **experiment** file to select what person/pedigree/markers to look at. So

> **geno** ***example.ds2.snp.cfg*** **--compare -v**

will generate exactly what we saw previously with **--verify** before the **--commit** because the data are now in the database.

What happens if you run

> **geno** ***example.ds2.snp.cfg*** **--commit**

again. dbVOR will recognize that the data are already in the database and NOT enter it again. (Try it.) But suppose somehow the original data were incorrect and you really want to replace them. (Perhaps, some data was recorded incorrectly or you have decided to change from number to letter allele labels.) Adding the **--replace** option will reload the entries from the **experiment** file.

geno ***example.ds2.snp.cfg*** **--commit --replace**

Of course, you might somehow discover that you need to totally delete some entries of the **experiment** file. Changing **--replace** above to **--delete** will remove the original **experiment** file data. Then you can edit the experiment file and remove the entries and resubmit the **--commit**. Alternatively, you could just type:

dbvor DeleteExperiment --project=*project*

--technology=*exampletech2* --experiment=*dataset2.txt* --commit

to delete all the genotype data for the indicated experiment.

2.5.7 Problems during Merging Markers

In the past examples, we were adding new markers or markers for which the new experimental values agreed with the existing values. These are the simple cases. Let us now look at a new data set for the same two markers that we have been using, *dataset3.txt*:

```
pedigree    person    rs72921657-1  rs72921657-2  rs1073516-1  rs1073516-2
1           1         0             0             0            0
1           2         C             A             0            0
1           3         A             A             0            0
1           4         A             C             0            0
2           2_101     0             0             1            2
2           2_201     A             C             2            1
2           2_301     A             A             2            2
2           2_401     C             A             2            1
2           2_501     C             C             1            1
3           1         A             A             1            2
3           2         C             C             1            1
3           3         A             C             2            1
3           4         0             0             2            2
```

The configuration file for this data is similar to the previous dataset's configuration file:

```
technology:  exampletech1
experiment:  dataset3.txt
pedigree:    1
person:      2
type:        wide
marker:      3
```

When we run

geno example.ds3.snp.cfg --verify

we will discover problems and new printout sections that explain these issues. In the log listing, after the display of the command arguments and configuration but before the summary of pedigree data and marker data is a new subsection, *genotypewide*: (I have elided lines 32 through 62 of this section because they introduce nothing new for the explanation.)

```
 1 \\ project: project, id 1
 2 \\ technology: exampletech1, id 1
 3 \\ experiment: "dataset3.txt", id 0
 4 \\
 5 \\ file "./dataset3.txt"
 6 \\
 7 \\ ===== Starting the "genotypewide" subsection
 8 \\
 9 \\ Line         6: pedigree:person 2:2_101 [marker rs1073516]
10 \\ verify: different genotypes found.
11 \\ new: #0, db id        0, allele 1/2, project/exampletech1/dataset3.txt
12 \\  db: #1, db id        5, allele A/G, project/exampletech1/dataset1.txt
13 \\  db: #2, db id       71, allele G/A, project/exampletech2/dataset2.txt
14 \\
15 \\ Line         7: pedigree:person 2:2_201 [marker rs72921657]
16 \\ verify: different genotypes found.
17 \\ new: #0, db id        0, allele A/C, project/exampletech1/dataset3.txt
18 \\  db: #1, db id       72, allele C/C, project/exampletech2/dataset2.txt
19 \\
20 \\ Line         7: pedigree:person 2:2_201 [marker rs1073516]
21 \\ verify: different genotypes found.
22 \\ new: #0, db id        0, allele 2/1, project/exampletech1/dataset3.txt
23 \\  db: #1, db id        6, allele G/A, project/exampletech1/dataset1.txt
```

```
24 \\  db: #2, db id       73, allele A/G, project/exampletech2/dataset2.txt
25 \\
26 \\ Line          8: pedigree:person 2:2_301 [marker rs72921657]
27 \\ verify: different genotypes found.
28 \\ new: #0, db id        0, allele A/A, project/exampletech1/dataset3.txt
29 \\  db: #1, db id       74, allele C/A, project/exampletech2/dataset2.txt
30 \\
31 \\ Line          8: pedigree:person 2:2_301 [marker rs1073516]
...
63 \\
64 \\ ===== The text above shows the first 10 records from a total of 11 \
     records in the log file for the "genotypewide" subsection
65 \\
66 \\
67 \\ 13 members included; 0 members excluded
68 \\
```

What is important here, is that during a **verify/compare** operation, if the new data are different from the existing data in the database, you will be informed[23].

2.5.7.1 Lets look at a block of output.

lines	observations
9	shows the input line number and the pedigree:person and marker being processed
10	indicates that the new genotype conflicts with the existing ones
11-14	shows the conflicts
11	new: indicates this is the new data; so the database entry id (db id) is 0 (not inserted yet). Finally, the allele values and project/technology/experiment are shown
12,13	as above except there are real database id's for these records<br>Note: alleles A/G and G/A are the same for our purposes and do not represent a conflict. They were just entered into the database that way.

This scenario is repeated on lines 20 thru 25 and in several other sets not shown (9 in total). The problem illustrated is that the lab has reported the alleles now with the numbers: 1 and

[23] Actually, you can also ask to be informed if the new data are the same as existing data. To do this add the switch, **--verify_show_all**.

2 but with letters previously. If your lab never does this, skip this subsection. But our lab does occasionally report alleles different ways. Sometimes we can obtain the letter alleles from them but sometimes not. dbVOR can help here. (We will discuss line groupings like 15-19 in a later section (2.5.7.4).)

2.5.7.2 Using the concordance matrix

Towards the end of the log listing, we find:

```
105 \\ ===== Starting the "marker_allconcordance" subsection
106 \\ Summary by marker:
107 \\ ===== "marker_allconcordance" record 1
108 \\                                             missing or
109 \\ marker snp name    dup missing concordant concordant discordant
110 \\     1 rs1073516     13       1          0          3          9
111 \\     1 rs1073516  VS: exp 0 (cnt 13) & exp 1 (cnt 9): below 9;
112 \\    exp 0 == dataset3.txt,  exp 1 == dataset1.txt
113 \\                                          00   AA   AG   GG
114 \\                                     00    1    1    2    0
115 \\                                     11    0    0    0    1
116 \\                                     12    0    0    3    0
117 \\                                     22    0    1    0    0
118 \\ diagonal      3, rest     2 [1:A, 2:G], marker rs1073516, \
        exp 0:1 (dataset3.txt:dataset1.txt)
119 \\ transpose     5, rest     0 [1:G, 2:A], marker rs1073516, \
        exp 0:1 (dataset3.txt:dataset1.txt)
120 \\
121 \\     1 rs1073516  VS: exp 0 (cnt 13) & exp 3 (cnt 13): below 13;
122 \\    exp 0 == dataset3.txt,  exp 3 == dataset2.txt
123 \\                                          00   AA   AG   GG
124 \\                                     00    4    0    0    0
125 \\                                     11    0    0    0    2
126 \\                                     12    0    0    5    0
127 \\                                     22    0    2    0    0
128 \\ diagonal      5, rest     4 [1:A, 2:G], marker rs1073516, \
        exp 0:3 (dataset3.txt:dataset2.txt)
129 \\ transpose     9, rest     0 [1:G, 2:A], marker rs1073516, \
        exp 0:3 (dataset3.txt:dataset2.txt)
130 \\
131 \\     1 rs1073516  VS: exp 1 (cnt 9) & exp 3 (cnt 13): below 9;
132 \\    exp 1 == dataset1.txt,  exp 3 == dataset2.txt
133 \\                                          00   AA   AG   GG
134 \                                      00    1    0    0    0
```

```
135 \\
136 \\
137 \\
138 \\ diagonal      5, rest    0
139 \\ transpose     3, rest    2
140 \\
```

AA	1	1	0	0
AG	2	0	3	0
GG	0	0	0	1

Back in section 2.5.6.2, we introduced a concordance matrix for SNP *rs1073516* that compared the data from *dataset1.txt* with that of *dataset2.txt*. (And note that this same matrix can be seen on lines 131 thru 140 above.) Lines 111 thru 120 and 121 thru 130 compare the new experiment, *dataset3.txt*, with *dataset1*.txt and *dataset2.txt*, respectively. The mapping of letter allele to number allele is easily obtained from the matrix. It is also summarized on lines 118 & 119 and 128 & 129. The summaries show that if you look down the diagonal of the matrix, you will account for 3 of the 5 conflict entries in the first case and 5 of the 9 in the second. This is not very good agreement. The diagonal of the first concordance matrix indicates a mapping of 1 to A and 2 to G. On the other hand, if you look at the transposed diagonal line [sloping up from left (22, AA) to right (11, GG)] of the matrix, there is perfect agreement. We can be fairly confident that 1 was coded for G and 2 for A for this new data. Now, in practice, the counts representing samples are much larger and there are always a few outliers so that you do not see perfect matches of all samples on the diagonal or transpose. Human expertise is necessary to look at the matrices and make the call. In our case, we edited *dataset3.txt* to create *dataset3p.txt* using the 1 to G and 2 to A mapping for SNP rs1073516. Of course, we might want to record the data as it was provided to us.

2.5.7.3 Resolvable Disagreements

An alternative exists to creating the *dataset3p.txt* (as explained above). We could create a mapping table that says for experiment *dataset3.txt* and marker *rs1073516*, replace allele *1* with *G* and allele *2* with *A*. The table, *allelemap.txt*, is illustrated below, but in typical usage would have many more rows.

```
dataset3.txt   rs1073516    1:G        2:A
```

The command, **dbvor allelemap**, will load the mapping data. Once the data is loaded into the database, whenever the alleles for rs1073516 in experiment dataset3.txt are fetched the values will be transformed. To run **dbvor allelemap**, we need to specify the configuration information:

**--input**	the input file, *allelemap.txt*
**--experiments**	the column number of the experiment name field
**--marker**	the column number of the marker name field
**--map**	the column number of an allele mapping field (repeat as needed)
**-active**	specifies active flag value to store in database for this entry
**--maptype**	1 -> indicates that the mapping applies to genotype data. (default)<br>2 -> indicates that the mapping applies to GWAS data.<br>3 -> indicates that the mapping should be use for the marker after the data conflicts (section 2.6.3) are resolved.<br>4 -> like 1 but the allele mapping is reversed . The example above would be processed as G->1 and A->2.<br>5 -> like 2 but the allele mapping is reversed.<br>6 -> line 3, but the allele mapping is reversed.

Extra fields, needed for any configuration, were explained in Section2.4.1. The configuration file, ***allelemap.ds3.cfg*** , for our data is:

```
input:          allelemap.txt
experiments:    1
marker:         2
allelemap:      3
allelemap:      4
```

Typing:

dbvor allelemap *allelemap.ds3.cfg –commit*

on the command line will update the permanent mapping table. Note: you need to run:

geno example.ds3.snp.cfg --commit

to enter the new markers into the database first.

2.5.7.4 Unresolved Disagreements

Going back to the log listing, we see lines 15 thru 19 and 26 thru 30 illustrates a different problem:

```
15 \\ Line          7: pedigree:person 2:2_201 [marker rs72921657]
16 \\ verify: different genotypes found.
17 \\ new: #0, db id        0, allele A/C, project/exampletech1/dataset3.txt
18 \\  db: #1, db id       72, allele C/C, project/exampletech2/dataset2.txt
19 \\
```

Here, the alleles for SNP rs72921657 from experiment *dataset3.txt* and experiment *dataset2.txt* are just different. There is no programmatic way to decide which is correct. The **commit** to the database will just store the new values and leave the old value. When the information is extracted from the database via **genout**, several mechanisms are available to choose which allele pair to use. By default, the unmeasured value, 0/0, is returned when there is a conflict.

These differences are also summarized in the "marker_allconcordance" section (below).

```
\\ marker snp name   dup missing concordant concordant discordant
\\       2 rs72921657  13       3          8          0     2
\\       2 rs72921657 VS: exp 0 (cnt 13) & exp 3 (cnt 13): below 13;
\\      exp 0 == dataset3.txt,  exp 3 == dataset2.txt
\\                                              00   AA   AC   CC
\\                                         00    3    0    0    0
\\                                         AA    0    2    1    0
\\                                         AC    0    0    4    1
\\                                         CC    0    0    0    2
\\ diagonal       8, rest      2
\\ transpose      4, rest      6
\\
```

It is easy to see the off diagonal points (AA, AC) and (AC, CC) in the matrix.

2.5.7.5 Final commit

As we explained earlier, we have chosen to rewrite *dataset3.txt* into *dataset3p.txt* and make a corresponding configuration file. With it we finally, commit these new data to the database:

geno example.ds3p.snp.cfg --commit

All the data and configuration files are provided in the ***data*** directory, as is our custom.

2.5.8 Coda

There are a few more dbVOR features that are useful but we have excluded in the previous illustrations. These mostly apply to the **geno** program for allele data entry.

2.5.8.1 Restricting Pedigree and Persons

Suppose, samples for some people are bad and should not be entered into the database. Since the experiment file contains these samples, they will be added to the database. There are two arguments: **--include** and **--exclude** which lets you control which samples to add/exclude by person and pedigree. Typically, these arguments would be placed in the configuration file. Each argument can be followed by multiple person-pedigree pairs separated by commas (",") and/or there can be multiple **include** and/or **exclude** lines in the configuration file, viz.:

```
#include/exclude: pedigree:person
include: 2:, 3:
exclude: 101
exclude: 201
exclude: 3:1, 3:2
```

As shown in the comment in the above example, people are specified by writing the pedigree, a ":", and the associated person. You can specify both a pedigree and person to identify a unique individual. If you specify just a pedigree and no person, then all persons of that pedigree will be considered for inclusion/exclusion. If you specify a person and no pedigree, or if you just specify a token without an initial ":", then that person identifier in any pedigree will be considered for inclusion/exclusion.

The meaning of **exclude** is simple: the specified people should not be processed. **include** is a bit more complicated: it indicates that the specified people and only those people should be considered. Thus only people explicitly **include**d from the input file can be included. So if you want to include most people of a pedigree, you can **include** by pedigree and **exclude** by the specific person. Thus, the above configuration file chooses the non-founders for pedigrees 2 and 3.

2.5.8.2 Splitting up the configuration file

If there are a lot of people to be included/exclude in a configuration, it might be convenient to list these entries in a separate file and to include this file in the normal configuration file. The argument, **source**, specifies an additional file to process. Note: all **source** files are processed after all of the other configuration file arguments are processed and all **source** files are processed in the order they appear in the configuration file.

2.5.8.3 Changing the log file name

The **log** file is always stored in the directory specified with the **--dir** argument. The argument, **--log**, specifies the log file name; otherwise, the **log** file name is the configuration file stem (the trailing *.cfg* is removed) with the date/time added and with a trailing suffix of "*.log.txt*", viz.

example.ds3p.snp_20110912_12_17_34.log.txt

The above is an example from the previous ***example.ds3p.snp.cfg*** run. Note: default **log** files names are obviously unique.

2.5.8.4 Less log output

The flag, **nostat**, will inhibit **geno** and **genout** from generating summary statistics. We believe that you almost always do not want to use this flag.

2.6 genout: Data Extraction

2.6.1 Data Summary

Although we could reuse the configuration file we used for **genout** back in section 2.4.4, we will create a new one and further rename the output as shown below in ***out.cfg***:

```
type:      half
output:    out
```

running

genout out.cfg

produces the output file, ***out.txt*** below:

#PEDIGR	PERSON	FATHER	MOTHER	SEX	trait1	trait2	rs72921657	rs1073516
1	1	0	0	1	NA	NA	00	00
1	2	0	0	2	2	2	AC	AG
1	3	1	2	1	2	1	AA	AA
1	4	1	2	2	1	1	AC	AG
2	101	0	0	1	NA	NA	00	AG
2	201	0	0	2	2	2	00	AG
2	301	101	201	1	2	2	00	AA
2	401	101	201	2	1	2	AC	AG
2	501	101	201	2	2	2	CC	GG
3	1	0	0	1	NA	1	AA	AG
3	2	0	0	2	NA	2	CC	GG
3	3	1	2	1	NA	1	AC	AG
3	4	1	2	2	NA	NA	00	AA

This file aggregates all the data we have processed thus far. The data can be presented in numerous other ways.

2.6.2 Controlling Output Format

We specify the output format to use with the **--type** argument. The values are:

**value**	**meaning**
wide	each allele is placed in a separate column of the output file
half	each allele **pair** is placed in a separate column of the output file
ped	PLINK ped format
tped	PLINK tped format
lgen	PLINK lgen format: one pedigree/person/marker allele per line
bed	PLINK binary format, normally snp-major order but **--person_order** forces individual-major order.
long	like PLINK lgen format, each line specifies a pedigree/person/marker and one allele pair
mega2	mega2 file format

2.6.3 Resolving Data Conflicts

In section 2.5.7.4, we noted that there was an unresolved measurement conflict for pedigree 2 person 2_201 concerning marker rs72921657. In the above listing, we see an allele pair of 00 in that field. But suppose we know that technology **exampletech2** is always more accurate than technology **exampletech1**. Alternatively, we might know that experiment ***dataset3.txt*** was not very good and that we would prefer any other measurement than ones from ***dataset3.txt***, if there is a choice. The mechanism for indicating these kinds of choices is accessed via the "**active**" argument. Each genotype measurement is labeled with the value of the **active** argument that was set when the data was processed. Thus we could have indicated:

> **geno** ***example.ds2.snp.cfg*** **--commit --active=2**

or

> **geno** ***example.ds3.snp.cfg*** **--commit --active=0**

The default value for the **--active** flag is 1.

The way the genotypes are combined when there is more than one *different* entry for a given pedigree/person/marker is that if the active value is 0, the allele pair is ignored[24], for all other allele pairs only the one(s) with the numerically highest active flag are selected[25]. If there is only one pair with the highest active flag, that pair is chosen. If there are more than one allele pair and they are different, the unmeasured value (00) is used. The above two examples, then, realize the results of our motivation paragraph. The first says to prefer experiment *dataset2.txt* while the second says to not use experiment *dataset3.txt*.

2.6.3.1 Setting "active" flag

An alternative to using the above two commands is **dbvor active**:

> **dbvor active --experiment** *dataset2.txt* **--technology=***exampletech2* **--project=***project* **--commit --update --active=2**

and

[24] The flag, **use_inactive**, can be set to force pairs with active values of 0 to be used.

[25] This feature requires the **filter_by_active** flag to be True which is the default value.

dbvor active --experiment ***dataset3.txt*** **--technology=*exampletech1* --project=*project* --commit --update --active=0**

respectively. They change the **--active** flag without rerunning **geno**[26]. **dbvor active** is more powerful than rerunning **geno** because it lets you set the active flag as needed on individual pedigree/person/marker entries. If the argument **--input** is supplied, its value is a file that specifies a four tuple of pedigree, person, marker and active value. The configuration data are:

**--input**	the input file
**--pedigree**	the column number of the pedigree field
**--person**	the column number of the person field
**--marker**	the column number of the marker name
**--active**	the column number of the active value
**--project**	name of the project
**--technology**	name of the technology used
**--experiment**	name of the experiment, viz. dataset2.txt

The **dbvor active** configuration file***, active.cfg*** could look like:

```
input:       active.txt
pedigree:    1
person:      2
marker:      3
active       4
project:     example
technology:  exampletech2
experiment:  dataset2.txt
```

and the data in ***active.txt*** could be

```
1    1    rs1073516   7
2    201  rs72921657  14
3    3    rs1073516   21
```

This would change the active flag for one person in each pedigree to some multiple of 7; ideally, the changes for your experiments would be less whimsical.

[26]**dbvor active** does not have access to the genotype data; all it can do is update the active flag. Thus it must set the **--update** flag to request update vs replace semantics.

2.6.4 A Selection Example

genout selects different subsets of the data from the dbVOR database. This is done by specifying patterns for which to select the genotype data. Let us start with another whimsical example. We have chosen to specify many arguments on the command line that would probably be more sensibly included in the configuration file. We have also added the **--verbose** flag to get a little more feedback during processing. By typing:

genout out.cfg --include 2: --exclude 101 --exclude 201

--marker rs1073516

--trait "trait1|value|2" --trait trait2 --trait "gender|1" -v

The output file, ***out.txt***, is just a single line:

#PEDIGR	PERSON	FATHER	MOTHER	SEX	trait1	trait2	rs1073516
2	301	101	201	1	2	2	AA

The **log** listing is included below. We will follow it with our customary commentary.

```
\\ Config: parsed ~/.dbvorrc for config options
```

```
\\ START 09/03/2013 15:58:17, dbVOR version 0.55
\\
\\ cmd: /Users/rbaron/bin/genout out.cfg
     --include 2: --exclude 101 --exclude 201
     --marker rs1073516 --trait trait1|value|2
     --trait trait2 --trait gender|1 -v
\\ cwd: /Users/rbaron/dbVOR/doc/Book/data
\\
\\                 host: 'localhost'
\\                 user: 'example'
\\                   db: 'exampledb'
\\                  dir: './'
\\              project: 'project'
\\               config: 'out.cfg'
\\                 type: 'half'
```

```
\\                output: 'out'
\\               exclude: ['101', '201']
\\               include: ['2:']
\\                marker: ['rs1073516']
\\                 trait: ['trait1|value|2', 'trait2', 'gender|1']
\\               verbose: 1
\\ project project, id 1
\\
\\ collecting distinct markers from genotypes   0.000 sec
\\ collecting distinct markers from gwas   0.000 sec
\\
\\ ===== Starting the "db_position" subsection
\\ Total Markers: Genotypes       1, GWAS       0
\\
\\ ===== Starting the "exclmember" subsection
\\ excluding member 1:1
\\ excluding member 1:2
\\ excluding member 1:3
\\ excluding member 1:4
\\ excluding member 2:101
\\ excluding member 2:201
\\
\\ ===== Starting the "traitvalue" subsection
\\ skipping: pedigree:person 2:401 (id 8) trait "trait1" (id 1)
     value 1 not in allowed list ('2',)
\\
\\ ===== Starting the "traitgender" subsection
\\ skipping: pedigree:person 2:501 (id 9) gender
     value 2 not in allowed list ['1']
\\ excluding member 3:1
\\ excluding member 3:2
\\ excluding member 3:3
\\ excluding member 3:4
\\ members 1, markers 1 (snps 1, gwas 0)
\\
\\
\\ ===== Starting the "memberXmarker" subsection
\\    1/1: member       2:301     (#    7)        1 snps   0.000 sec
```

```
50 \\ total 1, possible_conflicts 1 (3), chose active 0,
      concordance 1, ignore zero 0, conflicts 0
51 \\
52 \\
53 \\
54 \\ ===== Starting the "marker_prune" subsection
55 \\ pruning markers: none pruned - 1 entries available
56 \\
57 \\ ===== Starting the "prune members" subsection
58 \\ pruning members: none pruned - 1 entries available
59 \\
60 \\ Work Summary:
61 \\  Created "half" format file: out.txt
62 \\
63 \\ SUCCESS: dbVOR version 0.55
64 \\
65 \\ END 09/03/2013 15:58:17, DURATION 0.061 sec
```

Lines 15, 16, 17 and 18 show the selections. Note: all of these selectors are lists and can contain more than one request.

**lines**	**observations**
15	requests that members 101 & 102 (for any pedigree) be excluded (but we are only processing pedigree 2 ...)
16	requests that only pedigree 2 be included
17	requests a list of marker(s) (You can also select by chromosome and/or position.)
18	requests that *trait1* have the value 2, allows any value of *trait2*, and requests that *gender* have the value 1. The syntax is explained in section 2.6.5.3.

Since we included the **--verbose** flag, further details were reported that we will comment on below:

lines	observations
28-46	shows all pedigree:persons from the database that are excluded and the reason why. The record is complete in the log file but truncated here. See line 47.
36-38	shows pedigree:persons that do not match trait constraints. Here the value is 1 but 2 was requested. (There can be multiple allowed values.)
39-40	shows pedigree:persons that do not match the gender constraint. Here the value is 2 but 1 was requested.
48-51	shows that there is only one person and one marker left. That the one marker appears 3 times in the database (dataset1.txt, dataset2,txt and dataset3.txt) and the values are all in concordance.
54-59	indicates that the members and markers were not further reduced because the all items were in the database. (In a larger study, some markers might not be measured for all people.)

2.6.5 Selector

Except for the **--exclude** option, all the selectors indicate some database entries to choose. If an entry is not requested, it is not part of the output. *However*, if no markers or no people are specified, the obvious default is chosen – to use all the of the markers or all of the people, respectively.

We have seen many of these selectors earlier in our toy example. Keep in mind that there are several ways to indicate repeats of a particular selector. Both

marker: rs72921657, rs1073516

and

marker: rs72921657

marker: rs1073516

choose the same set of two markers. An exposition of the selectors follows:

2.6.5.1 Select by marker, chromosome, etc.

argument	sample value	meaning
**--marker**	rs1073516	lists of markers to include
**--chromosome**	1	includes all markers in the database on a given chromosome
**--chrrange**	*chr*:*start*..*end*	includes all markers on chromosome *chr* between positions *start* thru *end*. Note: *chr* is followed by a ":" and *start* and *end* are separated by "..". Either *start* or *end* can be missing, in which case *start* is 0 and *end* is "the end"
**--chrCMrange**	*chr*:*start*..*end*	interprets values as above but the average genetic distance is used rather than the absolute position. Note: the numbers are decimal and can include fractions.

2.6.5.2 Select by member

argument	sample value	meaning
**--member**	pedigree:person	includes only those people with the indicated pedigree and person
**--include**	pedigree:person	includes only those people with the indicated pedigree and/or person as explained below.
**--exclude**	pedigree:person	excludes only those people with the indicated pedigree and/or person from the list of otherwise included persons
**--fill_pedigree**		after persons are selected by the other mechanisms explained in this table (and the trait selection below), add all the other persons in each of the selected pedigrees unless they are explicitly **--exclude**d (above).

--member request a specific pedigree and person.

--include and **--exclude** are exactly the same mechanism that was introduced in section 2.5.8.1 for filtering entries for the **geno** program. A ":" is used to separate the two parts: the

pedigree and the person. As explained previously, specifying both a pedigree and person will identify a unique individual. You may also specify just the pedigree to select all members in the pedigree (Indicated as "pedigree:", viz. omit the person specifier) or just the person to select that person id in any and all pedigrees that have the id (indicated as ":person", viz. omit the pedigree specifier, You can also omit the ":" in the latter case.)

2.6.5.3 Select by trait

In the examples below, the trait *name* is separated from the special keyword, **value**, and then from the possible values by the character "|". (**value** needs to be present so the expression looks like the marker specialization illustrated in the next section.)

<table>
<tr><th>argument</th><th>sample value</th><th>meaning</th></tr>
<tr><td>--trait</td><td>name|value|v1|v2
or
name|value|v1
name|value|v2</td><td>includes persons where the corresponding trait name has the values v1 or v2. You may indicate multiple values as done here by having them on the same line and separating them with "|". Or you can have one value specified on each of several lines as shown in the box to the left.</td></tr>
<tr><td>--trait</td><td>gender|value|v1|v2</td><td>as above. genout treats sex as a trait. (Though it is maintained as part of the family structure internally.)</td></tr>
<tr><td>--trait</td><td>name</td><td>selects name to appear in the output. Its value is not constrained in any way.</td></tr>
</table>

If at least one trait is selected this way, only selected traits will be output. Otherwise, the default is to show all traits.

2.6.5.4 Select by ...

What's left?

In the examples below, the marker *name* is separated from the special keywords, **technology** and **experiment**, and then from the possible values by the character "|".

<table>
<tr><th>argument</th><th>sample value</th><th>meaning</th></tr>
<tr><td>--technologies</td><td>exampletech1</td><td>indicates which technologies are to be included in the results</td></tr>
<tr><td>--experiments</td><td>dataset3.txt</td><td>indicates which experiments are to be included in the results</td></tr>
<tr><td>--marker</td><td colspan="2">name|technology|T1</td></tr>
<tr><td></td><td></td><td>is a specialization of selecting a marker but selects marker name measured with the technology T1. You may list multiple technology values, by separating them with “|”</td></tr>
<tr><td>--marker</td><td colspan="2">name|experiment|E1</td></tr>
<tr><td></td><td></td><td>as above, but selects by the experiment of the marker.</td></tr>
</table>

2.6.6 Filtering Experiment Data

In any large experiment, it is not uncommon that some of the data is bad. We would then like to not include the bad data in our results. It might be rather tedious to exclude data, by only including the “good” data around the bad ranges. So we, additionally, give you the ability to explicitly exclude data.

The first option, below, applies to selected markers for all the people of the experiment, *exp*. It could be used if some of the plate wells did not work or some of the markers were actually not polymorphic. The rest of the **suboptions** apply only to the selected person(s) of the experiment, *exp,* and may affect markers, whole chromosomes, or subranges of a chromosome. They could be used if particular markers did not type type well, for some people or ranges were not called well.

argument	suboption | value	meaning
**--experiment**	*exp*|**exclude_marker**|*M1:Chr1*|*M2:Chr2*	
		excludes the marker named *M1* for all persons of experiment, *exp*. You may optionally specify the chromosome of the marker by following the marker name, *M1*, by a ":" and the chromosome. You may list multiple **values**, by separating them with "|" or using separate lines.

argument	suboption | value	meaning
**--experiment**	*exp*|**exclude_person**|*Pped:Pper*	
		excludes all markers for person *Pped:Pper* in experiment, *exp*. Both *Pped* and *Pper* must be specified. You may list multiple **values**, by separating them with "|" or using separate lines.
**--experiment**	*exp*|**exclude_genotype**|*Pped:Pper:marker*	
		as above, but only exclude the given marker, *marker* for the indicated person. You may list multiple **values**, by separating them with "|" or using separate lines.
**--experiment**	*exp*|**exclude_chromosome**|*Pped:Pper:Chr*	
		excludes all markers on chromosome, *chr*, for person *Pped:Pper* in experiment, *exp*. Unlike earlier usage, both *Pped* and *Pper* must be specified. You may list multiple **values**, by separating them with "|" or ...
**--experiment**	*exp*|**exclude_chrranger**|*Pped:Pper:Chr:start..end*	
		as above, but only exclude markers with base pair position between *start* and *end* inclusive on chromosome *Chr*. *start* may be omitted and defaults to 0, while *end*, if omitted defaults to a really large number. You may list multiple **values**, by separating them with "|" or ...

argument	suboption | value	meaning
**--prune_members**		
		This is a filtering operation but different from those above. It removes all those members from output that have no markers, e.g. founders might not be labeled.<br>NOTE: If **--fill_pedigree** is also set, members are not removed if at least one other member of the pedigree has some markers.

2.6.7 Further Output Customization

There are several aspects of the output processing/formatting that are controlled by further arguments:

argument	meaning
**--no_traits**	ignore any available trait data. With this flag, it is valid to have no trait data.
**--no_markers**	ignore marker request in the config file. In addition, if there is no marker request do not default to all markers, default to NO markers. With this flag, it is valid to have no marker data.
**--separator**	supplies the output file column separator for wide/half/long
**--OFS**	is an alternate name for the **--separator**
**--short**	does not explicitly show unknown/unmeasured marker values, in long format. The long format needs no place holders like wide format does, since every line contains the person, marker and allele info.
**--funny**	uses special characters to indicate data problems, for wide/half/long formats:<br>Q indicates no trait value found.<br>Z indicates no samples measured.<br>F indicates conflicting allele pairs found.
**--person_order**	turns off statistics and other processing so as to minimize the memory footprint during processing. After all the markers for each person are processed, all data and meta data is purged. It is compatible with bed, ped, lgen, and mega2 formats. It will cause PLINK bed to produce a "individual major" binary file rather than the "snp major".

<table>
<tr><th>argument</th><th>meaning</th></tr>
<tr><td>--heterozygote</td><td>indicates how to order individual alleles in the heterozygous case.
<table>
<tr><th>value</th><th>meaning</th></tr>
<tr><td>unchanged</td><td>use order presented from the database</td></tr>
<tr><td>lexical</td><td>sort the alleles alphabetically</td></tr>
<tr><td>keep-allele-order</td><td>use allele order of first person in the database that has this marker</td></tr>
<tr><td>founders</td><td>place allele with lower minor allele frequency first. Frequency computed over just the founders.</td></tr>
<tr><td>allsubjects</td><td>place allele with lower minor allele frequency first. Frequency computed over all subjects.</td></tr>
<tr><td>NOTE</td><td>These last 3 option perform exactly like the same named PLINK options.</td></tr>
</table>
</td></tr>
</table>

2.6.8 Modified operations for genout

argument	meaning
**--unobserved**	**genout** counts the number of missing markers per person. The only output from **genout** is a table showing:<br>*person pedigree <total markers> <missing markers> <found markers>*<br>The table is sorted by <*missing markers*> then *pedigree* and *person*.

2.6.9 genout Statistics

The flag **--statistics** (also **--stat**) produces statistics derived from merging multiple experiments while the data filtered by **genout** is being processed. This feature is not on by default because there may not be enough memory to save the statistics for a GWAS size **dbVOR** database.

argument	meaning
**--statistics**	produces statistics, concordance matrix, etc. like **geno verify**.
**--stat**	is the same as **--statistics**

2.7 Adding genome-wide data

We describe here how to enter genome-wide data, taking advantages of the efficiencies implemented in dbVOR to store such large-scale data in blocks of genotypes. As an examples, we use data from some genome-wide association studies (GWAS). While strictly speaking, GWAS data is not necessarily the same thing as genome-wide data, we use these terms here exchangeably.

The previously illustrated technique for entry of small-scale marker data (Section 2.4.3) will work for only the smallest of genome-wide association study (GWAS) data sets. A small Affymetrix GWAS with 11,457 markers measured on 1,673 samples representing 19 million person/marker records loads in 1 hour and 25 minutes[27]. A hypothetical data set of 1,000,000 markers and 1,000 samples is 51 times larger and should be that many times slower to load. Thus, we are forced to take a different approach in handling genome-wide data by storing blocks of genotypes in the database. Using this approach, for the small GWAS example, the load time drops to 2 minutes and 7 seconds and date are stored in 61,722 records instead of 19 million records.

You may combine genome-wide and targeted data in the same database. All the mechanisms for allele conflict resolutions and data extraction discussed earlier (Sections 2.5.6.2, 2.5.7.2, 2.5.7.3, 2.5.7.4) are available.

There are two general work flows for adding genome-wide data, which depend on the input format. The first work flow is applicable to Illumina-format and Affymetrix-format data, while the second work flow is applicable to PLINK binary (bed) format data.

First genome-wide work flow:

Beginning in the immediately following section (2.7.1), we show the steps to load Illumina data (and/or Affymetrix data) into **dbVOR** and illustrate the process with an example HapMap GWAS data set in Illumina format. We introduce two new processing steps to define samples and to define blocks of markers for GWAS data. Next, we show an alternative mechanism for loading Affymetrix data. The essential steps in this work flow are:

1. Add member data
2. Add sample data
3. Add marker data

[27] The measurements were performed on an iMac with a 3.33Ghz Intel Core 2 Duo processor having 8 GB memory and running OS X 10.6.8.

4. Add genome-wide genotype data

Second genome-wide work flow: For PLINK binary (bed) format data files.

We show how to load PLINK data into the GWAS **dbVOR** representation in Section 2.7.6. PLINK `http://pngu.mgh.harvard.edu/~purcell/plink/` is a very popular program for filtering, analyzing, and processing large scale genomic data. It supports several data formats; of note, it has a binary format that provides a very succinct way to store and share genotype data. We support the PLINK binary format only. PLINK can easily convert any of its other data formats to binary format. The essential steps in the PLINK work flow are:

1. Add member, sample, trait data
2. Add marker data
3. Add genome-wide genotype data

Data extraction: After the genome-wide data have been successfully read into the database, it can then be extracted into a variety of formats using the **genout** command as described above in Section 2.6.

Limitations: Note that while when entering small-scale targeted data we have options for merging new genotype data into the database while comparing the new data to the already existing data (see Section 2.5.6). With genome-wide scale data, such operations are not currently available. Furthermore, currently you can only load the genome-wide data for a particular sample/experiment combination once - subsequent attempts to load what appears to be duplicate genome-wide data for the same sample from the same experiment will be ignored. Soon we hope to revise **dbVOR** to relax these restrictions and enhance handling of genome-wide scale data.

2.7.1 Genome-wide HapMap Example

Not only is genome-wide data voluminous but in our case the test Affymetrix data set we were using internally contains real patient data and its release is controlled by HIPPA and hence not publicly available. Fortunately, Faheem Mitha, the author of the SNPpy program[28] found such a publicly available data set of Illumina data. These data are made

[28] Mitha F, Herodotou H, Borisov N, Jiang C, Yoder J, et al. (2011) SNPpy - Database Management for SNP Data from Genome Wide Association Studies. PLoS ONE 6(10): e24982. doi:10.1371/journal.pone.0024982

available from the ftp.ncbi.nih.gov site and correspond to Gene Expression Omnibus accession numbers: GSE17205 (CEU), GSE17206 (CHB+JPT), and GSE17207 (YRI). We give instructions below how to download these data. The subsequent sections show how to manipulate these data. If you have your own data, skip to Section 2.7.2.

2.7.1.1 Family Data

SNPpy also has a set of phenotypes that we have copied to our tutorial Data/ directory: ceupheno.csv, chbjptpheno.csv and yripheno.csv. We have been granted permissions from Faheem Mitha, its author, to use these files for dbVOR. Each file looks like a PLINK .fam file with an additional 'race' column.

2.7.1.2 Plate Data

An annotation file, Human610-Quadv1_B.csv, is available from Illumina by typing (on one line):

```
wget -c --ftp-user=guest --ftp-password=illumina ftp://ftp.illumina.com//Whole%20Genome%20Genotyping%20Files/
Archived_Human_Products/Human610-Quad_v1_product_files/Human610-Quadv1_B%20files/Human610-Quadv1_B.csv
```

(**wget** is a generally available utility for fetching web pages. All the text after **wget** should be on a single line.)

2.7.1.3 Hapmap Data

To fetch GSE17205, GSE17206 and GSE17207, first create a new directory and type:

```
wget -c ftp://ftp.ncbi.nih.gov/pub/geo/DATA/supplementary/series/GSE17205/GSE17205_Human610-Quadv1_CEU_Final_Call_Report.csv.
wget -c ftp://ftp.ncbi.nih.gov/pub/geo/DATA/supplementary/series/GSE17206/GSE17206_Human610-Quadv1_CHB+JPT_Final_Call_Report.
wget -c ftp://ftp.ncbi.nih.gov/pub/geo/DATA/supplementary/series/GSE17207/GSE17207_Human610-Quadv1_YRI_Final_Call_Report.csv.
```

2.7.1.3.1 checksum and size

You should calculate these md5 checksums for the files:

```
9654f79def058020b7e2c41ce8e891a1 GSE17205_Human610-Quadv1_CEU_Final_Call_Report.csv.gz
6846fcce6fb8940e840121c7d26e76e4 GSE17206_Human610-Quadv1_CHB+JPT_Final_Call_Report.csv.gz
a97ab3f7b043de603603b9faba76a260 GSE17207_Human610-Quadv1_YRI_Final_Call_Report.csv.gz
```

The compressed file sizes are almost 1 Gigabyte and listed below:

```
967509110 GSE17205_Human610-Quadv1_CEU_Final_Call_Report.csv.gz
992630807 GSE17206_Human610-Quadv1_CHB+JPT_Final_Call_Report.csv.gz
1022864215 GSE17207_Human610-Quadv1_YRI_Final_Call_Report.csv.gz
```

2.7.1.3.2 unzip

These files need to be "unzip"ed to be subsequently processed. So we will type:

```
gunzip *.gz
```

These resulting uncompressed files have a format that is reminiscent of PLINK lgen format with additional frequency and scoring information on each line. The header reads:

```
SNP Name,Sample ID,Allele1 - Forward,Allele2 - Forward,GC Score,X,Y,X Raw,Y Raw,Log R Ratio,B Allele Freq
```

2.7.1.3.3 Summary

In addition to providing the **Family Data** (*pheno.csv) files, we also supply the dbVOR configuration files which we will describe below. In these latter files, we will call the database **hapmap**, the user will be **watson** with password **crick**. The project will be "**project**", the technology will be **illumina** and the experiments will be **CEU**, **YRI** and **CHBJPT**.

2.7.2 Genome-wide Prerequisites

You may have a database that contains only genome-wide GWAS data or it may contain both simple targeted marker data and genome-wide GWAS data. In both cases, you may need to add the new samples to the table that describes the pedigree members (Section 2.4.1). You may also want to specify the traits for any new individuals (Section 2.4.2).

2.7.2.1 Create a database

If you have not already done so, before attempting to enter data you must create a MySQL database. To do so, following the instructions in Section 2.3 issue a command similar to this:

dbvor createdb --db=*exampledb* --host=*localhost* --user=*example* --root=*root* --sqlpath=<SQLPATH>

where <SQLPATH> is replaced by the complete path to the directory containing the SQL table definitions (See Section 1.4.4 for details). This is the path to the **sql** directory of the **dbvor.100** folder.

2.7.2.2 Add Members for the HapMap Example

We will need to load the new members. (We will illustrate this and later concepts for CEU, but the file formats and hence most of the data in the .cfg files will be the same for each data set.) The ceupheno.csv file is a standard .fam file with an extra column. (The yripheno.csv and chbjptpheno.csv have the same format as ceupheno.csv.) We need the pedigree, person and sex columns. We would like the father and mother but as we do not understand the semantics of the values used, we will ignore them. To setup a .cfg file, we supply:

**--project**	project name
**--sep**	field separator character
**--dir**	directory of the **--input** file
**--input**	the input file, *ceupheno.csv*
**--pedigree**	the pedigree column
**--person**	the person column
**--gender**	the gender column

The ***ceuMembers.cfg*** file should look like:

```
project:    GARM
sep:        ,
dir:        .
input:      ceupheno.csv
pedigree:   1
person:     2
gender:     5
```

Specifying a separator, **sep**, is necessary because the file does not use white space between values. The file ceuMembers.cfg is supplied; so we type:

```
dbvor Member ceuMembers.cfg -v --commit
```

Note that **dbVOR** subcommands are not case-sensitive, so we could have typed 'member' instead of 'Member'.

The related command should be repeated for the other data sets:

```
dbvor Member yriMembers.cfg -v --commit
dbvor Member chbjptMembers.cfg -v --commit
```

Typically after experiments are established, the members do not change so we do not expect to run this command again. (Of course, we can if we need to add new subjects.)

Note: ceuMembers.cfg references the ceupheno.csv file, while the yriMembers.cfg file references yripheno.csv, etc.

We will not add any phenotypes for these members.

2.7.3 Genome-wide Samples

GWAS data sets typically contain more elaborate names for samples that include a file name prefix, date, and retry identifier. In addition, for quality control it is desirable to record the plate position, well information, and other details describing the sample. The **dbvor** subcommand, **sample**. loads these data viz.:

dbvor sample *dbvorSample.cfg* --commit -v

The configuration file needs to supply:

**--project**	project name
**--technology**	technology used
**--experiment**	experiment name
**--dir**	directory of the **--input** file
**--sep**	input field separator if necessary
**--input**	the input file, *ceuSamples.txt*

The ***ceuSamples.cfg*** file might look like:

```
project:     project
technology:  illumina
experiment:  CEU
dir:         .
sep:         ,
input:       ceuSamples.txt
```

The **--input** file has a header line and then rows describing each sample. The columns are specified in a fixed order as follows:

column name	column	interpretation
subject_id	1	simple name for sample
active_value	2	active flag value for data records generated for this sample
pedigree	3	matching pedigree value for the sample
person	4	matching person value for sample
plate	5	plate identifier for sample
well	6	well identifier for sample on plate
misc	7	additional information about the sample
sample_id	8	file name that contains the lab sample

The most important information in the above mapping is the *sample_id* and the *pedigree/person* information. These data are used to connect the sample to the person pedigree information from Section 2.4.1. The *active_value* indicates the confidence or trust in this sample. For example, if some people were genotyped in multiple wells or multiple plates to serve as a control, you might choose to set the *active_value* on some of the duplicates to 0 to ignore them. The columns: *subject_id*, *plate*, *well*, and *misc* are not processed by dbVOR and are available to record information about the sample. (Note: It is suggested that the "column names" (above) be used as the header line of the file, but the header line is not processed.)

2.7.3.1 Create Samples for the HapMap Example

Although dbVOR requires a description of the samples being analyzed, we do not have this information available. So we will leave the plate, well, and misc fields empty. In addition, we do not have a true subject id or sample id, but we do observe that the person field is

unique. So we will use the person field repeatedly. The person and pedigree are important to link the sample to the subject. The sample_id is the lab name of the sample that will be found in the data received from the lab. The subject_id is a simple familiar name of the person that would be more important if the person field were not unique. There can be multiple samples for some subjects. We will make a simulated sample file by taking lines in the ceupheno.csv file, for example:

```
4,NA18500,3,2,1,0,YRI
```

and generating a corresponding entry for the sample file, ceusamples.csv:

```
NA18500,1,4,NA18500„„NA18500
```

Thus with the fields list in order as:

```
subject_id,active_value,pedigree,person,plate,well,misc,sample_id
```

We have specified that SUBJECT_ID, PERSON and SAMPLE_ID are NA18500, that ACTIVE_VALUE is 1 and PEDIGREE is 4 and the other fields are empty.

2.7.3.2 Add Samples for the HapMap Example

The ceuSamples.cfg file specifies where the input samples file is and that it is in "csv" format. The samples are loaded by typing:

```
dbvor Sample ceuSamples.cfg -v --commit
```

The related command should be repeated for the other data sets:

```
dbvor Sample yriSamples.cfg -v --commit
dbvor Sample chbjptSamples.cfg -v --commit
```

2.7.4 genoi: Simple genome-wide data (Illumina)

We are not intimating that Illumina Processing is simple, rather its data appear to be presented in a format that is simple to process, i.e. the data format is simple. Loading the GWAS data into the database is a two step process. First, the markers must be identified and then the data need to be specified. For GWAS data, **dbVOR** groups markers into blocks by their chromosome and position and blocks are presented and fetched to/from the database. The marker information is used to build the blocks and consequently to take the blocks apart. Reducing the number of items the database must track is what improves the GWAS performance of **dbVOR**.

The program, **genoi**, handles both steps for Illumina/simple data. The flag, **--make-snps**, must be set to select the first step, while it must be absent on the second step.

2.7.4.1 Simple GWAS Markers

As a prerequisite for this step, we will need the plate assay id, chromosome number, chromosome position and the marker id. All this data may be available from the vendor. Often, though, the marker id is missing and the probe flanking sequences are specified. In this case, a tool like BLAST can be used to find the marker. Alternatively, using the chromosome and position, a search through NCBI markers will allow a matching marker id to be found. Henceforth, we will assume all the requisite data has been acquired.

The program **genoi** (further described in Section 3.4.2) to load "simple" GWAS data is similar in name and spirit to the **geno** program that loads genotype data:

genoi ***ceuMarkers.cfg*** **--commit -v**

The configuration file needs to supply:

**--project**	project name
**--technology**	technology used
**--experiment**	experiment name
**--make-snps**	required!!
**--snp_db_output**	True
**--snp_file_output**	file name to store records into for debugging (optional)
**--build**	build id to use in marker_info table for data
**--dir**	directory of the **--ilmnmap** file
**--type**	***ilmn***
**--ilmnmap**	the input file
**--sep**	input file field separator
**--name**	plate assay name column number
**--chr**	marker chromosome number column number
**--position**	chromosome position column number
**--allele1**	SNP allele column number
**--allele2**	other SNP allele column number
**--alleles**	start column number if alleles are contiguous vs above
**--snp**	marker name if different from **--name**

Some explanation of these arguments is necessary. First, **--make-snps** must be specified without any value to execute this processing step. Second, the "**--type: ilmn**" and the **--ilmnmap** keywords indicate the legacy of this code; which is now more general than handling this specific vendor. Third, the argument, **--snp_db_output**, must be set to true (as well as **--commit** must be set) to store the marker data in the database. (The **--snp_file_output** argument is optional and currently is only used for debugging; it will store the marker data in the specified file.) Fourth, the **--build** argument is also optional and requests that the marker position information found in the **--ilmnmap** file, be copied into the database *Marker_info (Section 2.4.5)* table associating it with the selected build name. Finally, if the marker id and the assay name are the same, it is not necessary to specify the former.

2.7.4.1.1 Add Markers for the HapMap Example

The markers on the plate are described in the Human610-Quadv1_B.csv file. *But, there are extra data in the Human610-Quadv1_B.csv file that Mega2 can not process. In the current file, there are 9 lines before the header line that must be removed; in addition there*

are 23 lines starting with a line '>"–control–" at the end of the file that are spurious and also must be removed. You should create a new file without these extra data and name it Human610-Quadv1_BB.csv.

The header of this new file contains:

```
IlmnID,Name,IlmnStrand,SNP,AddressA_ID,AlleleA_ProbeSeq,AddressB_ID,AlleleB_ProbeSeq,
GenomeBuild,Chr,MapInfo,Ploidy,Species,Source,SourceVersion,SourceStrand,SourceSeq,
TopGenomicSeq,BeadSetID,Intensity_Only,Exp_Clusters,CNV_Probe
```

The correspondence is NAME for **name** , (column 2), the CHR for **chromosome** [column 10], the MAPINFO for **position** (column 11) and the SNP for **alleles** (column 4). We enter these keyword-values into ceuMarkers.cfg, and indicate the file is "csv" format. In examining the file, we observed that the mapping information came from build 36; thus we add an argument of **build** with the value GB36.

The ***ceuMarkers.cfg*** file might look like:

```
project:       project
technology:    illumina
experiment:    CEU
make_snp:
snp_db_output: True
build:         GB36
dir:           .
type:          ilmn
ilmnmap:       Human610-Quadv1_BB.csv
sep:           ,
name:          2
chr:           10
position:      11
alleles:       4
```

Now we can type:

```
genoi ceuMarkers.cfg -v --commit
```

We run this only once, not for each data set, because it describes the plate technology which is the same across all three data sets.

After this step has been performed, it is worth reviewing section 2.4.5 on the various ways to specify the latest marker positions information to the database. As discussed in relation to the **--build** argument, we can load the marker position information into the traditional *marker_info* table. But it is rare to have the average, male and female genetic positions available. Thus it is important to obtain this information via the mechanisms introduced earlier before fetching data from the database.

2.7.4.2 Simple GWAS Data

The **genoi** program also loads the genotype data using a few different arguments than we have just seen:

genoi *ExomeData.cfg* --commit -v

The configuration file needs to supply:

**--project**	project name
**--technology**	technology used
**--experiment**	experiment name
**--snp_db_input**	required (read markers from database)
**--db_output**	required (write blocks to database)
**--dir**	directory for everything but the input file
**--type**	***ilmn***
**--inputdir**	the directory for the **--input** file
**--input**	the input file (pattern)
**--multi**	indicates that there are multiple samples in the input file
**--sep**	input file field separator
**--name**	plate assay name column number
**--sample**	sample name column number
**--call**	call quality value for SNPs column number
**--allele1**	SNP allele column number
**--allele2**	other SNP allele column number

Some more explanation: The **--dir** argument specifies the directory for the log (and a long deprecated **--snp_file_input**), while **--inputdir** indicates where the GWAS data files are located. In our Example case, all the data are in one file. This is indicated by supplying the argument **--multi** with no arguments. In this case the value in the **sample** column must exactly match a SAMPLE_ID in the samples file (section 2.7.3.1).

Alternatively, there can be multiple files with a single sample in each data file. We allow the standard Unix shell file name expansion to be used indicated by embedding "*" and/or "?" in the file name. Note: Samples may not span multiple files.) Finally, the **--sample** column in the data file is first checked against input file name. It must match at the end of the input file name just before the suffix (viz. .csv, .txt, .gz, ...), i.e. sample 123_456 would be expected to be found in file "mydata_123_456.csv". The full file name "mydata_123_456.csv" is used to look up the matching pedigree/person in the *Sample* database table (Section 2.7.3).

2.7.4.2.1 Add Data for the HapMap Example

As we previously looked at the annotations file to decide where the fields that we needed were, we now look at the data file header:

```
SNP Name,Sample ID,Allele1 - Forward,Allele2 - Forward,GC Score,X,Y,X Raw,Y Raw,Log R Ratio,B Allele Freq
```

For the configuration file, we need the NAME for **name** (column 1), the SAMPLE for **sample** (column 2), ALLELE1 - FORWARD for **allele1** (column 3), ALLELE2 - FORWARD] for **allele2** (column 4) and GC SCORE for **call** (column 5). We also need some boiler plate to indicate the "**inputdir**" (.), the field separator [**sep**] (","), the data file name (**input**) and an indicator that there are multiple samples in the input file (**multi**). In addition, we indicate that the markers come from the database and that the results are stored in the database: **snp_db_input** and **db_output**.

The ***ceuData.cfg*** file might look like:

```
project:      project
technology:   illumina
experiment:   CEU
snp_db_input:
db_output:
dir:          .
```

```
type:       ilmn
sep:        ,
inputdir:   .
multi:
input:      GSE17205_Human610-Quadv1_CEU_Final_Call_Report.csv
name:       1
sample:     2
call:       5
allele1:    3
allele2:    4
```

with it you can type:

```
genoi ceuData.cfg -v --commit
```

The analogous commands:

```
genoi yriData.cfg -v --commit
genoi chbjptData.cfg -v --commit
```

should be used to load the other data sets; the yriData.cfg and chbjptData.cfg files are provided.

After the genome-wide data have been successfully read into the database, it can then be extracted into a variety of formats using the **genout** command as described above in Section 2.6.

2.7.5 genoa: Genome-wide Data (Affymetrix)

We have received Affymetrix GWAS data with multiple samples per data file; the samples span a number of contiguous columns. A decision was eventually made that the best way to process this data was to break the file into separate files, one for each sample. This makes a number of processes easier, such as data cleaning and reduces the problem of loading Affymetrix data to the problem of loading "simple" data described in the previous section. But there was the old way of processing Affymetrix data, which we describe here. While this code works, we recommend breaking the data into separate files and using the approaches above to process the files.

2.7.5.1 GWAS Samples Revisited – Affymetrix samples

Previously, we had said that the *sample_id* presented to the **dbvor sample** subprogram was the sample file name. This is no longer possible since a data file can contain multiple samples. Thus, the sample_id is just the sample's identifier. The latter is best defined by looking at the contents of the data file. Unfortunately, this can not be explained clearly until a future Section 2.7.5.3.

2.7.5.2 Affymetrix GWAS Markers

The program **genoa** to load "simple" GWAS data is similar in name and spirit to the **geno** and **genoi** programs that load genotype data and GWAS data, respectively:

genoa *ExomeMarker.cfg* --commit -v

The configuration file needs to supply:

**--project**	project name
**--technology**	technology used
**--experiment**	experiment name
**--dir**	directory of the **--affymap** file
**--type**	***affy***
**--affymap**	the input file
**--sep**	input file field separator
**--make-snps**	required!!
**--build**	build id to use in marker_info table for data
**--snp_file_output**	file name to store records into for debugging
**--snp_db_output**	True

The explanation of these arguments is very similar to those used by **genoi**. First, **--make-snps** must be specified without any value to execute this processing step. Second, the "**--type: affy**" and the **--affymap** keywords indicate Affymetrix processing. For this case, **genoa** knows the layout of the **--affymap** file: where the marker and assay names and positions are. Third, the argument, **--snp_db_output**, must be set to true (as well as **--commit** must be set) to store the marker data in the database. (The **--snp_file_output** argument is optional and currently is only used for debugging; it will store the marker data in the specified file.) Fourth, the **--build** argument is also optional and requests that the marker

position information found in the **--affymap** file, be copied into the database *Marker_info (Section 2.4.5)* table associating it with the selected build name. For more details about **genoa**'s options, see Section 3.4.4.

The ***ExomeMarker.cfg*** file might look like:

```
project:        GARM
technology:     Affymetrix
experiment:     Data1Oct2013
dir:            .
type:           affy
affymap:        affy.Xba131.txt
make_snp:
snp_db_output:  True
build:          Xba131
```

After this step has been performed, it is worth reviewing section 2.4.5 on the various ways to include the latest marker position information in the database. As discussed in relation to the **--build** argument, it is possible to load the marker position information used during the first processing step into the traditional *marker_info* table. But it is rare for that information to have the average, male and female genetic positions for the marker. Thus, it is important to obtain this information via the mechanisms introduced earlier before fetching data from the database.

An alternative exists to using **dbvor MissingMarkerInfo** to obtain a list of the markers. You may supply two additional (though deprecated) arguments:

**--make_rs_file**	required!!
**--rs_file_output**	file name to store list of markers in

The file will contain a list of all the markers seen in the input file, not just the ones that are "unknown" to the *Marker_info* table for a selected build.

2.7.5.3 Affymetrix GWAS Data

The **genoa** program, described in more detail in Section 3.4.4, also loads the genotype data using a few (more) different arguments than we have seen previously:

genoa *ExomeData.cfg* --commit -v

The configuration file needs to supply:

**--project**	project name
**--technology**	technology used
**--experiment**	experiment name
**--dir**	directory for everything but the input file
**--type**	***affy***
**--inputdir**	the directory for the **--input** file
**--input**	the input file (pattern)
**--extra_lines**	additional lines of header in addition to the column header line
**--file_separator**	the input file column separator
**--nocachefile**	do not cache the input file in memory even if it has multiple data columns
**--snp_db_input**	required (indicates markers are read from the database)
**--db_output**	required (indicates results are written to the database)
**--call_sfx**	suffix of column header that contains the called alleles
**--qual_sfx**	suffix of column header that contains the call quality
**--extra_hdr**	column headers that should be ignored during processing

Some more explanation; but this is slightly different than for the "simple" GWAS because we know the file format: The **--dir** argument specifies the directory for the log (and a long deprecated **--snp_file_input**), while **--inputdir** indicates where the GWAS data files are located. Although each file contains multiple samples, it is still common to find a multiple data files to keep the file size manageable. So, as before, rather than list many input files, we allow the standard Unix shell file name expansion to be used indicated by embedding "*" and/or "?" in the file name. Note: Samples do not span multiple files. Since there are multiple samples on each line of the file, multiple scans through the file are necessary. If the number of markers is small, the system can cache all the lines so as to not have to re-read and re-parse the file to get the next sample. **--nocachefile** turns this feature off for large files. And as noted earlier, a better solution is to split each Affymetrix data file into one file per sample. **--extra_lines** accounts for additional lines of commentary at the start of the data file; the file will always have a header that identifies what data is in each column.

In each file, there will be a group of columns for each sample. The interesting columns for us have headers that end with **--call_sfx** (for alleles) and **--quality_sfx** (for call quality [Note: We store but do not currently process call quality.]). The prefix of the column header indicates the sample id. Occasionally additional columns may appear in the data that we ignore; these columns are listed in the **--extra_hdr** argument. (Values may be separated by "," or the argument/value may be repeated.) Consider the column head "123_011013.chp Call". This would be identified as containing the allele data for sample_id 123_011013 (for the marker corresponding to the each row). The sample_id might be the concatenation of some sample information and a run date. In any case, the entire prefix, 123_011013 is the *sample_id* looked up in the *Sample* table to get the corresponding pedigree/person. (Section 2.7.5.1).

The ***ExomeData.cfg*** file might look like:

```
project:        GARM
technology:     Affymetrix
experiment:     Data1Oct2013
dir:            .
type:           affy
sep:            "     "
inputdir:       somewhere_else
input:          report*.txt
call_sfx:       .chp Call
qual_sfx:       .chp Confidence
snp_db_input:
db_output:
no_cache_file:
```

After the genome-wide data have been successfully read into the database, it can then be extracted into a variety of formats using the **genout** command as described above in Section 2.6.

2.7.6 genok: PLINK binary (bed) data files

The PLINK binary data representation (`http://pngu.mgh.harvard.edu/~purcell/plink/data.shtml#bed`) is composed of three files: a "**fam**" file (with the suffix *.fam*) which defines the pedigree (family, person, father, mother, gender, trait/case/control), a "**bim**" file

(with the suffix *.bim*) which describes the markers (name, positions, alleles) and the "**bed**" file (with the suffix (*.bed*) which contains the genotype data in a compressed format. (Technically, there are two different internal organizations to the **bed** file - since we support both, we will subsequently ignore this detail.)

As we did in the Illumina example (Section 2.3), we need to define the members and samples from the PLINK information. Unfortunately, in the PLINK data representation, there is no place to store the plate, the well, and other miscellaneous data. Rather than manually creating an *ad hoc* sample file (See Create Samples for Example [Section 2.7.3.1]), we will automatically generate sample data from the **fam** file. We also gather trait information from this file.

2.7.6.1 Processing Family Data using dbvor plink

The PLINK *.fam* file can contain the family, person, father, mother, gender, and a final column that can be a trait or a case/control indicator. All the fields do not have to be present but they appear in the order shown when they exist. The **dbvor** subprogram **plink** will extract all the data from the *.fam* file to populate the member table (2.4.1), the sample table (2.7.3.1) and even a trait column (2.4.2)

To be concrete we will assume the three PLINK files are named: *myplink.fam*, *myplink.bim* and *myplink.bed*. So let us type on the command line:

dbvor plink *Members.cfg* --commit -v

Members.cfg is an example configuration file. For **dbvor plink**, we need to specify the pedigree information:

**--project**	project name
**--technology**	technology used
**--experiment**	experiment name
**--dir**	directory for inputs and outputs
**--input**	the input file, *myplink.fam*
**--pedigree**	the column number of the pedigree field
**--person**	the column number of the person field
**--father**	the column number of the father field
**--mother**	the column number of the mother field
**--gender**	the column number of the gender field
**--trait**	the column number of the trait field
**--active**	active value to store with member data (default 1)
**--noheader**	should be 1 (True) since *.fam* files do not have headers

If the **parents**, **gender**, or **trait** are not known/available, the corresponding fields should not be provided.

There are a few more arguments available to describe a trait field:

**--missing_phenotype**	a trait value that indicates the trait is missing (default -9 or 0)
**--default_trait_type**	type to use for the trait column (default 'A' (for affection status); use 'T' for quantitative values)
**--default_trait_name**	name to use for the trait column (default 'PLINKTrait')
**--stat**	generate statistics for the trait: values and counts
**--nostat**	do not generate statistics for the trait

We label the first column of the input file: column 1 and subsequent columns are assigned consecutive integer values. Thus a ***Members.cfg*** file might look like:

```
project:      project
technology:  myplinktech
experiment:   myplinkexp
dir:          .
input:     myplink.fam
```

```
log:        myplink.Members%.log.txt
pedigree:   1
person:     2
father:     3
mother:     4
gender:     5
trait:      6
noheader:   true
```

The **plink** subprogram of **dbvor** runs several internal subprograms to populate the Members table (**dbvor member**), the Samples table (**dbvor famsample**), and the Traits table (**dbvor famtrait**). Each of these three programs generates the standard **dbvor** program output on the console and in log files. It is very possible that each of the three log files will have the same name and hence overwrite each other. This problem is mitigated by adding a trailing .M, .S, or .T, respectively, after the time stamp in the log name. If the log name is user specified, a ”%” in the name will be replaced by the indicator character as each subprogram is run.

2.7.6.2 PLINK genome-wide Markers

The PLINK *.bim* file contains the name, chromosome, position and two alleles for each marker. (Note: the PLINK binary representation can only contain biallelic markers.) The field layout is specified by PLINK and is fixed. All the flexibility that was necessary for Illumina markers (Section 2.7.4.1) is unnecessary for *.bim* files.

The program **genok**, described in more detail in Section 3.4.3, to load PLINK GWAS marker data is similar in name and spirit to the **genoi** program that loads Illumina GWAS markers/data:

genok ***Markers.cfg*** **--commit -v**

The configuration file needs to supply:

**--project**	project name
**--technology**	technology used
**--experiment**	experiment name
**--make-snps**	required!!
**--snp_db_output**	True
**--snp_file_output**	file name to store records into for debugging (optional)
**--build**	build id to use in marker_info table for data
**--dir**	the working directory for most input and all output
**--inputdir**	the directory containing the input *.bim* file
**--plinkbim**	the name of the input *.bim* file

Notes: First, **--make-snps** must be specified without any value to execute this processing step. Second, the argument, **--snp_db_output**, must be set to true (as well as **--commit** must be set) to store the marker data in the database. (The **--snp_file_output** argument is optional and currently is only used for debugging; it will store the marker data in the specified file.) Third, the **--build** argument is also optional and requests that the marker position information found in the *.bim* file, be copied into the database *Marker_ info (Section 2.4.5)* table associating it with the selected build name.

A concrete example of a configuration file is shown below:

```
project:        project
technology:  myplinktech
experiment:   myplinkexp
make_snps:
snp_db_output:  True
build:          myplink
dir:            .
inputdir:       .
plinkbim:       myplink.bim
```

2.7.6.3 PLINK bed genome-wide genotype data

The **genok** program also loads the genotype data using a few additional arguments:

genok ***Data.cfg*** **--commit -v**

The configuration file needs to supply:

**--project**	project name
**--technology**	technology used
**--experiment**	experiment name
**--snp_db_input**	required (read markers from database)
**--db_output**	required (write blocks to database)
**--dir**	directory for everything but the input files
**--inputdir**	the directory for the PLINK BED input files
**--plinkbed**	the input *.bed* file
**--plinkfam**	the input *.fam* file
**--plinkbim**	the input *.bim* file

Notes: The **--dir** argument specifies the directory for the log, while **--inputdir** indicates where the PLINK *.bed* data file and other PLINK files are located. The *.bim* and *.fam* files are necessary to properly index the *.bed* file. They specify the row and column labels, respectively, of the *.bed* file (or possibly the transpose).

The example of a configuration file, shown below, is rather similar to that just given above:

```
project:       project
technology:  myplinktech
experiment:   myplinkexp
snp_db_input:
db_output:
dir:           .
inputdir:      .
plinkbed:      myplink.bed
plinkfam:      myplink.fam
plinkbim:      myplink.bim
```

After the genome-wide data have been successfully read into the database, it can then be extracted into a variety of formats using the **genout** command as described above in Section 2.6.

2.7.6.4 Adding additional genome-wide data

After using **genok** to import genome-wide PLINK data, one may wish to add additional genome-wide data for the same individuals. This may require several steps:

1. Add another list of samples (which are specific to each genotyping experiment). The easiest way to do this would be to run **dbvor plinksample** using the original PLINK fam file as input while supplying new **--experiment** and **--technology** names. See Section 3.3.15 for more details.

2. If the set of markers differs from the initial set, then you will need to use **genok** to add in the new markers, using the approach described above in Section 2.7.6.2..

3. Add in the new genotypes using **genok**, using the approach described above in Section 2.7.6.3.

2.7.7 Deleting and reentering genome-wide data

The data entered for a single sample or a member may be incomplete or erroneous and need to be removed and reentered. On a larger scale, all the data for an experiment might need to be reentered and so might the marker data. The **dbvor** program with the options: **DeleteBlockSample, DeleteBlockMember, DeleteBlockAllSamples,** and **DeleteSnpBlockMap** will handle these cases (Note that the 'Block' term in the names of these options indicates these commands are applicable to genome-wide data stored using the genotype blocks approach - e.g., as entered by **genoi**, **genoa**, or **genok**). In addition, the option **ShowSample** displays and/or lists all of samples or specifically requested samples from the ***samples*** tables - this is a good way to get an overview of what genome-wide data has been entered for each sample.

2.7.7.1 dbvor DeleteBlockSample

The **dbvor DeleteBlockSample** deletes the genotype data for a list of samples. Each **sample** must have the indicated **experiment** and **technology**.

dbvor *DeleteBlockSample dbvor.cfg* --commit -v

The configuration file needs to supply:

**--project**	project name
**--technology**	technology used
**--experiment**	experiment name
**--sample**	a list of samples names to delete

Notes: Only one sample for each **--sample** and **--experiment** is deleted.

2.7.7.2 dbvor DeleteBlockMember

The **dbvor DeleteBlockMember** deletes the genotype data for a list of members. Each **member** must have the indicated **experiment** and **technology**.

dbvor ***DeleteBlockMember dbvor.cfg*** **--commit -v**

The configuration file needs to supply:

**--project**	project name
**--technology**	technology used
**--experiment**	experiment name
**--member**	a list of member names to delete

Notes: All samples for each **--member** and **--experiment** is deleted. There could be a main sample and a control sample, etc from the specified experiment mapping to a single member.

2.7.7.3 dbvor DeleteBlockAllSamples

The **dbvor DeleteBlockAllSamples** deletes all the genotype data for all samples and members of the indicated **experiment** and **technology**.

dbvor ***DeleteBlockAllSamples dbvor.cfg*** **--commit -v**

The configuration file needs to supply:

**--project**	project name
**--technology**	technology used
**--experiment**	experiment name

2.7.7.4 dbvor DeleteSnpBlockMap

The **dbvor DeleteSnpBlockMap** deletes all the marker data for the indicated **technology**.

dbvor ***DeleteSnpBlockMap dbvor.cfg*** **--commit -v**

The configuration file needs to supply:

**--project**	project name
**--technology**	technology used

Note: The genotype data for the experiments in this **technology** will be in the database, but will not be useable until the marker data is reloaded with a **--make-snps** option of a genome-wide data entry command.

2.7.7.5 dbvor ShowSamples

The **dbvor ShowSamples** lists all of samples or specifically requested samples from the ***samples*** table. This data includes the file name that the sample came from which might not be know when the table was originally created.

dbvor *ShowSamples dbvor.cfg* --commit -v

The configuration file needs to supply:

**--project**	project name
**--experiment**	experiment name, to be used whenever **--sample** is specified
**--sample**	a list of samples to display
**--technology**	the technology name, to be used whenever **--sample** is specified
**--output**	prefix for the output file name

If **--output** is specified, a unique named output file is generated for each experiment found in the ***samples*** table. Each file contains the sample entries for the particular experiment. The file name is <stem>.<experiment>.txt. <stem> comes from the **--output** argument while <experiment> comes from the *experiment* column in the ***samples*** table.

If no sample items are specified, then the entire sample table is displayed.

Chapter 3

Manual

3.1 Introduction

This chapter is focused on documenting each command in detail. To learn how to use the commands, please see the detailed tutorial in Chapter 2.

The first section gives the syntax for arguments and lists the required arguments and then the commonly occurring arguments. The next section describes all the "subprograms" of the **dbvor** program. These programs are used to enter the data into all the database tables except for the genotype data. The subsequent section shows the programs that enter genotype data into the database. That section is followed by the program that extracts data from the database. The final section covers a few programs missing from the previous discussion.

Below, we use a typographic convention for describing **dbvor** commands. Arguments (and their descriptions) are presented in italic when they are optional. When the descriptions are in a conventional type face, a value is required. (Note: the argument name, itself, will be in ***bold italic*** and **bold**, respectively, for these two cases.) Some arguments indicate the column number of the input file that contains data, while other arguments provide data directly. This is distinction is emphasized by embedding the phrase **COLUMN NUMBER** in the argument description with the typeface shown for the column number argument values.

3.2 General Arguments

Arguments that appear on the command line are introduced with two "-" characters followed by the argument name. The value of the argument, if necessary, is either appended to the

argument name separated with an "=" or appears as the next argument.[1] Arguments may also be appear in a *Configuration File;* in which case, the,argument name is not preceded by "-" characters and it is separated from its value by a ":" character. Generally, argument values are strings, but when arguments are used to select columns from tabular data files, the argument is interpreted as an integer[2]. In all other cases, we will indicate the exact type of the argument at the start of its definition. The additional value types are:

**type**	**definition**
boolean	true or 1 is boolean true; false or 0 is boolean false. The case for the true/false does not matter; vis. True, TRUE, or TruE are the same.)
count	value is the integer count of the number of times the argument is present
float	value is a floating point number
int	value is an integer
present/true	when the argument is present, the value though unspecified is boolean true
present/false	when the argument is present, the value though unspecified is boolean false
list	indicates that the argument can appear multiple times and have multiple values.<br>It is in addition to the basic type.

dbVOR searches for a specially named *Configuration File* in your *home* directory, ***.dbvorrc***[3]. This file is processed before any other arguments are; it is a good place to store common, constant, and required arguments. ***.dbvorrc*** can contain the value for any argument; command line values are processed after all arguments from *Configuration Files* are processed.

Note: A *Configuration File* that contains a password (viz. the ***.dbvorrc*** file) should have permissions set to only allow the owner to read the file.

[1]Note: Some arguments do not need values and some arguments only have a single "-".

[2]By convention, we label the first column of the input file: column 1 and subsequent columns are assigned consecutive integer values

[3]On Unix and MacOS X, there is a long standing tradition that files that begin with "." are considered hidden. The normal file listing utility, **ls**, does not show these files by default.

3.2.1 Required Arguments

Every **dbVOR** program requires the following arguments. They point to data file(s) and indicate the database connection.

**--dir** (or) **--directory**	directory of **--input** file and also the directory used to store logs, etc
**--db**	data schema to hold MySQL database tables
**--host**	MySQL database server machine
**--password**	password for **--user**
**--project** (or) **-p**	project name. This field has been deprecated though not totally removed from the system. At present, it is best to set the value to PROJECT
**--user**	user name to authenticate database access

Note: Although it is not a required argument, **--commit** *(below) must be specified or no changes will be made in the database.*

3.2.2 Common Arguments

These arguments are generally available for all programs and have the specified affect but are not necessary. We will not repeat them in the later program descriptions.

**--commit**	(present/true) enters records into the database that are not already in the database. If any processing errors arise, the **--commit** operation is aborted.<br>**--commit** and **--compare** are mutually exclusive.<br>**--commit** may be supplemented with **--replace**, **--update**, or **--delete**.

--compare (present/true) checks whether the data from the **--input** file are already in the database. **--commit** and **--compare** are mutually exclusive.
Rational: We might have intended to entered some data and do not remember if we did. Someone else may have entered the data or some of the data. We may be curious whether the a particular data file was actually what was used to populate the database. Finally, we might have changed some fields (parentage or sex) for some people in the file, but are not sure if the changes were committed into the database.

--debug (or) **-d** (count) shows **dbVOR** internal operations for debugging. The data are determined by the program that is running. Information displayed will have database identifiers useful for debugging.

--delete (present/true) removes existing records from the database determined by the records in the **--input** file. **--delete** requires that **--commit** also be specified.

--genote (present/true) on a **--commit** operation, saves the *Configuration File* and *Log File* into the "note" table as described further in Section 3.6.1

--help (or) **-h** shows all possible arguments and a brief synopsis for the specified program

--log (or) **-l** specifies an explicit name for the **log** file. The default is <ConfigFile>.<date>.log.txt. The <date> has resolution to the second.

--pdb (or) **-z** (present/true) indicates that errors should not be "caught". This is used for debugging with the Python debugger. Most users will not have need for this feature.

--replace (present/true) enters records into the database**. --replace** removes (i.e. **--delete**s) matching old database records and creates new database records. This may violate database constraints and not be allowed. See **--update --replace** requires that **--commit** also be specified.

--rollback (present/true) inserts database entries just as **--commit**, but when all processing is complete the database is rolled back to remove all the changes. Think of this as a dry run.

--screen_always

(list) lists log printout sections that are not truncated on standard output. The default is to print the first 10 records from each section. This argument can appear multiple times with different section names (each section has the section name printed in its header). The special value, **all**, means that all sections will be printed in their entirety on standard out. Note: All sections are printed in their entirety in the log file.

--sep indicates the column separator for multi column input files. The default separator is any kind of white space. Comma separated files (.csv) should use "," for the separator. Tab separated files should use "<tab>" as the separator. Note: The <tab> character must be enclose in quotes (").
If the file is tab separated and there are empty values it is very important to indicate the separator is "<tab>" and not white space.

--source (list) specifies additional file(s) to be read for configuration data. This argument may be specified several times in a *Configuration File.*

--update (present/true) replaces existing records in the database in place unlike **--replace** which removes the old database records and creates a new database record. This is necessary if there are enabled database constraints that might be violated. **--update** requires that **--commit** also be specified.

--verbose (or) **-v** (count) shows additional details chosen by the program

3.3 dbVOR Commands

3.3.1 dbvor active

The **active** field value for a genotype is a reflection of the confidence of the accuracy of the measurement. The higher the **active** value, the more confident the measurement is. When there are multiple measurements for the same genotype (pedigree/person/marker), the measurements are grouped by their **active** value. Only the data with the highest **active** value are considered. (This processing is controlled by the flag **--filter_by_active** which is True by default.) If there is only one measurement, then there is no conflict in the measurements (by definition) even though there may be differences with the other less trusted measured values. If there are multiple different values at the highest **active** value, a process resolves the differences. Note: The default **active** value is 1. Note: A genotype with an **active** value of 0 is ignored even if it is only measurement of the genotype. (This processing is controlled by the flag **--use_inactive** which is False by default.)

Note: Many other tables populated by the programs below, store an --active value to indicate the confidence of the measurement. Currently the --active values are only used for genotype conflict resolution (described above).

3.3.1.1 NAME

dbvor active - sets the active flag for individual pedigree/person/marker entries specified in the input file for the given technology/experiment

dbvor active - sets the active flag for the given technology/experiment

3.3.1.2 SYNOPSIS

dbvor active *{ConfigFile.cfg and/or arguments}*

3.3.1.3 ARGUMENTS

--active (int) the **column number** of the active value. If there is no specified **--input** file name, this is the number to assign to the **--active** field.

--experiment name of the experiment

--input The input file is optional; if it is missing, the **--pedigree**, **--person**, and **--marker** may not be specified. In this case, the **--active** value is assigned to the **active** column of all genotypes for the specified **--experiment** and **--technology**.
If there is an the input file, each genotype for pedigree/person/marker specified by lines in the **--input** file is given the corresponding **--active** value. Only genotypes of the specified **--experiment** and **--technology** are affected.

--marker the COLUMN NUMBER of the marker name

--pedigree the COLUMN NUMBER of the pedigree field

--person the COLUMN NUMBER of the person field

--project name of the project

--technology name of the technology

--update (present/true) **--update** should be specified with **--commit** for this program. The reason for this is that the genotype records to be changed have already been loaded into the database by the **geno** program.

3.3.2 dbvor allelemap

The command, **dbvor allelemap**, loads allele mapping data. The mappings indicate a replacement allele value for a given value. Whenever the alleles for the specified markers in the specified experiments are fetched, the allele values will be transformed according to the mapping.

3.3.2.1 NAME

dbvor allelemap - loads the allele mapping data

3.3.2.2 SYNOPSIS

dbvor allelemap *{ConfigFile.cfg and/or arguments}*

3.3.2.3 ARGUMENTS

--active (int) **active** flag value to store in database for this entry

--experiments the COLUMN NUMBER of the experiment name field

--input the input file

--map (list) the COLUMN NUMBER of an allele mapping field. Repeat for as many mappings that appear on an input line. A mapping is represented as <given_allele>:<new_allele>. The character separating the two alleles is a ":"

--maptype (int)
1 -> indicates that the mapping applies to genotype data. (default)
2 -> indicates that the mapping applies to genome-wide data.
3 -> indicates that the mapping should be applied to the allele value after the genotype conflicts are resolved.

--marker the COLUMN NUMBER of the marker name field

Sample mapping row:

```
dataset3.txt    rs1073516    1:G        2:A
```

Once the data is loaded into the database, whenever the alleles for rs1073516 in experiment dataset3.txt are fetched, the allele values will be transformed: 1 is replaced by "G" and 2 by "A".

3.3.3 dbvor createdb

This program requires that a MySQL server already exist. It can create a new database for **dbVOR**'s use and it can create a database login and allow it to access a database. You can create as many databases as required and give more than one user access to any database. Finally, you can undo what **dbvor createdb** does.

Note: When a database is created, it is loaded with the table definitions necessary for **dbVOR** to operate.

Note: The program will have to be supplied with an identity and password with privileges to create databases, users, and to load databases. This is typically **root**. Note: The program will ask for necessary passwords as it runs. Passwords are never typed on the command line.

dbvor createdb will create a ***.dbvorrc*** file (Section 3.2) from its data, if the file does not already exist. The .***dbvorrc*** file is created with permissions that make it readable only by the file's owner so the password is NOT exposed to everyone. The owner is the person who is running the **dbvor createdb** program.

This program is the ONE exception to needing the list of required arguments in Section 3.2.1.

3.3.3.1 NAME

dbvor createdb - create a new data schema (and, if necessary, user) in an existing MySQL server

3.3.3.2 SYNOPSIS

dbvor createdb --db=*exampledb* --host=*localhost* --user=*example* --root=*root* --sqlpath=*<SQLPATH>*

3.3.3.3 ARGUMENTS

--db the database name

--enablenetworkaccess

(present/true) create a user login that allows the user to connect to the server from any machine on the internet. The default only allows access from a user on the server machine.

--host the database server machine name

--revoke *(present/true) disconnect access and then destroy the user and/or database. This operation is reference counted so that if the database is still in use or the user still accesses other databases, only the access permissions will be removed.*

--root the "root" name for the database server

--sqlpath the directory containing the SQL table definitions for the **dbVOR** database. Replace <SQLPATH> by the complete path to the directory containing the SQL table definitions (See Section 1.4.4 for more details).

--user the user name

3.3.3.4 USAGE

For examples of usage, please see Section 2.3.

3.3.4 dbvor create_experiment

This program is seldom needed now. The program that enters a file of data in the database will enter the filename as the experiment name into the database.

3.3.4.1 NAME

dbvor create_experiment - register the technology and experiment names in the database

3.3.4.2 SYNOPSIS

dbvor create_experiment *{ConfigFile.cfg and/or arguments}*

3.3.4.3 ARGUMENTS

**--commit**	(present/true) records the **experiment** name and current date in the database
**--experiment**	name of the experiment
**--technology**	name of the technology

3.3.5 dbvor delete_experiment

This program will first remove all the genotype data associated with the experiment name. Then it will remove the experiment name from the database.

3.3.5.1 NAME

dbvor delete_experiment - removes the original **experiment** data

3.3.5.2 SYNOPSIS

dbvor delete_experiment *{ConfigFile.cfg and/or arguments}*

3.3.5.3 ARGUMENTS

--commit (present/true) removes ALL the **experiment** genotype data records and the experiment name from the database

--experiment name of the experiment

--technology name of the technology

3.3.6 dbvor gmimarker

The **gmi.pl** program (`htttp://watson.hugen.pitt.edu/register/doc/gmi.html`) retrieves the genetic and physical positions for the markers specified from the latest Ensembl build. The output file (<name>_gmi_out_annot.txt) provides all the information needed by **dbvor marker_info** in a set order. This program internally invokes **dbvor marker_info** providing it the data in the **gmi.pl** output file.

3.3.6.1 NAME

dbvor gmimarker - load genetic position information into the database.

3.3.6.2 SYNOPSIS

dbvor gmimarker *{ConfigFile.cfg and/or arguments}*

3.3.6.3 ARGUMENTS

--active (int) **active** flag value to store in database for this entry

--build the genome build to which the positions and chromosome apply. All the marker information that is stored in the database indicates the build the information came from. It is possible to have data from multiple builds in the database at the same time and it may be necessary to select the desired build for output. If there is only one build in the database, we do not require you to specify the build to be used for generating output.

--input the input file. The input file is expected to be in the format produced by the **gmi.pl** utility program. If you have the same data in a different order, see **dbvor marker_info.**

3.3.7 dbvor gmimarkeralias

The **gmi.pl** program (`htttp://watson.hugen.pitt.edu/register/doc/gmi.html`) retrieves the genetic and physical positions for the markers specified from the latest Ensembl build. The log file (<name>_gmi_out_log.txt) includes a list of markers that were discovered to be aliased to other markers. **dbvor gmimarkeralias** reads the log file and adds the marker remapping to the database.

3.3.7.1 NAME

dbvor gmimarkeralias - processes output log of the **gmi.pl** program to find markers that are aliased and stores the remapping in the database.

3.3.7.2 SYNOPSIS

dbvor gmimarkeralias *{ConfigFile.cfg and/or arguments}*

3.3.7.3 ARGUMENTS

--input the input file. The input file is expected to be in the format produced by the **gmi.pl** utility program; it contains a section describing how markers were remapped.

3.3.8 dbvor markeralias

When it is discovered that two marker names represent the same position on the same chromosome, one name is declared the preferred name. **dbVOR** will use this preferred name for output and accept either name for input.

3.3.8.1 NAME

dbvor markeralias - indicate which SNPS have been merged together

3.3.8.2 SYNOPSIS

dbvor makeralias *{ConfigFile.cfg and/or arguments}*

3.3.8.3 ARGUMENTS

--input the input file.
If there is no **--input** specified, then the arguments **--old** and **--new** are the marker names that are to be merged. Otherwise, they indicate the columns in the **--input** file that contains the pairs.

--new the COLUMN NUMBER of the merged marker

--old the COLUMN NUMBER of the original marker

3.3.9 dbvor markerinfo

This program loads the genetic and physical positions for a set of markers and stores the corresponding build name.

3.3.9.1 NAME

dbvor markerinfo - specify chromosome and (absolute and/or genetic) position of the markers

3.3.9.2 SYNOPSIS

dbvor markerinfo *{ConfigFile.cfg and/or arguments}*

3.3.9.3 ARGUMENTS

-active (int) **active** flag value to store in database for this entry

--average COLUMN NUMBER of the average genetic distance (cM)

--build the genome build to which the positions and chromosome number apply. All the marker information that is stored in the database indicates the build the information came from. It is possible to have data from multiple builds in the database at the same time and it may be necessary to select the desired build for output.
If there is only one build in the database, we do not require you to specify the build to be used for generating output.

--chromosome the COLUMN NUMBER of the chromosome name

--female the COLUMN NUMBER of female genetic distance

--input file containing position information about the markers

--male the COLUMN NUMBER of the male genetic distance

--marker the COLUMN NUMBER of the marker name

--position the COLUMN NUMBER of the base pair position on the chromosome

3.3.10 dbvor missingmarkerinfo

This program lists the markers that do not have physical and genetic information stored in the database. A build can be specified so that the program only lists markers of that build that have missing physical and genetic information.

3.3.10.1 NAME

dbvor missingmarkerinfo - print out a list of markers that need position data and suggests code to get the (genetic) position information.

3.3.10.2 SYNOPSIS

dbvor missingmarkerinfo *{ConfigFile.cfg and/or arguments}*

3.3.10.3 ARGUMENTS

--build the genome build to which the positions and chromosome apply. With no build argument, **dbvor missingmarkerinfo** will print out the entire list of valid markers into a file named, *MissingMarkerInfo.txt.* And it will print out the sample code for running **gmi.pl, dbvor gmimarker** and **dbvor gmimarkerALIAS** with an argument file of *MissingMarkerInfo.txt.*
If you supply a **--build** argument, **dbvor missingmarkerinfo** will generate a list of markers which have no genetic data in the ***marker_ info*** table for the chosen build. It will place the list in the file *MissingMarkerInfo_ <build name>.txt* and proceed to generate all the sample code to get marker genetic data for this build.

3.3.11 dbvor member

This program loads the pedigree and person data including the father, mother, and gender for a list of people.

3.3.11.1 NAME

dbvor member - loads pedigree data

3.3.11.2 SYNOPSIS

dbvor member *{ConfigFile.cfg and/or arguments}*

3.3.11.3 ARGUMENTS

**--active**	(int) **active** flag value to store in database for this entry
--gender	*the* COLUMN NUMBER *of the gender field (optional). If unspecified, use default value of the database* ***gender*** *field.*
**--input**	the input file
--father	*the* COLUMN NUMBER *of the father field (optional). If unspecified, use default value of the database* ***father*** *field.*
--mother	*the* COLUMN NUMBER *of the mother field (optional). If unspecified, use default value of the database* ***mother*** *field.*
--noheader	(boolean) indicates that the input file does not have a header line. *If unspecified, use default value False.*
**--pedigree**	the COLUMN NUMBER of the pedigree field
**--person**	the COLUMN NUMBER of the person field

3.3.11.4 USAGE

For examples of usage, see Section 2.4.1.

3.3.12 dbvor memberalias

Sometimes a lab processes a new blood sample with new id, not the original id of the person. Thus the new id corresponds to a person already in the database. We want genotyping for either id of the person to map to the same data.

3.3.12.1 NAME

dbvor memberalias - stores a mapping of new name to the old name

3.3.12.2 SYNOPSIS

dbvor memberalias *{ConfigFile.cfg and/or arguments}*

3.3.12.3 ARGUMENTS

--input the input file

--newpedigree the COLUMN NUMBER of the alias pedigree name

--newperson the COLUMN NUMBER of the alias person name

--oldpedigree the COLUMN NUMBER of the original pedigree name

--oldperson the COLUMN NUMBER of the original person name

3.3.13 dbvor newmarker

Marker names supplied in a data file (i.e. genotype or ped file) must be declared in advance. Otherwise, it is assumed that a new marker name is merely a typing error.

3.3.13.1 NAME

dbvor newmarker - indicate valid marker names

3.3.13.2 SYNOPSIS

dbvor newmarker *{ConfigFile.cfg and/or arguments}*

3.3.13.3 ARGUMENTS

--active (int) **active** flag value to store in database for this entry

--input the input file

--marker the COLUMN NUMBER of marker name in the input file

3.3.14 dbvor plink

This program reads a PLINK .fam file and loads the pedigree and person data including the father, mother, and gender for a list of people into the Member table. Then it reprocesses the same data to populate the Sample table. Finally, if a trait column is available, these data are used to create an entry in the Trait table for each person. Typical usage requires the specification of at least the pedigree and person column numbers.

3.3.14.1 NAME

dbvor plink - loads pedigree data, sample data, and trait data from a PLINK *.fam* file

3.3.14.2 SYNOPSIS

dbvor plink *{ConfigFile.cfg and/or arguments}*

3.3.14.3 ARGUMENTS

**--active**	(int) **active** flag value to store in database for this entry
--gender	*the* COLUMN NUMBER *of the gender field (optional). If unspecified, use default value of the database* ***gender*** *field.*
**--input**	the input file
--father	*the* COLUMN NUMBER *of the father field (optional). If unspecified, use default value of the database* ***father*** *field.*
--mother	*the* COLUMN NUMBER *of the mother field (optional). If unspecified, use default value of the database* ***mother*** *field.*
--noheader	(boolean) indicates that the input file does not have a header line. *If unspecified, use default value False.* **NOTE: This argument should always be TRUE for .fam files.**
**--pedigree**	the COLUMN NUMBER of the pedigree field
**--person**	the COLUMN NUMBER of the person field

**--trait**	the COLUMN NUMBER of the trait field *(optional)*. This argument causes the indicated column to be loaded into the Trait table of **dbvor**.

3.3.14.4 ADDITIONAL TRAIT ARGUMENTS

If a **--trait** column is specified, the additional arguments below may be supplied:

**--missing_phenotype**	a trait value that indicates the trait is missing (default -9 or 0)
**--default_trait_type**	a type to use for trait column (default 'A' (for affection status); use 'T' for quantitative value)
**--default_trait_name**	name to use for trait column (default 'PLINKTrait')
**--stat**	generate statistics for the trait: show values and counts
**--nostat**	do not generate statistics for the trait

3.3.15 dbvor plinksample

This program reads a PLINK .fam file and processes the data to populate the Sample table. Each row added will contain the experiment name specified in the configuration file, because the sample name generated from a .fam file, <pedigree>_<person> may not by itself be unique. Typical usage requires specifying both the pedigree and person column numbers. If only the person column is specified, the sample names generated will be in the form UNUSED_<person>.

3.3.15.1 NAME

dbvor plinksample - loads sample data from a PLINK *.fam* file

3.3.15.2 SYNOPSIS

dbvor plinksample *{ConfigFile.cfg and/or arguments}*

3.3.15.3 ARGUMENTS

--active (int) **active** flag value to store in database for this entry

--experiment name of the experiment

--input the input PLINK fam file

--noheader (boolean) indicates that the input file does not have a header line. *If unspecified, use default value False.* **NOTE: This argument should always be TRUE for .fam files.**

--technology name of the technology use by the experiment

--pedigree the COLUMN NUMBER of the pedigree field in the PLINK fam file

--person the COLUMN NUMBER of the person field in the PLINK fam file

3.3.16 dbvor sample

The sample table is used to relate a laboratory sample to its meta data and ultimately to the pedigree:person to whom the measurements belong. An attempt to process data that does not have a sample record in the database will fail; it will not be processed. If the pedigree and person are both NULL, the data record will be stored in the database, but currently such a record can not be accessed and its data will not appear in any output request. *This mechanism is currently only used for genome-wide data.*

3.3.16.1 NAME

dbvor sample - load pedigree mapping as well as plate, well and other details of a samples

3.3.16.2 SYNOPSIS

dbvor sample *{ConfigFile.cfg and/or arguments}*

3.3.16.3 ARGUMENTS

--active (int) **active** flag value to store in database for this entry

--input the input file. The first line is a header line and the remaining lines are interpreted according to the table below.

--project project name

--experiment name of the experiment

--technology the technology

3.3.16.3.1 sample file format The first line of the sample file is a header line, and the remaining lines are interpreted according to this table:

**column name**	**column**	**interpretation**
subject_id	1	simple name for sample (for reference only)
active_value	2	active flag value for genotype records generated for this sample
pedigree	3	corresponding pedigree value for the sample
person	4	corresponding person value for sample
plate	5	plate identifier for sample (for reference only)
well	6	well identifier for sample on plate (for reference only)
misc	7	additional information about the sample (for reference only)
sample_id	8	the laboratory name of the sample

Note: Only the first 8 columns of each line are examined; any additional columns are not processed.

3.3.17 dbvor set_experiment_date

The database experiment record lets you associate a date with the experiment. By default, the date is the time the experiment name is entered into the database. It might be desirable to change the date to when the information was collected, etc.

3.3.17.1 NAME

dbvor set_experiment_date - set experiment's date field

3.3.17.2 SYNOPSIS

dbvor set_experiment_date *{ConfigFile.cfg and/or arguments}*

3.3.17.3 ARGUMENTS

**--experiment**	name of the experiment
**--experiment_date**	the format is either MM/DD/YYYY or YYYY-MM-DD
**--technology**	name of the technology

3.3.18 dbvor trait

dbvor trait enters trait names and values and validates the values based on the trait types

3.3.18.1 NAME

dbvor trait - store one or more trait values for specified pedigree members

3.3.18.2 SYNOPSIS

dbvor trait *{ConfigFile.cfg and/or arguments}*

3.3.18.3 ARGUMENTS

--columns (list) the **COLUMN NUMBER** of a trait field. This field can be repeated as many times as necessary.

--input the input file

--nostat *(present/true) do not print trait value statistics*

--pedigree the **column number** of the pedigree field

--person the **column number** of the person field

--record DEFUNCT!

--stat *(present/true) print trait value statistics*
Below is a small sample of trait statistics:

```
==== Starting the "trait1" subsection
trait "trait1" type A col 6 (db id 4)
           value   count
            '0'        2
            '1'        2
            '2'        5
trait1: min 0.0, max 2.0, avg 1.33333333333, count 9, ...
NA's 0, ""'s 0
```

--titles *(list) the name of the trait column. If not specified the name is taken from the header of the specified column in the* ***--input*** *file. This field can be repeated as many times as necessary.*

--types (list) the type of the corresponding column. This field can be repeated as many times as necessary. **A** is used to indicate an affective trait (values 0, 1, or 2). **T** is used to indicate a numeric (or categorical) trait.

3.3.19 dbvor DeleteBlockAllSamples

Deletes all the rows in the *GenotypeBlocks* table for the specified experiment. It also clears the file name column of the *Samples* table which generally indicates where the data for the sample came from. (The data structures used to store genome-wide data are explained more fully in sections: 3.4.2, 3.4.3, 3.4.4, 3.3.16, and 2.7.)

3.3.19.1 NAME

dbvor DeleteBlockAllSamples - deletes genome-wide style information for experiments

3.3.19.2 SYNOPSIS

dbvor DeleteBlockAllSamples *{ConfigFile.cfg and/or arguments}*

3.3.19.3 ARGUMENTS

--experiment name of the experiment

--technology name of the technology

3.3.20 dbvor DeleteBlockMember

Deletes the rows in the *GenotypeBlocks* table for the specified member(s). It also clears the filename column of the *Samples* table which generally indicates where the data for the sample came from. (The data structures used to store genome-wide data are explained more fully in sections: 3.4.2, 3.4.3, 3.4.4, 3.3.16, and 2.7.)

3.3.20.1 NAME

dbvor DeleteBlockMember - deletes genome-wide style information for members

3.3.20.2 SYNOPSIS

dbvor DeleteBlockMember *{ConfigFile.cfg and/or arguments}*

3.3.20.3 ARGUMENTS

--experiment name of the experiment

--technology name of the technology

--member (list) the name of a person (pedigree:person) whose data should be deleted

3.3.21 dbvor DeleteBlockSample

Deletes the rows in the *GenotypeBlocks* table for the specified experiment and the given samples. It also clears the filename column of the *Samples* table which generally indicates where the data for the sample came from. (The data structures used to store genome-wide data are explained more fully in sections: 3.4.2, 3.4.3, 3.4.4, 3.3.16, and 2.7.)

3.3.21.1 NAME

dbvor DeleteBlockSample - deletes genome-wide style information for samples

3.3.21.2 SYNOPSIS

dbvor DeleteBlockSample *{ConfigFile.cfg and/or arguments}*

3.3.21.3 ARGUMENTS

√

**--experiment**	name of the experiment
**--technology**	name of the technology
**--sample**	(list) the name of a sample whose data should be deleted

3.3.22 dbvor DeleteSnpBlockMap

Deletes all the rows in the *SnpBlocks* table for the specified technology. This undoes the **--make-snps** operation that loads marker names and marker position data to this table. (The data structures used to store genome-wide data are explained more fully in sections: 3.4.2, 3.4.3, 3.4.4, 3.3.16, and 2.7.)

3.3.22.1 NAME

dbvor DeleteSnpBlockMap - deletes genome-wide style information for the technology

3.3.22.2 SYNOPSIS

dbvor DeleteSnpBlockMap *{ConfigFile.cfg and/or arguments}*

3.3.22.3 ARGUMENTS

--technology name of the technology to forget

3.3.23 dbvor ShowSamples

Displays the contents of the samples table. The table maps a sample name to the pedigree/person. It also indicates where the data for each sample was loaded from and what experiment name is. To allow for maximum flexibility the same sample name may appear in several rows iff the experiment name is different.

3.3.23.1 NAME

dbvor ShowSample - displays the sample table

3.3.23.2 SYNOPSIS

dbvor ShowSample *{ConfigFile.cfg and/or arguments}*

3.3.23.3 ARGUMENTS

--experiment an experiment name, to be used whenever **--sample** (below) is specified

--sample (list) a sample name to display info for (*Optional*)

--technology the technology name, to be used whenever **--sample** is specified

--output the stem for the output file *(Optional)*

If **--output** is specified, a unique named output file is generated for each experiment. The file contains the sample entries for the particular experiment. The file name is <stem>.<experiment>.txt. <stem> comes from the **--output** argument while <experiment> comes from the column in the samples table.

If no sample items are specified, then the entire sample table is displayed.

3.4 Genotype Entry

We call a collection of SNP data associated with some markers and some people an *experiment*. A study will have one or more experiments. As data are generated, they should be captured in a file and the file can then be loaded into the database. The allele measurements are made using a *technology* such as TaqMan, iPLEX, etc. We record the *technology* for later data selection; we also record the file name as the name of the *experiment*.

3.4.1 Geno

geno loads genotype data from targeted experiments into the database; it stores each genotype as a separate table row. It should not be used for genome-wide data.

3.4.1.1 NAME

geno - enter genotype data into the genotype table

3.4.1.2 SYNOPSIS

geno *{ConfigFile.cfg and/or arguments}*

3.4.1.3 ARGUMENTS

The arguments: **--compare, --delete, --replace, and --verify** are mutually exclusive.

--active (active) value for **--active** field of each genotype record

--compare *(present/true)*
--compare *will only examine data that is already in the database. It uses the* ***experiment*** *file to select what person/pedigree/markers to look at. It will indicate what conflicts there are in the genotype values already in the database. If you want to check what data conflicts there are between the* ***experiment*** *file data and the existing database, use* ***--verify****.*
running ***geno Config.cfg --compare -v***
after a ***--commit*** *will generate exactly what* ***--verify*** *did before because the data are now in the database.*

--delete (present/true) removes the data matching the original **experiment** file data from the database

--exclude *(list) pedigree and* persons *NOT to include from those otherwise being included. The records will be ignored. The "normally" included are all the people in* ***experiment*** *file or if* ***--include*** *is specified, just those pedigree/persons being explicitly included. In some sense,* ***--exclude*** *happens after* ***--include*** *and prunes individuals.*
This argument can be followed by multiple person-pedigree pairs separated by commas (",") and/or there can be multiple ***--exclude*** *lines in the configuration file. People are specified by writing the pedigree, a ":", and the associated person. You can specify both a pedigree and person to identify a unique individual. If you specify just a pedigree and no person, then all persons of that pedigree will be excluded. If you specify a person and no pedigree, or if you just specify a token without an initial ":", then that person identifier in every pedigree will be excluded.*

--experiment the name of the file that contains the experiment data. As a convenience, this name will be entered into the database as the experiment name if it is not there already.

--include *(list) explicit pedigree and persons proposed for inclusion from the **experiment** file. They will be included unless they also appear in the **--exclude** list. For example, if you want to include most but not all people of a pedigree;you can **--include** by the pedigree and **--exclude** the specific person.*
*This argument can be followed by multiple person-pedigree pairs separated by commas (",") and/or there can be multiple **--include** lines in the configuration file. People are specified by writing the pedigree, a ":", and the associated person. You can specify both a pedigree and person to identify a unique individual. If you specify just a pedigree and no person, then all persons of that pedigree will be considered for inclusion. If you specify a person and no pedigree, or if you just specify a token without an initial ":", then that person identifier in every pedigree will be considered for inclusion.*

--insert_many *(int) the number of genotype records to collect before a single database insert of the genotypes is performed. Default is 100 records.*

--marker the COLUMN NUMBER of the first marker field. Each column starting with the **--marker** column specifies alleles. So for example, in "wide" format (**--type=wide**), the **--marker** column contains the first allele and the next column the second. If there were more markers on the line, the next marker would take the next pair of columns, etc

--nostat *(present/true) inhibits the generation of statistics during the run*

--pedigree the COLUMN NUMBER of the pedigree field

--person the COLUMN NUMBER of the person field

--quality *(float) value for **--quality** field of each genotype record*

--replace (present/true) reloads the genotype entries from the **experiment** file

--sep *the column separator for multi column files. The default separator is any kind of white space. Comma separated files (.csv) should use "," for the separator. Tab separated files should use "<tab>" as the separator.*
If the file is tab separated and there are empty values it is very important to indicate the separator is "<tab>" and not white space.

--stat *(present/true) print statistics at the end of the run*

--technology name of the technology

--type description of how the alleles are presented in the file

**value**	**meaning**
wide	each allele is placed in a separate column of the **--experiment** file. For each marker, the column headers contain the marker name followed by a "-1" or a "-2" depending whether this is the first or second allele for the marker.
half	each allele pair is placed in a separate column of the **--experiment** file
long	like plink lgen format, each line specifies a pedigree/person/marker and one allele pair

--verify *(present/true)*
--verify *will pretend that the* ***experiment*** *file was added to the database. It will indicate what conflicts there would be with the genotype values already in the database versus the new values in the* ***experiment*** *file. Conflicts arise when a given person/pedigree/markers already has a value in the database but has a different value an the* ***experiment*** *file.*
If you want to check what genotype data conflicts there are already in a database, for the values to be entered by an ***experiment*** *use:*
geno Config.cfg --compare -v

--verify_in *(present/true)* ***--verify*** *(and* ***--compare****) is used to show conflicts between the database and the* ***--experiment file****. If there are no matching genotypes in the database, there can be no conflict. If not having a match indicates an actual error for the protocol you are using, this option will print each element that is not in the database.*

--verify_show_all
(present/true) ***--verify*** *(and* ***--compare****) is used to show conflicts between the database and the* ***--experiment*** *file. They do not indicate that there are multiple genotypes when the values are the same or when some values indicate an unsuccessful measurement. This option will print an indication for these "uninteresting" cases.*

3.4.1.4 Deprecated Arguments

--allele_flip (int)
--output
--translate (present/true)
--xlate (present/true)

3.4.2 Genoi

Processing a genome-wide format file requires two steps. The first step builds an index that will provide an offset, block number, and chromosome for every marker. This step is invoked by supplying the argument, **--make-snps**, to the **genoi** command. The second step reads all the genotypes and places the allele data for each marker into the appropriate block and offset. There are **dbvor** commands to delete these data are: 3.3.22, 3.3.19, 3.3.20, and 3.3.21 and and the command to show the relevant sample entries is: 3.3.23. They were presented earlier in the **dbvor** section.

3.4.2.1 NAME

genoi --make-snps - load marker names and marker position data

genoi - load genotype data

3.4.2.2 SYNOPSIS

genoi --make-snps *{ConfigFile.cfg and/or arguments}*

genoi *{ConfigFile.cfg and/or arguments}*

3.4.2.3 ARGUMENTS

genoi --make-snps program invocation indicates how to find the marker information in the **--ilmnmap** file. Note: The name **ilmnmap** is historical now the program can be used for any type of genome-wide marker data that is easily describable in a columnar format.

3.4.2.3.1 make-snps marker data

--active (int) value for **--active** field of each marker record

--alleles the COLUMN NUMBER of combined alleles. This is used to capture the special 5 character allele format [A/B]; where A and B are the two alleles. This format is used in place of the two allele options below.

**--allele1**	the **COLUMN NUMBER** of SNP first allele. This indicates the reference allele value.
**--allele2**	the **COLUMN NUMBER** of SNP second allele. This indicates the other allele value.
--build	*build id to use in marker_info table for markers. If* ***--build*** *is present, the available chromosome and position data will be copied from the* ***--ilmnmap*** *file into the marker_info table.*
**--chr**	the **COLUMN NUMBER** of SNP chromosome
--dedup	*DEPRECATED. (present/true) special invocation that causes the* ***--active*** *flag to be set to 0, for all but the first instance of all the SNPs sampled multiply (same SNP with different assay name). The SNP marker name for a repetition will have a suffix of ".#", where # is an increasing integer value.*<br>*The program will terminate after performing this request.*
**--experiment**	experiment name
**--ilmnmap**	the input file for SNP data
**--make-snps**	(present/true) (No value should be specified.)
**--name**	the **COLUMN NUMBER** of plate assay name for the wells
**--position**	the **COLUMN NUMBER** of chromosome position
--probeid_remap	*DEPRECATED. file containing a remapping for assay names. Superseded by marker_info table.*
**--project**	project name
**--reposition**	*DEPRECATED. update chromosome, physical and genetic position data from* ***gmi.pl*** *results. Superseded by marker_info table.*
--rs_remap	*DEPRECATED. file containing a remapping for SNP names. Superseded by marker_info table.*

--seqlocklim *(int) DEPRECATED. Sequence bound. Each index block contains an integer value from 0 to this value; an equal number of blocks have the value 0, and 1 and 2 ...*

--snp COLUMN NUMBER of marker name if available and different from **--name**

--snp_gclean_file_output *DEPRECATED. file name for external file. SNP data will be written to this file in Geneva data cleaning format.*

--snp_db_output (boolean) True (**True must be specified**.) The SNP to block index should be written to the database.

--snp_exclude *(list) list of assay names to exclude from the database. Multiple names separated by comma and/or multiple statements may be used.*

--snp_file_output *file name to store SNP to block index into for debugging. If this argument is not present, the index is not written.*

--technology technology name

--type ilmn (This constant is required as is.) Note: More than the Illumina GWAS format is supported by this program.

3.4.2.3.2 genotype data **genoi** program will read all the genotype data from the **--input** files and load it into the genome-wide blocks.

--allele1 the COLUMN NUMBER of SNP first allele

--allele2 the COLUMN NUMBER of SNP second allele

--call the COLUMN NUMBER of call quality value for SNPs. Note: This information is NOT stored in the database currently, but is could be.

--db_output (present/true) **required to be set.** This value indicates that the genotype data blocks should be written to the database.

--experiment experiment name

--filter_samples *(boolean) 0/1. If true, then a data sample that is not in the samples table or does not map to a valid pedigree/person or is not active, is not stored in the database.*

--file_output *file name to store records into for debugging. If this argument is not present, the data is not written.*

--inputdir the directory for the **--input** file(s)

--input the input file (pattern) We allow the standard Unix shell file name expansion indicated by embedding "*" and/or "?" in the file name. Note: Samples may not span multiple files. One sample per file is preferred.

--multi (present/true) the input file contains multiple blocks of contiguous samples

--name the **COLUMN NUMBER** of plate assay name

--NoCall *(boolean) 0/1. Print markers that could not be called.*

--NotSampled *(boolean) 0/1. Print markers that were not sampled in the input file(s).*

--project project name

--sample the COLUMN NUMBER of sample name.
If **--multi** is set, this column contains the sample name which is used to look up the name in the *Sample* database table.
Otherwise, the **--sample** column in the data file is first checked against the input file name. It must match the end of the input file name just before the suffix (viz. .csv, .txt, .gz, ...), i.e. sample 123_456 would be expected to be found in file "mydata_123_456.csv". Note: The full file name "mydata_123_456.csv" is used to look for the matching pedigree/person in the *Sample* database table.

--snp_db_input (present/true) **required to be set**. This value indicates that the assay to block/offset mapping should be read from the database.

--snp_file_input *DEPRECATED. external file. This value indicates that the assay to block/offset mapping should be read from the external file.*

--technology technology name

--type ilmn (This constant is required.)

3.4.3 Genok

Processing genome-wide PLINK binary data requires two steps. The first step builds an index that will provide an offset, block number, and chromosome for every marker. This step is invoked by supplying the argument, **--make-snps**, to the **genok** command. The second step reads all the genotypes from the *.bed* file and places the allele data for each marker into the appropriate block and offset. There are **dbvor** commands to delete these data are: 3.3.22, 3.3.19, 3.3.20, and 3.3.21 and and the command to show the relevant sample entries is: 3.3.23. They were presented earlier in the **dbvor** section.

3.4.3.1 NAME

genok --make-snps - load marker names and marker position data

genok - load genotype data

3.4.3.2 SYNOPSIS

genok --make-snps *{ConfigFile.cfg and/or arguments}*

genok *{ConfigFile.cfg and/or arguments}*

3.4.3.3 ARGUMENTS

genok --make-snps program invocation processes the marker information in the **--plinkbim** file.

3.4.3.3.1 make-snps marker data

--active (int) value for **--active** field of each marker block

--build *build id to use in marker_info table for markers. If* ***--build*** *is present, the available chromosome and position data will be copied from the* ***--plinkbim*** *file into the marker_info table.*

--experiment experiment name

--inputdir directory containing the *.bim* file

--make-snps (present/true) (No value should be specified.)

--plinkbim the name of the input *.bim* file

--project project name

--snp_db_output (boolean) True (**True must be specified**.) The SNP to block index should be written to the database.

--snp_file_output *file name to store SNP to block index into for debugging. If this argument is not present, the block index is not written.*

--technology technology name

3.4.3.3.2 genotype data **genok** program will read all the genotype data from the PLINK **bed** file and load it into the genome-wide blocks.

--active (int) value for **--active** field of each data block

--db_output (present/true) **required to be set.** This value indicates that the genotype data blocks should be written to the database.

--experiment experiment name

--inputdir the directory for the PLINK *.bed*, *.fam*, and *.bim* files.

--plinkbed the name of the input *.bed* file

--plinkfam the name of the input *.fam* file

--plinkbim the name of the input *.bim* file

--project project name

--snp_db_input (present/true) **required to be set**. This value indicates that the block/offset mapping should be read from the database.

--technology technology name

3.4.4 Genoa

While this feature works as described, THIS FEATURE IS DEPRECATED AND MAY NOT BE SUPPORTED IN THE FUTURE.

Processing a genome-wide format file requires two steps. The first step builds an index that will provide an offset, block number, and chromosome for every marker. This step is invoked by supplying the argument, **--make-snps**, to the **genoa** command. The second step reads all the genotypes and places the allele data for each marker into the appropriate block and offset. There are **dbvor** commands to delete these data are: 3.3.22, 3.3.19, 3.3.20, and 3.3.21 and and the command to show the relevant sample entries is: 3.3.23. They were presented earlier in the **dbvor** section.

3.4.4.1 NAME

genoa --make-snps - load marker names and marker position data

genoa - load genotype data

3.4.4.2 SYNOPSIS

genoa --make-snps *{ConfigFile.cfg and/or arguments}*

genoa *{ConfigFile.cfg and/or arguments}*

3.4.4.3 ARGUMENTS

genoa --make-snps program invocation indicates how to find the marker information in the **--affymap** file. Note: This program processes several different Affymetrix plates. The code is general enough to serve as a template for additional plates from Affymetrix and could be extended to other vendors.

3.4.4.3.1 make-snps marker data

--active (int) value for **--active** field of each marker record

--affymap the input file

--affymapfmt *the input file format Xba132 or default*

--build *build id to use in marker_info table for markers. If* ***--build*** *is present the available chromosome and position data will be copied into the marker_ info table.*

--dedup *DEPRECATED. (present/true) special invocation that causes the* ***--active*** *flag to be set to 0, for all but the first instance of all the SNPs sampled multiply (same SNP with different assay name). The SNP marker name for a repetition will have a suffix of ".#", where # is an increasing integer value.*
The program will terminate after performing this request.

--experiment experiment name

--genmap *DEPRECATED. (int) item in* ***--genmapfield*** *of chosen genetic average distance. Replaced by* ***dbvor gmimarker*** *and* ***dbvor markerinfo****.*

--genmapfield *DEPRECATED. (int) field in the input record that specifies the average genetic distance items. Replaced by* ***dbvor gmimarker*** *and* ***dbvor markerinfo****.*

--make_ samples *DEPRECATED. (present/true) make sample table. The file data is provided in by the* ***--samplemap*** *argument. Program terminates after this operation. This is replaced by* ***dbvor samples****.*

--make-snps (present/true) (No value should be specified.)

--more_ samples *DEPRECATED. (present/true) augment sample table. The file data is provided in by the* ***--samplemap*** *argument. Program terminates after this operation. This is replaced by* ***dbvor samples****.*

--probeid_remap *DEPRECATED. file containing a remapping for assay names. Superseded by marker_info table.*

--old_snp_remap *DEPRECATED. file containing a remapping for one particular use case, too idiosyncratic to describe.*

--project project name

--reposition *DEPRECATED. update chromosome, physical and genetic position data from* ***gmi.pl*** *results. Superseded by marker_info table.*

--rs_remap *DEPRECATED. file containing a remapping for SNP names. Superseded by marker_info table.*

--samplemap *DEPRECATED. file that maps sample to pedigree person and provides additional sample meta data. It is used by* ***--make_samples*** *and* ***--more_samples****.*

--seqlocklim *DEPRECATED. (int) Sequence bound. Each index block contains an integer value from 0 to this value; an equal number of blocks have the value 0, and 1 and 2 ...*

--snp_gclean_file_output
DEPRECATED. file name for external file. SNP data will be written to this file in Geneva data cleaning format.

--snp_db_output (boolean) True (**True must be specified,**) The SNP to block index should be written to the database.

--snp_exclude *(list) list of assay names to exclude from the database. Multiple names separated by comma and/or multiple statements may be used.*

--snp_file_output *file name to store records into for debugging. This puts the* SNP to block *index in an external file. If this argument is not present, the index is not written.*

--technology technology name

--type affy (This constant is required.) **genoa** knows the layout of the **--affymap** file; it knows where the marker and assay names and positions are.

3.4.4.3.2 genotype data **genoa** program will read all the genotype data from the **--input** file pattern and load it into the genome-wide blocks.

--call_sfx (list) suffix of column header that contains the called alleles. If the **call_sfx** were ".chp Call" and a the column header was "123_011013.chp Call", this column would be identified as containing the allele data for sample_id 123_011013. (The sample_id might be the concatenation of some sample information and a run date.) The entire prefix, 123_011013, is used as the *sample_id* and looked up in the *Sample* table to get the corresponding pedigree/person.

--db_output (present/true) **required to be set**. This value indicates that the genotype data blocks should be written to the database.

--experiment experiment name

--extra_hdr *(list) column headers that should be ignored during processing. Values may be separated by "," or the argument/value may be repeated.*

--extra_lines *(int) additional lines of commentary in the header in addition to the column header line. These lines will be ignored.*

--filter_samples *(boolean) 0/1. If true, a data sample that is not in the samples table or does not map to a valid pedigree/person or is not active, is not stored in the database.*

--file_output *file name to store records into for debugging. If this argument is not present, the data is not written.*

--inputdir the directory for the **--input** file(s)

--input the input file (pattern) We allow the standard Unix shell file name expansion indicated by embedding "*" and/or "?" in the file name. Note: Samples may not span multiple files, but a file often has multiple samples in disjoint columns.

--nocachefile *(present/true) do not cache the input file in memory even if it has multiple samples. If there are multiple samples on each line of the file, multiple scans through the file are necessary. If the number of markers is small,* ***genoa*** *can cache all the lines and not have to re-read and re-parse the file to get the next sample. (Note:* ***genoa*** *processes all the markers for a sample, before processing any markers of the next sample.)* ***--nocachefile*** *turns this feature off for large files.*

--NoCall *(boolean) 0/1. Print markers that could not be called.*

--NotSampled *(boolean) 0/1. Print markers that were not sampled in the input file.*

--project project name

--qual_sfx (list) suffix of column header that contains the call quality. We do not currently process call quality.

--snp_db_input (present/true) **required to be set**. This value indicates that the assay to block/offset mapping should be read from the database.

--snp_file_input *DEPRECATED. external file. This value indicates that the assay to block/offset mapping should be read from the external file.*

--subsample *DEPRECATED. (list) list of sample names. The specified samples are selected from the input files and stored in individual files.*

--subsampleall *DEPRECATED. directory name. All the samples from the input files are located and stored in individual files in the specified directory.*

--subsamplecnt *DEPRECATED. (int) number of columns in a sample. (default 5). When selecting the subsamples from the input files, assume that a sample consists of this many columns from the start column.*

--technology technology name

--type affy (This constant is required.) **genoa** knows the layout of the **--affymap** file, i.e. where the marker and assay names and positions are.

3.5 Data extraction using genout

The **genout** program selects target experiment and genome-wide experiment genotype data from the relevant database tables. It resolves inconsistencies if there are more than one measurement for a given pedigree/person/marker. It also allows you to select only a subset of people and markers.

3.5.1 NAME

genout - selects different subsets of the data from the dbVOR database

3.5.2 SYNOPSIS

genout *{ConfigFile.cfg and/or arguments}*

3.5.3 ARGUMENTS

3.5.3.1 Formatting Arguments

argument	meaning
**--build**	the genome build from which to extract the chromosome, physical positions and genetic positions. This information is kept in the database.<br>If there is only one build in the database, we do not require you to specify the build to be used for generating output.
--filter_by_active	*(boolean) only fetch the genotype's alleles with the highest* ***active*** *value(s) when there is more than one measurement available. (The default value is True.)*
--funny	*(boolean) use special characters to indicate data problems, for wide/half/long output formats:*<br>*Q indicates no trait value found.*<br>*Z indicates no samples measured.*<br>*F indicates conflicting allele pairs found.*

--heterozygote *indicates how to order individual alleles in the heterozygous case. (see table below) (Default is "lexical".)*

--gmi_position *file of **gmi.pl** produced data.*
*These data will be used for the genetic information rather than data from the marker_info table for the chosen **build.** For Mega2 format output, lines of this file will be copied to the created map file instead of using the database marker_info table.*

--member *(list) a list of pedigree,":", and person concatenated together entries. These specify a selection of people to be processed.*

--missing_phenotype
*value to be used for PLINK style output (**bed**, **lgen**, **ped**, and **tped**) if the actual phenotype value is missing. By default, this value is the string "-9".*

--no_markers *(present/true) ignore markers listed in the config file. In addition, if there are no marker names listed, do not default to all markers, default to NO markers.*

--no_traits *(present/true) ignore any available trait data.*

--onetwo *(boolean) code the output alleles as "A" and "B" for the first allele and other allele versus the actual letter alleles. This is to produce results compatible with other old programs/data.*

--output indicates the stem of the output file. Some formats generate more than one file.

--OFS *is an alternate name for the **--separator***

--person_order *(boolean) turns off statistics and other processing so as to minimize the memory footprint during processing. After all the markers for each person are processed, all data and meta data are purged. This request is compatible with bed, ped, lgen, and mega2 formats. It will cause plink bed to produce a "individual major" binary file rather than the "snp major".*

--separator *supplies the output file column separator for wide/half/long*

--short *(boolean) do not print out unmeasured marker values in long format output. The long format needs no place holders like wide format does, since every line contains the person, marker and allele info.*

--statistics *(present/true) produces statistics, agreement matrix, etc. derived from merging multiple experiments while the data are filtered and processed. This feature is not on by default because there may not be enough memory to save the statistics for a genome-wide size **dbVOR** database.*

--stat *(present/true) is the same as **--statistics***

--tech *(boolean) 0/1/ restrict pedigree:persons for analysis to be those that are measured in the technologies explicitly selected (later).*

--type indicates the output format desired. (see table below)

--unobserved *ALMOST DEPRECATED (present/true) counts the number of missing markers per person. This inhibits all other output processing and the output from **genout** is a table showing:*
person pedigree <total markers> <missing markers> <found markers>
The table is sorted by <missing markers> then pedigree and person.

--use_inactive *(boolean) allow genotypes with **active** value of 0 to be selected from the database. (The default value is False.)*

3.5.3.2 Type values

value	meaning
bed	PLINK binary format, normally snp-major order but **--person_order** forces individual-major order

half	each allele pair is placed in a separate column of the output file
lgen	PLINK lgen format: one pedigree/person/marker allele per line
long	like PLINK lgen format, each line specifies a pedigree/person/marker and one allele pair
mega2	Mega2 file format
ped	PLINK ped format
tped	PLINK tped format
wide	each allele is placed in a separate column of the output file

3.5.3.3 Heterozygote values

**value**	**meaning**
allsubjects	place allele with lower minor allele frequency first. Frequency computed over all subjects. This option performs exactly like the same named PLINK option.
founders	place allele with lower minor allele frequency first. Frequency computed over just the founders. This option performs exactly like the same named PLINK option.
keep-allele-order	use allele order of first person in the database that has this marker. This option performs exactly like the same named PLINK option.
lexical	sort the alleles alphabetically.
unchanged	use order presented from the database.

3.5.3.4 Filters

Note: Since all filter are optional, we will not make all the text below italic to indicate an optional argument.

3.5.3.4.1 Select by marker, chromosome, etc.

argument **value template** **meaning**

**--chrCMrange**	*chr*:*start*..*end*	(list) include all markers on chromosome *chr* between average genetic positions *start* thru *end*. Note: *chr* is followed by a ":" and *start* and *end* are separated by "..". Either *start* or *end* can be missing, in which case *start* is 0 and *end* is "the end". Note: The values are decimal fractions. Note: All marker positions are chosen from the selected genome build.
**--chromosome**	1	(list) includes all markers in the database on a given chromosome.
**--chrrange**	*chr*:*start*..*end*	(list) include all markers on chromosome *chr* between physical positions *start* thru *end*. Note: *chr* is followed by a ":" and *start* and *end* are separated by "..". Either *start* or *end* can be missing, in which case *start* is 0 and *end* is "the end". Note: All marker positions are chosen from the selected genome build.
**--marker**	rs1073516	(list) lists of markers to output. Note: If no marker_info data (i.e. physical or genetic position) exist for the marker, the only way to select it for output is to list it explicitly.

3.5.3.4.2 Select by member

argument	value template	meaning

**--exclude**	pedigree:person	(list) excludes those people with the indicated pedigree and/or person from the list of otherwise included persons.<br>A ":" is used to separate the two parts: the pedigree and the person. Specifying both a pedigree and person identifies a unique individual. You may also specify just the pedigree to select all members in the pedigree (Indicated as "pedigree:", viz. omit the person) or specify just the person to select that person id in all pedigrees that have the id (indicated as ":person", viz. omit the pedigree specifier, You can also omit the ":" in this case.)
**--fill_pedigree**		(boolean) after persons are selected by the mechanisms explained in this section (and the trait section below), add all persons in each of the pedigrees that have at least one person selected unless these persons are explicitly **--exclude**d (above).
**--include**	pedigree:person	(list) includes only those people with the indicated pedigree and/or person.<br>A ":" is used to separate the pedigree and the person. Specifying both a pedigree and person identifies a unique individual. You may specify just the pedigree to select all members in the pedigree (Indicated as "pedigree:", viz. omit the person specifier) or just the person to select that person id in all pedigrees that have the id (indicated as ":person", viz. omit the pedigree specifier, You can also omit the ":" in the latter case.)
**--member**	pedigree:person	(list) includes only those people with the indicated pedigree and person

3.5.3.4.3 Select by trait

<table>
<tr><th>argument</th><th>value template</th><th>meaning</th></tr>
<tr><td>--trait</td><td>*name*|**value**|*v1*|*v2*
or
name|**value**|*v1*
name|**value**|*v2*</td><td>(list) includes persons where the indicated trait *name* has the values *v1* or *v2.* You may indicate multiple values as done in the first example by having them on the same line and separating them with "|". Or you can have one value specified on each of several lines.
If at least one trait is selected, only selected traits will appear in the output. Otherwise, the default is to output all traits values.</td></tr>
<tr><td>--trait</td><td>*gender*|**value**|*v1*|*v2*</td><td>(list) as above. **genout** treats sex as a trait. (Though it is maintained as part of the family structure internally.)</td></tr>
<tr><td>--trait</td><td>*name*</td><td>(list) selects trait *name* to appear in the output. It does not filter the members to output.</td></tr>
</table>

3.5.3.4.4 Select by technology and experiment

<table>
<tr><th>argument</th><th>value template</th><th>meaning</th></tr>
<tr><td>--experiments</td><td>dataset3.txt</td><td>(list) indicates which experiments are to be included in the results</td></tr>
<tr><td>--marker</td><td>*name*|**technology**|*T1*</td><td></td></tr>
</table>

		(list) selects genotypes of marker *name* that were measured with the technology *T1.* You may list multiple technology names, by separating them with "|" or using separate lines; the same choices that were explained for **trait** above.
**--marker**	*name*|**experiment**|*E1*	
		(list) as above, but selects genotypes of the marker coming from the selected experiment. You may list multiple experiment names, by separating them with "|" or using separate lines; the same choices that were explained for **technology** above.
**--technologies**	exampletech1	(list) indicates which technologies are to be included in the results

3.5.3.4.5 Filtering Experiment Data These options are useful when cleaning reports that some measurements are unreliable. They always specify at least an experiment name.

**argument**	**value template**	**meaning**
**--experiment**	*exp*|**exclude_chromosome**|*Pped:Pper:Chr*	
		(list) excludes all markers on chromosome, *chr*, for person *Pped:Pper* in experiment, *exp.* Unlike earlier usage, both *Pped* and *Pper* must be specified. You may list multiple **values**, by separating them with "|" or using separate lines.
**--experiment**	*exp*|**exclude_chrranger**|*Pped:Pper:Chr:start..end*	

		(list) as above, but only exclude markers with base pair position between *start* and *end* inclusive on chromosome *Chr* for the selected person. *start* may be omitted and defaults to 0, while *end*, if omitted defaults to the end of the particular chromosome. You may list multiple **values**, by separating them with "|" or using separate lines.
**--experiment**	*exp*|**exclude_genotype**|*Pped:Pper:marker*	(list) as above, but only exclude a specific marker, *marker* for the indicated person. You may list multiple **values**, by separating them with "|" or using separate lines.
**--experiment**	*exp*|**exclude_marker**|*M1:Chr1*|*M2:Chr2*	(list) excludes the marker named *M1* for all persons of experiment, *exp*. You may optionally specify the chromosome of the marker by following the marker name, *M1*, by a ":" and the chromosome. You may list multiple **values**, by separating them with "|" or using separate lines.
**--experiment**	*exp*|**exclude_person**|*Pped:Pper*	(list) excludes all markers for person *Pped:Pper* in experiment, *exp*. Both *Pped* and *Pper* must be specified. You may list multiple people, by separating them with "|" or using separate lines.
**argument**	**value**	**meaning**

**--prune_members**	(boolean) This is a filtering operation but different from those above. It removes all those members from the output that have no markers measured, e.g. founders might not be labeled or the sample may be severely contaminated.<br>Note: If **--fill_pedigree** is also set, members of a pedigree are not removed if at least one other member of the pedigree has some measured markers.

3.6 Miscellaneous Commands

3.6.1 Genote

It is common in the course of analyzing data to have a "notes" file that shows changes made to the data files as the work proceeded. Typically, there are one or more notes files describing one or more data files and experiments. The **genote** program lets you keep all these notes in the database along with the data.

3.6.1.1 NAME

genote - store a note file and related data in a table.

3.6.1.2 SYNOPSIS

dbvor <operation> <argument> **--**commit **--**genote

genote **--**db <where> **--**file <filename> **--**store **--**note <note_filename> **--**comment "... comments ..." **--**keyword kwd1 **--**keyword kwd2

3.6.1.3 ARGUMENTS

3.6.1.3.1 Insert mode Insert mode of note entry is requested explicitly by the command **genote** followed by at least one of the arguments described below. Note: No particular field listed below is required, but typically the note file is specified. (It is possible to carry the note information in the **--comment** or **--keywords**.) Note: This program automatically **--commit**s its data to the database ***note*** table.

argument	short argument	meaning
**--comment**	**-c**	comment lines field for the note
**--date**		a date in MM/DD/YYYY or YYYY-MM-DD format
**--file**	**-f**	an *external file* name that the note references
**--keywords**	**-k**	(list) keyword(s) for the note. (May appear multiple times.)
**--note**	**-n**	a *note file* to store in the database

**--storefile**	**-s**	(present/true) also store the *external file* content (above) in the database
**--summary**		(list) additional lines to add to the **comment** field (may appear multiple times)

3.6.1.3.2 Automatic insertion mode Automatic mode of note entry is requested by adding the argument **--genote** to any **dbVOR** command.

argument	meaning
**--genote**	adds one record to the ***note*** table with the *Configuration File* as the *note file* and the log file contents of the program becomes the *external file* (and the file is stored).<br>If a **--genote_note** or **--genote_text** field is specified with the program, then two records are added to the ***note*** table. The first *note* record is created with the argument **--genote_note** values as the *note file* and the log contents stored as the *external file.* A second record is created with the *Configuration File* as the *note file* and the log file name as the *external file.* (The contents are not stored a second time.)
**--genote_date**	a **filedate** for this note entry
**--genote_note**	a *note file* to be added to database
**--genote_summary**	(list) additional lines added to the **comment** field (may appear multiple times)
**--genote_text**	(list) text to use as the contents of an anonymous *note file* to be added to the ***note*** table. If the argument appears multiple times, all the text is concatenated.

3.6.1.3.3 Fetch mode Fetch mode retrieves information from the ***note*** table. One argument will indicate how much information is returned and another will serve as a filter for retrieving matching records.

Selection: One of these note selection modes must be specified.

argument	meaning
**--fetch**	(present/true) print the non-file data for the note entries matching a search and copy the *note file* and *external file* data from the database for these entries
**--head**	(present/true) for fetch, show only the first 5 lines of the *note file*
**--list**	(present/true) print the non-file data for the note entries matching a search
**--view**	(present/true) print the non-file data for the note entries matching a search and print the *note file* and *external file* data from the database for these entries

Filters: One of these filters should be chosen. Note: below "date" refers to a month, day, year in traditional format mm/dd/yyyy (or yyyy-mm-dd). "time" has two components: first a "date" and then the clock time. Clock time is indicated hh:mm:sec.

argument	meaning
**--all**	(present/true) match all *note* entries
**--comment**	match note entries with given phrase in **comment** field
**--date**	specify a date and match note entries with **filedate** == this date

**--datege**	specify a date and match note entries with **filedate** >= this date
**--datele**	specify a date and match note entries with **filedate** <= this date
**--file**	match note entries with specified *external file* in **filename** field
**--id**	(list) match a single note entry with specified **id** (may appear multiple times)
**--keywords**	match note entries with given word in **keywords** field
**--timege**	specify a time and match note entries with **time_stamp** >= this time
**--timele**	specify a time and match note entries with **time_stamp** <= this time

3.6.1.3.4 Administrative These operations do not ask for confirmation and can destroy data; so be careful. In addition, they all imply that a database **--commit** should happen.

**argument**	**meaning**
**--create**	(present/true) create a new *note* table in the existing database
**--delete**	(int) delete a note entry having the indicated **id**
**--drop**	(present/true) drop the database *note* table

3.7 License agreement

dbVOR is distributed under the GNU General Public License Version 3.

3.7.1 GNU General Public License Version 3 for dbVOR

GNU GENERAL PUBLIC LICENSE

Version 3, 29 June 2007

Copyright (C) 2007 Free Software Foundation, Inc. <http://fsf.org/>
Everyone is permitted to copy and distribute verbatim copies
of this license document, but changing it is not allowed.

Preamble

The GNU General Public License is a free, copyleft license for software and other kinds of works.

The licenses for most software and other practical works are designed to take away your freedom to share and change the works. By contrast, the GNU General Public License is intended to guarantee your freedom to share and change all versions of a program--to make sure it remains free software for all its users. We, the Free Software Foundation, use the GNU General Public License for most of our software; it applies also to any other work released this way by its authors. You can apply it to your programs, too.

When we speak of free software, we are referring to freedom, not price. Our General Public Licenses are designed to make sure that you have the freedom to distribute copies of free software (and charge for them if you wish), that you receive source code or can get it if you want it, that you can change the software or use pieces of it in new free programs, and that you know you can do these things.

To protect your rights, we need to prevent others from denying you these rights or asking you to surrender the rights. Therefore, you have certain responsibilities if you distribute copies of the software, or if you modify it: responsibilities to respect the freedom of others.

For example, if you distribute copies of such a program, whether gratis or for a fee, you must pass on to the recipients the same freedoms that you received. You must make sure that they, too, receive or can get the source code. And you must show them these terms so they

know their rights.

Developers that use the GNU GPL protect your rights with two steps: (1) assert copyright on the software, and (2) offer you this License giving you legal permission to copy, distribute and/or modify it.

For the developers' and authors' protection, the GPL clearly explains that there is no warranty for this free software. For both users' and authors' sake, the GPL requires that modified versions be marked as changed, so that their problems will not be attributed erroneously to authors of previous versions.

Some devices are designed to deny users access to install or run modified versions of the software inside them, although the manufacturer can do so. This is fundamentally incompatible with the aim of protecting users' freedom to change the software. The systematic pattern of such abuse occurs in the area of products for individuals to use, which is precisely where it is most unacceptable. Therefore, we have designed this version of the GPL to prohibit the practice for those products. If such problems arise substantially in other domains, we stand ready to extend this provision to those domains in future versions of the GPL, as needed to protect the freedom of users.

Finally, every program is threatened constantly by software patents. States should not allow patents to restrict development and use of software on general-purpose computers, but in those that do, we wish to avoid the special danger that patents applied to a free program could make it effectively proprietary. To prevent this, the GPL assures that patents cannot be used to render the program non-free.

The precise terms and conditions for copying, distribution and modification follow.

TERMS AND CONDITIONS

0. Definitions.

"This License" refers to version 3 of the GNU General Public License.

"Copyright" also means copyright-like laws that apply to other kinds of works, such as semiconductor masks.

"The Program" refers to any copyrightable work licensed under this License. Each licensee is addressed as "you". "Licensees" and "recipients" may be individuals or organizations.

To "modify" a work means to copy from or adapt all or part of the work

in a fashion requiring copyright permission, other than the making of an exact copy. The resulting work is called a "modified version" of the earlier work or a work "based on" the earlier work.

A "covered work" means either the unmodified Program or a work based on the Program.

To "propagate" a work means to do anything with it that, without permission, would make you directly or secondarily liable for infringement under applicable copyright law, except executing it on a computer or modifying a private copy. Propagation includes copying, distribution (with or without modification), making available to the public, and in some countries other activities as well.

To "convey" a work means any kind of propagation that enables other parties to make or receive copies. Mere interaction with a user through a computer network, with no transfer of a copy, is not conveying.

An interactive user interface displays "Appropriate Legal Notices" to the extent that it includes a convenient and prominently visible feature that (1) displays an appropriate copyright notice, and (2) tells the user that there is no warranty for the work (except to the extent that warranties are provided), that licensees may convey the work under this License, and how to view a copy of this License. If the interface presents a list of user commands or options, such as a menu, a prominent item in the list meets this criterion.

1. Source Code.

The "source code" for a work means the preferred form of the work for making modifications to it. "Object code" means any non-source form of a work.

A "Standard Interface" means an interface that either is an official standard defined by a recognized standards body, or, in the case of interfaces specified for a particular programming language, one that is widely used among developers working in that language.

The "System Libraries" of an executable work include anything, other than the work as a whole, that (a) is included in the normal form of packaging a Major Component, but which is not part of that Major Component, and (b) serves only to enable use of the work with that Major Component, or to implement a Standard Interface for which an implementation is available to the public in source code form. A

"Major Component", in this context, means a major essential component (kernel, window system, and so on) of the specific operating system (if any) on which the executable work runs, or a compiler used to produce the work, or an object code interpreter used to run it.

The "Corresponding Source" for a work in object code form means all the source code needed to generate, install, and (for an executable work) run the object code and to modify the work, including scripts to control those activities. However, it does not include the work's System Libraries, or general-purpose tools or generally available free programs which are used unmodified in performing those activities but which are not part of the work. For example, Corresponding Source includes interface definition files associated with source files for the work, and the source code for shared libraries and dynamically linked subprograms that the work is specifically designed to require, such as by intimate data communication or control flow between those subprograms and other parts of the work.

The Corresponding Source need not include anything that users can regenerate automatically from other parts of the Corresponding Source.

The Corresponding Source for a work in source code form is that same work.

2. Basic Permissions.

All rights granted under this License are granted for the term of copyright on the Program, and are irrevocable provided the stated conditions are met. This License explicitly affirms your unlimited permission to run the unmodified Program. The output from running a covered work is covered by this License only if the output, given its content, constitutes a covered work. This License acknowledges your rights of fair use or other equivalent, as provided by copyright law.

You may make, run and propagate covered works that you do not convey, without conditions so long as your license otherwise remains in force. You may convey covered works to others for the sole purpose of having them make modifications exclusively for you, or provide you with facilities for running those works, provided that you comply with the terms of this License in conveying all material for which you do not control copyright. Those thus making or running the covered works

for you must do so exclusively on your behalf, under your direction and control, on terms that prohibit them from making any copies of your copyrighted material outside their relationship with you.

Conveying under any other circumstances is permitted solely under the conditions stated below. Sublicensing is not allowed; section 10 makes it unnecessary.

3. Protecting Users' Legal Rights From Anti-Circumvention Law.

No covered work shall be deemed part of an effective technological measure under any applicable law fulfilling obligations under article 11 of the WIPO copyright treaty adopted on 20 December 1996, or similar laws prohibiting or restricting circumvention of such measures.

When you convey a covered work, you waive any legal power to forbid circumvention of technological measures to the extent such circumvention is effected by exercising rights under this License with respect to the covered work, and you disclaim any intention to limit operation or modification of the work as a means of enforcing, against the work's users, your or third parties' legal rights to forbid circumvention of technological measures.

4. Conveying Verbatim Copies.

You may convey verbatim copies of the Program's source code as you receive it, in any medium, provided that you conspicuously and appropriately publish on each copy an appropriate copyright notice; keep intact all notices stating that this License and any non-permissive terms added in accord with section 7 apply to the code; keep intact all notices of the absence of any warranty; and give all recipients a copy of this License along with the Program.

You may charge any price or no price for each copy that you convey, and you may offer support or warranty protection for a fee.

5. Conveying Modified Source Versions.

You may convey a work based on the Program, or the modifications to produce it from the Program, in the form of source code under the terms of section 4, provided that you also meet all of these conditions:

a) The work must carry prominent notices stating that you modified it, and giving a relevant date.

b) The work must carry prominent notices stating that it is

released under this License and any conditions added under section 7. This requirement modifies the requirement in section 4 to "keep intact all notices".

c) You must license the entire work, as a whole, under this License to anyone who comes into possession of a copy. This License will therefore apply, along with any applicable section 7 additional terms, to the whole of the work, and all its parts, regardless of how they are packaged. This License gives no permission to license the work in any other way, but it does not invalidate such permission if you have separately received it.

d) If the work has interactive user interfaces, each must display Appropriate Legal Notices; however, if the Program has interactive interfaces that do not display Appropriate Legal Notices, your work need not make them do so.

A compilation of a covered work with other separate and independent works, which are not by their nature extensions of the covered work, and which are not combined with it such as to form a larger program, in or on a volume of a storage or distribution medium, is called an "aggregate" if the compilation and its resulting copyright are not used to limit the access or legal rights of the compilation's users beyond what the individual works permit. Inclusion of a covered work in an aggregate does not cause this License to apply to the other parts of the aggregate.

6. Conveying Non-Source Forms.

You may convey a covered work in object code form under the terms of sections 4 and 5, provided that you also convey the machine-readable Corresponding Source under the terms of this License, in one of these ways:

a) Convey the object code in, or embodied in, a physical product (including a physical distribution medium), accompanied by the Corresponding Source fixed on a durable physical medium customarily used for software interchange.

b) Convey the object code in, or embodied in, a physical product (including a physical distribution medium), accompanied by a written offer, valid for at least three years and valid for as long as you offer spare parts or customer support for that product

model, to give anyone who possesses the object code either (1) a copy of the Corresponding Source for all the software in the product that is covered by this License, on a durable physical medium customarily used for software interchange, for a price no more than your reasonable cost of physically performing this conveying of source, or (2) access to copy the Corresponding Source from a network server at no charge.

c) Convey individual copies of the object code with a copy of the written offer to provide the Corresponding Source. This alternative is allowed only occasionally and noncommercially, and only if you received the object code with such an offer, in accord with subsection 6b.

d) Convey the object code by offering access from a designated place (gratis or for a charge), and offer equivalent access to the Corresponding Source in the same way through the same place at no further charge. You need not require recipients to copy the Corresponding Source along with the object code. If the place to copy the object code is a network server, the Corresponding Source may be on a different server (operated by you or a third party) that supports equivalent copying facilities, provided you maintain clear directions next to the object code saying where to find the Corresponding Source. Regardless of what server hosts the Corresponding Source, you remain obligated to ensure that it is available for as long as needed to satisfy these requirements.

e) Convey the object code using peer-to-peer transmission, provided you inform other peers where the object code and Corresponding Source of the work are being offered to the general public at no charge under subsection 6d.

A separable portion of the object code, whose source code is excluded from the Corresponding Source as a System Library, need not be included in conveying the object code work.

A "User Product" is either (1) a "consumer product", which means any tangible personal property which is normally used for personal, family, or household purposes, or (2) anything designed or sold for incorporation into a dwelling. In determining whether a product is a consumer product, doubtful cases shall be resolved in favor of coverage. For a particular

product received by a particular user, "normally used" refers to a typical or common use of that class of product, regardless of the status of the particular user or of the way in which the particular user actually uses, or expects or is expected to use, the product. A product is a consumer product regardless of whether the product has substantial commercial, industrial or non-consumer uses, unless such uses represent the only significant mode of use of the product.

"Installation Information" for a User Product means any methods, procedures, authorization keys, or other information required to install and execute modified versions of a covered work in that User Product from a modified version of its Corresponding Source. The information must suffice to ensure that the continued functioning of the modified object code is in no case prevented or interfered with solely because modification has been made.

If you convey an object code work under this section in, or with, or specifically for use in, a User Product, and the conveying occurs as part of a transaction in which the right of possession and use of the User Product is transferred to the recipient in perpetuity or for a fixed term (regardless of how the transaction is characterized), the Corresponding Source conveyed under this section must be accompanied by the Installation Information. But this requirement does not apply if neither you nor any third party retains the ability to install modified object code on the User Product (for example, the work has been installed in ROM).

The requirement to provide Installation Information does not include a requirement to continue to provide support service, warranty, or updates for a work that has been modified or installed by the recipient, or for the User Product in which it has been modified or installed. Access to a network may be denied when the modification itself materially and adversely affects the operation of the network or violates the rules and protocols for communication across the network.

Corresponding Source conveyed, and Installation Information provided, in accord with this section must be in a format that is publicly documented (and with an implementation available to the public in source code form), and must require no special password or key for unpacking, reading or copying.

7. Additional Terms.

"Additional permissions" are terms that supplement the terms of this License by making exceptions from one or more of its conditions. Additional permissions that are applicable to the entire Program shall be treated as though they were included in this License, to the extent that they are valid under applicable law. If additional permissions apply only to part of the Program, that part may be used separately under those permissions, but the entire Program remains governed by this License without regard to the additional permissions.

When you convey a copy of a covered work, you may at your option remove any additional permissions from that copy, or from any part of it. (Additional permissions may be written to require their own removal in certain cases when you modify the work.) You may place additional permissions on material, added by you to a covered work, for which you have or can give appropriate copyright permission.

Notwithstanding any other provision of this License, for material you add to a covered work, you may (if authorized by the copyright holders of that material) supplement the terms of this License with terms:

a) Disclaiming warranty or limiting liability differently from the terms of sections 15 and 16 of this License; or

b) Requiring preservation of specified reasonable legal notices or author attributions in that material or in the Appropriate Legal Notices displayed by works containing it; or

c) Prohibiting misrepresentation of the origin of that material, or requiring that modified versions of such material be marked in reasonable ways as different from the original version; or

d) Limiting the use for publicity purposes of names of licensors or authors of the material; or

e) Declining to grant rights under trademark law for use of some trade names, trademarks, or service marks; or

f) Requiring indemnification of licensors and authors of that material by anyone who conveys the material (or modified versions of it) with contractual assumptions of liability to the recipient, for any liability that these contractual assumptions directly impose on those licensors and authors.

All other non-permissive additional terms are considered "further

restrictions" within the meaning of section 10. If the Program as you received it, or any part of it, contains a notice stating that it is governed by this License along with a term that is a further restriction, you may remove that term. If a license document contains a further restriction but permits relicensing or conveying under this License, you may add to a covered work material governed by the terms of that license document, provided that the further restriction does not survive such relicensing or conveying.

If you add terms to a covered work in accord with this section, you must place, in the relevant source files, a statement of the additional terms that apply to those files, or a notice indicating where to find the applicable terms.

Additional terms, permissive or non-permissive, may be stated in the form of a separately written license, or stated as exceptions; the above requirements apply either way.

8. Termination.

You may not propagate or modify a covered work except as expressly provided under this License. Any attempt otherwise to propagate or modify it is void, and will automatically terminate your rights under this License (including any patent licenses granted under the third paragraph of section 11).

However, if you cease all violation of this License, then your license from a particular copyright holder is reinstated (a) provisionally, unless and until the copyright holder explicitly and finally terminates your license, and (b) permanently, if the copyright holder fails to notify you of the violation by some reasonable means prior to 60 days after the cessation.

Moreover, your license from a particular copyright holder is reinstated permanently if the copyright holder notifies you of the violation by some reasonable means, this is the first time you have received notice of violation of this License (for any work) from that copyright holder, and you cure the violation prior to 30 days after your receipt of the notice.

Termination of your rights under this section does not terminate the licenses of parties who have received copies or rights from you under this License. If your rights have been terminated and not permanently

reinstated, you do not qualify to receive new licenses for the same material under section 10.

9. Acceptance Not Required for Having Copies.

You are not required to accept this License in order to receive or run a copy of the Program. Ancillary propagation of a covered work occurring solely as a consequence of using peer-to-peer transmission to receive a copy likewise does not require acceptance. However, nothing other than this License grants you permission to propagate or modify any covered work. These actions infringe copyright if you do not accept this License. Therefore, by modifying or propagating a covered work, you indicate your acceptance of this License to do so.

10. Automatic Licensing of Downstream Recipients.

Each time you convey a covered work, the recipient automatically receives a license from the original licensors, to run, modify and propagate that work, subject to this License. You are not responsible for enforcing compliance by third parties with this License.

An "entity transaction" is a transaction transferring control of an organization, or substantially all assets of one, or subdividing an organization, or merging organizations. If propagation of a covered work results from an entity transaction, each party to that transaction who receives a copy of the work also receives whatever licenses to the work the party's predecessor in interest had or could give under the previous paragraph, plus a right to possession of the Corresponding Source of the work from the predecessor in interest, if the predecessor has it or can get it with reasonable efforts.

You may not impose any further restrictions on the exercise of the rights granted or affirmed under this License. For example, you may not impose a license fee, royalty, or other charge for exercise of rights granted under this License, and you may not initiate litigation (including a cross-claim or counterclaim in a lawsuit) alleging that any patent claim is infringed by making, using, selling, offering for sale, or importing the Program or any portion of it.

11. Patents.

A "contributor" is a copyright holder who authorizes use under this License of the Program or a work on which the Program is based. The work thus licensed is called the contributor's "contributor version".

A contributor's "essential patent claims" are all patent claims owned or controlled by the contributor, whether already acquired or hereafter acquired, that would be infringed by some manner, permitted by this License, of making, using, or selling its contributor version, but do not include claims that would be infringed only as a consequence of further modification of the contributor version. For purposes of this definition, "control" includes the right to grant patent sublicenses in a manner consistent with the requirements of this License.

Each contributor grants you a non-exclusive, worldwide, royalty-free patent license under the contributor's essential patent claims, to make, use, sell, offer for sale, import and otherwise run, modify and propagate the contents of its contributor version.

In the following three paragraphs, a "patent license" is any express agreement or commitment, however denominated, not to enforce a patent (such as an express permission to practice a patent or covenant not to sue for patent infringement). To "grant" such a patent license to a party means to make such an agreement or commitment not to enforce a patent against the party.

If you convey a covered work, knowingly relying on a patent license, and the Corresponding Source of the work is not available for anyone to copy, free of charge and under the terms of this License, through a publicly available network server or other readily accessible means, then you must either (1) cause the Corresponding Source to be so available, or (2) arrange to deprive yourself of the benefit of the patent license for this particular work, or (3) arrange, in a manner consistent with the requirements of this License, to extend the patent license to downstream recipients. "Knowingly relying" means you have actual knowledge that, but for the patent license, your conveying the covered work in a country, or your recipient's use of the covered work in a country, would infringe one or more identifiable patents in that country that you have reason to believe are valid.

If, pursuant to or in connection with a single transaction or arrangement, you convey, or propagate by procuring conveyance of, a covered work, and grant a patent license to some of the parties receiving the covered work authorizing them to use, propagate, modify

or convey a specific copy of the covered work, then the patent license you grant is automatically extended to all recipients of the covered work and works based on it.

A patent license is "discriminatory" if it does not include within the scope of its coverage, prohibits the exercise of, or is conditioned on the non-exercise of one or more of the rights that are specifically granted under this License. You may not convey a covered work if you are a party to an arrangement with a third party that is in the business of distributing software, under which you make payment to the third party based on the extent of your activity of conveying the work, and under which the third party grants, to any of the parties who would receive the covered work from you, a discriminatory patent license (a) in connection with copies of the covered work conveyed by you (or copies made from those copies), or (b) primarily for and in connection with specific products or compilations that contain the covered work, unless you entered into that arrangement, or that patent license was granted, prior to 28 March 2007.

Nothing in this License shall be construed as excluding or limiting any implied license or other defenses to infringement that may otherwise be available to you under applicable patent law.

12. No Surrender of Others' Freedom.

If conditions are imposed on you (whether by court order, agreement or otherwise) that contradict the conditions of this License, they do not excuse you from the conditions of this License. If you cannot convey a covered work so as to satisfy simultaneously your obligations under this License and any other pertinent obligations, then as a consequence you may not convey it at all. For example, if you agree to terms that obligate you to collect a royalty for further conveying from those to whom you convey the Program, the only way you could satisfy both those terms and this License would be to refrain entirely from conveying the Program.

13. Use with the GNU Affero General Public License.

Notwithstanding any other provision of this License, you have permission to link or combine any covered work with a work licensed under version 3 of the GNU Affero General Public License into a single combined work, and to convey the resulting work. The terms of this License will continue to apply to the part which is the covered work,

but the special requirements of the GNU Affero General Public License, section 13, concerning interaction through a network will apply to the combination as such.

14. Revised Versions of this License.

The Free Software Foundation may publish revised and/or new versions of the GNU General Public License from time to time. Such new versions will be similar in spirit to the present version, but may differ in detail to address new problems or concerns.

Each version is given a distinguishing version number. If the Program specifies that a certain numbered version of the GNU General Public License "or any later version" applies to it, you have the option of following the terms and conditions either of that numbered version or of any later version published by the Free Software Foundation. If the Program does not specify a version number of the GNU General Public License, you may choose any version ever published by the Free Software Foundation.

If the Program specifies that a proxy can decide which future versions of the GNU General Public License can be used, that proxy's public statement of acceptance of a version permanently authorizes you to choose that version for the Program.

Later license versions may give you additional or different permissions. However, no additional obligations are imposed on any author or copyright holder as a result of your choosing to follow a later version.

15. Disclaimer of Warranty.

THERE IS NO WARRANTY FOR THE PROGRAM, TO THE EXTENT PERMITTED BY APPLICABLE LAW. EXCEPT WHEN OTHERWISE STATED IN WRITING THE COPYRIGHT HOLDERS AND/OR OTHER PARTIES PROVIDE THE PROGRAM "AS IS" WITHOUT WARRANTY OF ANY KIND, EITHER EXPRESSED OR IMPLIED, INCLUDING, BUT NOT LIMITED TO, THE IMPLIED WARRANTIES OF MERCHANTABILITY AND FITNESS FOR A PARTICULAR PURPOSE. THE ENTIRE RISK AS TO THE QUALITY AND PERFORMANCE OF THE PROGRAM IS WITH YOU. SHOULD THE PROGRAM PROVE DEFECTIVE, YOU ASSUME THE COST OF ALL NECESSARY SERVICING, REPAIR OR CORRECTION.

16. Limitation of Liability.

IN NO EVENT UNLESS REQUIRED BY APPLICABLE LAW OR AGREED TO IN WRITING WILL ANY COPYRIGHT HOLDER, OR ANY OTHER PARTY WHO MODIFIES AND/OR CONVEYS

THE PROGRAM AS PERMITTED ABOVE, BE LIABLE TO YOU FOR DAMAGES, INCLUDING ANY GENERAL, SPECIAL, INCIDENTAL OR CONSEQUENTIAL DAMAGES ARISING OUT OF THE USE OR INABILITY TO USE THE PROGRAM (INCLUDING BUT NOT LIMITED TO LOSS OF DATA OR DATA BEING RENDERED INACCURATE OR LOSSES SUSTAINED BY YOU OR THIRD PARTIES OR A FAILURE OF THE PROGRAM TO OPERATE WITH ANY OTHER PROGRAMS), EVEN IF SUCH HOLDER OR OTHER PARTY HAS BEEN ADVISED OF THE POSSIBILITY OF SUCH DAMAGES.

17. Interpretation of Sections 15 and 16.

If the disclaimer of warranty and limitation of liability provided above cannot be given local legal effect according to their terms, reviewing courts shall apply local law that most closely approximates an absolute waiver of all civil liability in connection with the Program, unless a warranty or assumption of liability accompanies a copy of the Program in return for a fee.

END OF TERMS AND CONDITIONS

How to Apply These Terms to Your New Programs

If you develop a new program, and you want it to be of the greatest possible use to the public, the best way to achieve this is to make it free software which everyone can redistribute and change under these terms.

To do so, attach the following notices to the program. It is safest to attach them to the start of each source file to most effectively state the exclusion of warranty; and each file should have at least the "copyright" line and a pointer to where the full notice is found.

<one line to give the program’s name and a brief idea of what it does.>
Copyright (C) <year> <name of author>
This program is free software: you can redistribute it and/or modify it under the terms of the GNU General Public License as published by the Free Software Foundation, either version 3 of the License, or (at your option) any later version.
This program is distributed in the hope that it will be useful, but WITHOUT ANY WARRANTY; without even the implied warranty of MERCHANTABILITY or FITNESS FOR A PARTICULAR PURPOSE. See the GNU General Public License for more details.
You should have received a copy of the GNU General Public License along with this program. If not, see <http://www.gnu.org/licenses/>.

Also add information on how to contact you by electronic and paper mail.

```
  If the program does terminal interaction, make it output a short
notice like this when it starts in an interactive mode:
    <program>  Copyright (C) <year>  <name of author>
    This program comes with ABSOLUTELY NO WARRANTY; for details type ‘show w’.
    This is free software, and you are welcome to redistribute it
    under certain conditions; type ‘show c’ for details.
The hypothetical commands ‘show w’ and ‘show c’ should show the appropriate
parts of the General Public License.  Of course, your program’s commands
might be different; for a GUI interface, you would use an "about box".
  You should also get your employer (if you work as a programmer) or school,
if any, to sign a "copyright disclaimer" for the program, if necessary.
For more information on this, and how to apply and follow the GNU GPL, see
<http://www.gnu.org/licenses/>.
  The GNU General Public License does not permit incorporating your program
into proprietary programs.  If your program is a subroutine library, you
may consider it more useful to permit linking proprietary applications with
the library.  If this is what you want to do, use the GNU Lesser General
Public License instead of this License.  But first, please read
<http://www.gnu.org/philosophy/why-not-lgpl.html>.
```

Chapter 4

Index

Index

A

active flag
  setting, 63
Affymetrix, 90
aliases
  markers, 39
  members, 41
arguments, 103

D

database
  initialization, 20
dbvor active, 63, 109
dbvor allelemap, 111
dbvor creatdb, 191
dbvor create_experiment, 115
dbvor createdb, 20, 113
dbvor delete_experiment, 116
dbvor deleteblockallsamples, 135
dbvor deleteblockMember, 136
dbvor deleteblocksample, 137
dbvor deletesnpblockmap, 138
dbvor gmimarker, 36, 117
dbvor gmimarkeralias, 118
dbvor markeralias, 39, 119
dbvor markerinfo, 35, 120
dbvor member, 24, 123
  adding data, 39
dbvor memberalias, 42
dbvor missingmarkerinfo, 37, 122
dbvor newmarker, 126
dbvor plink, 95, 127
dbvor plinksample, 129
dbvor sample, 130
dbvor set_experiment_date, 132
dbvor showsamples, 139
dbvor trait, 29, 133
.dbvorrc configuration file, 21

E

export data, 33

F

Filtering, 71

G

geno, 31, 140
genoa, 91, 152
genoi, 85, 145
genok, 150
genote, 168
genout, 33, 61, 158
  selection, 65
  statistics, 76

I

Illumina, 85
input
  genome-wide marker data, 77
  map data, 35
  marker data, 31
  pedigree data, 24
  PLINK family data, 95

PLINK genotype data, 98
PLINK marker data, 97
trait data, 29
installation, 14
prerequisites, 14
SQLPATH, 15

M

map data
input, 35
marker data
input genome-wide data, 77
input targeted data, 31
MySQL mini-tutorial, 191

N

notes, 168

P

PLINK format data
reading in, 94

T

trait data
input, 29

Appendix A

MySQL mini-tutorial

Let us discuss how to use MySQL to look at our data. What's the point of a powerful tool if you can not dig yourself into a really big hole. Be careful. I will assume that you followed section 2 to create the database, **exampledb**, and the user, **example**. You also populated the database with several tables. If you want to look more deeply at how this was done, look at the **dbvor creatdb** code, look for the expression "cmd =" in the code and try to make sense of the string after the "=". (You might want to use Google or a MySQL manual.)

The way you get a MySQL shell is to type:

mysql -u example -p -host localhost exampledb

This will ask for your secret password and will log the user **example** into the database **exampledb**. As I said before you can use Google to look up the details. At this point, you will get a prompt "mysql> ". Commands you type are followed by a ";".

Let us type:

show databases;

This request will show you a listing of the databases you have created, probably just **exampledb** plus a special database called **information_schema**. (information_schema contains information about the database itself and is way beyond the scope of this document.)

Now type:

show tables;

This will print out the names of all of the tables structures that were defined for your current database. A few of the tables are: markers, marker_info and members. These are used to store data that we have discussed in this tutorial. Of course, right after the database is created the tables are empty. To see the field definitions for the table, **members**, type:

describe members;

You will see

```
+------------+------------------+------+-----+-------------------+-----------------------------+
| Field      | Type             | Null | Key | Default           | Extra                       |
+------------+------------------+------+-----+-------------------+-----------------------------+
| id         | int(10) unsigned | NO   | PRI | NULL              | auto_increment              |
| projectid  | int(10) unsigned | NO   | MUL | NULL              |                             |
| pedigree   | varchar(30)      | NO   |     | NULL              |                             |
| person     | varchar(30)      | NO   |     | NULL              |                             |
| father     | varchar(30)      | NO   |     | 0                 |                             |
| mother     | varchar(30)      | NO   |     | 0                 |                             |
| gender     | int(11)          | NO   |     | 0                 |                             |
| type       | char(1)          | NO   |     | U                 |                             |
| variant    | mediumint(9)     | NO   |     | 1                 |                             |
| active     | tinyint(4)       | NO   |     | 1                 |                             |
| time_stamp | timestamp        | NO   |     | CURRENT_TIMESTAMP | on update CURRENT_TIMESTAMP |
+------------+------------------+------+-----+-------------------+-----------------------------+
11 rows in set (0.05 sec)
```

The **Field** column shows the obvious fields used to store the pedigree data (section 2.4.1) plus a few more needed fields. You can look up the meaning in the other columns if you are curious.

If you want to see the actual data in the **members** table, you would type:

select * from members;

```
+----+-----------+----------+--------+--------+--------+--------+------+---------+--------+---------------------+
| id | projectid | pedigree | person | father | mother | gender | type | variant | active | time_stamp          |
+----+-----------+----------+--------+--------+--------+--------+------+---------+--------+---------------------+
|  1 |         1 | 1        | 1      | 0      | 0      |      2 | U    |       1 |      1 | 2011-09-23 13:48:49 |
|  2 |         1 | 1        | 2      | 0      | 0      |      1 | U    |       1 |      1 | 2011-09-23 13:48:49 |
```

3	1	1	3	1	2	1	U	1	1	2011-09-23 13:48:49
4	1	1	4	1	2	2	U	1	1	2011-09-23 13:48:49
5	1	2	101	0	0	2	U	1	1	2011-09-23 13:48:49
6	1	2	201	0	0	1	U	1	1	2011-09-23 13:48:49
7	1	2	301	101	201	1	U	1	1	2011-09-23 13:48:49
8	1	2	401	101	201	2	U	1	1	2011-09-23 13:48:49
9	1	2	501	101	201	2	U	1	1	2011-09-23 13:48:49
10	1	3	1	0	0	2	U	1	1	2011-09-23 15:35:31
11	1	3	2	0	0	1	U	1	1	2011-09-23 15:35:31
12	1	3	3	1	2	1	U	1	1	2011-09-23 15:35:31
13	1	3	4	1	2	2	U	1	1	2011-09-23 15:35:31

13 rows in set (0.00 sec)

You can look at this table and verify that the people have the correct mother and father according to the data that was input. In general, the **select** command is very powerful and allows you to select the columns you want displayed as well as allowing you to filter over the columns to select the rows that you want. Let us look at the pedigree, person, and gender of the non founder (father and mother specified) of pedigree 2. (I will explain the id column later.) You type:

select pedigree, person, gender, id from members where pedigree = 2 and father != 0 and mother != 0;

and get an answer of:

pedigree	person	gender	id
2	301	1	7
2	401	2	8
2	501	2	9

3 rows in set (0.00 sec)

showing the gender values we are interested in. We have constructed the **id** column of the table so it is guaranteed to be unique. We can use this **id** column to safely change the values in a column for only one row. Suppose we need to change the gender for pedigree 2 person 501 to 1. In our case, pedigree 2 person 501 is unique, but sometimes it is not as easy to find a filter that will easily identify the row you want to change. Here **id** value of 9 will find the row we want. The command to change the gender is:

update members set gender = 1 where id = 9;

(Note**: update members set gender = 1 where pedigree = 2 and person = 501;**

is equivalent. But **update members set gender = 1 where pedigree = 2;** would be very wrong.)

NOTE: Directly changing the database like this is a very bad idea since no record is left of how/when/why the change was made. And the database will not correspond to the data in your flat files.

An important features of a database is that changes are not visible to other users of the database until the "work is officially finished". So another user of the computer viewing the same database just after the above change will still see the gender 2 not 1. You must type the command:

commit;

for other users of the database to see the change.

Of course, had you made a mistake, say typed:

update members set gender = 1 where pedigree = 2;

by accident. No one knows it until the **commit** command is run. And if you instead type:

rollback;

the change is undone. You will see the database restored to what it was before the bad update was performed and no one else on the computer will ever see the mistake.

Finally, typing the command:

quit;

will exit the mysql shell.
