# Supplementary material for: dbVOR: a database system for importing pedigree, phenotype and genotype data and exporting selected subsets
Source: BMC Bioinformatics. 2015 Mar 18;16(1):91. doi: 10.1186/s12859-015-0505-4 (PMC4407391; doi:10.1186/s12859-015-0505-4)
Supplement: Additional file 1 — Contains the entire dbVOR package, version 1.11. [file 12859_2015_505_MOESM1_ESM.zip › 1652824117132674_add1.tgz!/dbvor.1.11/doc/Book/dbVORtutorial.navigation.html]

xml version="1.0" encoding="UTF-8"?


Converted document


Chapter 1: Introduction

1.1: Organization of this Document

1.2: Motivation

1.3: Prerequisites

1.4: Download and Install dbVOR

1.4.1: Install prerequisites

1.4.2: Download and decompress dbVOR

1.4.3: Installation of dbVOR as a Python application

1.4.4: Note and record the path to the ’sql’ folder for later use

1.4.5: Create a dbVOR database

1.4.6: Add information into the database

1.5: Citing dbVOR

1.6: Support, bug reports, and feedback

1.6.1: dbVOR Google Group

1.6.2: Bug reports

1.7: Contact information

1.8: Acknowledgments

Chapter 2: Tutorial

2.1: Introduction

2.2: Overview of dbvor commands

2.3: Creating a database

2.3.1: Using the .dbvorrc configuration file

2.4: Populating the database

2.4.1: Adding Pedigree Data using dbvor member

2.4.2: Adding Trait Data using dbvor trait

2.4.3: Adding targeted marker data using geno

2.4.4: Retrieving and exporting data using genout

2.4.5: Adding Marker Position Data using GMI

2.4.6: More GMI Marker Position Data

2.4.7: Manually adding Marker Position Data and Aliasing Data

2.5: Adding Additional Data

2.5.1: Adding more Pedigree Data

2.5.2: Adding additional marker data using geno

2.5.3: Merged Markers / Marker Aliases

2.5.4: Member Aliases

2.5.5: Another trait

2.5.6: Merging Experiments

2.5.6.1: Dry run

2.5.6.2: Verify Run

2.5.6.3: Verbose Verify Run

2.5.6.4: Commit

2.5.6.5: More Pesky Details

2.5.7: Problems during Merging Markers

2.5.7.1: Lets look at a block of output.

2.5.7.2: Using the concordance matrix

2.5.7.3: Resolvable Disagreements

2.5.7.4: Unresolved Disagreements

2.5.7.5: Final commit

2.5.8: Coda

2.5.8.1: Restricting Pedigree and Persons

2.5.8.2: Splitting up the configuration file

2.5.8.3: Changing the log file name

2.5.8.4: Less log output

2.6: genout: Data Extraction

2.6.1: Data Summary

2.6.2: Controlling Output Format

2.6.3: Resolving Data Conflicts

2.6.3.1: Setting active flag

2.6.4: A Selection Example

2.6.5: Selector

2.6.5.1: Select by marker, chromosome, etc.

2.6.5.2: Select by member

2.6.5.3: Select by trait

2.6.5.4: Select by ...

2.6.6: Filtering Experiment Data

2.6.7: Further Output Customization

2.6.8: Modified operations for

2.6.9: genout Statistics

2.7: Adding genome-wide data

2.7.1: Genome-wide HapMap Example

2.7.1.1: Family Data

2.7.1.2: Plate Data

2.7.1.3: Hapmap Data

2.7.1.3.1: checksum and size

2.7.1.3.2: unzip

2.7.1.3.3: Summary

2.7.2: Genome-wide Prerequisites

2.7.2.1: Create a database

2.7.2.2: Add Members for the HapMap Example

2.7.3: Genome-wide Samples

2.7.3.1: Create Samples for the HapMap Example

2.7.3.2: Add Samples for the HapMap Example

2.7.4: genoi: Simple genome-wide data (Illumina)

2.7.4.1: Simple GWAS Markers

2.7.4.1.1: Add Markers for the HapMap Example

2.7.4.2: Simple GWAS Data

2.7.4.2.1: Add Data for the HapMap Example

2.7.5: genoa: Genome-wide Data (Affymetrix)

2.7.5.1: GWAS Samples Revisited — Affymetrix samples

2.7.5.2: Affymetrix GWAS Markers

2.7.5.3: Affymetrix GWAS Data

2.7.6: genok: PLINK binary (bed) data files

2.7.6.1: Processing Family Data using dbvor plink

2.7.6.2: PLINK genome-wide Markers

2.7.6.3: PLINK bed genome-wide genotype data

2.7.6.4: Adding additional genome-wide data

2.7.7: Deleting and reentering genome-wide data

2.7.7.1: DeleteBlockSample

2.7.7.2: DeleteBlockMember

2.7.7.3: DeleteBlockAllSamples

2.7.7.4: DeleteSnpBlockMap

2.7.7.5: ShowSamples

Chapter 3: Manual

3.1: Introduction

3.2: General Arguments

3.2.1: Required Arguments

3.2.2: Common Arguments

3.3: dbVOR Commands

3.3.1: dbvor active

3.3.1.1: NAME

3.3.1.2: SYNOPSIS

3.3.1.3: ARGUMENTS

3.3.2: dbvor allelemap

3.3.2.1: NAME

3.3.2.2: SYNOPSIS

3.3.2.3: ARGUMENTS

3.3.3: dbvor createdb

3.3.3.1: NAME

3.3.3.2: SYNOPSIS

3.3.3.3: ARGUMENTS

3.3.3.4: USAGE

3.3.4: dbvor create\_experiment

3.3.4.1: NAME

3.3.4.2: SYNOPSIS

3.3.4.3: ARGUMENTS

3.3.5: dbvor delete\_experiment

3.3.5.1: NAME

3.3.5.2: SYNOPSIS

3.3.5.3: ARGUMENTS

3.3.6: dbvor gmimarker

3.3.6.1: NAME

3.3.6.2: SYNOPSIS

3.3.6.3: ARGUMENTS

3.3.7: dbvor gmimarkeralias

3.3.7.1: NAME

3.3.7.2: SYNOPSIS

3.3.7.3: ARGUMENTS

3.3.8: dbvor markeralias

3.3.8.1: NAME

3.3.8.2: SYNOPSIS

3.3.8.3: ARGUMENTS

3.3.9: dbvor markerinfo

3.3.9.1: NAME

3.3.9.2: SYNOPSIS

3.3.9.3: ARGUMENTS

3.3.10: dbvor missingmarkerinfo

3.3.10.1: NAME

3.3.10.2: SYNOPSIS

3.3.10.3: ARGUMENTS

3.3.11: dbvor member

3.3.11.1: NAME

3.3.11.2: SYNOPSIS

3.3.11.3: ARGUMENTS

3.3.11.4: USAGE

3.3.12: dbvor memberalias

3.3.12.1: NAME

3.3.12.2: SYNOPSIS

3.3.12.3: ARGUMENTS

3.3.13: dbvor newmarker

3.3.13.1: NAME

3.3.13.2: SYNOPSIS

3.3.13.3: ARGUMENTS

3.3.14: dbvor plink

3.3.14.1: NAME

3.3.14.2: SYNOPSIS

3.3.14.3: ARGUMENTS

3.3.14.4: ADDITIONAL TRAIT ARGUMENTS

3.3.15: dbvor plinksample

3.3.15.1: NAME

3.3.15.2: SYNOPSIS

3.3.15.3: ARGUMENTS

3.3.16: dbvor sample

3.3.16.1: NAME

3.3.16.2: SYNOPSIS

3.3.16.3: ARGUMENTS

3.3.16.3.1: sample file format

3.3.17: dbvor set\_experiment\_date

3.3.17.1: NAME

3.3.17.2: SYNOPSIS

3.3.17.3: ARGUMENTS

3.3.18: dbvor trait

3.3.18.1: NAME

3.3.18.2: SYNOPSIS

3.3.18.3: ARGUMENTS

3.3.19: dbvor DeleteBlockAllSamples

3.3.19.1: NAME

3.3.19.2: SYNOPSIS

3.3.19.3: ARGUMENTS

3.3.20: dbvor DeleteBlockMember

3.3.20.1: NAME

3.3.20.2: SYNOPSIS

3.3.20.3: ARGUMENTS

3.3.21: dbvor DeleteBlockSample

3.3.21.1: NAME

3.3.21.2: SYNOPSIS

3.3.21.3: ARGUMENTS

3.3.22: dbvor DeleteSnpBlockMap

3.3.22.1: NAME

3.3.22.2: SYNOPSIS

3.3.22.3: ARGUMENTS

3.3.23: dbvor ShowSamples

3.3.23.1: NAME

3.3.23.2: SYNOPSIS

3.3.23.3: ARGUMENTS

3.4: Genotype Entry

3.4.1: Geno

3.4.1.1: NAME

3.4.1.2: SYNOPSIS

3.4.1.3: ARGUMENTS

3.4.1.4: Deprecated Arguments

3.4.2: Genoi

3.4.2.1: NAME

3.4.2.2: SYNOPSIS

3.4.2.3: ARGUMENTS

3.4.2.3.1: make-snps marker data

3.4.2.3.2: genotype data

3.4.3: Genok

3.4.3.1: NAME

3.4.3.2: SYNOPSIS

3.4.3.3: ARGUMENTS

3.4.3.3.1: make-snps marker data

3.4.3.3.2: genotype data

3.4.4: Genoa

3.4.4.1

3.4.4.2: SYNOPSIS

3.4.4.3: ARGUMENTS

3.4.4.3.1: make-snps marker data

3.4.4.3.2: genotype data

3.5: Data extraction using genout

3.5.1: NAME

3.5.2: SYNOPSIS

3.5.3: ARGUMENTS

3.5.3.1: Formatting Arguments

3.5.3.2: Type values

3.5.3.3: Heterozygote values

3.5.3.4: Filters

3.5.3.4.1: Select by marker, chromosome, etc.

3.5.3.4.2: Select by member

3.5.3.4.3: Select by trait

3.5.3.4.4: Select by and

3.5.3.4.5: Filtering Experiment Data

3.6: Miscellaneous Commands

3.6.1: Genote

3.6.1.1: NAME

3.6.1.2: SYNOPSIS

3.6.1.3: ARGUMENTS

3.6.1.3.1: Insert mode

3.6.1.3.2: Automatic insertion mode

3.6.1.3.3: Fetch mode

3.6.1.3.4: Administrative

3.7: License agreement

3.7.1: GNU General Public License Version 3 for dbVOR

Chapter 4: Index

Index

Appendix A: MySQL mini-tutorial

---

Document generated by eLyXer 1.2.5 (2013-03-10) on 2015-02-16T11:17:24.442337
