# Supplementary material for: dbVOR: a database system for importing pedigree, phenotype and genotype data and exporting selected subsets
Source: BMC Bioinformatics. 2015 Mar 18;16(1):91. doi: 10.1186/s12859-015-0505-4 (PMC4407391; doi:10.1186/s12859-015-0505-4)
Supplement: Additional file 2 — Contains the entire Genetic Map Interpolator (GMI) package, version 1.4. [file 12859_2015_505_MOESM2_ESM.zip › 1652824117132674_add2.tgz!/GMI_bioc_v1.4/GMI_manual_v1.4.doc]

#### Genetic Map Interpolator (GMI)

**Bioconductor version**

Version 1.4

November 13, 2014

Originally written by Xinyu Tang and Nandita Mukhopadhyay under the supervision of Daniel E. Weeks. Updated by Robert V. Baron.

Copyright © 2010-14 by Xinyu Tang, Nandita Mukhopadhyay, Robert V. Baron, Daniel E. Weeks, and the University of Pittsburgh.

Supported by NIH grant R01GM076667 "Mega2: Manipulation Environment for Genetic Analyses" (PI Daniel E. Weeks) and the University of Pittsburgh.

### Overview

Many statistical algorithms for analyzing genetic data require ***genetic*** ***maps*** of the markers, which specify the recombination rates between adjacent markers. However, while it is relatively easy to extract physical map positions from the online databases, it is more difficult to extract genetic map positions. Furthermore, some of the genetic map tools require that one input not only the marker ID, but also the marker's physical position in a specified older build of the genome.

The Genetic Map Interpolator (GMI) package is designed to create interpolated genetic maps of single nucleotide polymorphism (SNP) markers. Starting from a list of single nucleotide polymorphism (SNP) **rs** numbers, GMI uses the R packages biomaRt and RMySQL to fetch the most up-to-date SNP and microsatellite physical positions from Ensembl, and then combines these with the Rutgers combined genetic and physical map (Matise et al., 2007; Kong et al., 2004; Kong and Matise, 2004) to estimate the corresponding Kosambi genetic positions by linear interpolation for these SNPs. The resulting information is then output in map files formatted to be read in by our data-reformatting program, Mega2 (Mukhopadhyay et al., 2005; <http://watson.hgen.pitt.edu/register/>).

SNPs were assigned rs IDs at the time of discovery, and, later if another SNP was found to be identical to a previously discovered SNP, their rs IDs were merged to one representative rs ID (usually the one with the lower number). If a SNP is not found in Ensembl, GMI then queries NCBI’s Entrez SNP database using *eutils* to see if this SNP has been renamed; if so, GMI then searches Ensembl a second time with the new SNP ID. The user has the option to use either the old or the new ID in the output map.

For each of these database queries, the list of SNPs being queried is broken up into smaller chunks so that each individual query can be completed within a few seconds. This has been done to prevent premature timing out of web connections, and in order to obey the limit guidelines required for Entrez queries.

GMI is a Unix-based program written in Perl and R, and is portable over most Unix platforms. We have used and tested it extensively on both Intel and PPC-based Macs. Setup is done via a text-based user-interface within a Unix shell. Subsequently, queries can be executed from the Unix command line.

GMI has a number of useful features (see Figure 1):

- GMI automatically looks up and uses the physical positions for each marker from the *most recent* Ensembl build.
- For each SNP that is not initially found in Ensembl, GMI automatically checks to see if that SNP has been assigned a new rs number.
- GMI automatically figures out which chromosome each marker is on, so knowledge of a marker's chromosome is not required to run GMI.

Please note that GMI relies crucially upon the Rutgers Combined Linkage-Physical Map, created by Tara Matise and colleagues (Matise et al., 2007; Kong et al., 2004; Kong and Matise, 2004), and we would like to thank them for making this important well-validated framework map available to the scientific community. GMI would not be possible without it.

**Figure 1:** A flow chart outlining GMI's operations; this was constructed when the current Ensembl build was 'Release 59'.

### Installation

#### Overview of the installation steps

Here is a brief outline of the steps required to install GMI within a Unix environment.

1. Install required software:
   a. R
   b. Perl
   c. ftp or wget
   d. The R package 'biomaRt'
   e. The R package 'RMySQL'
   f. The R package 'DBI'
2. Download the compressed tar archive GMI_bioc_v1.4.tar.gz from our software distribution site <http://watson.hgen.pitt.edu/register>
3. Decompress and expand the archive: tar -xzf GMI_bioc_v1.4.tar.gz
4. Change directory into the newly-created GMI_bioc_v1.4 folder
5. Run gmi_bioc_install.pl to install GMI

After successful installation, here is the simplest way to use GMI:

1. List your SNP rs names, one per line, in a text file named 'snp_list.txt'.
2. Run GMI on your file via this Unix command: gmi.pl snp_list.txt

This should create the following files:

snp_mapped_annot.txt	Map file in Mega2's annotated format

snp_mapped_nonannot.txt	Map file in Mega2's unannotated format

snp_mapped.pdf	PDF plots

snp_mapped_log.txt	Run summary file

### Detailed installation instructions

#### Requirements

GMI depends on various external components that need to be installed onto your computer before you can run GMI. As stated previously, GMI is a Unix-based program; therefore, it is assumed that you have some familiarity with using Unix. On a Mac OS X machine, you can access the underlying Unix environment by opening the Terminal application and typing the Unix commands within that window. On a Windows machine, we recommend using the Cygwin package (See the Mega2 documentation for more details).

1. **R v2.8.0 or above available from** [**http://cran.r-project.org/**](http://cran.r-project.org/)

GMI uses R extensively; therefore, you need to have R installed on your computer. R is a free, publicly available and popular statistical package that has an extensive collection of libraries and toolboxes for a wide range of statistical applications. The *biomaRt* package is used to look up the most up-to-date physical positions for SNPs from Ensembl. The *RMySQL* package is used to directly query the Ensembl microsatellite MySQL database for the most up-to-date physical positions. GMI also uses R to interpolate genetic map positions from physical map positions for SNPs that are not found in the Rutgers combined maps. For this, it uses the *approxfun* linear interpolation function from the *Stats* package, which is part of the basic R distribution.

2. **Perl v5.6.0 or above available from** [**http://www.perl.org/**](http://www.perl.org/)

Perl is needed to query the BioMart web site for the latest version number of the *H. Sapiens* SNP database; the installation and query scripts are also written in Perl. If you are running Linux or Mac OS X, Perl is very likely to be already installed on your system. However, make sure that the version is at least 5.6 or higher.

3. **ftp or wget**

The ftp program comes installed on most Unix computers. To see if this is available, use the *which* command at the Unix prompt:

which ftp

You should see something like:

/usr/bin/ftp

Alternately, you can obtain the wget program, a free GNU software package used for retrieving files using HTTP, HTTPS and FTP internet protocols. It can easily be installed following instructions from the web site http://www.gnu.org/software/wget. GMI requires one of these programs to fetch a few necessary bits of information from BioMart’s web site.

[5. OPTIONAL: **MySQL 5.0 or above available from http://mysql.com/**

If you need to install RMySQL from source (instead of from an R package), then MySQL should be installed first on your computer.]

#### Installation and setup

##### Install the biomaRt package in R

To install the package, start R and enter, at the R prompt, these R commands:

source( "http://www.bioconductor.org/biocLite.R")

biocLite("biomaRt")

##### Detailed information can be found at:

<http://www.bioconductor.org/packages/2.2/bioc/html/biomaRt.html>

##### I. Install the RMySQL package in R

Use the Package Installer menu of R to install the RMySQL package, choosing the option to install dependencies. Since RMySQL depends on the DBI package, this should install both the RMySQL package and the DBI package.

Alternatively, download the source code from <http://cran.r-project.org/>, and install the package using an R CMD install command at the Unix prompt like this:

R CMD install RMySQL_0.7-4.tar.gz

Prior to running the R install command, you may also need to configure your Unix environment in a way such that RMySQL is able to find the information that it needs about your MySQL installation. For details on how to do this correctly, follow the installation instructions at:

<http://cran.r-project.org/web/packages/RMySQL/INSTALL>

##### II. Install the DBI package in R

Check that the DBI package was installed in the previous step by typing

library(DBI)

at the R prompt. If it has been installed, you will be immediately returned to the R prompt with no errors. If DBI has not been installed, then you will see something like this:

Error in library(DBI): there is no package called 'DBI'

If you encounter such an error, then use the Package Installer menu of R to install the DBI package, or download the source code from <http://cran.r-project.org/>, and install the package using an R CMD install command at the Unix prompt like this:

R CMD install [DBI_0.2-4.tar.gz](http://cran.r-project.org/src/contrib/RMySQL_0.7-4.tar.gz)

##### III. Prepare to install Bioconductor version GMI

Download the Bioconductor version of GMI "GMI_bioc_v1.4.tar.gz" from our software distribution site: <http://watson.hgen.pitt.edu/register>

Untar and unzip the GMI package “GMI_bioc_v1.4.tar.gz”:

tar -xzf GMI_bioc_v1.4.tar.gz

This creates the folder GMI_bioc_v1.4 containing these files:

1. **gmi_bioc_install.pl**

This is the install script, typically run only once when you download a new version of GMI. For GMI to be installed within system level folders, installation should be done by an administrative user with read-write privileges to system folders.

2. **gmi.bioc.setdata.R**

This is an R source file defining the R function *gmi.bioc.setdata*, which looks for the most up-to-date SNP and microsatellite physical positions from Ensembl.

3. **gmi.pl**

This is a Perl program which queries the Ensembl database for a given list of SNP *rs IDs*, and creates the genetic map. This is a front-end to the R mapping function described below.

4. **gmi.bioc.mapping.R**

R source file defining the R function *snp.bioc.mapping*, which maps physical positions to genetic positions in Kosambi centiMorgans.

5. **gmi_bioc_utils.pl**

This contains some utility functions that are used by the installation and querying programs.

6. **gmi.bioc.checkver.R**

When a query is run, this R utility compares the Ensembl build number with the version number of the SNP data files used by GMI to create the map. If the Ensembl version is newer, the user may choose to terminate the query.

The GMI folder will be subsequently populated with other files and folders in the following steps. **This folder is integral to our mapping package. Therefore, it should not be moved or otherwise modified.**

##### IV. Run gmi_bioc_install.pl to install and configure GMI

To install GMI, *cd* into the GMI package folder GMI_bioc_v1.4/ and type, at the Unix prompt, the following:

./gmi_bioc_install.pl

The install script requires you to specify several things:

i. ***GMI executables_folder name***

This is the folder where the query program *gmi.pl* should be installed, typically the folder where you normally place all your executable programs. This folder name can also be either relative or absolute. The default folder is set to **/usr/local/bin.**

In Unix, programs available to all users are typically placed inside /usr/bin or /usr/local/bin; these directories typically need administrative privileges to write to. Alternatively, the *bin* folder within each user’s home folder is another standard location for executable programs that are available to that user, and needs only user-level read-write privileges. Therefore, if you are installing GMI as a normal user inside your home folder, you could use your *bin* folder as the GMI *executables_folder***.**

ii. ***Whether Rutgers map files should be downloaded***

The maps are required for installation to proceed when you install GMI for the first time. The installer downloads the Rutgers maps from <http://compgen.rutgers.edu/RutgersMap/Default.aspx>. If these were already downloaded during a previous installation, you map skip this step by answering “no” at the prompt.

iii. ***Your e-mail address***

This is necessary to query the Entrez SNP database, Please make sure to provide a valid e-mail address, as this is then used by NCBI uses this e-mail to track the status of your query, and notify you if something went wrong.

If *gmi_bioc_install.pl* ran successfully, you will see messages like these at the end:

```
Creating merged maps for SNPs on all chromosomes ...1 2 3 4 5 6 7 8 9 10 11 12 13 14 15 16 17 18 19 20 21 22 X
Creating merged maps for microsatellites on all chromosomes ...


Finished installing GMI.
To re-configure, run gmi_bioc_install.pl again from within this folder.
To use GMI, run gmi.pl.
Installed the following GMI components:
  GMI query program:   /Users/nandita/bin/gmi.pl
  Rutgers map files:   /Users/nandita/GMI_bioc_v1.4
  Merged map files:    rutgers_merged_59
  Configuration file:  /Users/nandita/GMI_bioc_v1.4/CONFIG.txt
gmi.pl written to /usr/bin.
```

The configuration file *CONFIG.txt* can be used if you need to re-install GMI, simply modify the file as necessary, and run **gmi_install.pl** followed by the configuration file name:

gmi_install.pl CONFIG.txt

This installation process copies the appropriately configured query Perl program to the executables folder ***executables_folder***. It also runs the R script gmi.bioc.setdata.R to set up local merged physical and genetic maps, fetching the most recent physical map for SNPs and microsatellites, merging these with the Rutgers maps to create up-to-date flat files named *chr#.comb* (the *#* denoting chromosome number), within a new folder named *rutgers_combined_# (# is the most recent Ensembl version number)* inside the GMI install folder. If a Rutgers marker is not found inside the Ensembl database, its physical position is coded as NA.

After installation, the GMI install folder should not be modified or moved, since this contains critical components needed for GMI to function.

On some Unix computers, you may need to type 'rehash' to update internal system information, so that *gmi.pl* is now part of the list of applications available to you.

You can re-run the install script *gmi_bioc_install.pl* to update these maps, when

Ensembl publishes a new version of the SNP database. The query utility *gmi.pl* checks for the latest version on Ensembl and warns the user if the local files need to be updated.

Here is an excerpt from the local combined maps set up by GMI for autosomal chromosomes:

```
Markers_name    Build36_map_physical_position   Sex.averaged_map_position       Smoothed_sex.averaged_map_position      Female_map_position   Smoothed_Female_map_position    Male_map_position       Smoothed_male_map_position      Ensembl_map_physical_position
D20S1155        30753   0       0       0       0       0       0       83173
rs1342137       73121   0       0.08    0       0.09    0       0.07    125121
rs735669        76713   0       0.08    0       0.09    0       0.08    128713
D20S1157        79508   0       0.09    0       0.1     0       0.08    131870
rs1434789       85900   0       0.12    0       0.11    0.44    0.13    137900
rs1865432       94951   0       0.17    0       0.13    0.44    0.24    146951
rs1858597       95685   0       0.18    0       0.13    0.44    0.25    147685
rs751596        99330   0       0.2     0       0.14    0.44    0.29    151330
rs722829        117701  0       0.31    0       0.18    0.44    0.52    169701
rs1469781       118642  0       0.32    0       0.18    0.44    0.53    170642
```

The X chromosome file *chr23.comb* is a special case with only 6 columns:

```
Markers_name    Build36_map_physical_position   Female_map_position     Smoothed_Female_map_position    Male_map_position       Ensembl_map_physical_position
DYS402  818410  0       0       0       898410
GATA2A12        860144  0       0.03    0       NA
DXS6814 1306723 0       0.31    6.1     NA
DXS1071 1627620 0       0.65    9.42    NA
rs924904        2578904 2.82    4.14    23.65   2568904
rs310136        2621451 2.82    4.63    25.98   2611451
rs311071        2642822 2.82    4.96    25.98   2632822
rs1268  2810677 2.89    7.01    28.77   2800677
rs211657        2816589 9.27    7.05    28.77   2806589
```

Warnings: You may notice one or more files named *chr#.warnings*. These files contain markers with duplicated records from Ensembl. Below are a few lines from “chr1.warnings” as an example:

```
Duplicate Markers Found in Ensembl:
D1S167	1	88740607
D1S167	1	88740598
D1S191	1	185821687
D1S191	1	185821622
D1S193	1	43019300
D1S193	1	43019292
D1S194	1	165437271
D1S194	1	165437258
```

If you list the contents of the GMI installation folder at this point, you will see your *smooth_map_b36* folder, a version file *version.txt*, and a new sub-folder called *rutgers_merged_[Ensembl version number]*, containing data and maybe warnings files for chromosomes 1 through 23.

### Usage

##### Using gmi.pl to create genetic maps

Once the installation process above has been completed, you should be able to create new map files using the query program by simply typing *gmi.pl* at the Unix prompt like so:

gmi.pl [snplist]

where [snplist] is the name of a text file containing a list of SNP rs IDs.

The *gmi.pl* script checks the Ensembl release version first. If your Ensembl release version used for creating local merged files is not the latest released version, it will print a warning message:

Local databases need to be updated, continue with GMI query? [answer "yes" or "no"] (default no) > yes

If you answer “no”, the query will terminate. The update can be done by running *gmi_bioc_install.pl* inside the GMI install folder again.

##### Getting help on using gmi.pl

To list the various options, simply call gmi.pl without any arguments; a list of options and their use are printed on the screen.

```
Usage: gmi.pl snp_list_file [additional args]

Arguments:  --namecol [col], default 1
            --poscol [col], default = -1, meaning absent
            --chrcol [col], default = -1 meaning absent
            --header (no arguments), first line will be skipped if specified
            --outfile [output-file-prefix], default snp_mapped
            --noplots (no arguments), plotting will be skipped if specified
```

**Input file formats**

GMI will accept a variety of input file formats. The input file can be as simple as a list of rs numbers, one per line. Or the input file can be more complicated, with multiple columns of information, with or without a header line. **Important:** SNP IDs must be “rs” numbers, otherwise these will not be found by Ensembl and Entrez.

##### 1. rs IDs only

This input file is a text file containing only a list of SNP rs IDs, one name per line.

***2. rs IDs plus chromosomal and physical position information***

If you already have pre-specified chromosomes and physical positions, you can use GMI to compute genetic positions for these as well as Ensembl positions.

***Example input files***

The GMI distribution folder contains a subfolder named *example* containing input files with SNPs on autosomes, sex chromosomes and mitochondrial SNPs. It also includes output files and figures created by GMI.

Please take some time to familiarize yourself with GMI using these example files.

1. ***Simple input file format: snp_list.txt***

This input file is a text file containing only a list of SNP IDs, one name per line:

rs6679445

rs6713305

rs1879804

rs6787513

rs6862022

rs1457266

etc.

There are other similar input files for X, Y, and mitochondrial chromosomes named *X_snp_list.txt****,*** *Y_snp_list.txt* and *mito_snp_list.txt*. SNPs on X chromosomes produce only female genetic map distances, whereas Y and mitochondrial SNPs do not have any genetic positions (as there is no recombination).

You may also specify a *prefix*, i.e. a string which is used to label the output file names, by adding the option *–outfile [prefix]* after the input file name.

2. ***Input file with pre-specified chromosomes and physical positions: snp_list_3col.txt***

If you already have pre-specified chromosomes and physical positions, you can use GMI to compute genetic positions for these as well as Ensembl positions.

rs6679445 1 240942076

rs6713305 2 177855789

rs1879804 3 125560353

rs6787513 3 155686602

rs6862022 5 160037922

rs1457266 8 24825757

rs7840334 8 53276984

rs16875341 8 108018022

etc.

For this file, when you run the query, you also need to specify these additional arguments, *--namecol 1* (column number containing SNP names), *--chrcol 2* (column number containing chromosome numbers) and *--poscol 3 (*column number containing physical positions). So, to run GMI on this input file, we would use this command:

gmi.pl snp_list_3col.txt --namecol 1 --chrcol 2 --poscol 3

To learn about all input arguments to ***gmi.pl,*** simply type this command at the Unix prompt:

gmi.pl

Please note that if you wish to provide your own positions, your input file must contain both position and chromosome numbers, otherwise user-specified map information will be ignored. Also make sure that these options are given ***after*** the input file name.

***Tip:*** You can use any table-format file as an input file as long as you correctly specify the names column (and if present, chromosome and position columns), such as this file from a previous run of GMI.

```
Chromosome   Map.k.a    Name            Map.k.m   Map.k.f   Ensembl57.p
1            269.3765   rs6679445      194.1398  348.2916   242875453
1            281.5089   rs12094001     209.0345  357.5789   248090251
1            281.5948   rs1933162      209.0774  357.6648   248179029
1            281.7533   rs7537031      209.1567  357.8233   248342837
```

In this case, the file contains a header line that should be skipped; therefore, you need to also supply the *–header* option.

***3. Output files for snp_list.txt***

GMI creates several output files during each run, including (i) an annotated format Mega2 map file, (ii) a non-annotated format map file, (iii) a PDF file of plots for genetic vs. physical positions for each chromosome contained in the data, and (iv) a diagnostic file containing SNPs which could not be mapped. If the user provided a prefix when executing the query, these files will be named *prefix_annot.txt, prefix_nonannot.txt, prefix.pdf,* and *prefix_log.txt* respectively. The default prefix is “snp_mapped”. The output files are a little different between the two types of input files.

For the simple SNP file *snp_list.txt*, the corresponding output files are *snp_list_annot.txt*, *snp_list_nonannot.txt*, *snp_list.pdf* and *snp_list_log.txt*. There are corresponding files for each of the other input files inside the *example/* folder. The following files were created using Ensembl build 57.

(i) *snp_list_nonannot.txt*

```
Chromosome  Kosambi  Name       Male     Female   Ensembl57     Extrapolation
1           269.3765 rs6679445  194.1398 348.2916 242875453       0
1           281.5089 rs12094001 209.0345 357.5789 248090251       1
1           281.5948 rs1933162  209.0774 357.6648 248179029       1
1           281.7533 rs7537031  209.1567 357.8233 248342837       1
1           281.7724 rs10888327 209.1662 357.8424 248362579       1
1           281.8929 rs4451579  209.2264 357.9629 248487016       1
14          40.4533  rs2050481  40.0416  41.29    41071635        0
15          6.1668   rs9944233  5.917    6.378    25786005        0
16          121.0994 rs2934467  94.1398  150.0774 84971423        0
```

This file is in Mega2 non-annotated map file format.

(ii) *snp_list_annot.txt*

```
Chromosome Map.k.a  Name       Map.k.m   Map.k.f     Ensembl57.p X.Extrapolation
1       269.3765    rs6679445  194.1398  348.2916    242875453       0
1       281.5089    rs12094001 209.0345  357.5789    248090251       1
1       281.5948    rs1933162  209.0774  357.6648    248179029       1
1       281.7533    rs7537031  209.1567  357.8233    248342837       1
1       281.7724    rs10888327 209.1662  357.8424    248362579       1
1       281.8929    rs4451579  209.2264  357.9629    248487016       1
14      40.4533     rs2050481  40.0416    41.29      41071635        0
15      6.1668      rs9944233  5.917       6.378     25786005        0
16      121.0994    rs2934467  94.1398   150.0774    84971423        0
```

This file is in Mega2 annotated map file format.

The last column 'X.Extrapolation' denotes whether a SNP position needed to be **extrapolated*,*** i.e. there were no anchored SNPS in the Rutgers maps that flank both sides of that SNP. Extrapolation is less accurate than interpolation; therefore the user may opt to exclude these SNPs based on the Extrapolation flag.

(iii) *snp_list.pdf* is a PDF file containing two types of plots: plots of physical positions vs. sex-averaged, female and male genetic positions, and ladder plots of sex-averaged, female and male genetic positions. Each plot represents a single chromosome.
(iv) *snp_list_log.txt* is a diagnostic file containing run-related information. It contains the run date, input file name and problematic SNPs (possible problems listed below).

```
---------------------------------------------------
   Names of the following SNPs have changed:
---------------------------------------------------
Old     New
rs1234455       rs854057
---------------------------------------------------

---------------------------------------------------
   SNPs with extrapolated map positions:
---------------------------------------------------
Name    Chromosome      Ensembl.p       Map.k.a Map.k.f Map.k.m Extrapolation
rs12094001      1       248090251       281.5089        357.5789        209.0345        1
rs1933162       1       248179029       281.5948        357.6648        209.0774        1
rs7537031       1       248342837       281.7533        357.8233        209.1567        1
rs10888327      1       248362579       281.7724        357.8424        209.1662        1
rs4451579       1       248487016       281.8929        357.9629        209.2264        1
---------------------------------------------------

---------------------------------------------------
   SUMMARY OF WARNINGS
---------------------------------------------------
  -> Some SNP names have changed in Ensembl.
       Map files contain old SNP names.
  -> 5 Markers on chromosome 1 have extrapolated map positions.
---------------------------------------------------
```

In general the diagnostics may include:

a. SNP IDs not found from Ensembl
b. SNPs which have been assigned new IDs
c. SNPs on Chromosome Y or Irregular Chromosomes
d. Bad SNPs with multiple records in Ensembl
e. SNPs with extrapolated map positions
f. SNPs with inconsistent map orders (where map order for user-provided positions does not match the map order for Ensembl positions)
g. SNPs with negative genetic distances.

4. ***Output files for snp_list_3col.txt***

The output map files contain extra columns for user-supplied physical positions:

(i) *snp_list_3col_annot.txt*

See Appendix A for a more readable version of this output file:

```
Chromosome	Map.k.a	Name	Map.k.m	Map.k.f	Build55.p	X.Extrapolation	X.Chromosome.e	X.Map.k.a.e	X.Map.k.m.e	X.Map.k.f.e	X.Ensembl.p	X.Extrapolation.e
1	263.9442	rs6679445	187.3606	344.0451	240942076	0	1	269.3765	194.1398	348.2916	242875453	0
1	278.4221	rs12094001	205.8335	354.857	246156874	0	1	281.5089	209.0345	357.5789	248090251	1
1	278.6498	rs1933162	206.0951	355.0457	246245652	0	1	281.5948	209.0774	357.6648	248179029	1
1	279.0698	rs7537031	206.5777	355.3939	246409460	0	1	281.7533	209.1567	357.8233	248342837	1
```

The first seven columns (from Chromosome to X.Extrapolation) are based on the user-provided position information, which means the genetic positions (Map.k.a, Map.k.m, Map.k.f) are interpolated using the user-provided physical positions (User.p) and chromosome numbers (Chromosome). The remaining six columns are Ensembl records, which means those genetic positions are interpolated using the physical positions and chromosome numbers retrieved from Ensembl. All headers for Ensembl records end with a suffix “.e”.

(ii) *snp_list_3col_nonannot.txt*

```
CHROMOSOME	KOSAMBI	NAME	MALE	FEMALE	X.PHYSICAL	X.EXTRAPOLATION	X.CHROMOSOME.E	X.KOSAMBI.E	X.MALE.E	X.FEMALE.E	X.PHYSICAL.E	X.EXTRAPOLATION.E
1	263.9442	rs6679445	187.3606	344.0451	240942076	0	1	269.3765	194.1398	348.2916	242875453	0
1	278.4221	rs12094001	205.8335	354.857	246156874	0	1	281.5089	209.0345	357.5789	248090251	1
1	278.6498	rs1933162	206.0951	355.0457	246245652	0	1	281.5948	209.0774	357.6648	248179029	1
1	279.0698	rs7537031	206.5777	355.3939	246409460	0	1	281.7533	209.1567	357.8233	248342837	1
1	279.1204	rs10888327	206.6359	355.4359	246429202	0	1	281.7724	209.1662	357.8424	248362579	1
```

Non-annotated format output files have the same format as annotated output file except for different headers. Both annotated format output file and non-annotated format output file have mega2 style headers.

(iii) *snp_list_3col.pdf*

There are three types of plots: (1) plots of physical positions vs. sex-averaged, female and male genetic positions, (2) ladder plots of sex-averaged, female and male genetic positions, and (3) plots of differences between user-specified and Ensembl physical positions. All three sets of plots are drawn chromosome by chromosome.

(iv) *snp_list_3col_log.txt*

The diagnostic file contains names of unmapped SNPs, SNPs with multiple Ensembl entries, SNPs with genetic distances that are negative or not in increasing order for both user-supplied and Ensembl physical maps.

### Troubleshooting

We have tried to anticipate problems with input files, and GMI checks for and successfully handles these:

1. Non-existent or unreadable input file:

The query terminates with the appropriate information.

2. Entrez SNP IDs should be non-zero integers prefixed with “rs”.

These SNPs are not queried and simply assigned unknown map positions.

3. Duplicate SNP IDs inside the input file:

These are detected and written out into a separate file *prefix_duplicates.txt*. The output map files contain only unique SNP names.

4. Header line is present but *–header* option was not supplied:

Since GMI has no control over the words uses in the header, this causes the first line to be read in as an input SNP name. This will very likely not be a valid SNP name and be treated as a non-conformant SNP ID (as in 2).

### Performance

GMI’s performance is bound by the speed with which it can fetch SNP information from Ensembl. We tested GMI on 300,000 Human HapMap SNPs on a Mac Powerbook. The time taken was 34 minutes, spent almost entirely on querying the Ensembl SNP database. In the future, we plan to use locally available annotated SNP files downloaded from Ensembl as flat files) and/or pre-computed common SNP panels (such as Illumina and Affymetrix panels) to speed up this process.

### References

Kong X, Murphy K, Raj T, He C, White PS, Matise TC. A combined linkage-physical map of the human genome. Am J Hum Genet. 2004 Dec;75(6):1143-8. Epub 2004 Oct 14. Erratum in: Am J Hum Genet. 2005 Feb;76(2):373. PubMed PMID: 15486828; PubMed Central PMCID: PMC1182151.

Kong X, Matise TC. MAP-O-MAT: internet-based linkage mapping. Bioinformatics. 2005 Feb 15;21(4):557-9. Epub 2004 Sep 16. PubMed PMID: 15374870.

Matise TC, Chen F, Chen W, De La Vega FM, Hansen M, He C, Hyland FC, Kennedy GC, Kong X, Murray SS, Ziegle JS, Stewart WC, Buyske S. A second-generation combined linkage physical map of the human genome. Genome Res. 2007 Dec;17(12):1783-6. Epub 2007 Nov 7. PubMed PMID: 17989245; PubMed Central PMCID: PMC2099587.

Mukhopadhyay N, Almasy L, Schroeder M, Mulvihill WP, Weeks DE. Mega2: data-handling for facilitating genetic linkage and association analyses. Bioinformatics. 2005 May 15;21(10):2556-7. Epub 2005 Mar 3. PubMed PMID: 15746282.

##### Links

Rutgers combined linkage-physical map: <http://compgen.rutgers.edu/RutgersMap/>

Mega2: <http://watson.hgen.pitt.edu/register/>

GMI: <http://watson.hgen.pitt.edu/register/>

##### Distribution

GMI is available from <http://watson.hgen.pitt.edu/register>. You need to register a valid e-mail address in order to download software from our web site. Once you have done so, you can download GMI and other genetics analysis programs using your registered e-mail. If you so indicate on your registration form, we will send you e-mail notifications when updates are made to GMI.

##### Feedback and bug-reports

You can use the feedback form linked to from the registration page at <http://watson.hgen.pitt.edu/register/feedback> to submit comments, suggestions and bug-reports.

**License**

This program is free software: you can redistribute it and/or modify it under the terms of the GNU General Public License as published by the Free Software Foundation, either version 3 of the License, or (at your option) any later version.

This program is distributed in the hope that it will be useful, but WITHOUT ANY WARRANTY; without even the implied warranty of MERCHANTABILITY or FITNESS FOR A PARTICULAR PURPOSE. See the GNU General Public License for more details.

You should have received a copy of the GNU General Public License along with this program. If not, see <http://www.gnu.org/licenses/>.

**Changes**

Version 1.4:

Fixed several bugs that caused GMI to fail under certain circumstances.

Version 1.3:

Updated broken link to the Rutgers map file.

**Appendix A**

Output from issuing the command:

gmi.pl snp_list_3col.txt --namecol 1 --chrcol 2 --poscol 3 --outfile snp_list_3col

The snp_list_3col_annot.txt file, where some of the names in the title line have been shortened so as to allow the more readable format here:

```
Chr Map.k.a   Name        Map.k.m   Map.k.f   User.p      X.Ext  X.Chr.e  X.Map.k.a.e X.Map.k.m.e X.Map.k.f.e X.Ens57.p   X.Ext.e
1   263.9442  rs6679445   187.3606  344.0451  240942076   0      1        269.3765    194.1398    348.2916    242875453   0
1   278.4221  rs12094001  205.8335  354.857   246156874   0      1        281.5089    209.0345    357.5789    248090251   1
1   278.6498  rs1933162   206.0951  355.0457  246245652   0      1        281.5948    209.0774    357.6648    248179029   1
1   279.0698  rs7537031   206.5777  355.3939  24640946    0      1        281.7533    209.1567    357.8233    248342837   1
1   279.1204  rs10888327  206.6359   55.4359  246429202   0      1        281.7724    209.1662    357.8424    248362579   1
14  40.1032   rs2050481   39.5732    41.2066  40141385    0      14       40.4533     40.0416     41.29       41071635    0
15  1.8686    rs9944233   1.7871     1.9515   23337098    0      15       6.1668      5.917       6.378       25786005    0
16  114.4439  rs2934467   89.939     141.1317 83528924    0      16       121.0994    94.1398     150.0774    84971423    0
2   184.5231  rs6713305   133.6204   237.3531 177855789   0       2       184.8313    133.8818    237.7008    178147543   0
3   133.6064  rs1879804   101.9584   167.8211 125560353   0       3       131.9522    100.6258    165.7007    124077663   0
3   165.7928  rs6787513   121.9074   212.2667 155686602   0       3       164.8238    120.5712    211.8365    154203908   0
5   168.8454  rs6862022   124.2886   215.4291 160037922   0       5       168.8877    124.3278    215.4773    160105344   0
8   45.7974   rs1457266   44.8464    47.7395  24825757    0       8       45.7689     44.8042     47.6989     24769852    0
8   66.8914   rs7840334   54.0524    80.7379  53276984    0       8       66.63       54.04       80.23       53114431    0
8   116.908   rs16875341  76.754     159.118  108018022   0       8       116.848     76.7045     159.045     107948846   0
U   NA        rs1234455   NA         NA       NA          0       7       105.7662    73.5256     139.4718    95303755    0
U   NA        rs4451579   NA         NA       NA          0       1       281.8929    209.2264    357.9629    248487016   1
```

The snp_list_3col.nonannot.txt file:

```
Chr     Kosambi  Name       Male      Female    User.p     Ext  Chr.E Kosambi.e  Male.e   Female.e  Ensembl57.p     Ext.e
1       263.9442 rs6679445  187.3606  344.0451  240942076  0    1     269.3765   194.1398 348.2916  242875453       0
1       278.4221 rs12094001 205.8335  354.857   246156874  0    1     281.5089   209.0345 357.5789  248090251       1
1       278.6498 rs1933162  206.0951  355.0457  246245652  0    1     281.5948   209.0774 357.6648  248179029       1
1       279.0698 rs7537031  206.5777  355.3939  246409460  0    1     281.7533   209.1567 357.8233  248342837       1
1       279.1204 rs10888327 206.6359  355.4359  246429202  0    1     281.7724   209.1662 357.8424  248362579       1
14      40.1032 rs2050481   39.5732   41.2066   40141385   0   14     40.4533    40.0416  41.29     41071635        0
15      1.8686  rs9944233   1.7871    1.9515    23337098   0   15      6.1668    5.917    6.378     25786005        0
16     114.4439 rs2934467   89.939   141.1317   83528924   0   16     121.0994   94.1398  150.0774  84971423        0
2      184.5231 rs6713305   133.6204  237.3531  177855789  0    2     184.8313   133.8818 237.7008  178147543       0
3      133.6064 rs1879804   101.9584  167.8211  125560353  0    3     131.9522   100.6258 165.7007  124077663       0
3      165.7928 rs6787513   121.9074  212.2667  155686602  0    3     164.8238   120.5712 211.8365  154203908       0
5      168.8454 rs6862022   124.2886  215.4291  160037922  0    5     168.8877   124.3278 215.4773  160105344       0
8       45.7974 rs1457266   44.8464   47.7395   24825757   0    8      45.7689   44.8042  47.6989   24769852        0
8       66.8914 rs7840334   54.0524   80.7379   53276984   0    8       66.63    54.04    80.23     53114431        0
8       116.908 rs16875341  76.754    159.118   108018022  0    8       116.848  76.7045  159.045   107948846       0
999     -99.00  rs1234455  -99.00    -99.00    -99.00      0    7       105.7662 73.5256  139.4718  95303755        0
999     -99.00  rs4451579  -99.00    -99.00    -99.00      0    1       281.8929 209.2264 357.9629  248487016       1
```
