# Supplementary material for: dbVOR: a database system for importing pedigree, phenotype and genotype data and exporting selected subsets
Source: BMC Bioinformatics. 2015 Mar 18;16(1):91. doi: 10.1186/s12859-015-0505-4 (PMC4407391; doi:10.1186/s12859-015-0505-4)
Supplement: Additional file 2 — Contains the entire Genetic Map Interpolator (GMI) package, version 1.4. [file 12859_2015_505_MOESM2_ESM.zip › 1652824117132674_add2.tgz!/GMI_bioc_v1.4/example/3col.pdf]

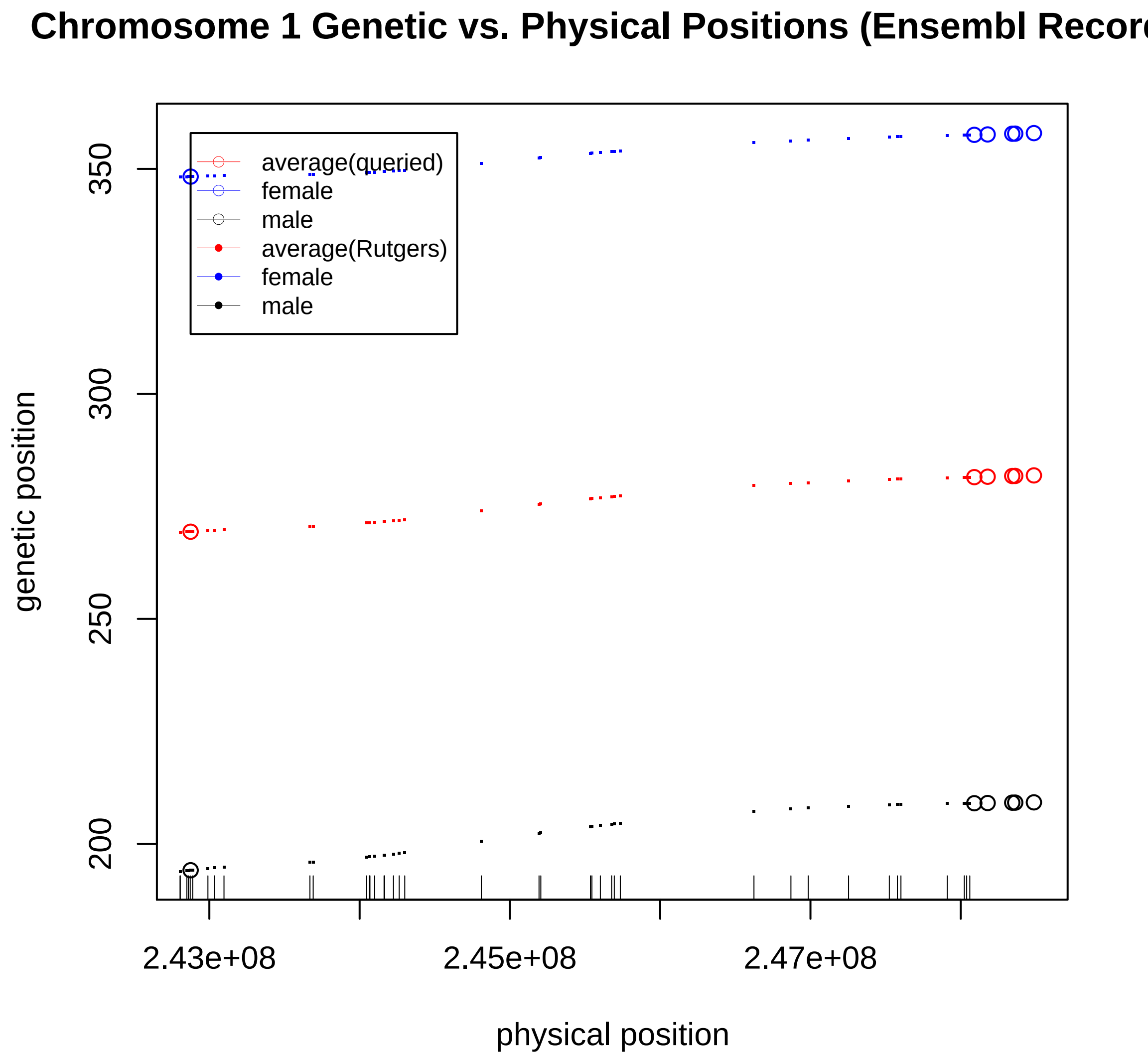

Chromosome 1 Genetic vs. Physical Positions (Ensembl Recor
average(queried)
female
male
average(Rutgers)
female
male
genetic position
350
300
250
200
2.43e+08
2.45e+08
2.47e+08
physical position

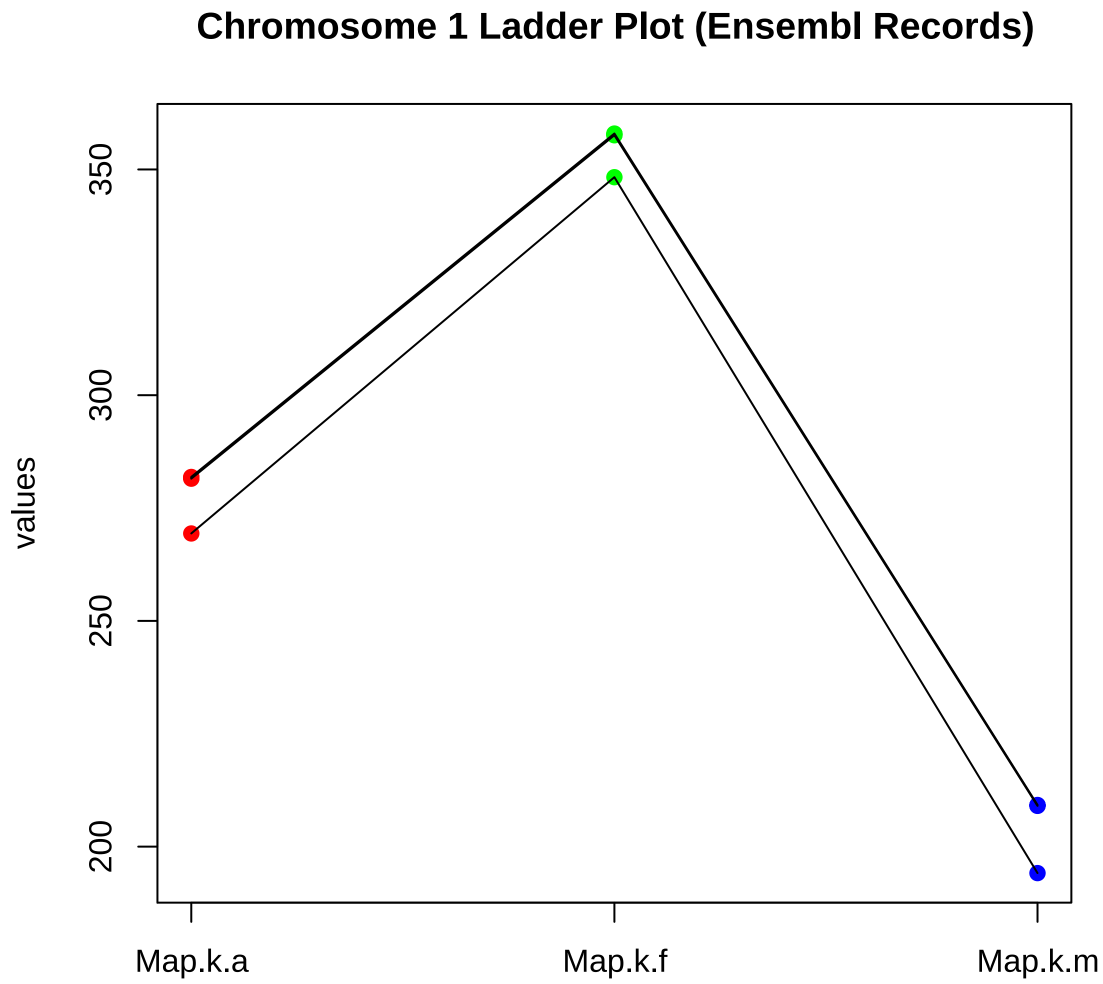

Chromosome 1 Ladder Plot (Ensembl Records)
values
350
300
250
200
Map.k.a
Map.k.f
Map.k.m

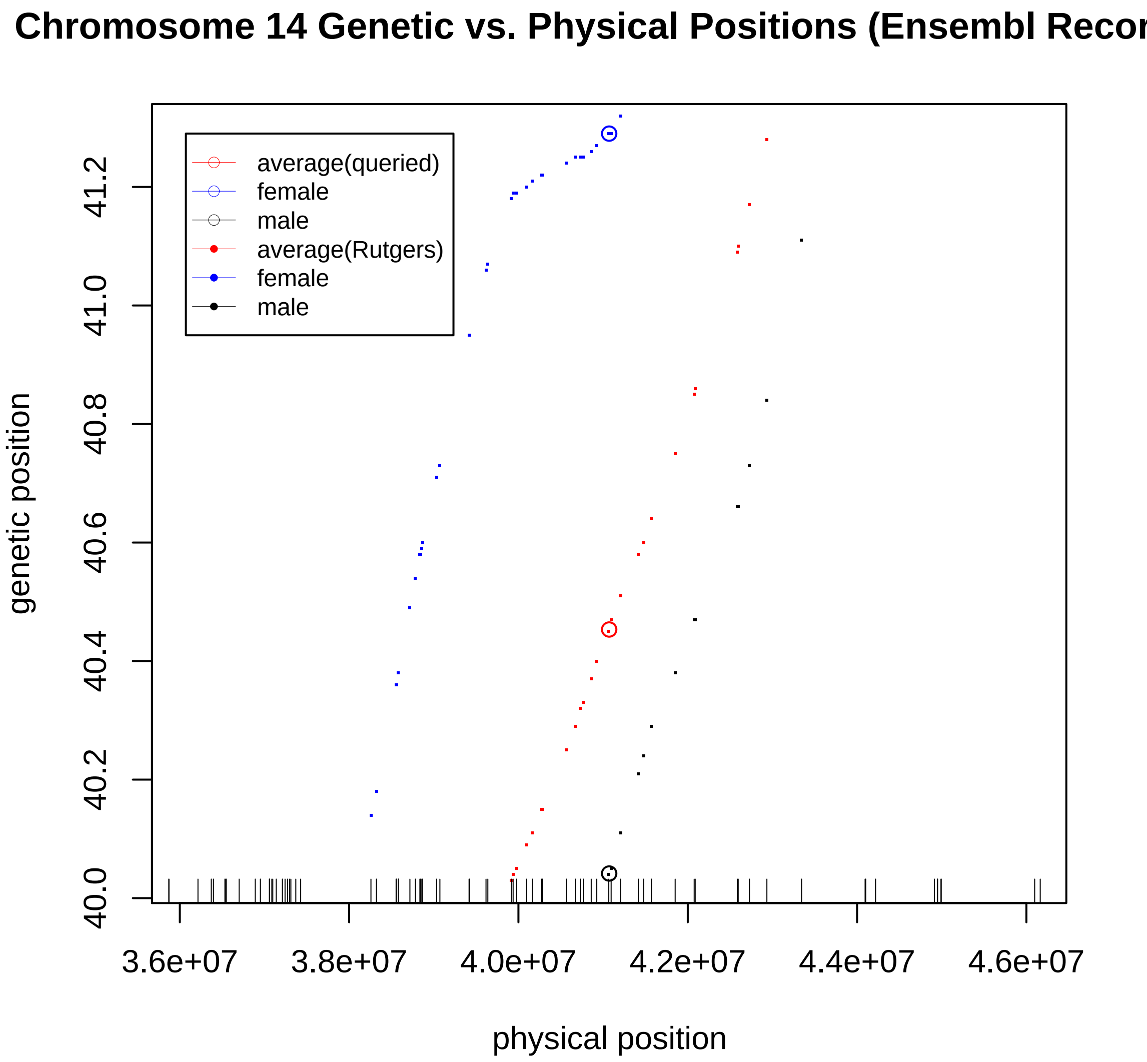

Chromosome 14 Genetic vs. Physical Positions (Ensembl Recor
average(queried)
female
male
average(Rutgers)
female
male
genetic position
40.0
40.2
40.4
40.6
40.8
41.0
41.2
physical position
3.6e+07
3.8e+07
4.0e+07
4.2e+07
4.4e+07
4.6e+07

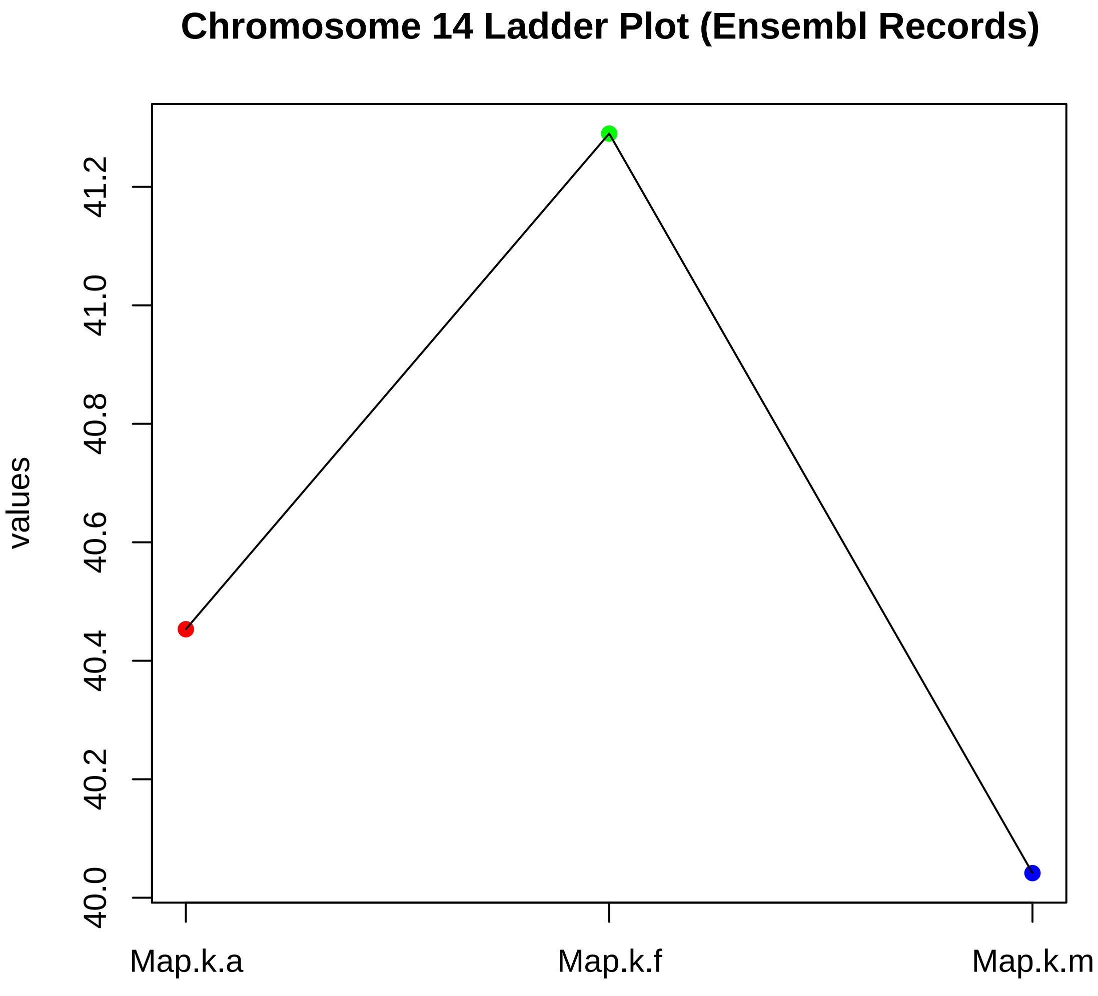
Chromosome 14 Ladder Plot (Ensembl Records)
values
41.2
41.0
40.8
40.6
40.4
40.2
40.0
Map.k.a
Map.k.f
Map.k.m

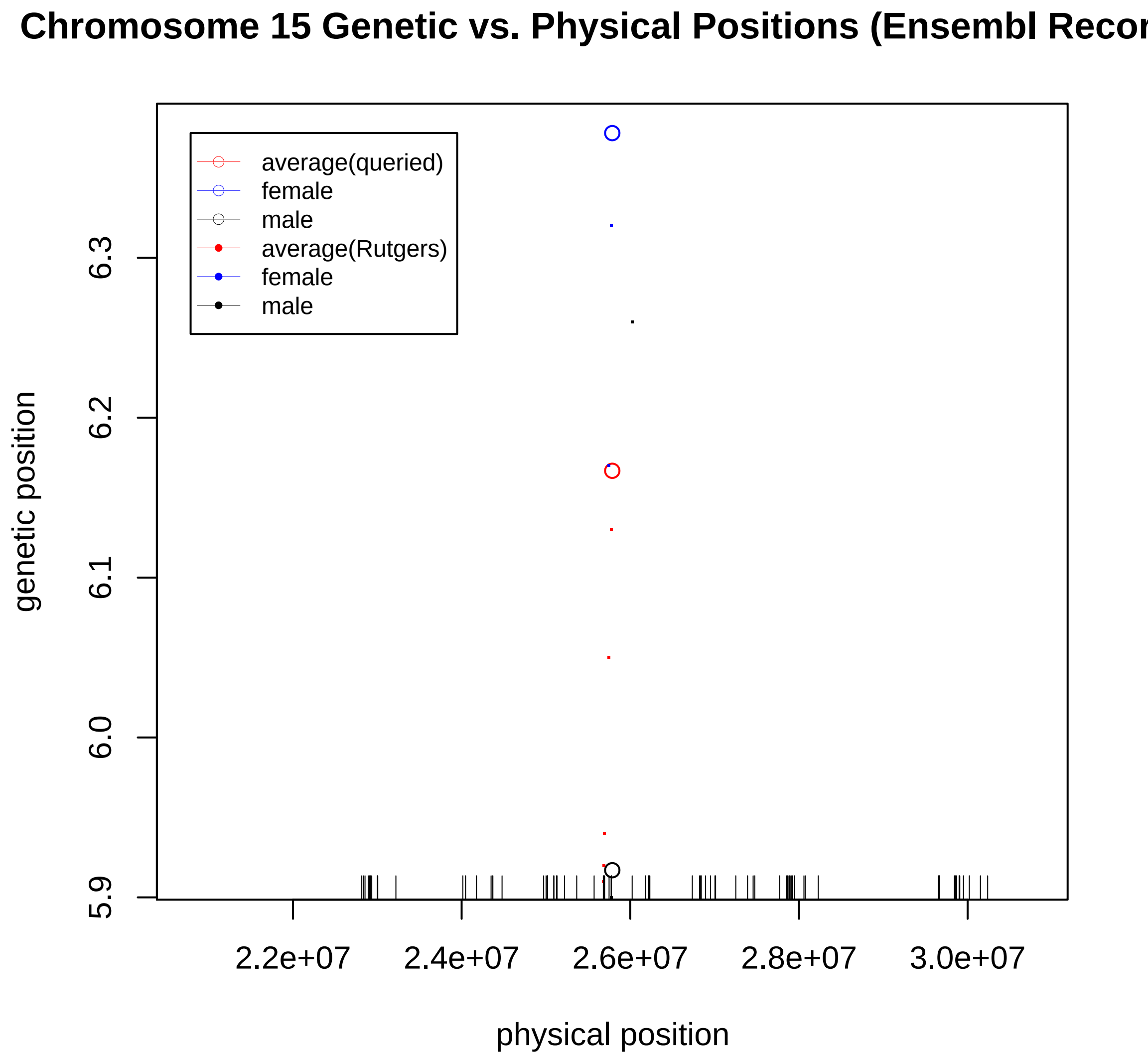

Chromosome 15 Genetic vs. Physical Positions (Ensembl Recor
average(queried)
female
male
average(Rutgers)
female
male
genetic position
6.3
6.2
6.1
6.0
5.9
2.2e+07
2.4e+07
2.6e+07
2.8e+07
3.0e+07
physical position

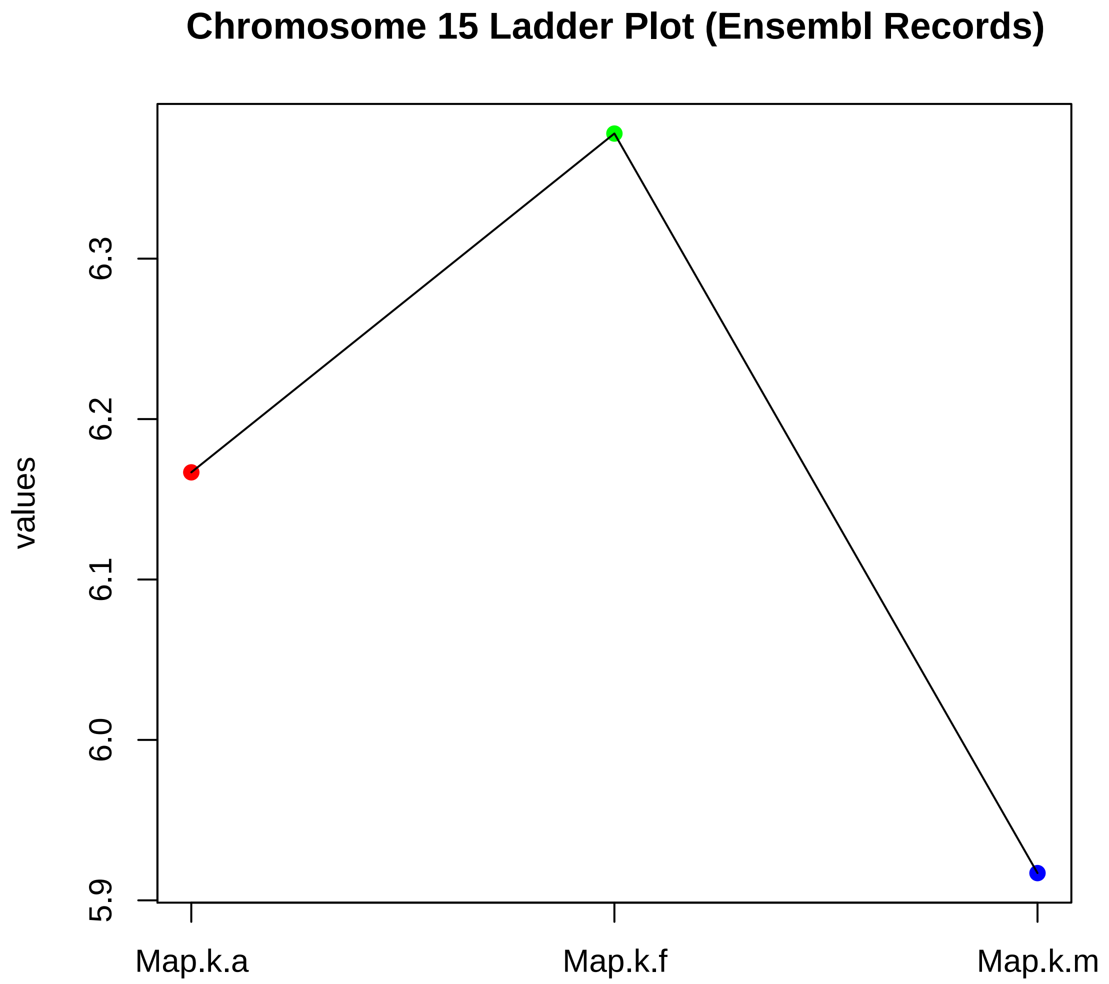

Chromosome 15 Ladder Plot (Ensembl Records)
values
6.3
6.2
6.1
6.0
5.9
Map.k.a
Map.k.f
Map.k.m

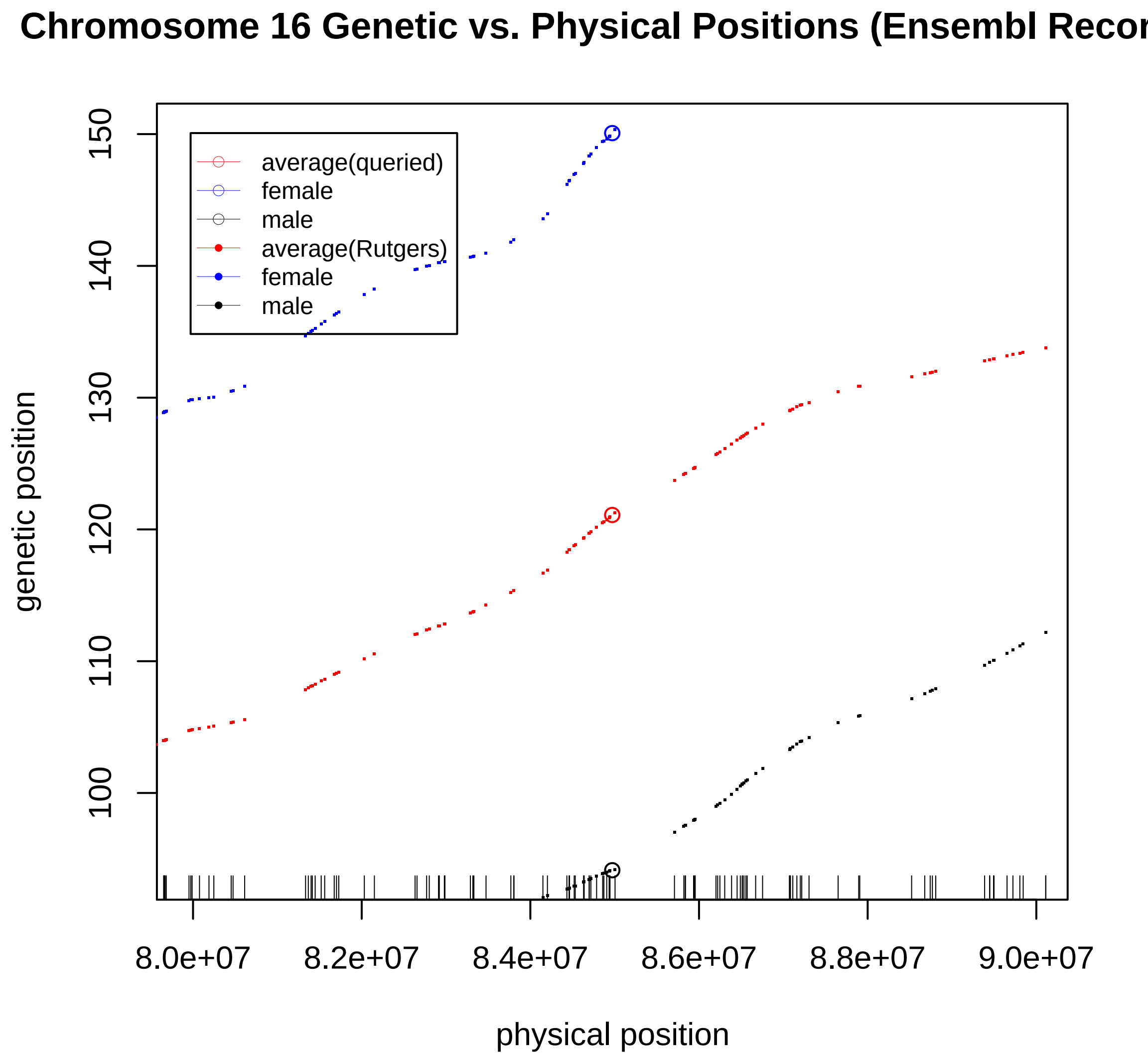

Chromosome 16 Genetic vs. Physical Positions (Ensembl Recor
average(queried)
female
male
average(Rutgers)
female
male
genetic position
100
110
120
130
140
150
physical position
8.0e+07
8.2e+07
8.4e+07
8.6e+07
8.8e+07
9.0e+07

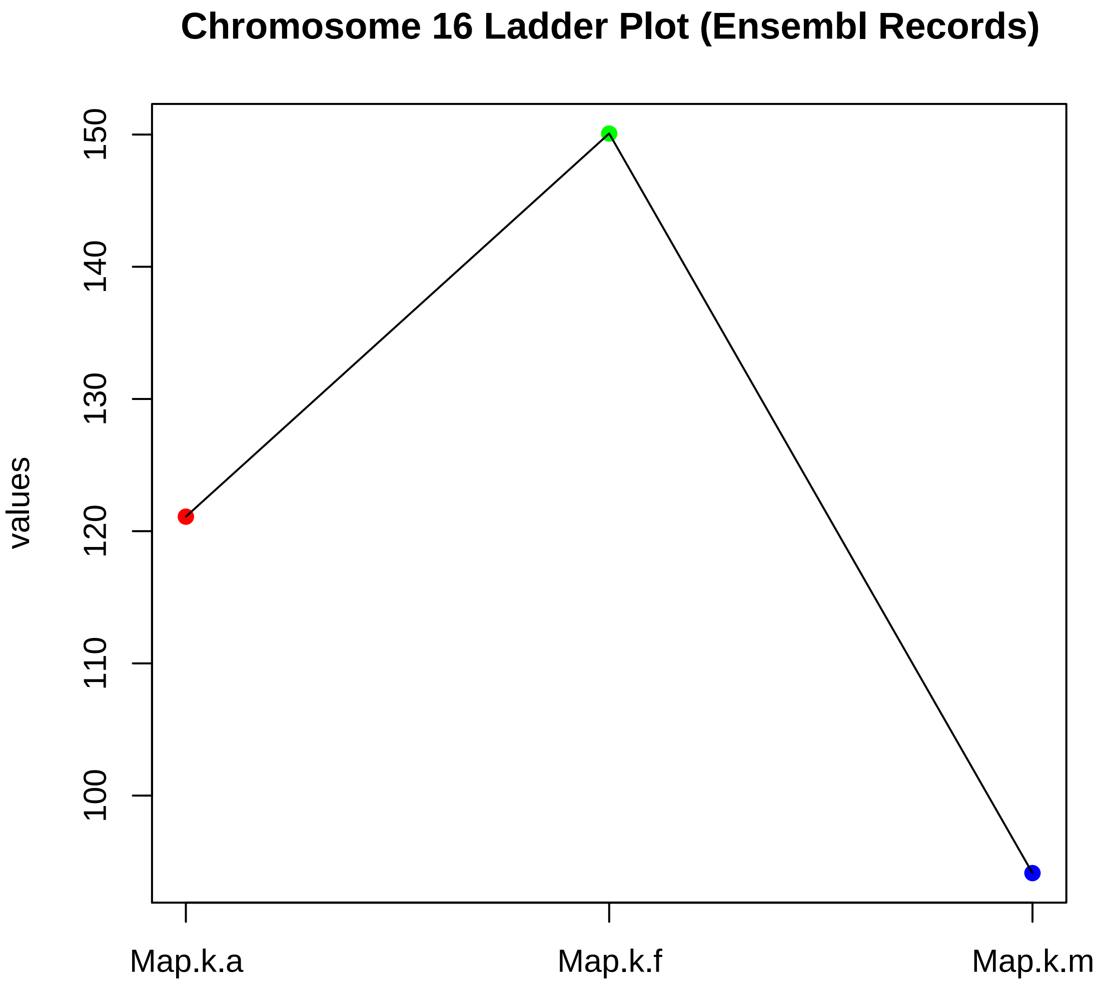

Chromosome 16 Ladder Plot (Ensembl Records)
values
150
140
130
120
110
100
Map.k.a
Map.k.f
Map.k.m

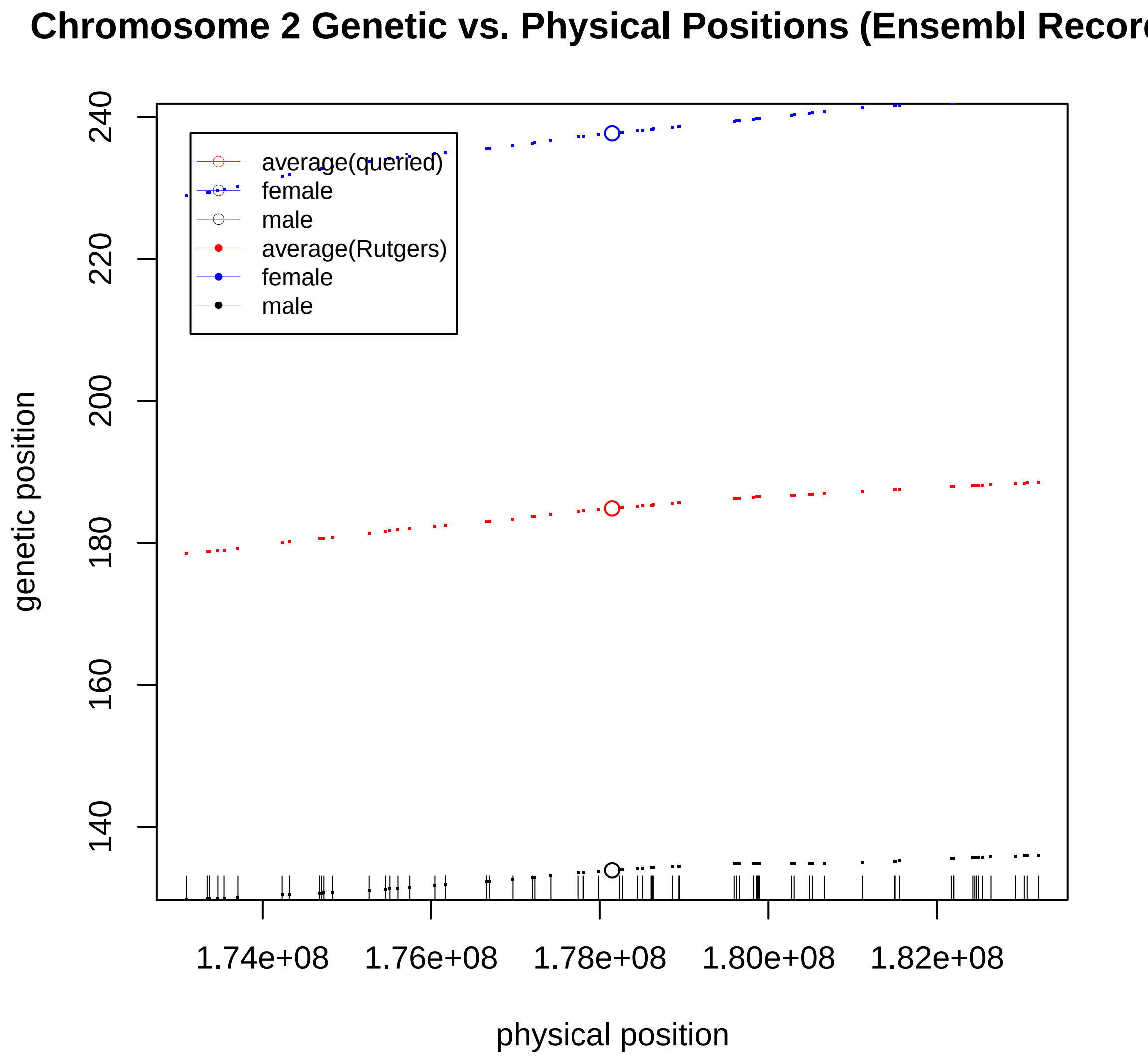

Chromosome 2 Genetic vs. Physical Positions (Ensembl Recor
average(queried)
female
male
average(Rutgers)
female
male
genetic position
240
220
200
180
160
140
1.74e+08
1.76e+08
1.78e+08
1.80e+08
1.82e+08
physical position

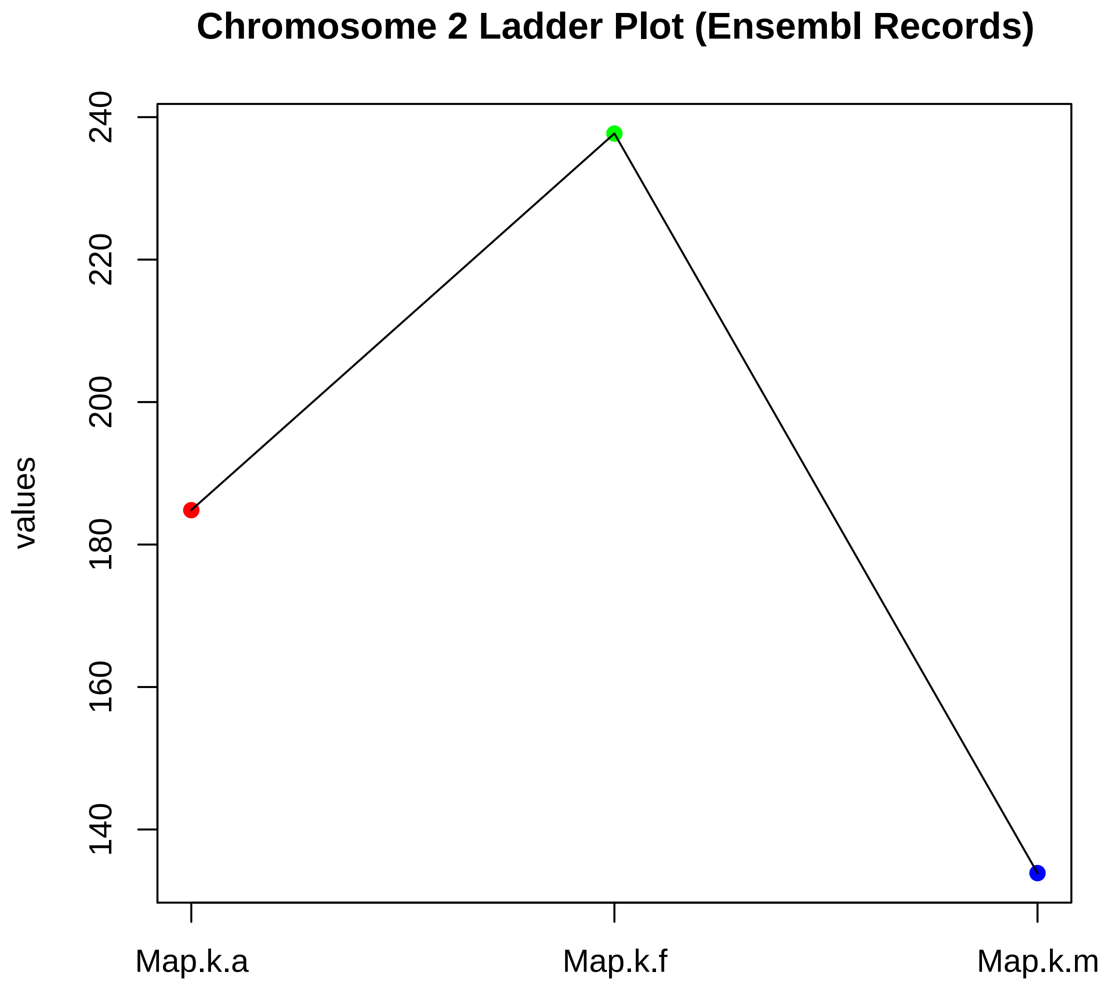

Chromosome 2 Ladder Plot (Ensembl Records)
values
240
220
200
180
160
140
Map.k.a
Map.k.f
Map.k.m

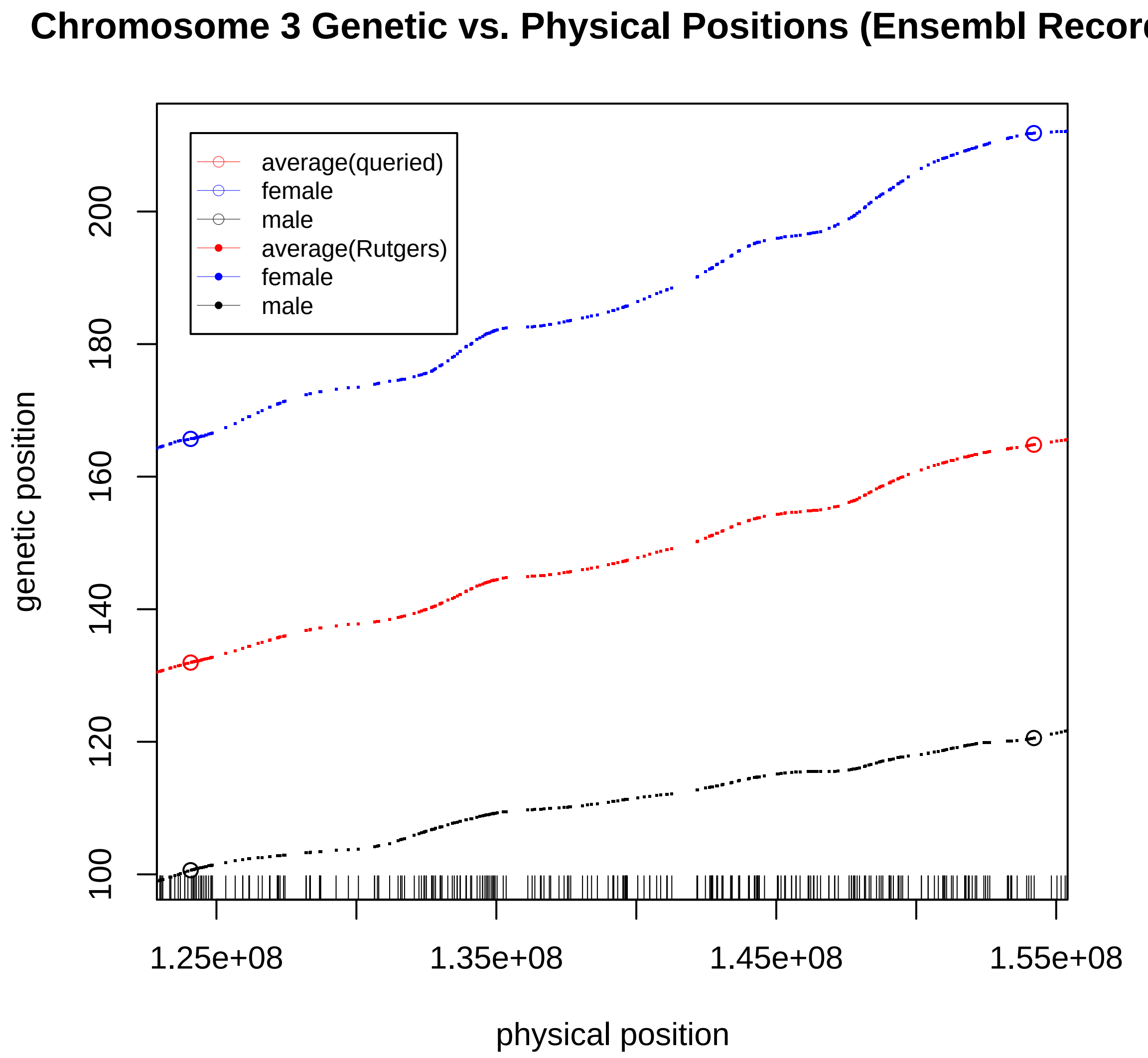
Chromosome 3 Genetic vs. Physical Positions (Ensembl Recor
average(queried)
female
male
average(Rutgers)
female
male
genetic position
100
120
140
160
180
200
1.25e+08
1.35e+08
1.45e+08
1.55e+08
physical position

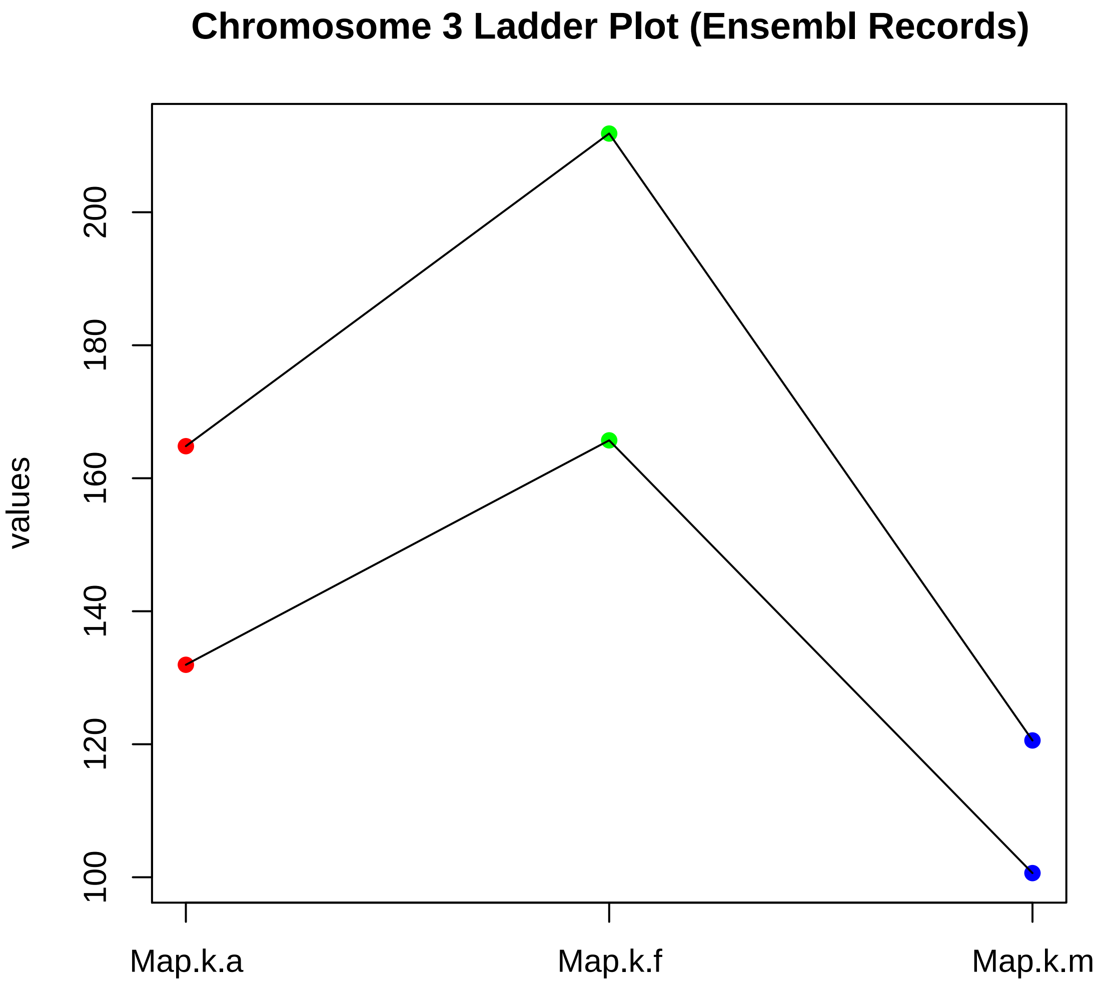
Chromosome 3 Ladder Plot (Ensembl Records)
values
200
180
160
140
120
100
Map.k.a
Map.k.f
Map.k.m

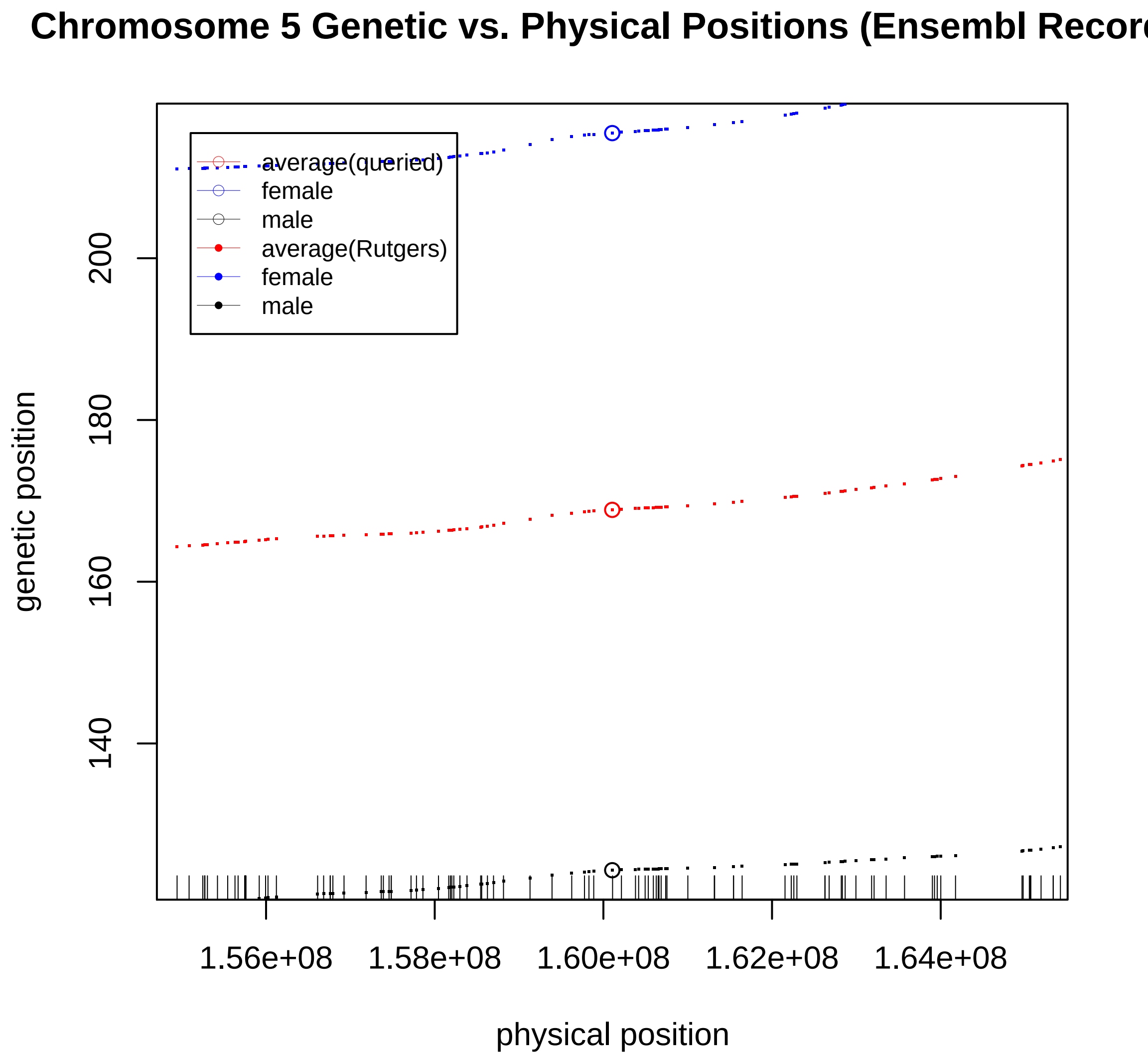

Chromosome 5 Genetic vs. Physical Positions (Ensembl Record
average(queried)
female
male
average(Rutgers)
female
male
200
180
160
140
genetic position
1.56e+08
1.58e+08
1.60e+08
1.62e+08
1.64e+08
physical position

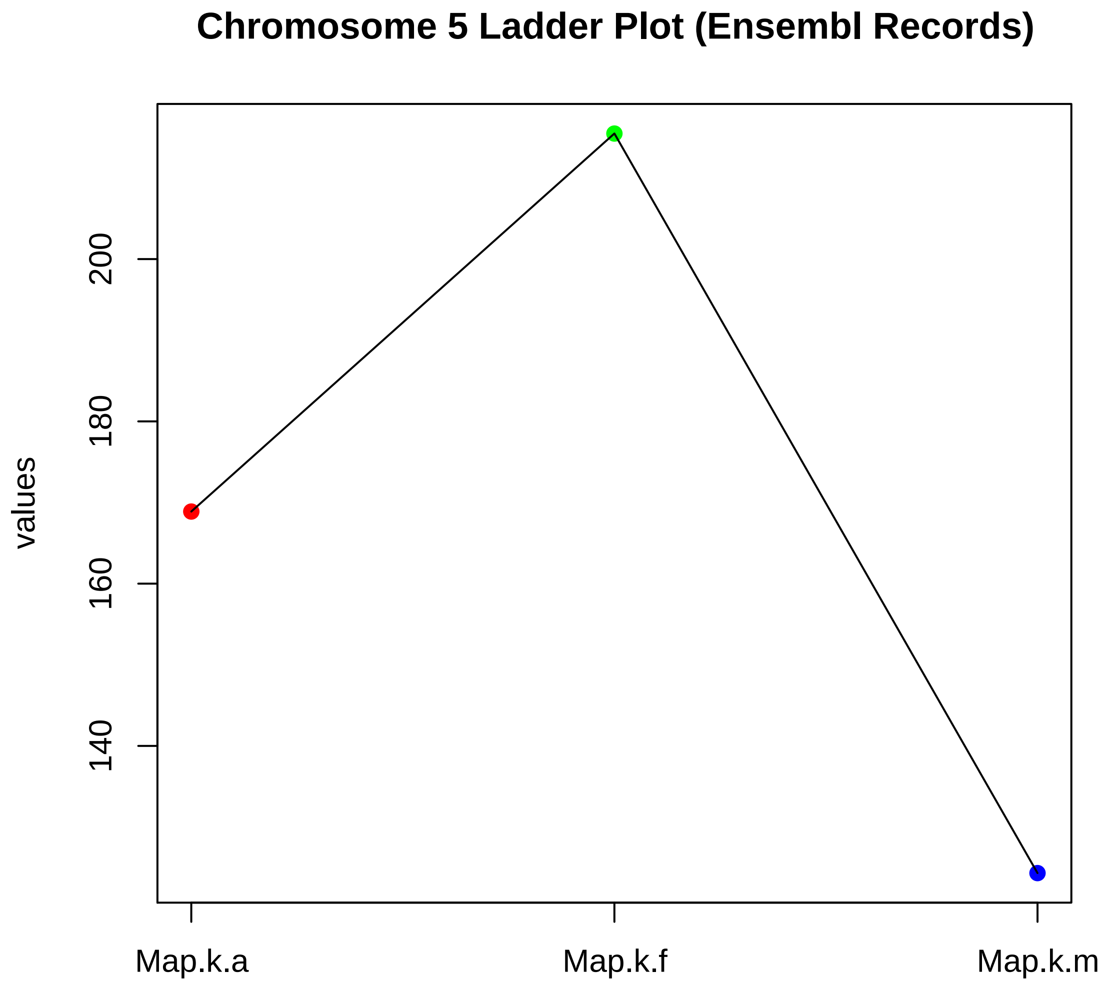
Chromosome 5 Ladder Plot (Ensembl Records)
values
140
160
180
200
Map.k.a
Map.k.f
Map.k.m

Chromosome 7 Genetic vs. Physical Positions (Ensembl Record

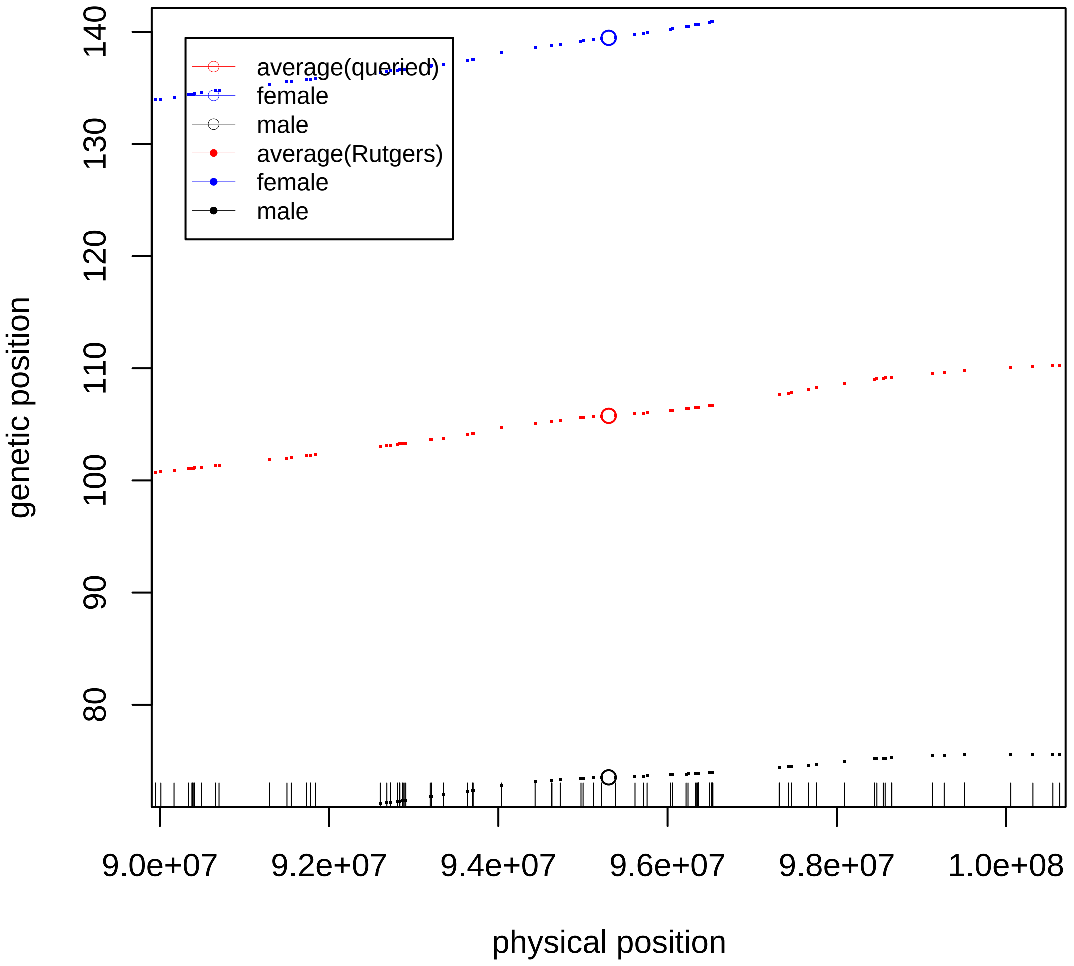

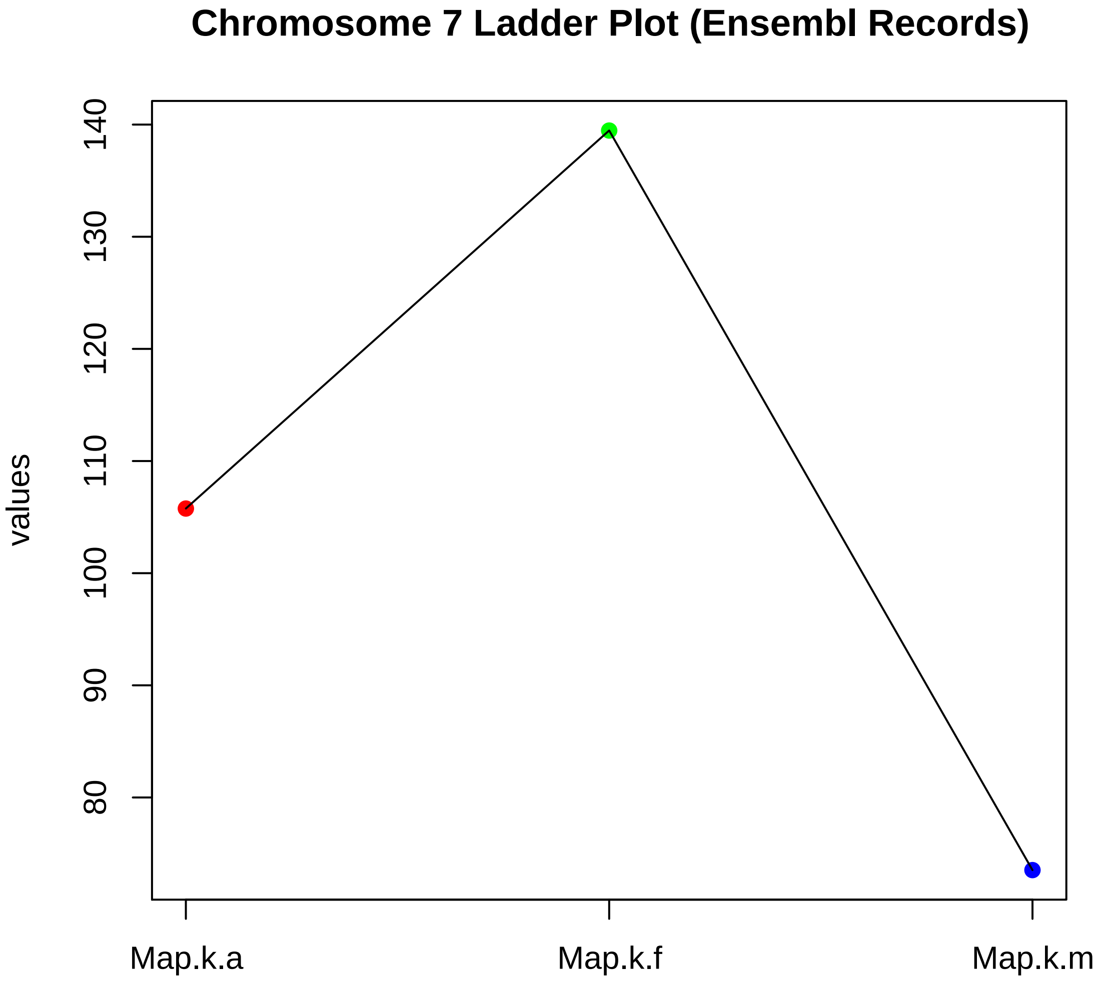
Chromosome 7 Ladder Plot (Ensembl Records)
values
140
130
120
110
100
90
80
Map.k.a
Map.k.f
Map.k.m

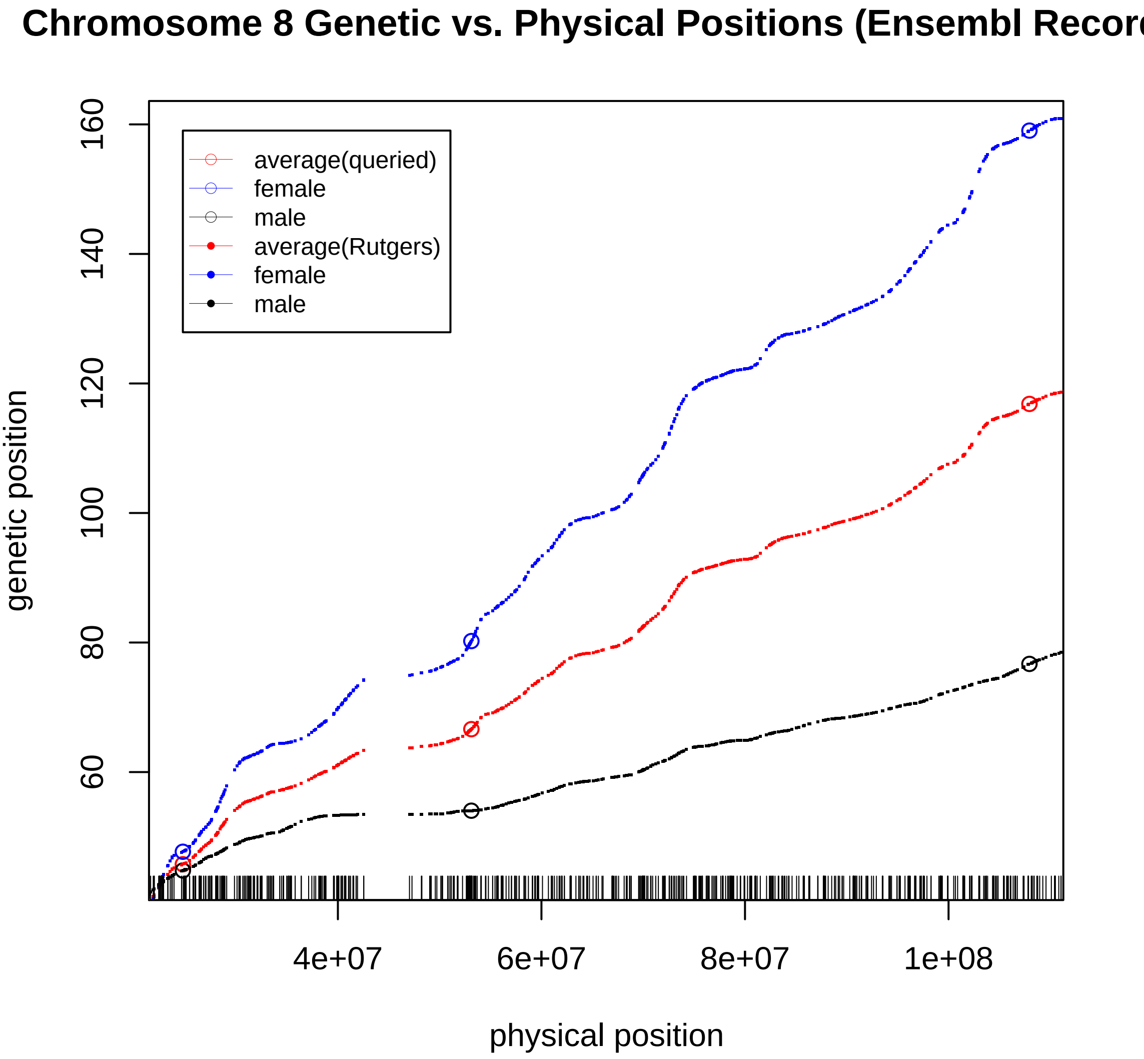

Chromosome 8 Genetic vs. Physical Positions (Ensembl Record
average(queried)
female
male
average(Rutgers)
female
male
genetic position
160
140
120
100
80
60
4e+07
6e+07
8e+07
1e+08
physical position

Chromosome 8 Ladder Plot (Ensembl Records)

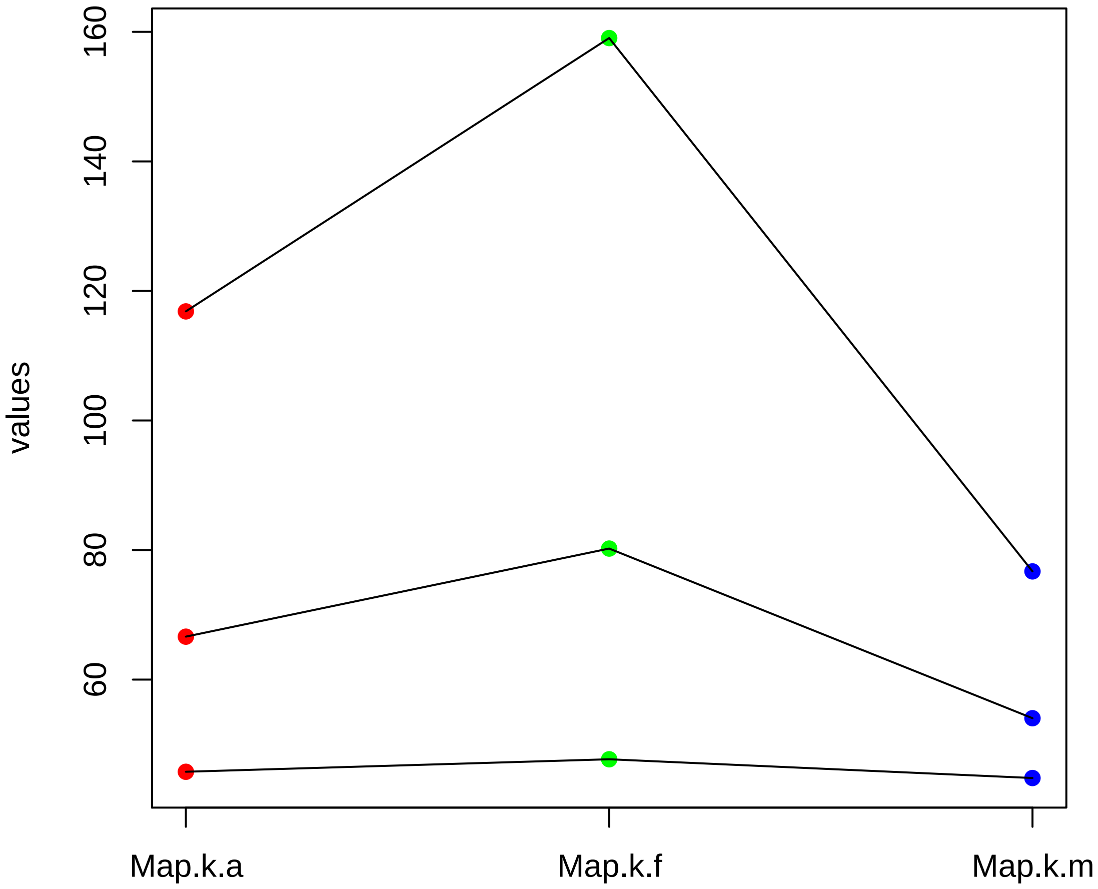

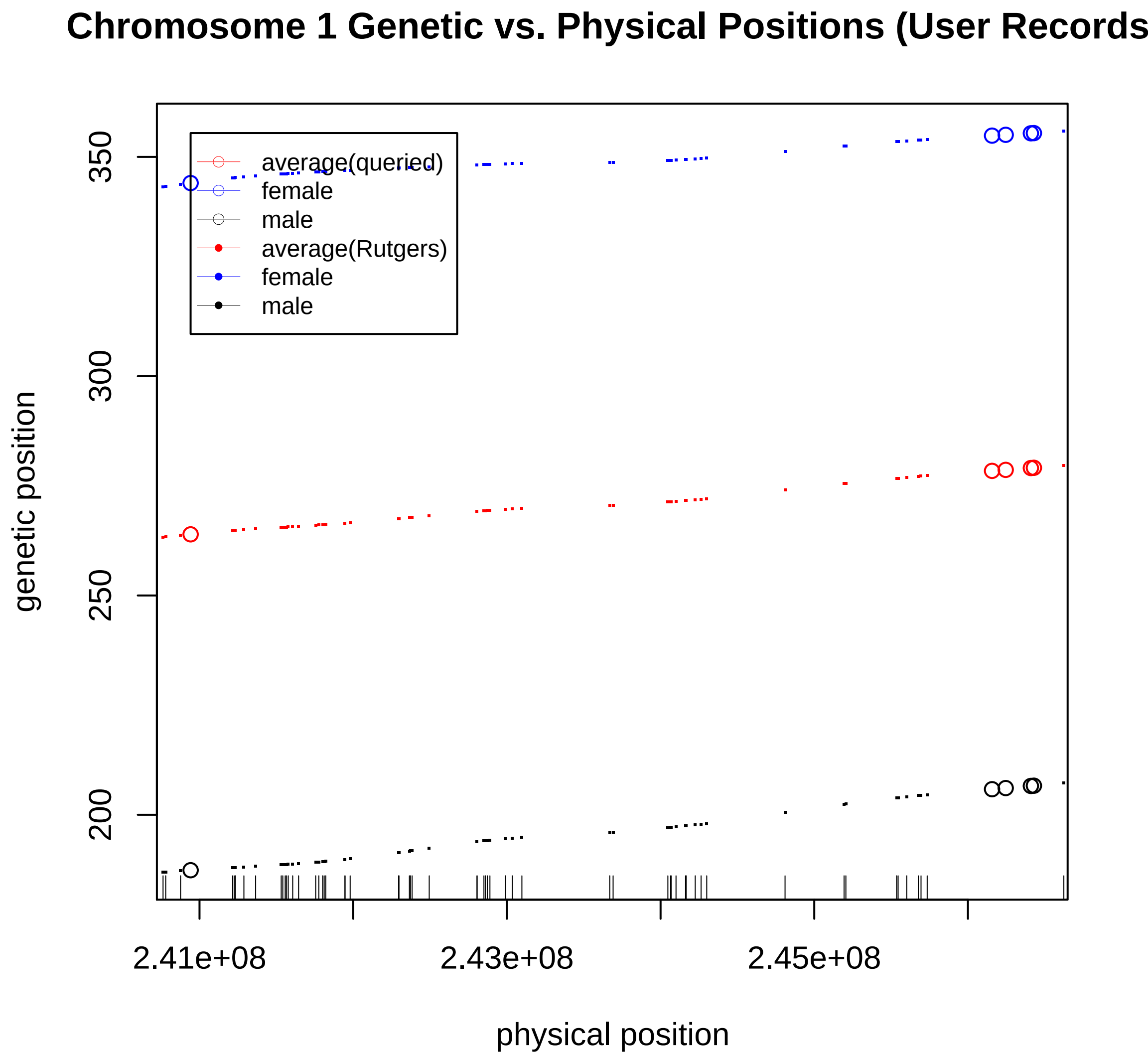
Chromosome 1 Genetic vs. Physical Positions (User Records
average(queried)
female
male
average(Rutgers)
female
male
genetic position
350
300
250
200
2.41e+08
2.43e+08
2.45e+08
physical position

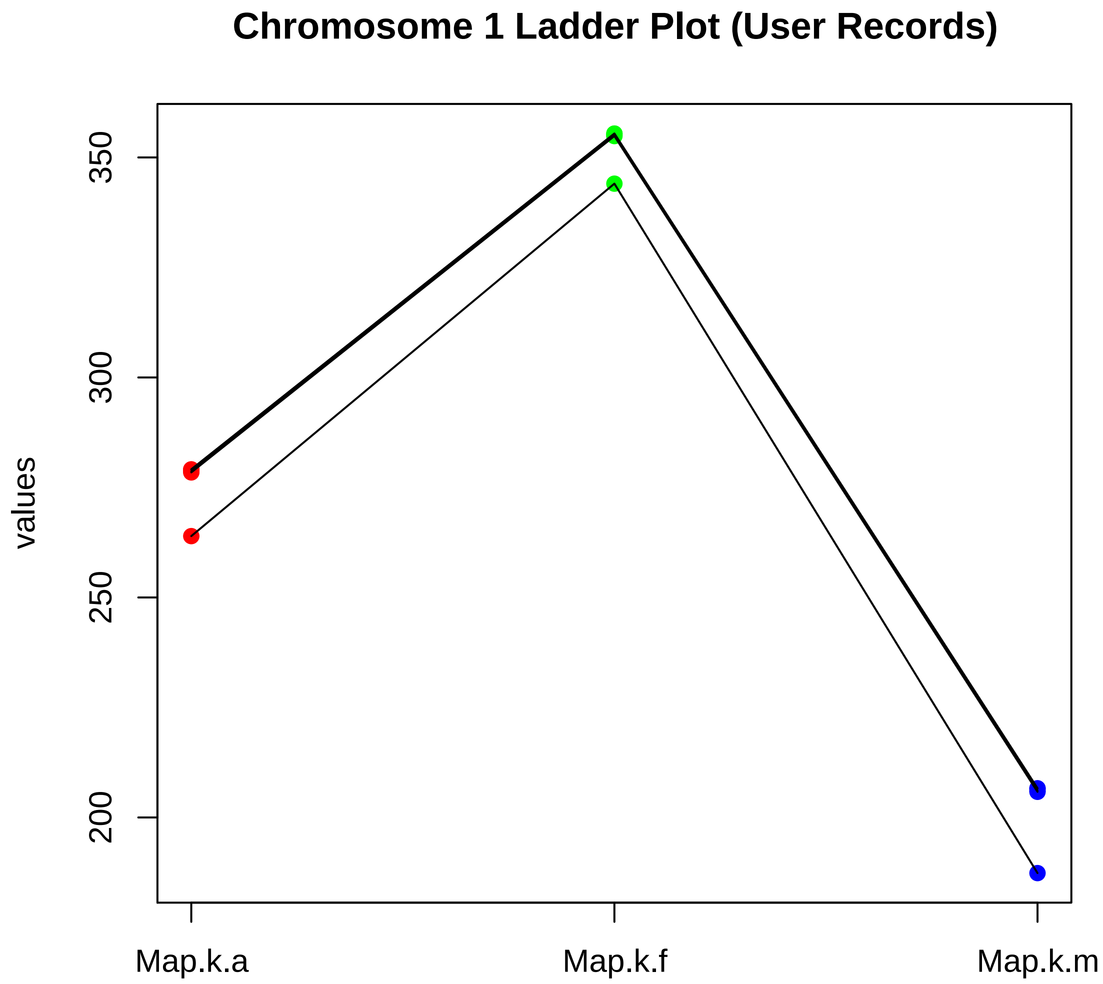

Chromosome 1 Ladder Plot (User Records)
values
350
300
250
200
Map.k.a
Map.k.f
Map.k.m

Chromosome 14 Genetic vs. Physical Positions (User Records

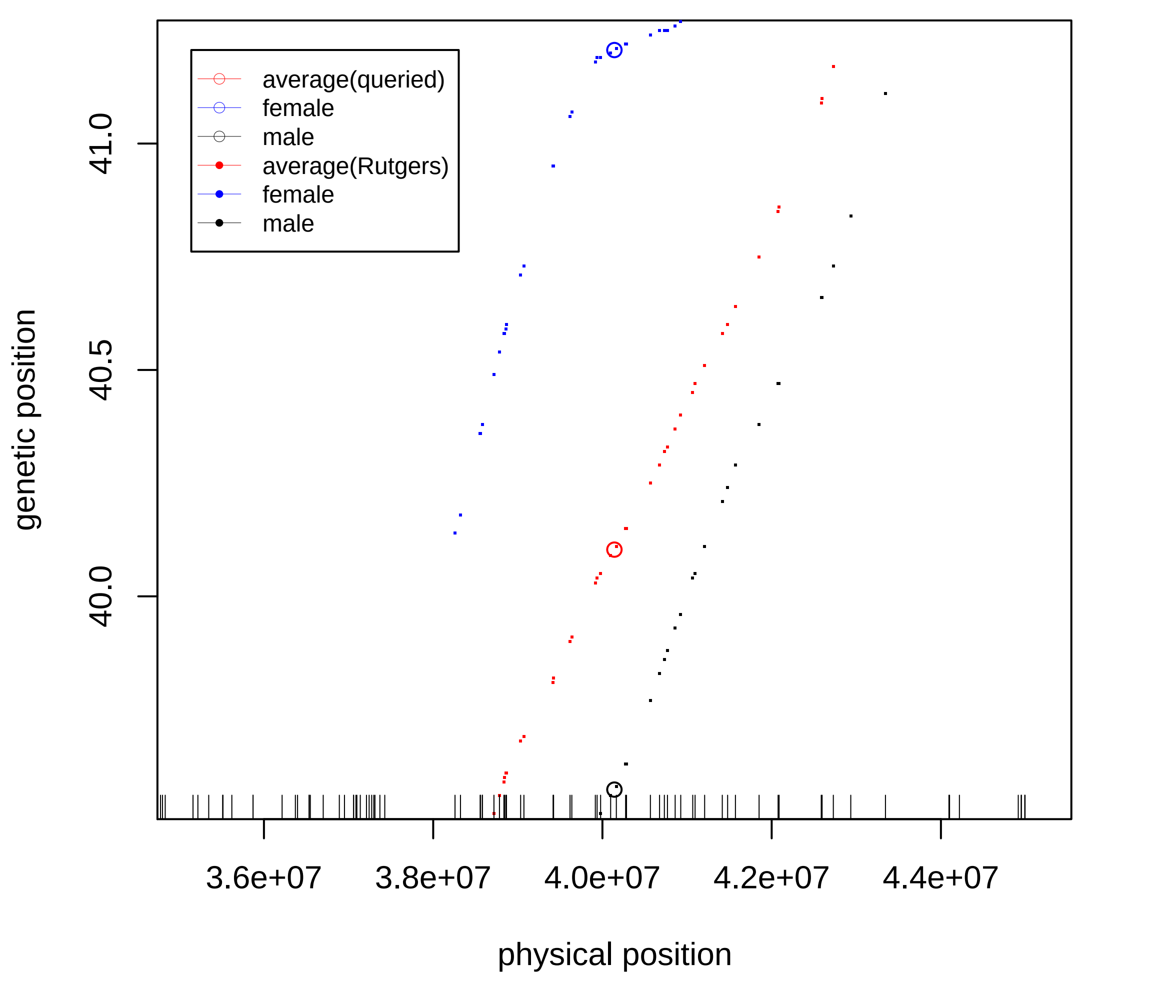

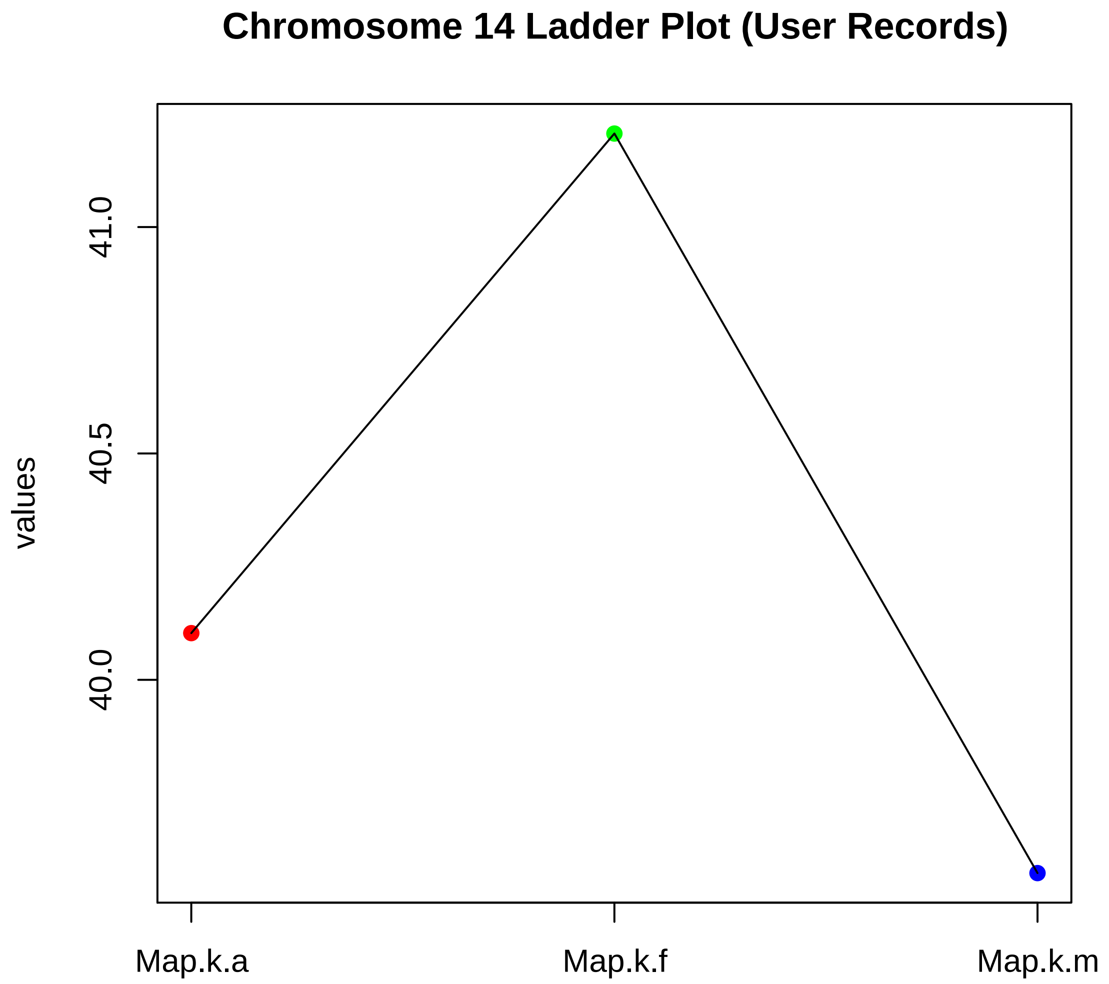
Chromosome 14 Ladder Plot (User Records)
values
41.0
40.5
40.0
Map.k.a
Map.k.f
Map.k.m

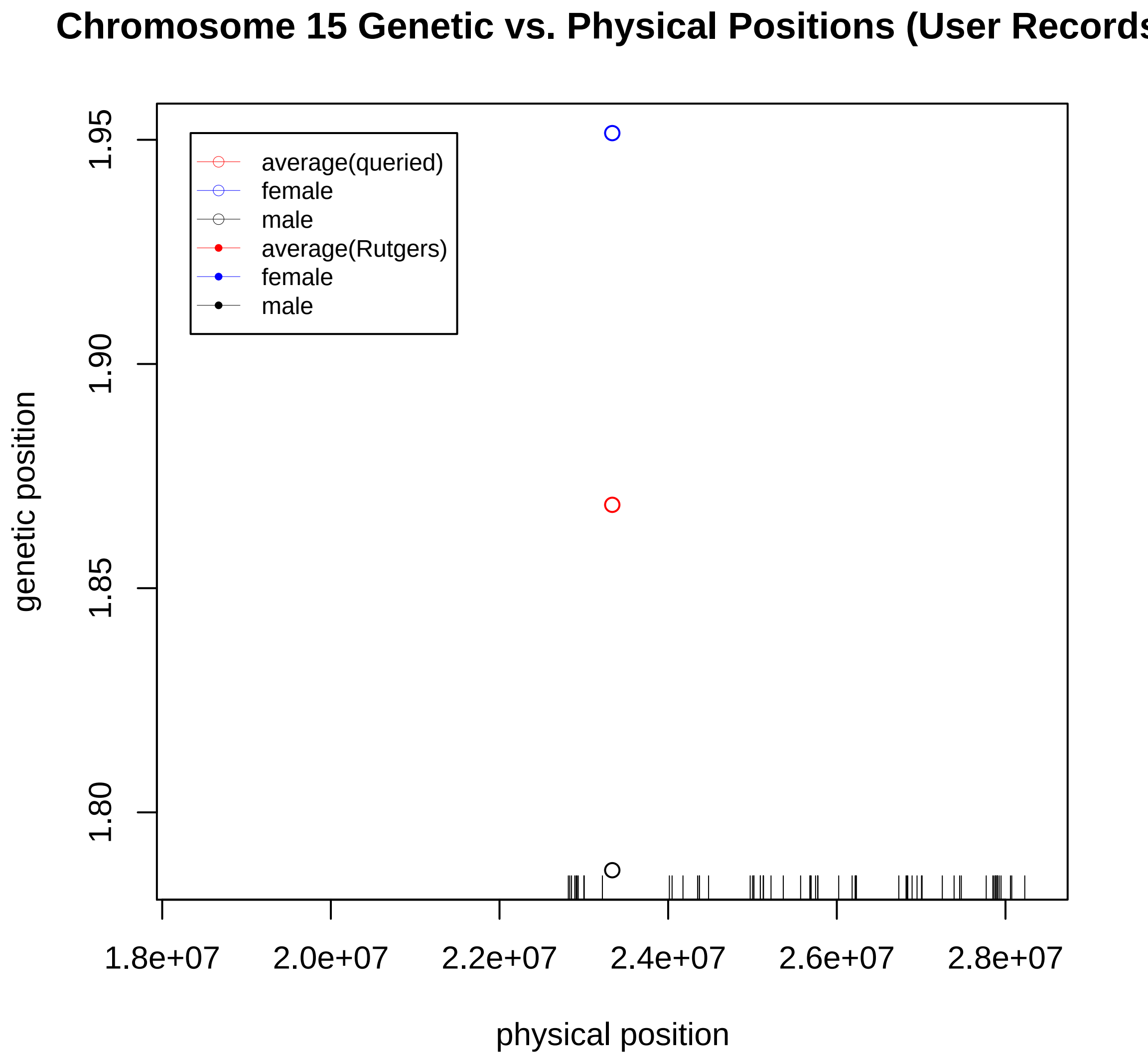

Chromosome 15 Genetic vs. Physical Positions (User Records
genetic position
1.80
1.85
1.90
1.95
average(queried)
female
male
average(Rutgers)
female
male
1.8e+07
2.0e+07
2.2e+07
2.4e+07
2.6e+07
2.8e+07
physical position

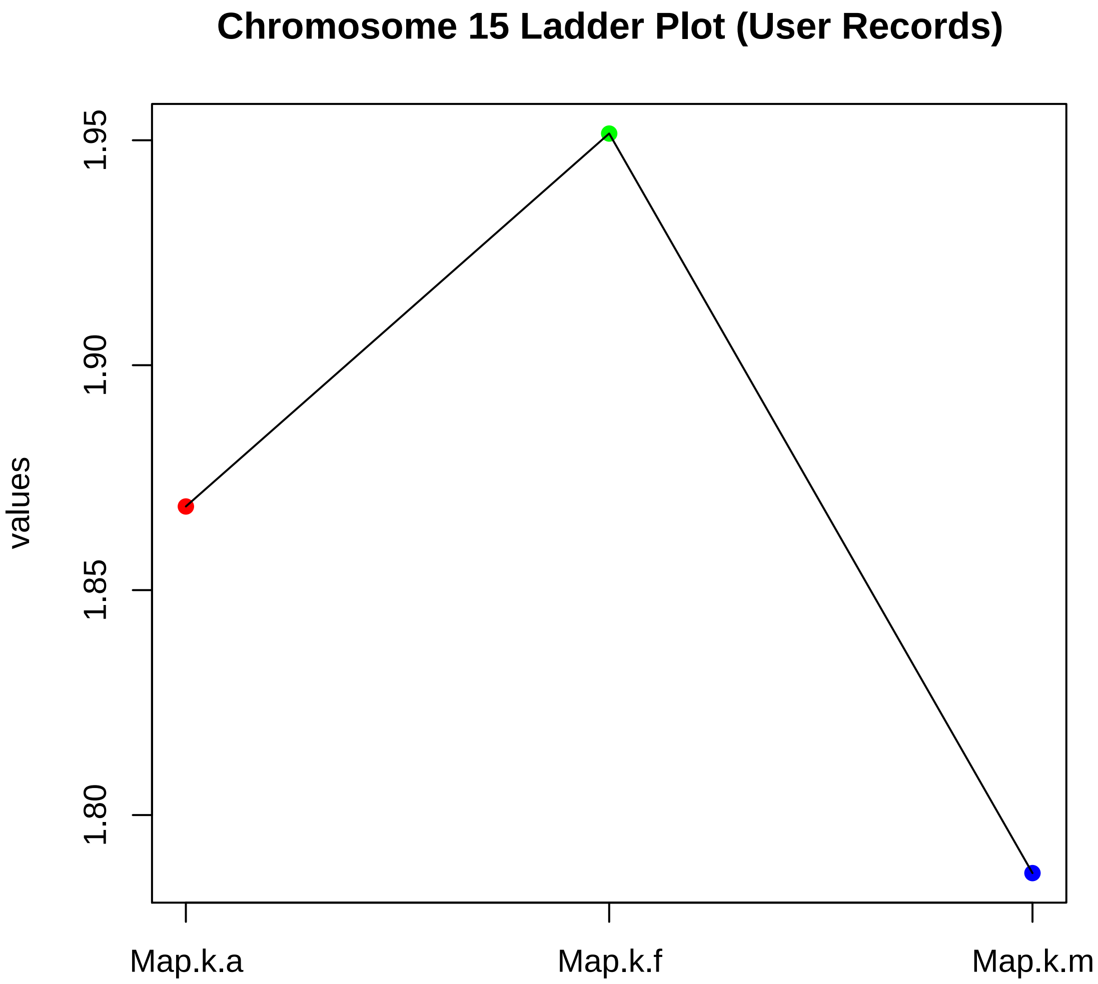

Chromosome 15 Ladder Plot (User Records)
values
1.95
1.90
1.85
1.80
Map.k.a
Map.k.f
Map.k.m

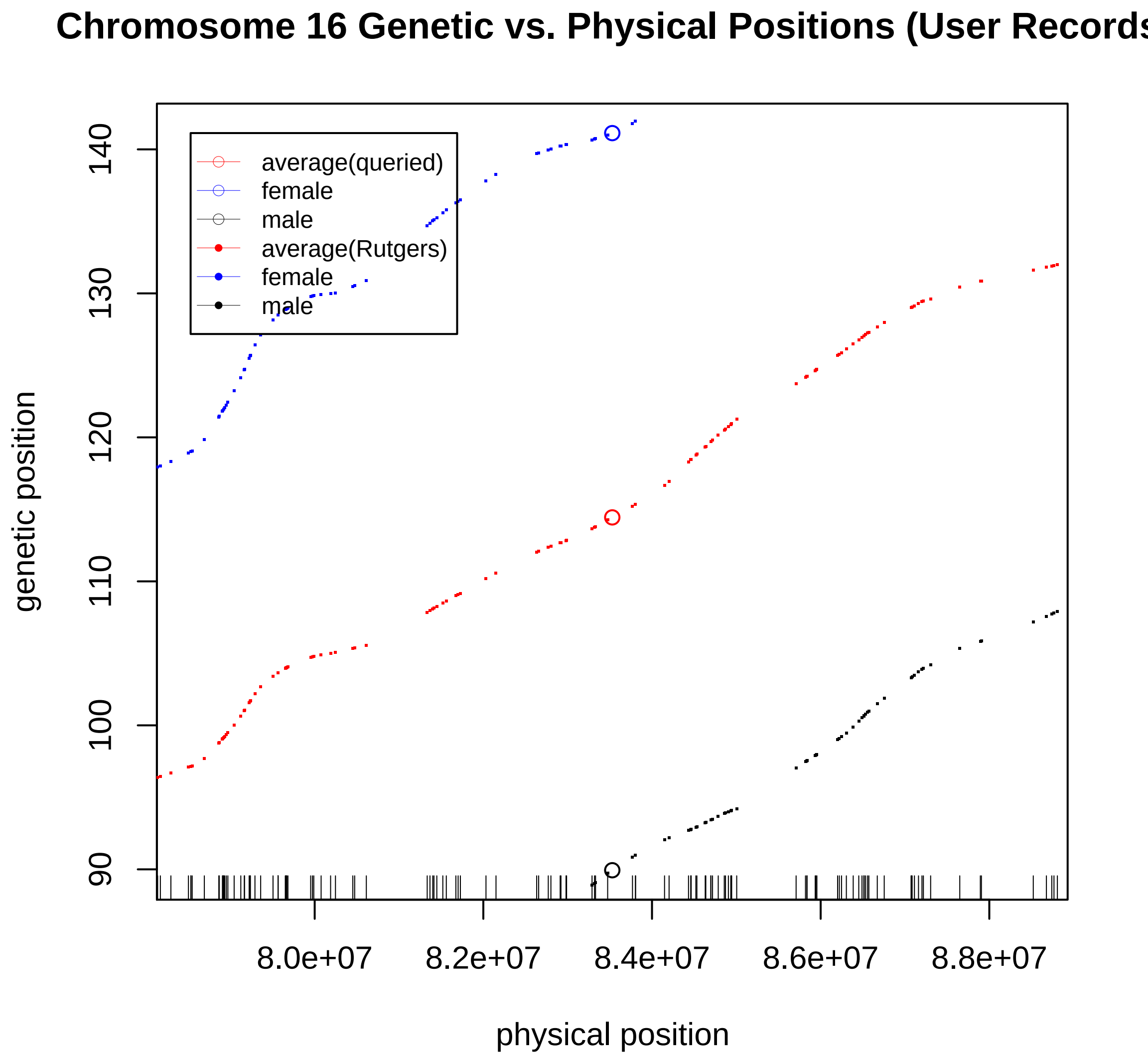
Chromosome 16 Genetic vs. Physical Positions (User Records
average(queried)
female
male
average(Rutgers)
female
male
genetic position
90
100
110
120
130
140
8.0e+07
8.2e+07
8.4e+07
8.6e+07
8.8e+07
physical position

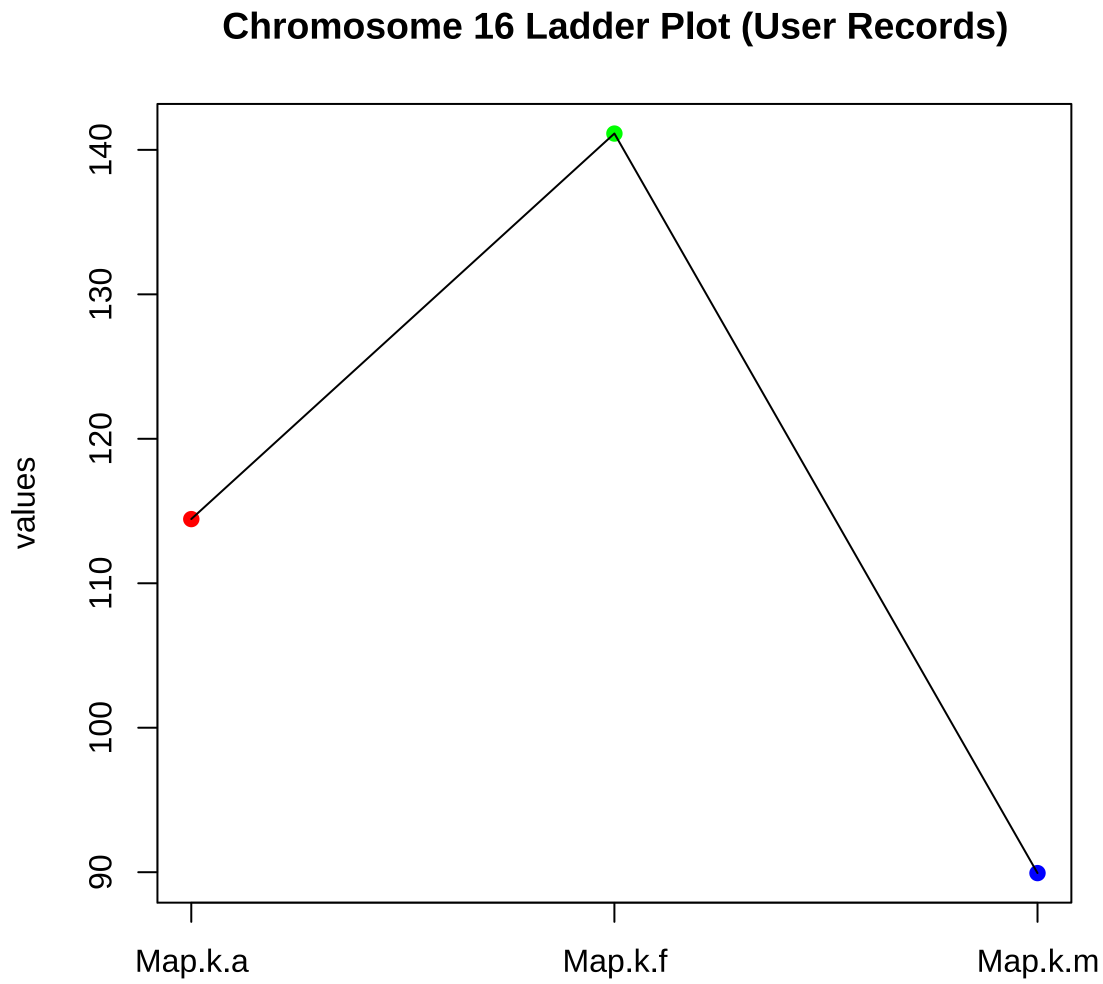

Chromosome 16 Ladder Plot (User Records)
values
140
130
120
110
100
90
Map.k.a
Map.k.f
Map.k.m

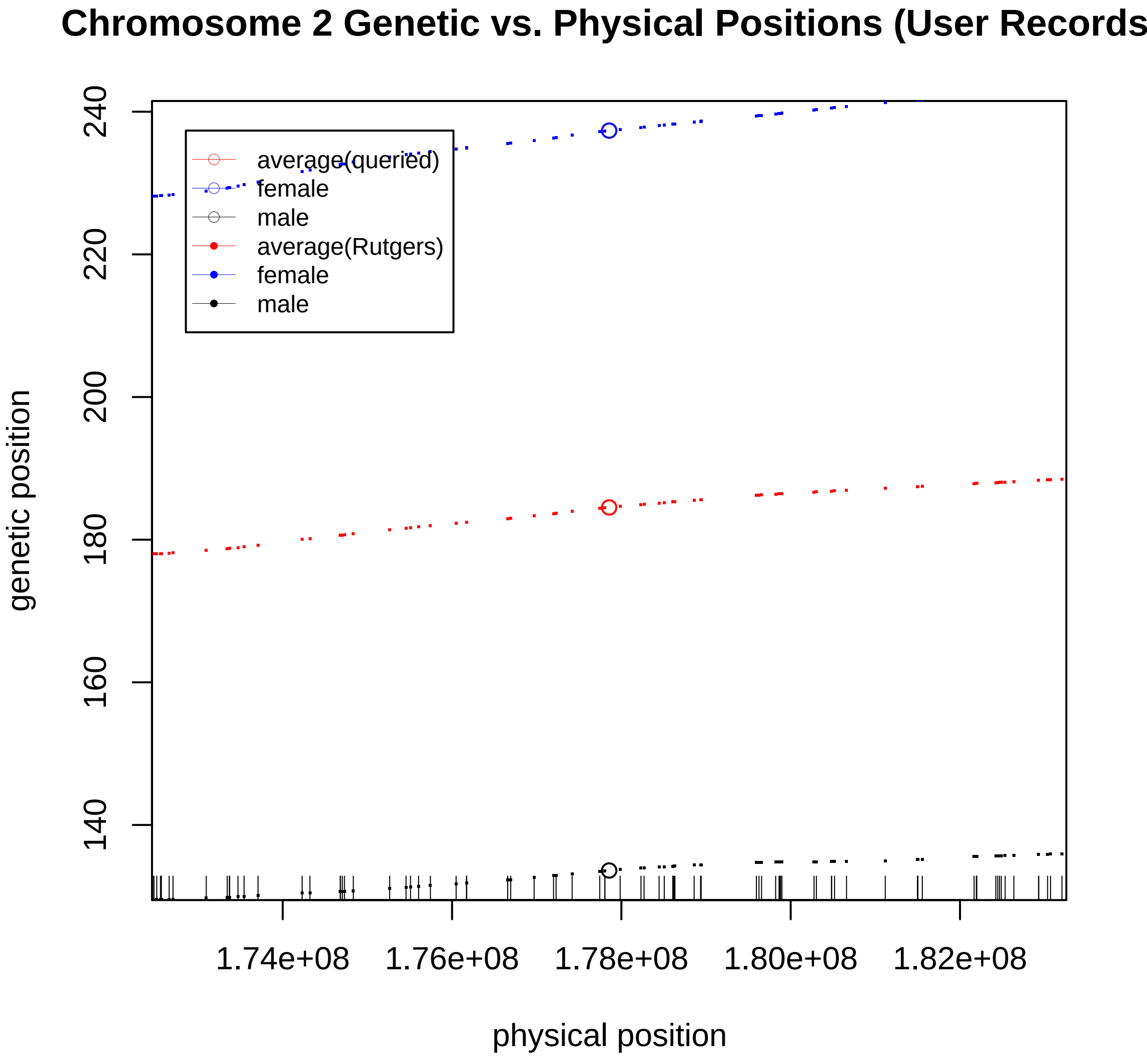

Chromosome 2 Genetic vs. Physical Positions (User Records
average(queried)
female
male
average(Rutgers)
female
male
genetic position
physical position
140
160
180
200
220
240
1.74e+08
1.76e+08
1.78e+08
1.80e+08
1.82e+08

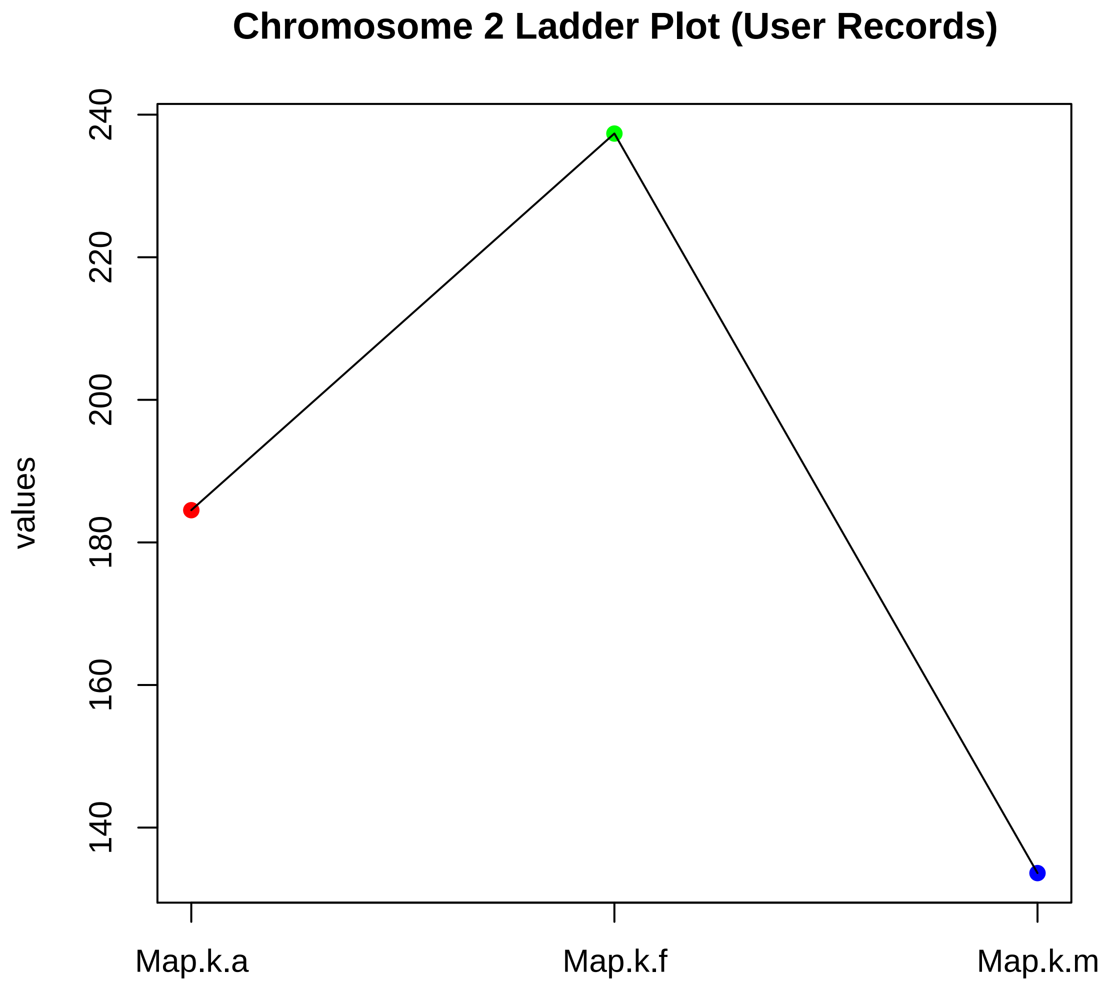
Chromosome 2 Ladder Plot (User Records)
values
240
220
200
180
160
140
Map.k.a
Map.k.f
Map.k.m

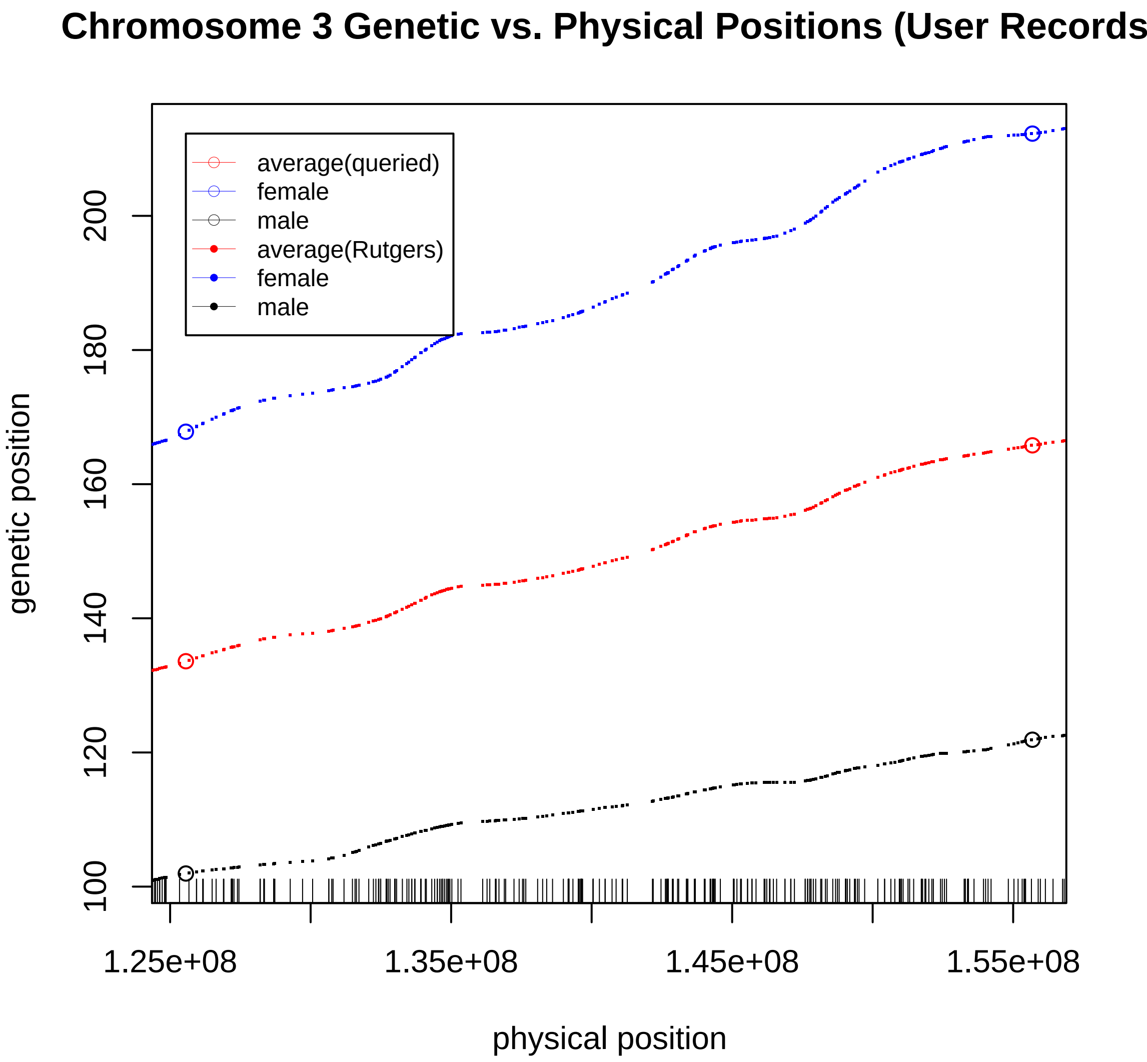

Chromosome 3 Genetic vs. Physical Positions (User Records
average(queried)
female
male
average(Rutgers)
female
male
genetic position
100
120
140
160
180
200
physical position
1.25e+08
1.35e+08
1.45e+08
1.55e+08

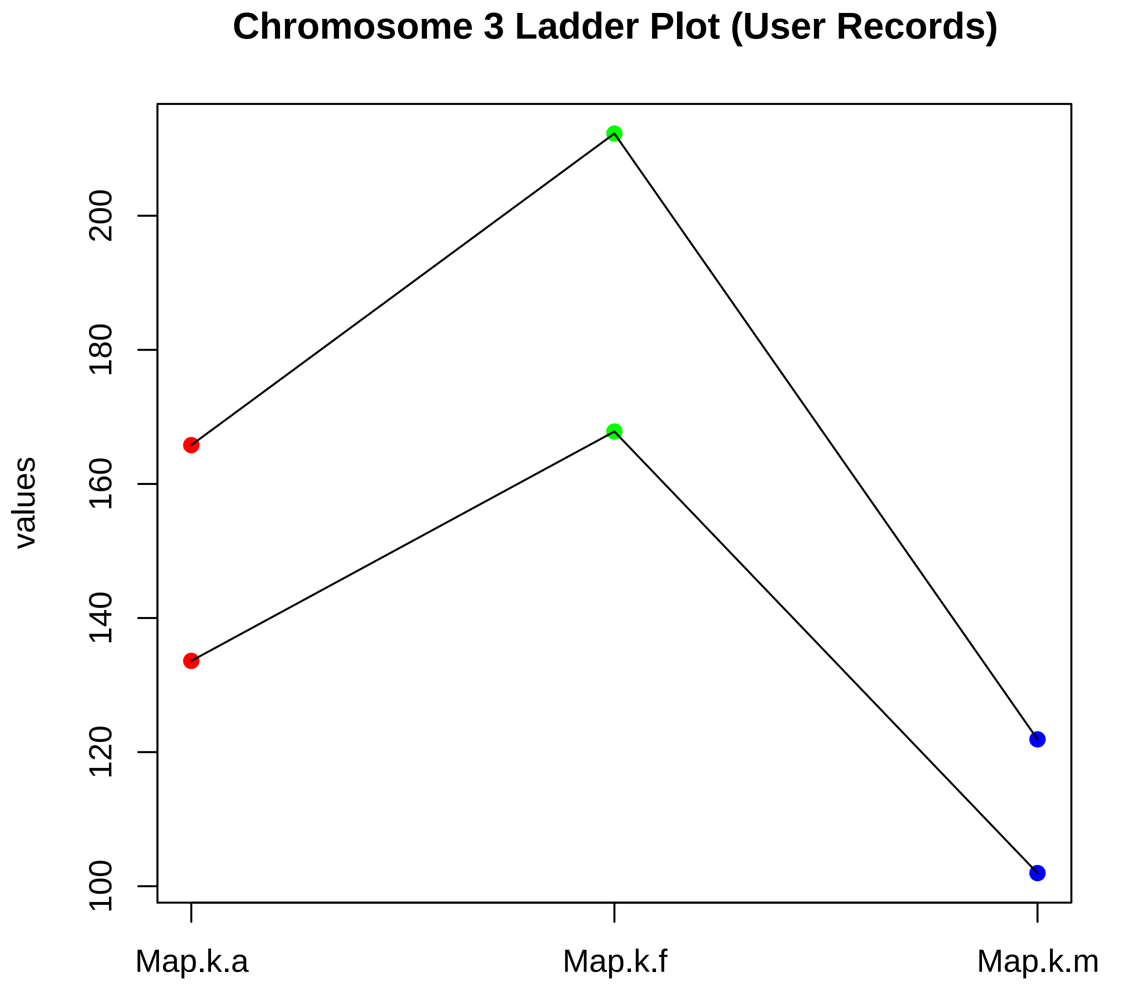

Chromosome 3 Ladder Plot (User Records)
values
100
120
140
160
180
200
Map.k.a
Map.k.f
Map.k.m

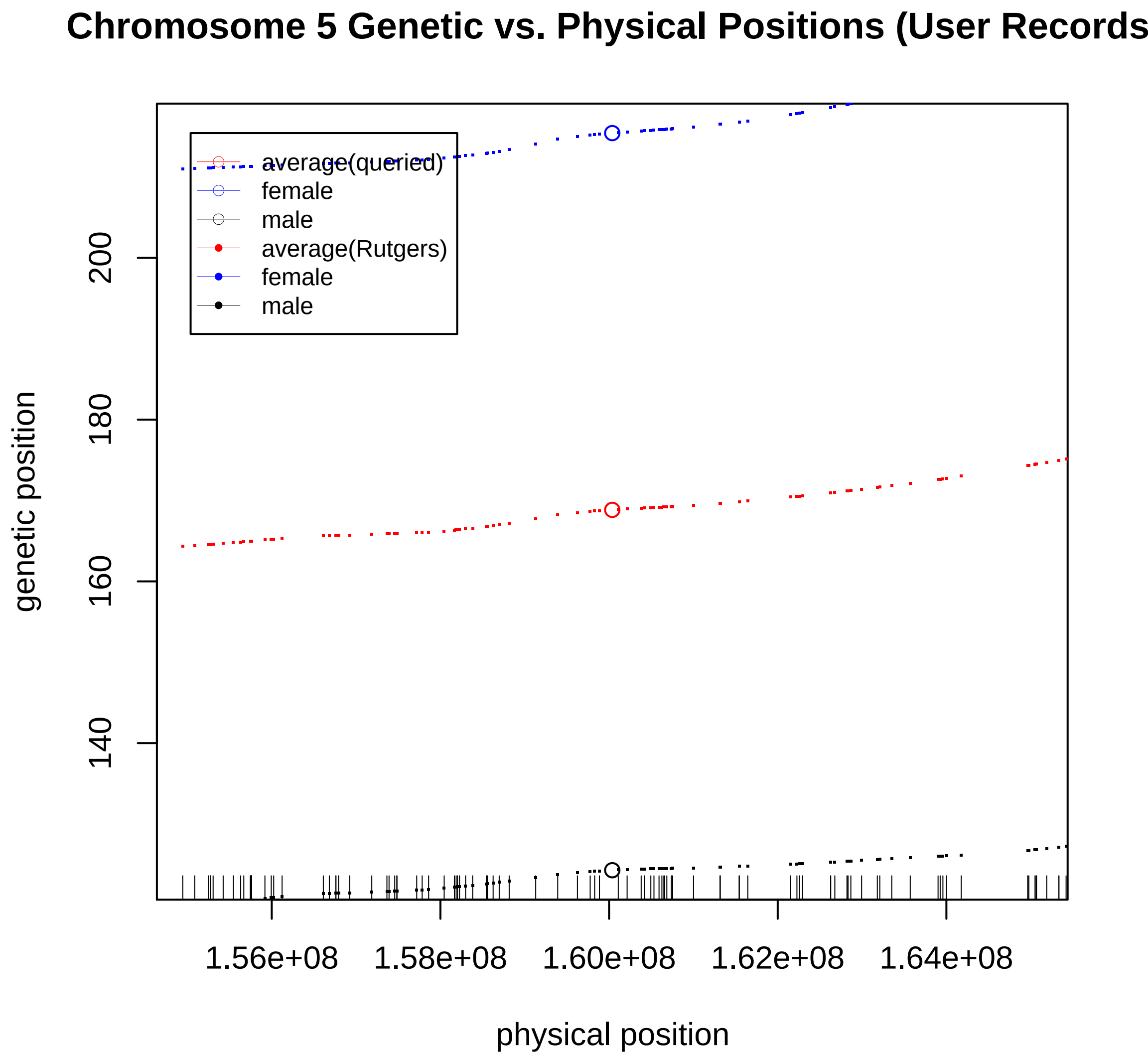

Chromosome 5 Genetic vs. Physical Positions (User Records
average(queried)
female
male
average(Rutgers)
female
male
genetic position
140
160
180
200
physical position
1.56e+08
1.58e+08
1.60e+08
1.62e+08
1.64e+08

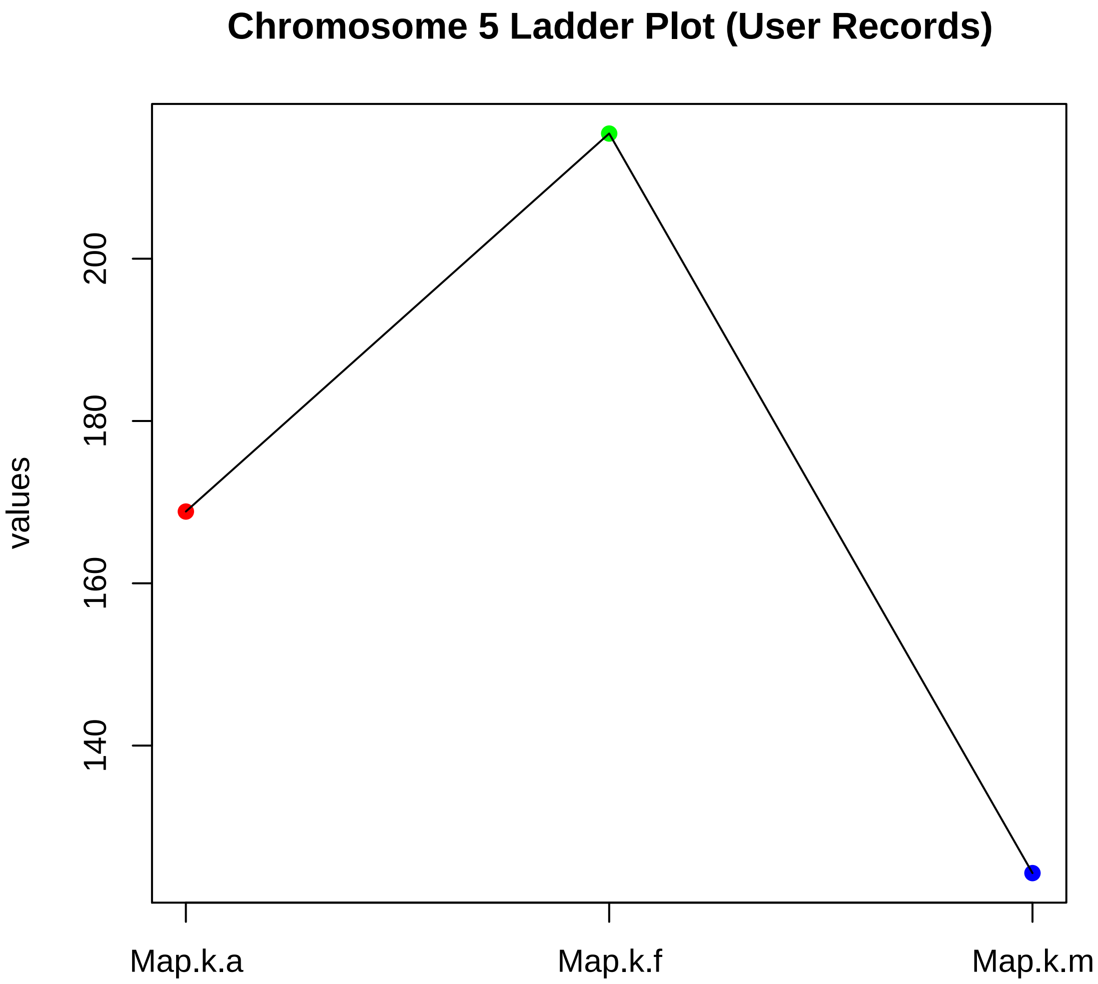
Chromosome 5 Ladder Plot (User Records)
values
200
180
160
140
Map.k.a
Map.k.f
Map.k.m

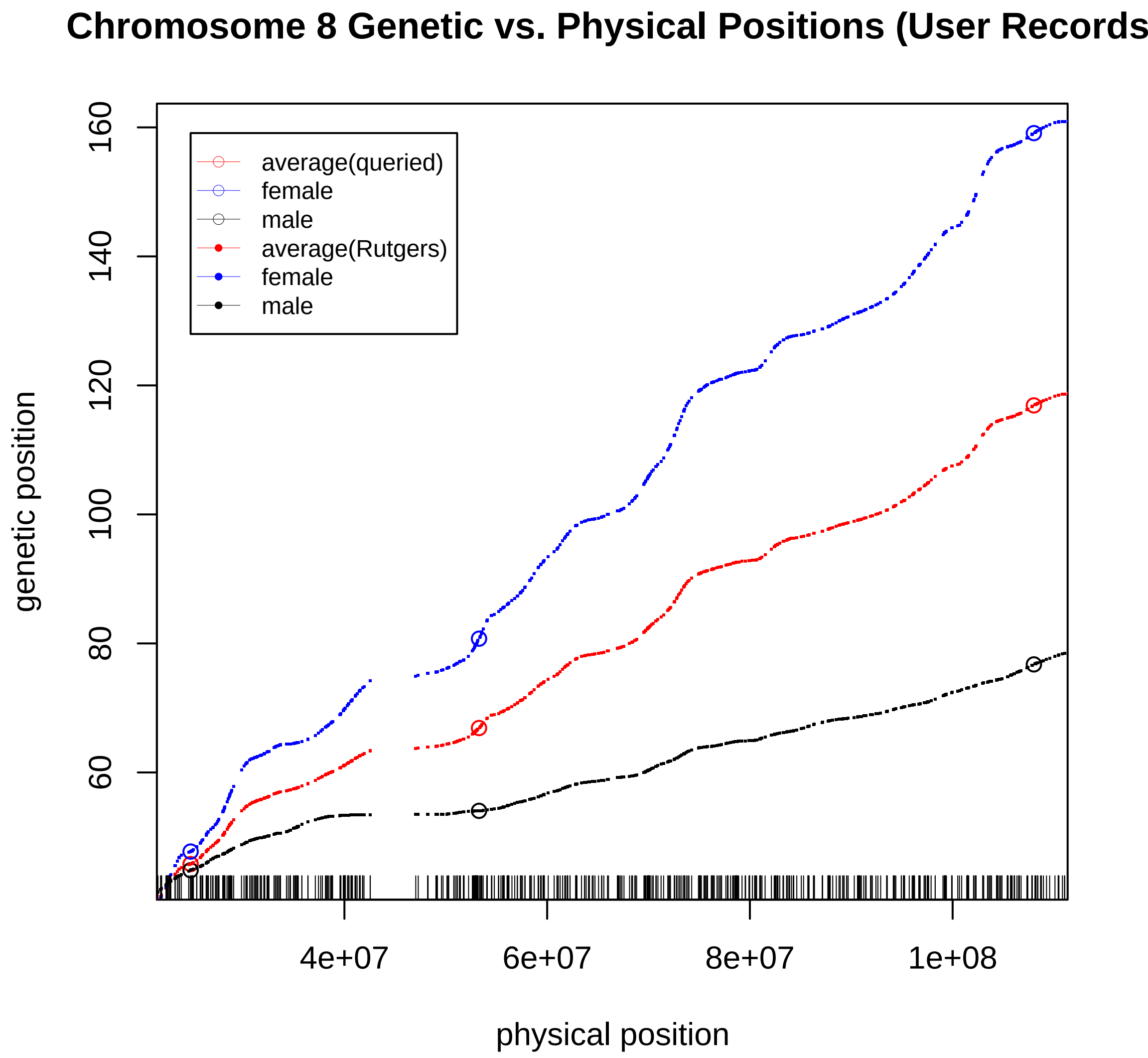

Chromosome 8 Genetic vs. Physical Positions (User Records
average(queried)
female
male
average(Rutgers)
female
male
genetic position
160
140
120
100
80
60
physical position
4e+07
6e+07
8e+07
1e+08

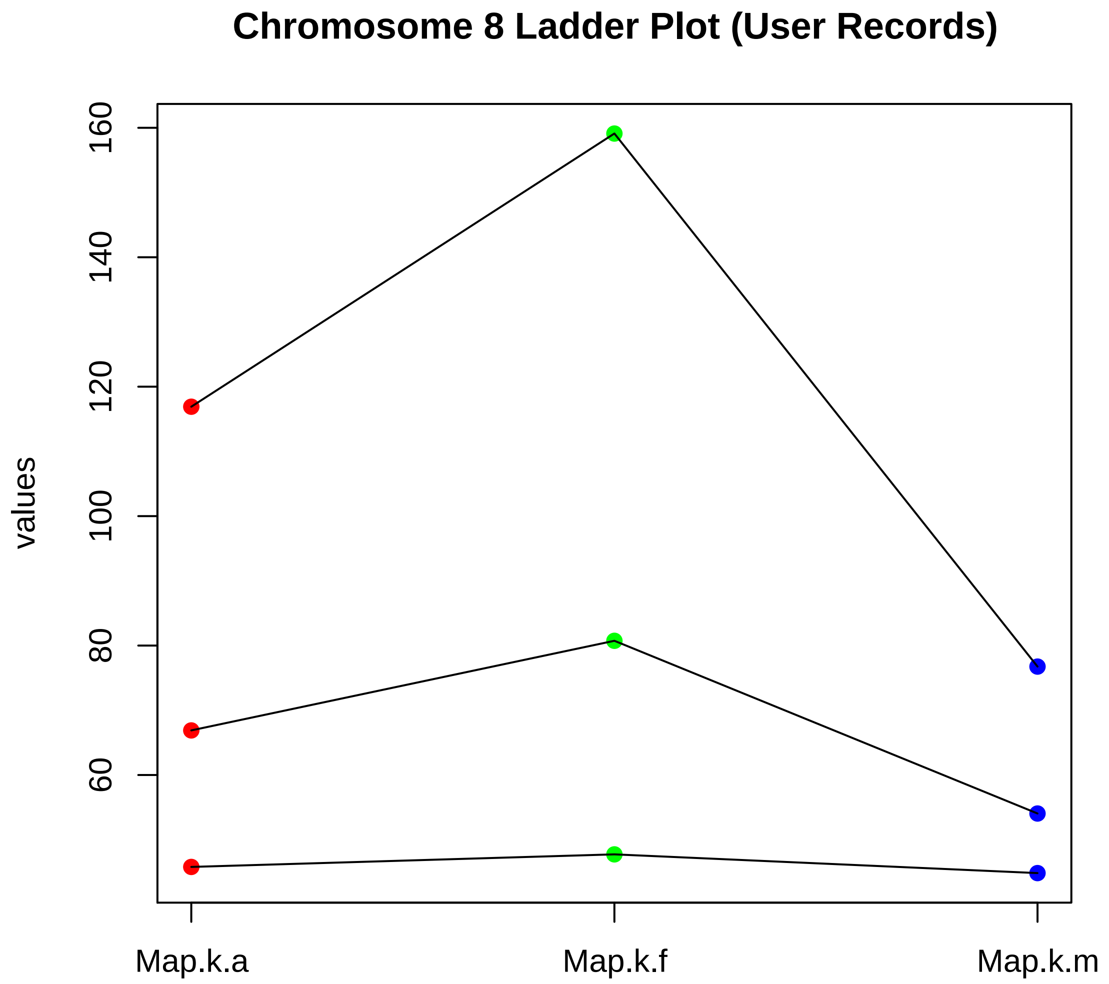

Chromosome 8 Ladder Plot (User Records)
values
160
140
120
100
80
60
Map.k.a
Map.k.f
Map.k.m

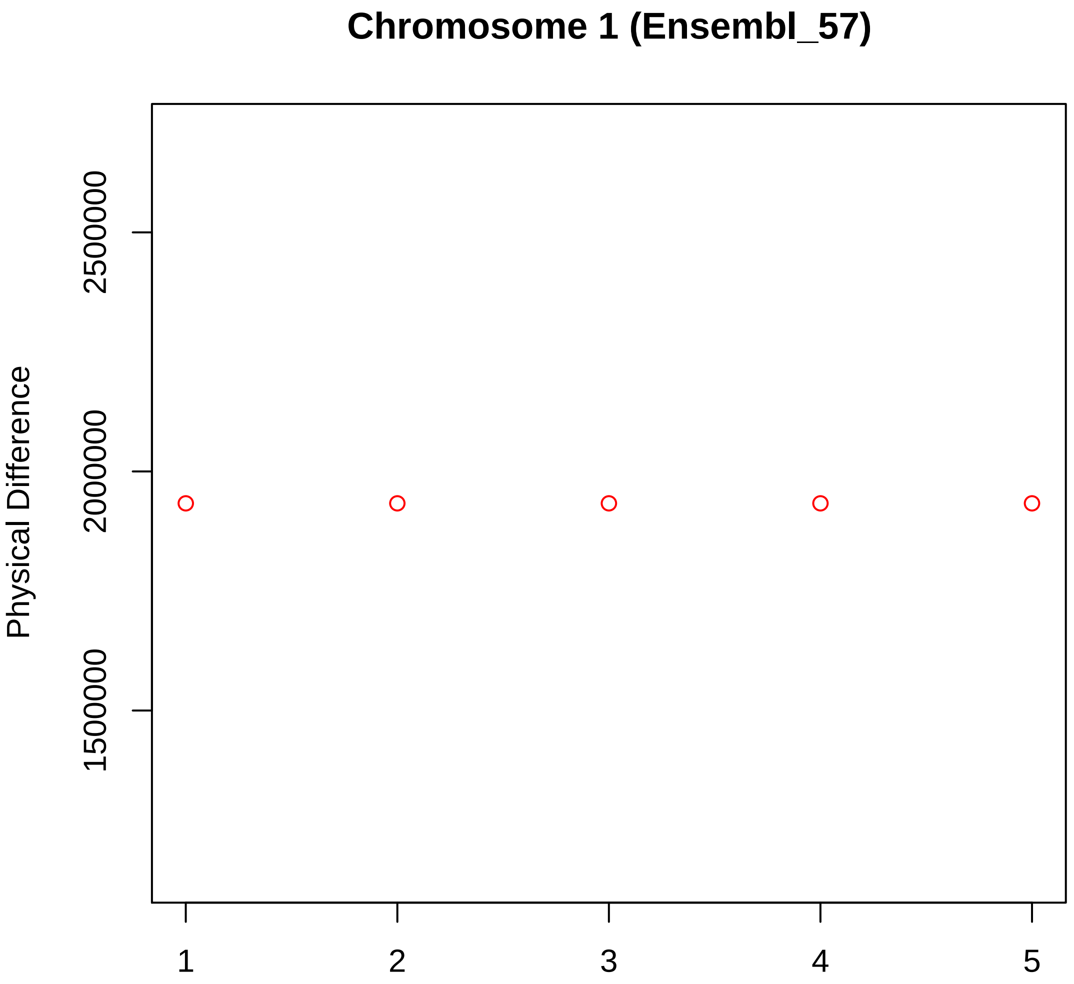
Chromosome 1 (Ensembl_57)
Physical Difference
25000000
20000000
15000000
1
2
3
4
5

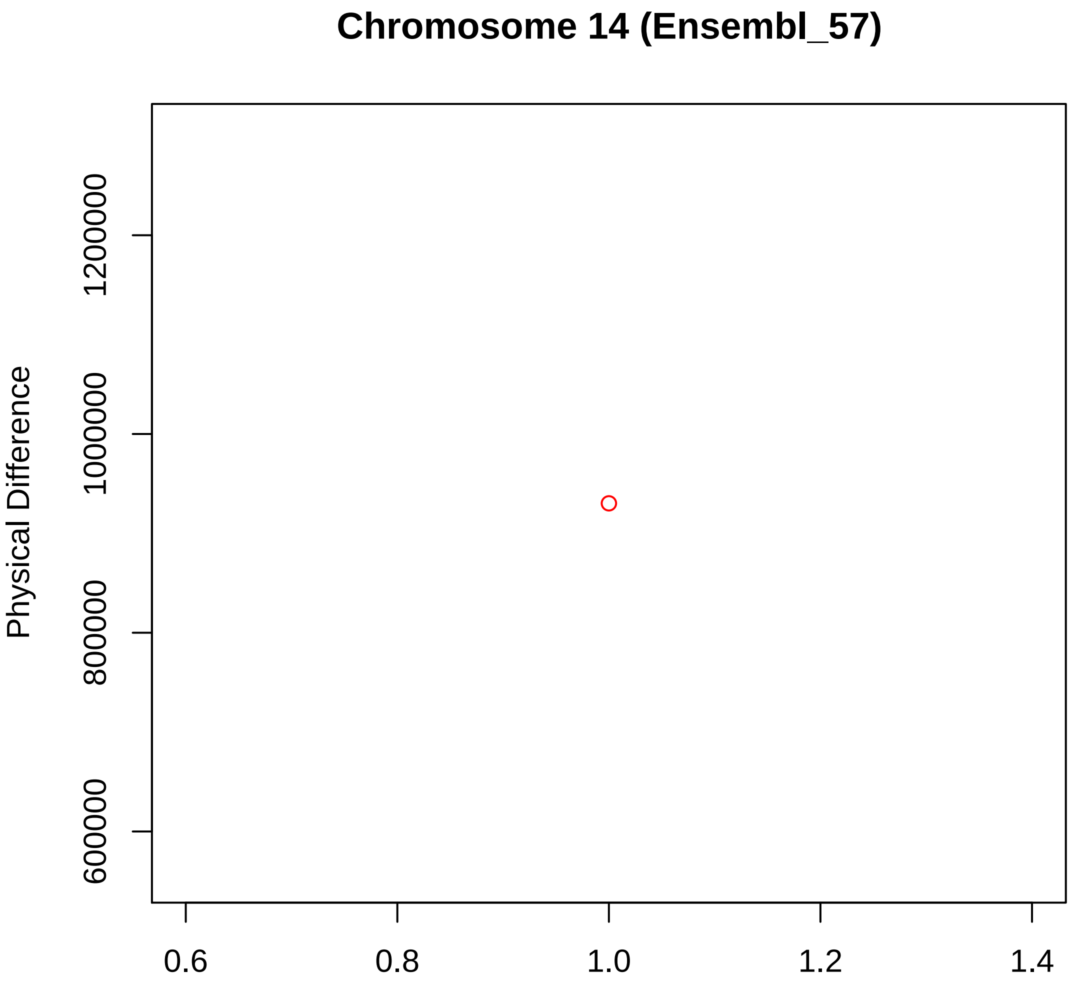
Chromosome 14 (Ensembl_57)
Physical Difference
600000
800000
10000000
12000000
0.6
0.8
1.0
1.2
1.4

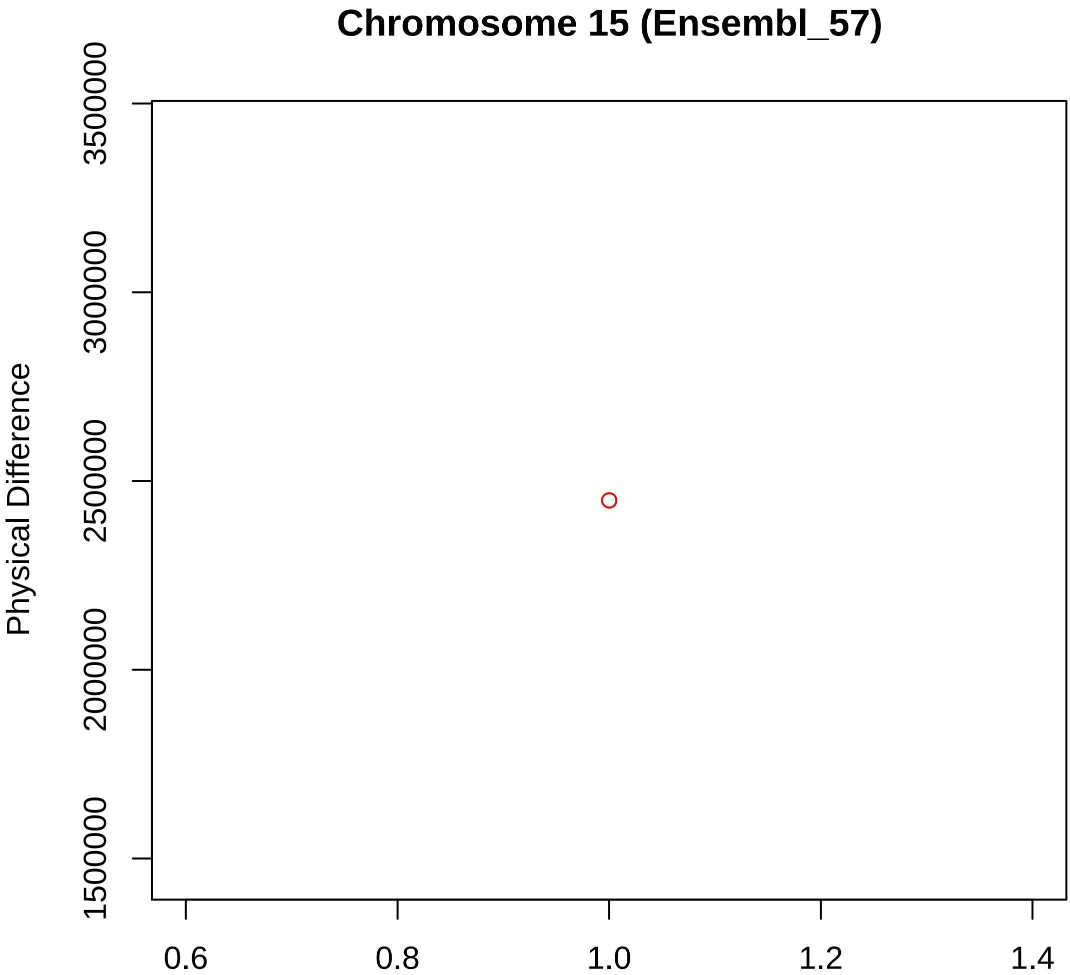
Chromosome 15 (Ensembl_57)
Physical Difference
15000000
20000000
25000000
30000000
35000000
0.6
0.8
1.0
1.2
1.4

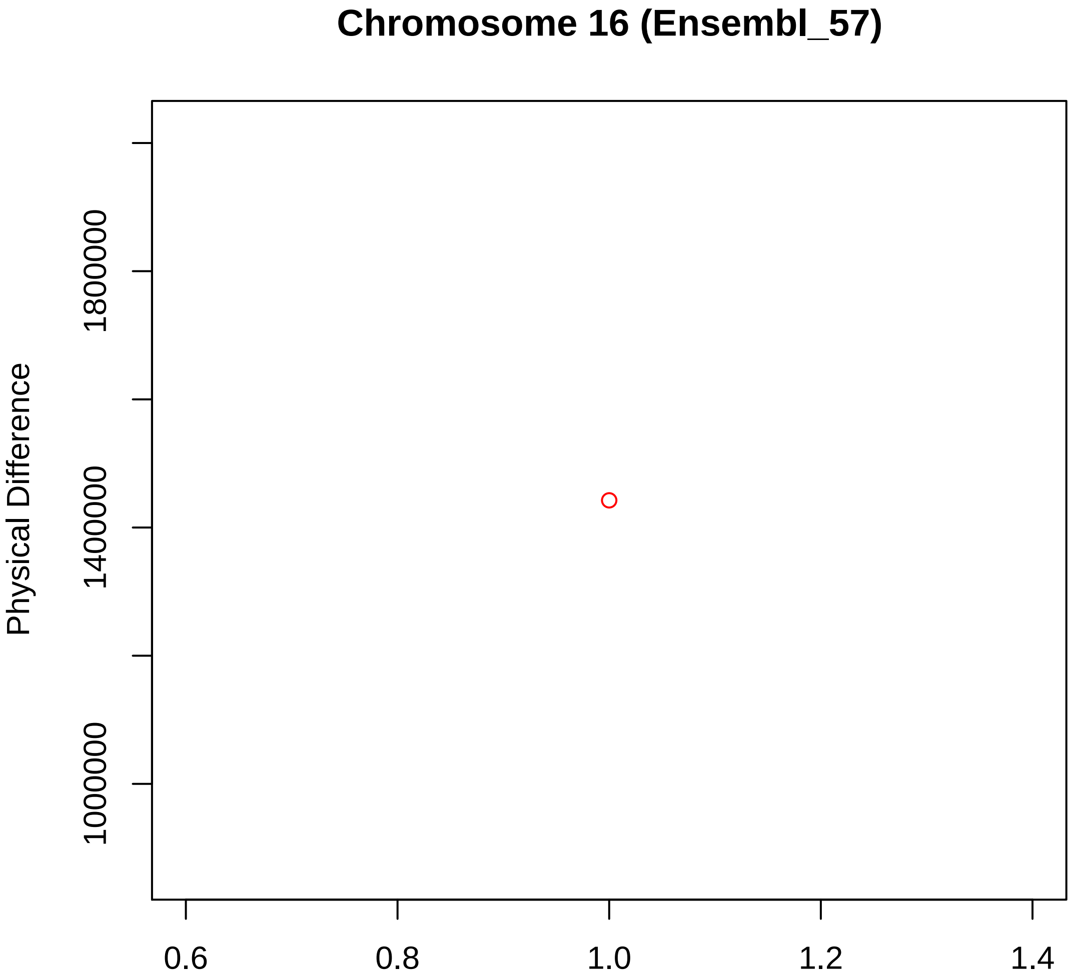
Chromosome 16 (Ensembl_57)
Physical Difference
10000000
14000000
18000000
0.6
0.8
1.0
1.2
1.4

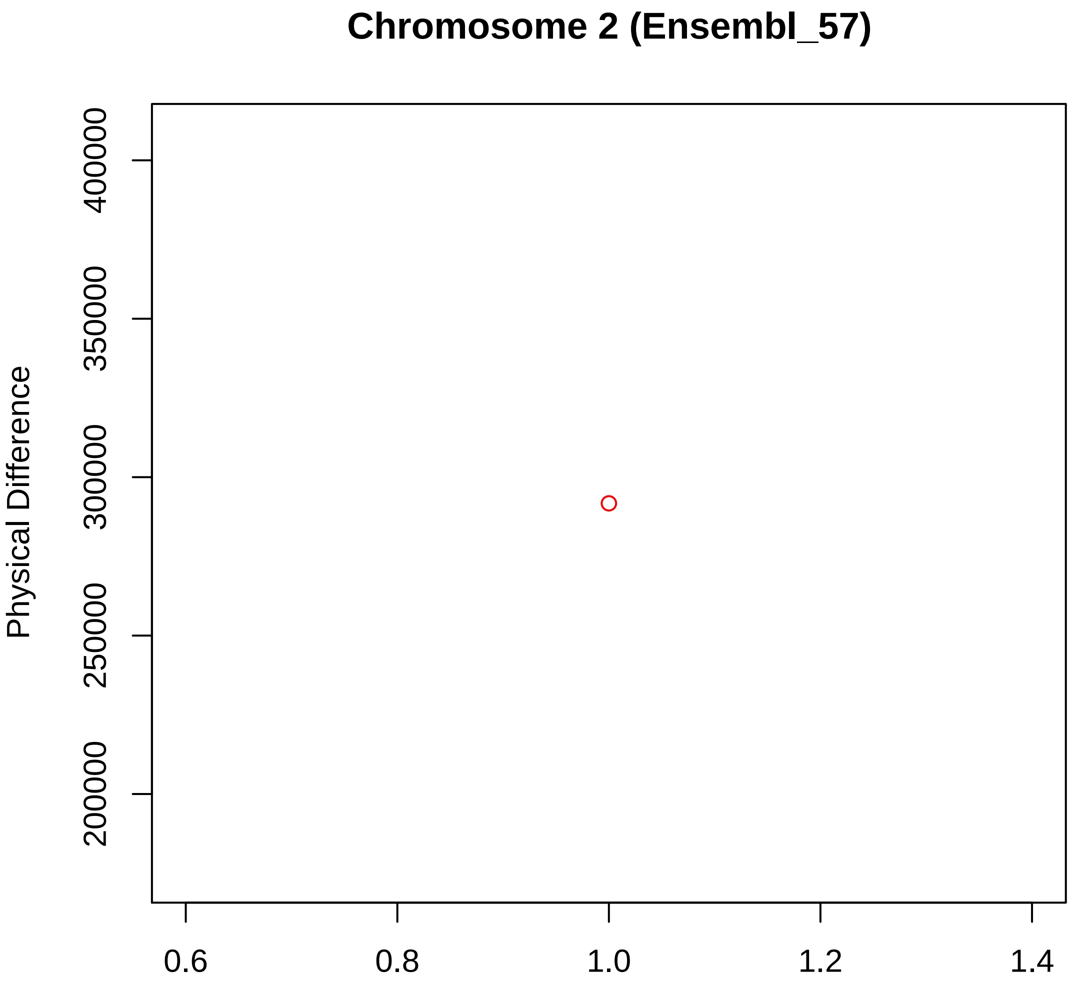
Chromosome 2 (Ensembl_57)
Physical Difference
4000000
3500000
3000000
2500000
2000000
0.6
0.8
1.0
1.2
1.4

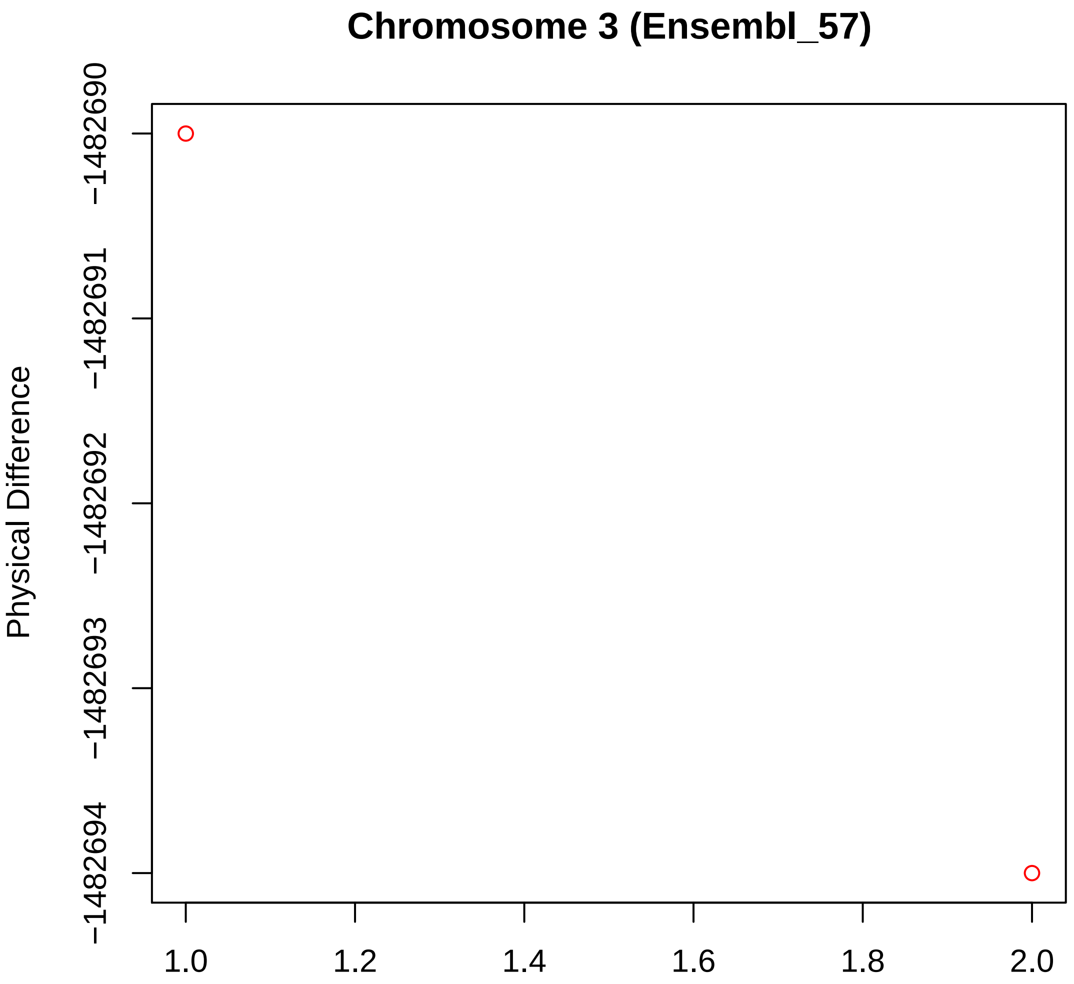

Chromosome 3 (Ensembl_57)
Physical Difference
−1482690
−1482691
−1482692
−1482693
−1482694
1.0
1.2
1.4
1.6
1.8
2.0

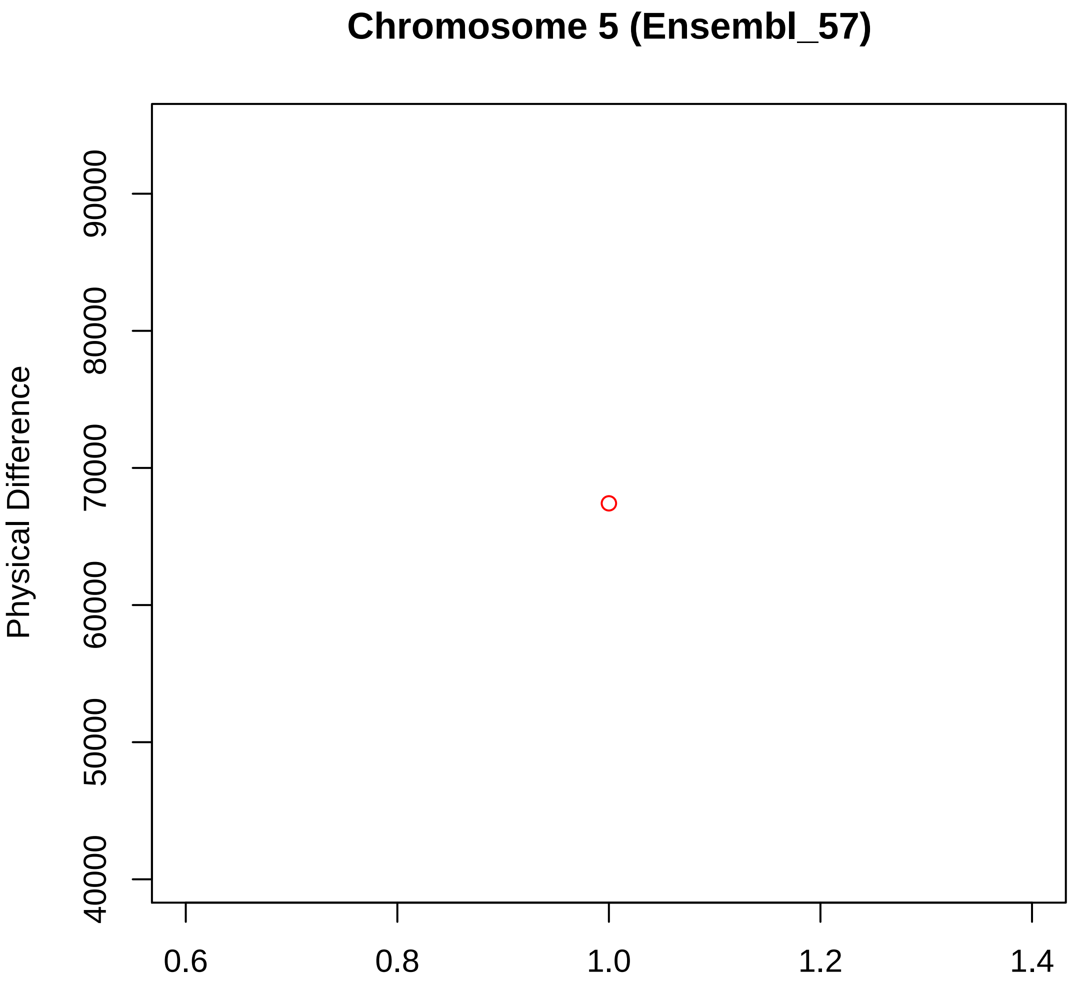
Chromosome 5 (Ensembl_57)
Physical Difference
40000
50000
60000
70000
80000
90000
0.6
0.8
1.0
1.2
1.4

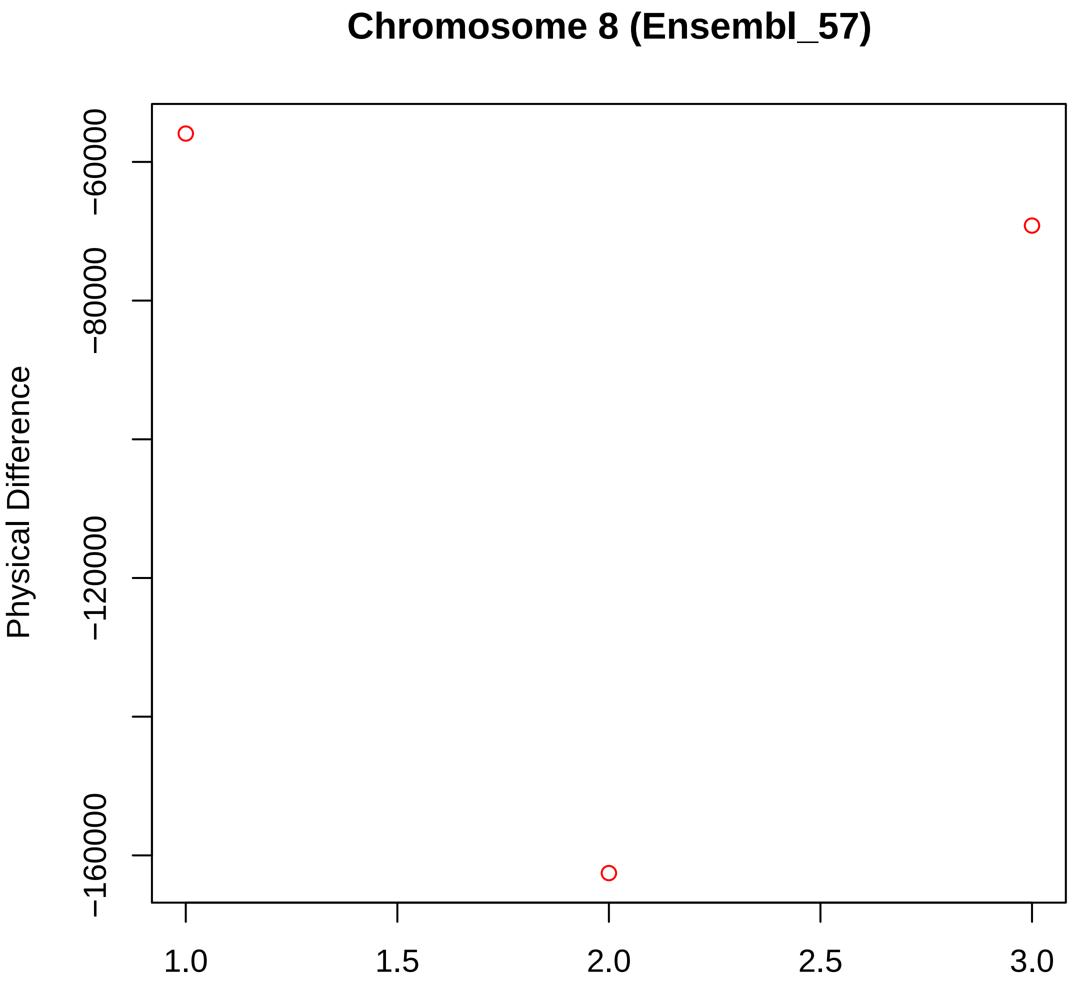

Chromosome 8 (Ensembl_57)
Physical Difference
−600000
−800000
−1200000
−1600000
1.0
1.5
2.0
2.5
3.0
