# Supplementary material for: dbVOR: a database system for importing pedigree, phenotype and genotype data and exporting selected subsets
Source: BMC Bioinformatics. 2015 Mar 18;16(1):91. doi: 10.1186/s12859-015-0505-4 (PMC4407391; doi:10.1186/s12859-015-0505-4)

Chromosome X Genetic vs. Physical Positions

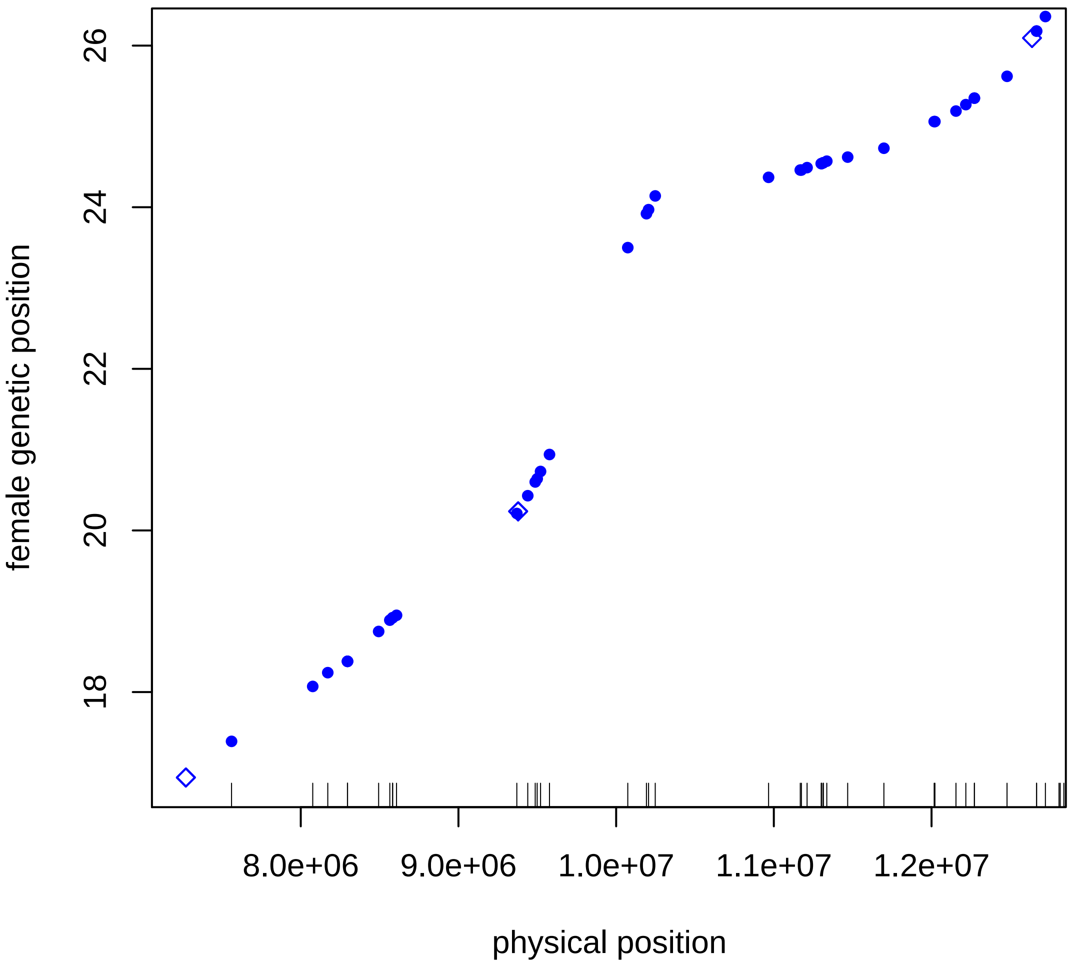

Supplement: Additional file 2 — Contains the entire Genetic Map Interpolator (GMI) package, version 1.4. [file 12859_2015_505_MOESM2_ESM.zip › 1652824117132674_add2.tgz!/GMI_bioc_v1.4/example/X_snp.pdf]
