# Supplementary material for: dbVOR: a database system for importing pedigree, phenotype and genotype data and exporting selected subsets
Source: BMC Bioinformatics. 2015 Mar 18;16(1):91. doi: 10.1186/s12859-015-0505-4 (PMC4407391; doi:10.1186/s12859-015-0505-4)
Supplement: Additional file 2 — Contains the entire Genetic Map Interpolator (GMI) package, version 1.4. [file 12859_2015_505_MOESM2_ESM.zip › 1652824117132674_add2.tgz!/GMI_bioc_v1.4/example/snp_mapped.pdf]

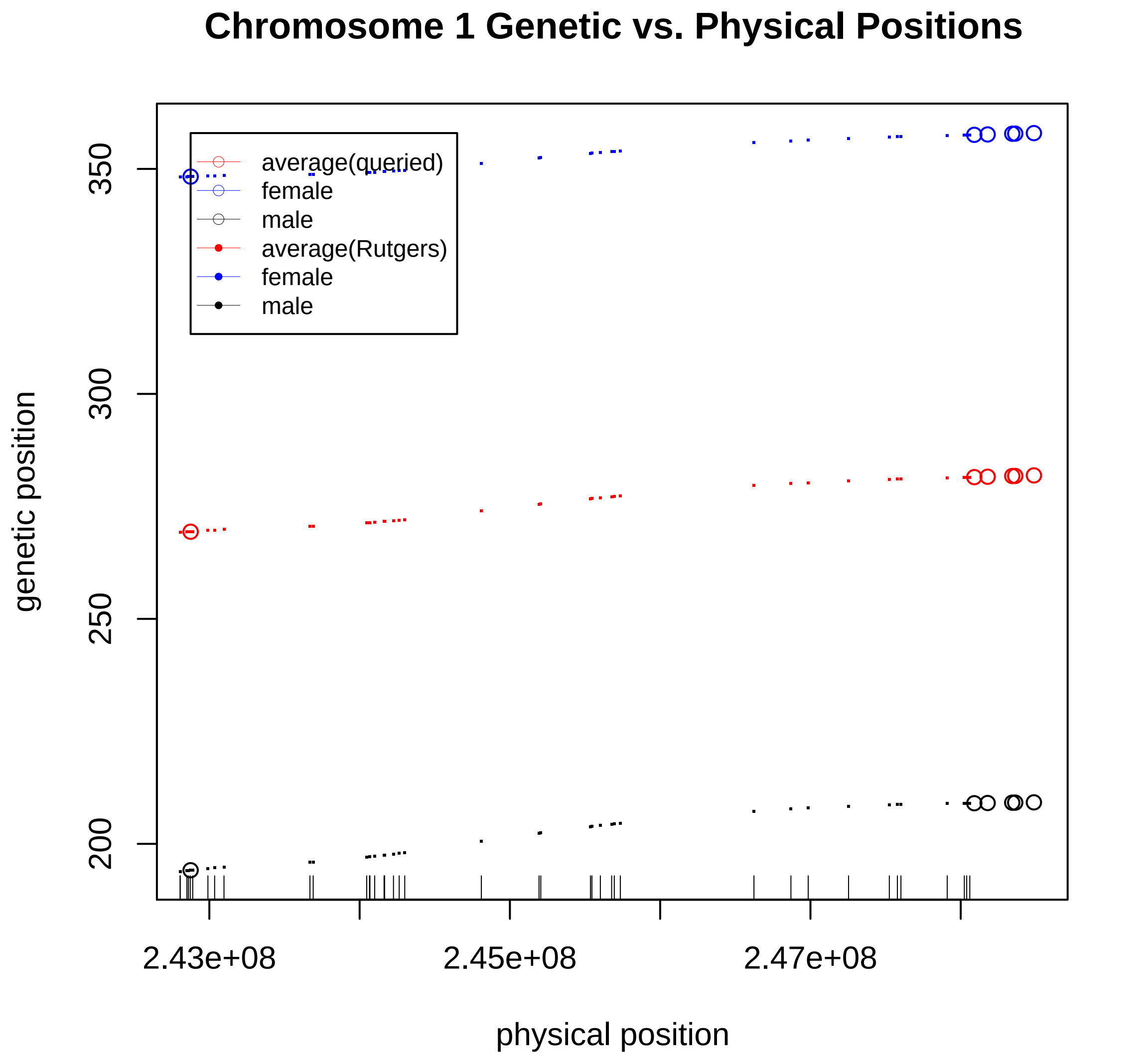

Chromosome 1 Genetic vs. Physical Positions
genetic position
physical position
350
300
250
200
2.43e+08
2.45e+08
2.47e+08
average(queried)
female
male
average(Rutgers)
female
male

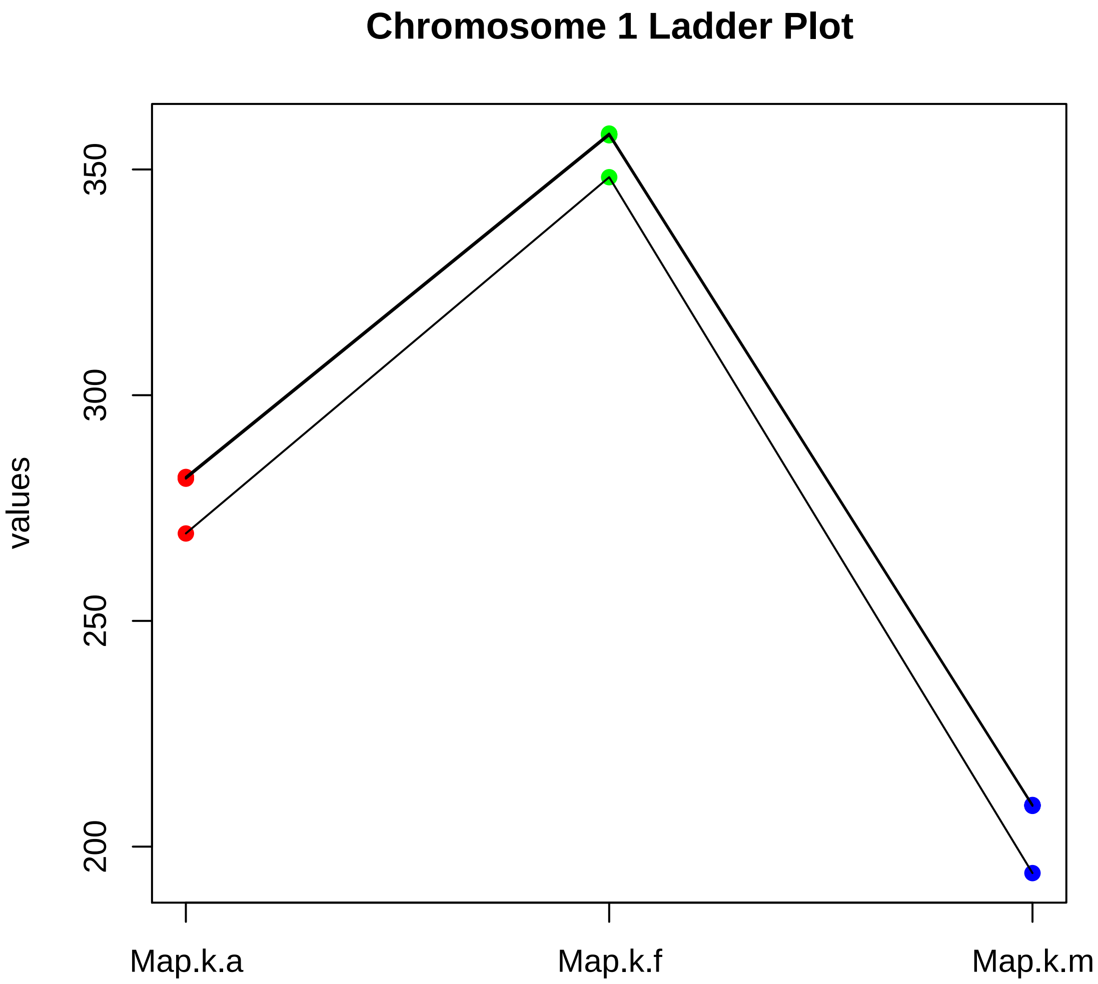

Chromosome 1 Ladder Plot
values
350
300
250
200
Map.k.a
Map.k.f
Map.k.m

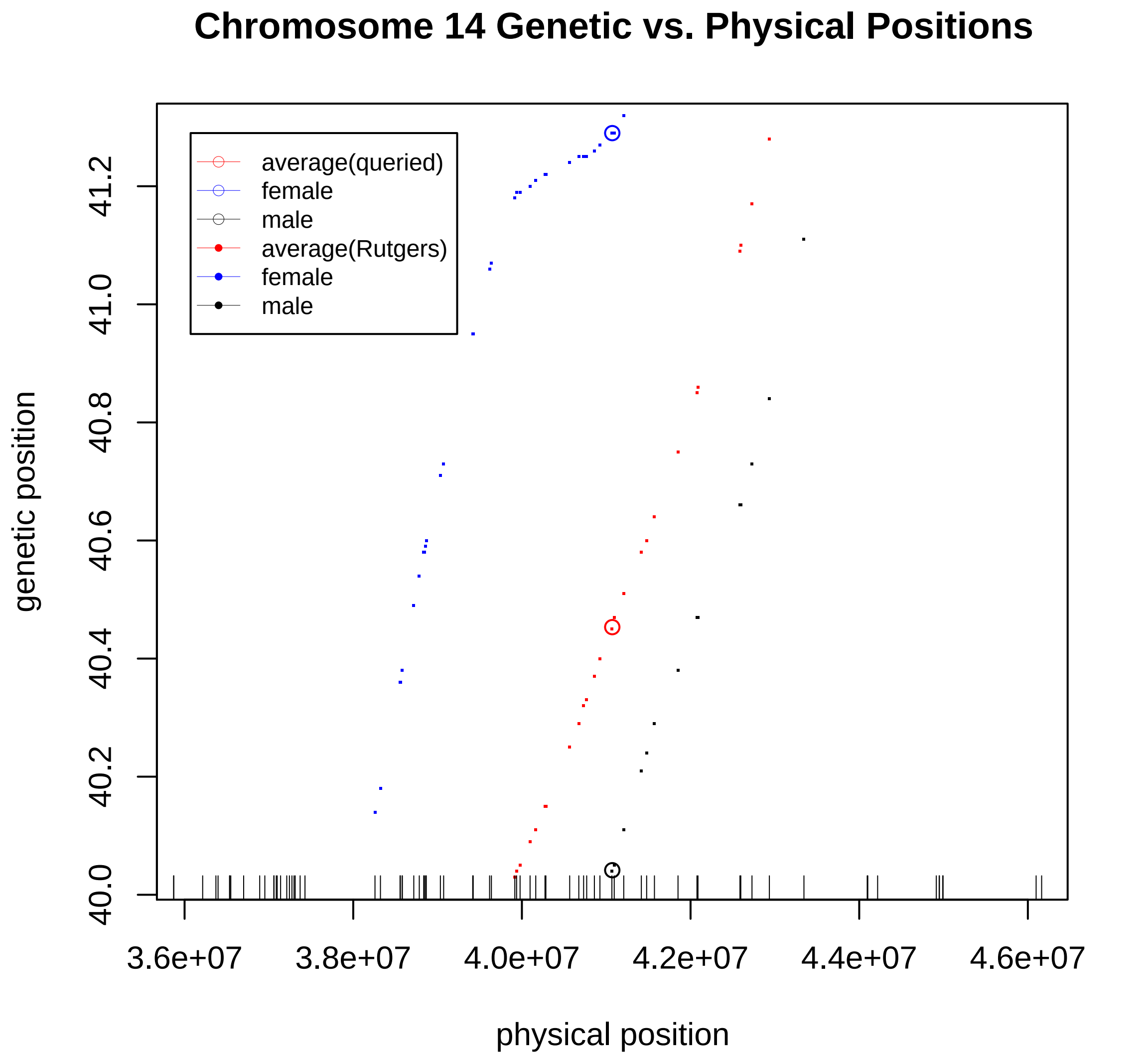

Chromosome 14 Genetic vs. Physical Positions
average(queried)
female
male
average(Rutgers)
female
male
genetic position
40.0
40.2
40.4
40.6
40.8
41.0
41.2
3.6e+07
3.8e+07
4.0e+07
4.2e+07
4.4e+07
4.6e+07
physical position

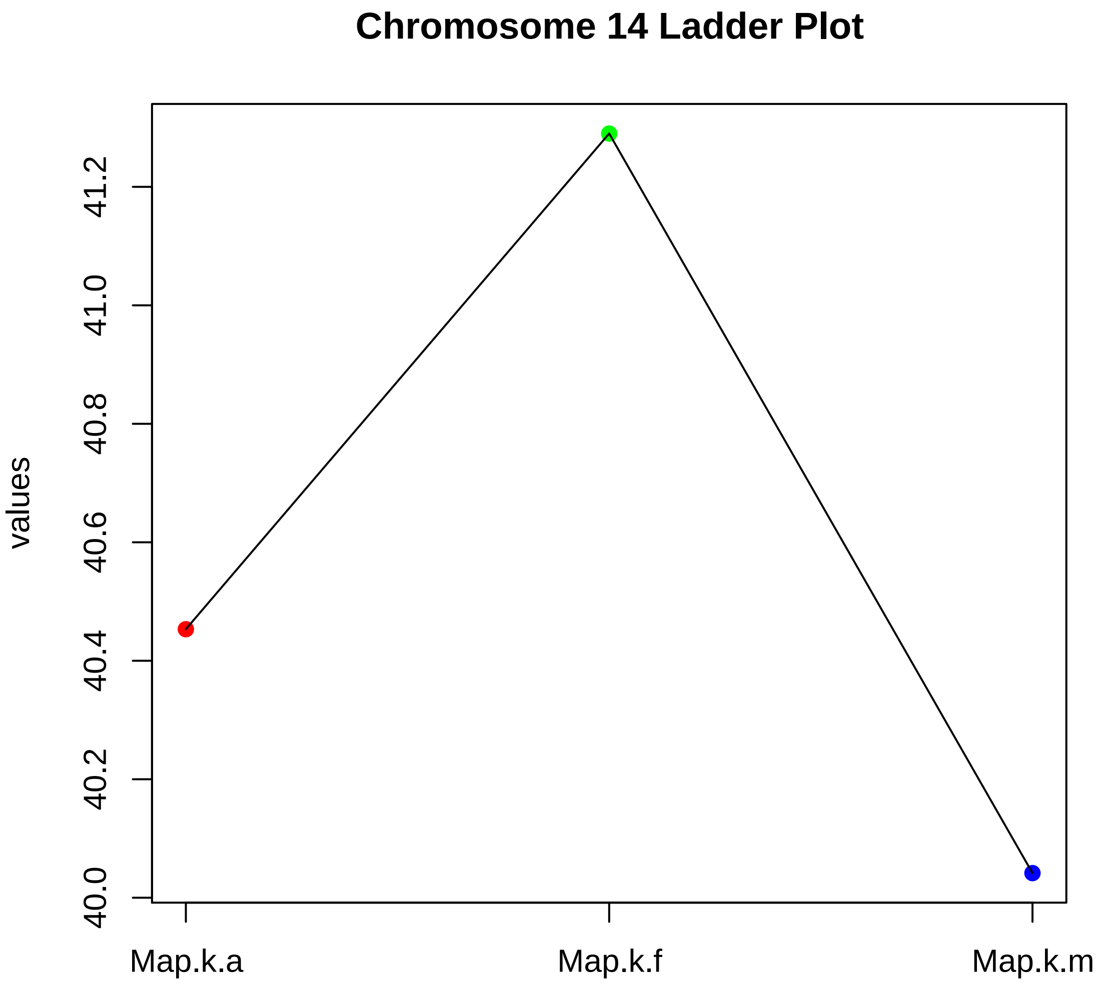
Chromosome 14 Ladder Plot
values
40.0
40.2
40.4
40.6
40.8
41.0
41.2
Map.k.a
Map.k.f
Map.k.m

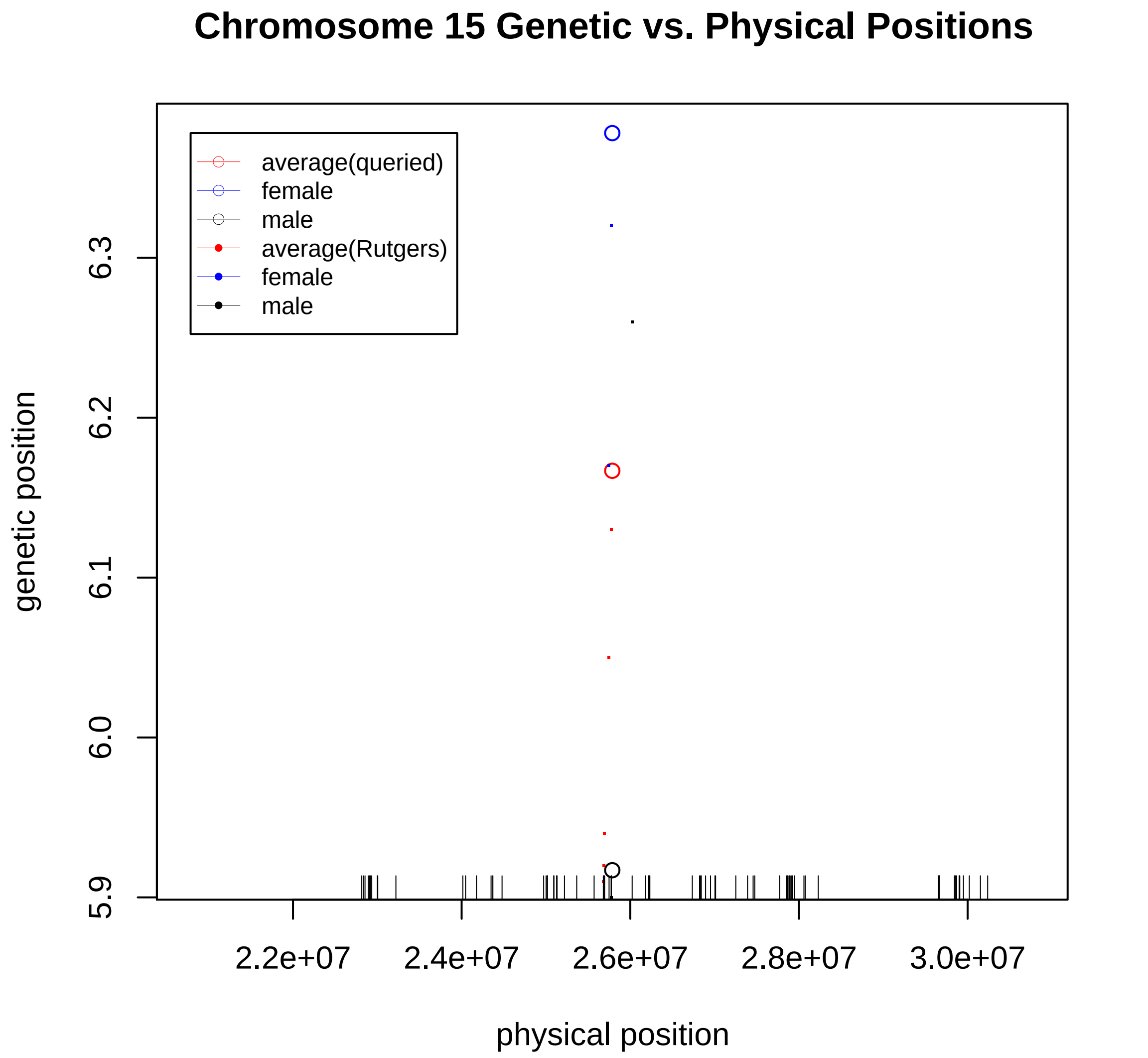
Chromosome 15 Genetic vs. Physical Positions
average(queried)
female
male
average(Rutgers)
female
male
genetic position
5.9
6.0
6.1
6.2
6.3
physical position
2.2e+07
2.4e+07
2.6e+07
2.8e+07
3.0e+07

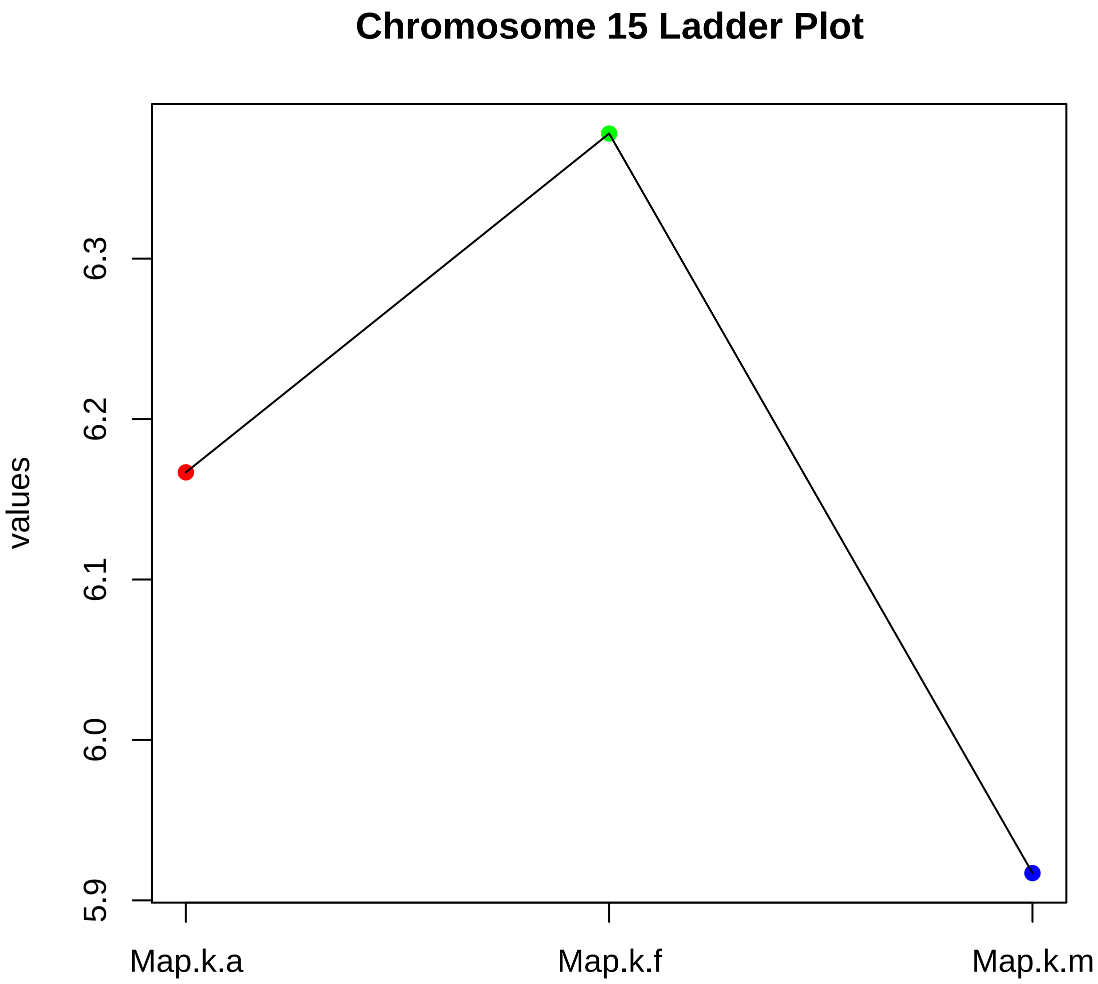

Chromosome 15 Ladder Plot
values
5.9
6.0
6.1
6.2
6.3
Map.k.a
Map.k.f
Map.k.m

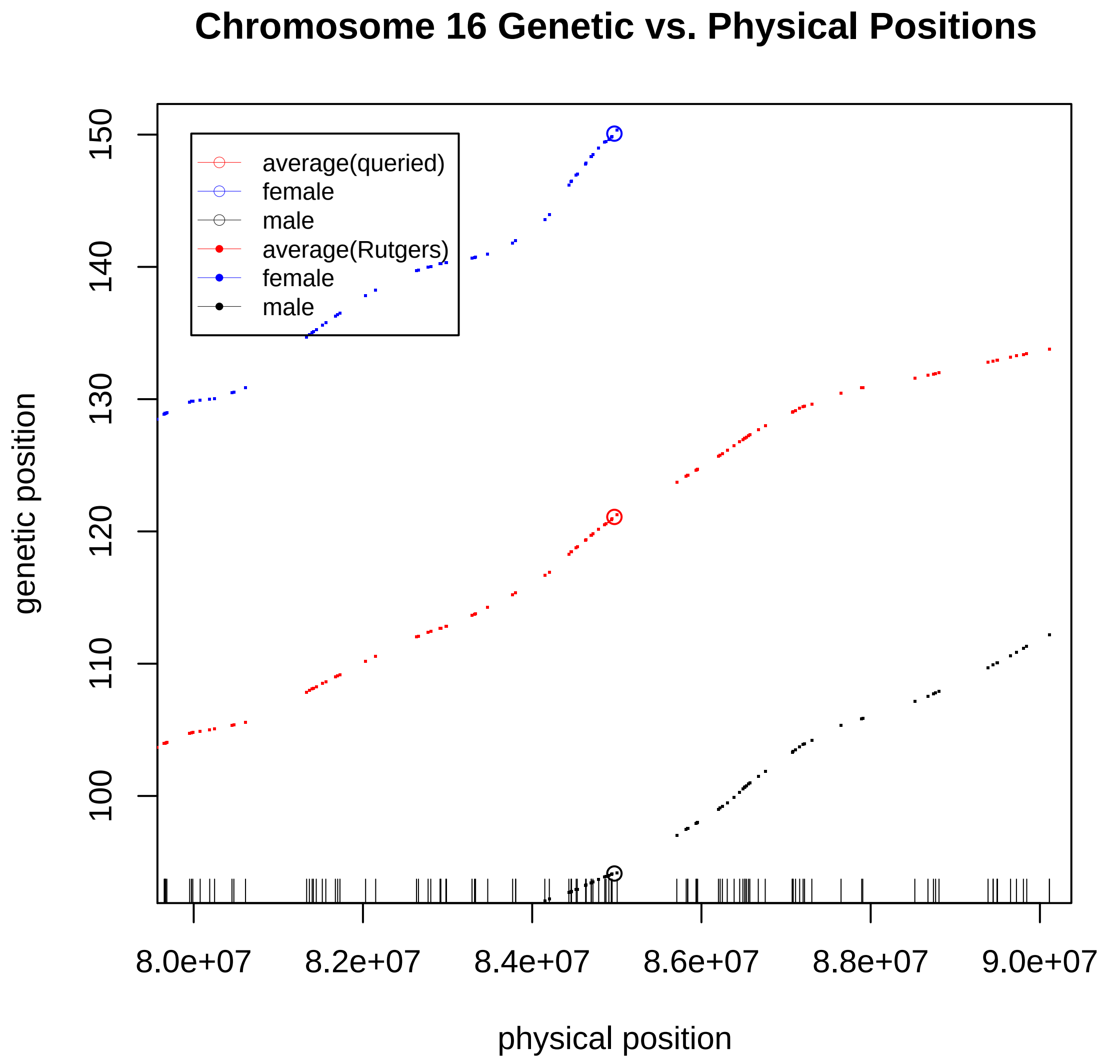

Chromosome 16 Genetic vs. Physical Positions
genetic position
physical position
100
110
120
130
140
150
8.0e+07
8.2e+07
8.4e+07
8.6e+07
8.8e+07
9.0e+07
average(queried)
female
male
average(Rutgers)
female
male

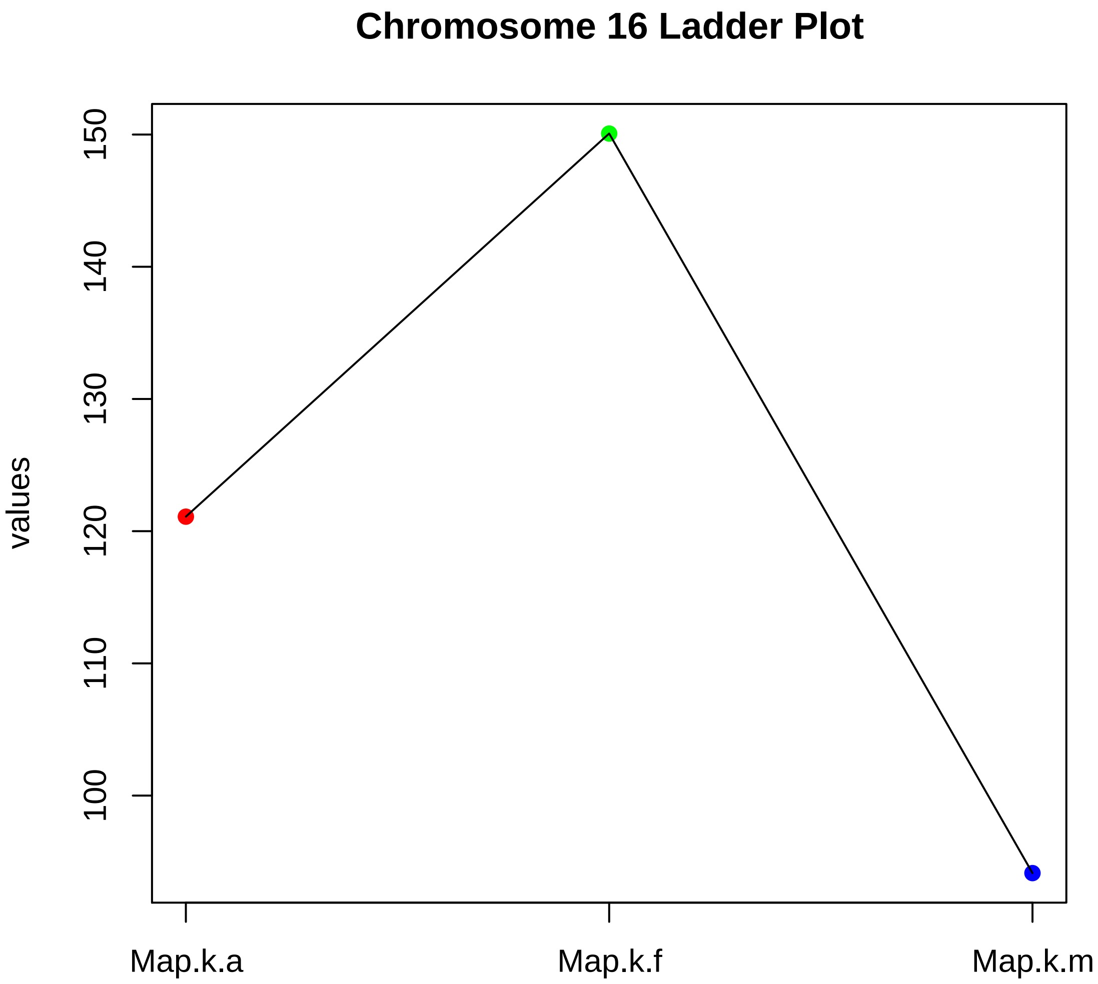
Chromosome 16 Ladder Plot
values
150
140
130
120
110
100
Map.k.a
Map.k.f
Map.k.m

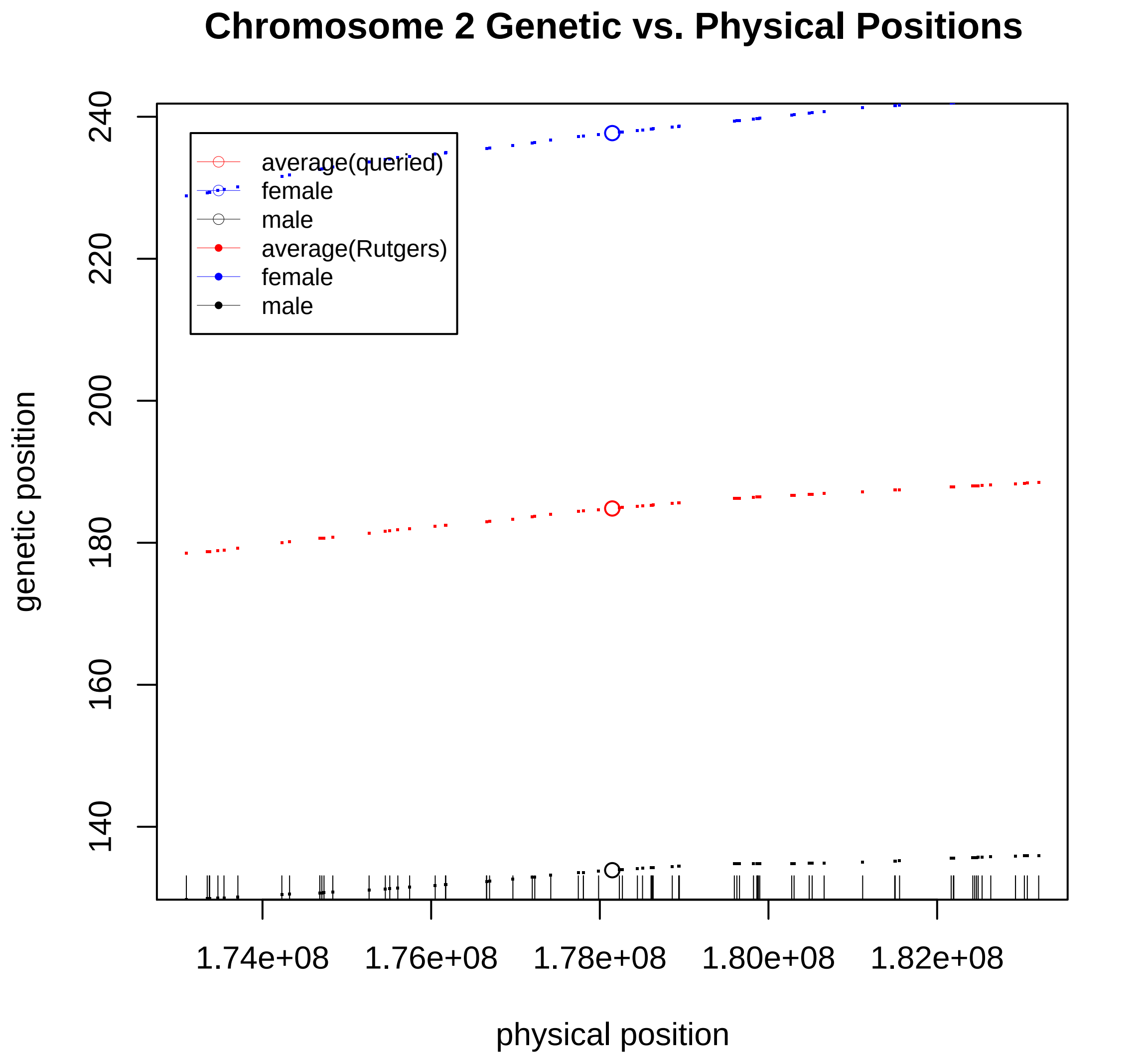

Chromosome 2 Genetic vs. Physical Positions
average(queried)
female
male
average(Rutgers)
female
male
genetic position
140
160
180
200
220
240
physical position
1.74e+08
1.76e+08
1.78e+08
1.80e+08
1.82e+08

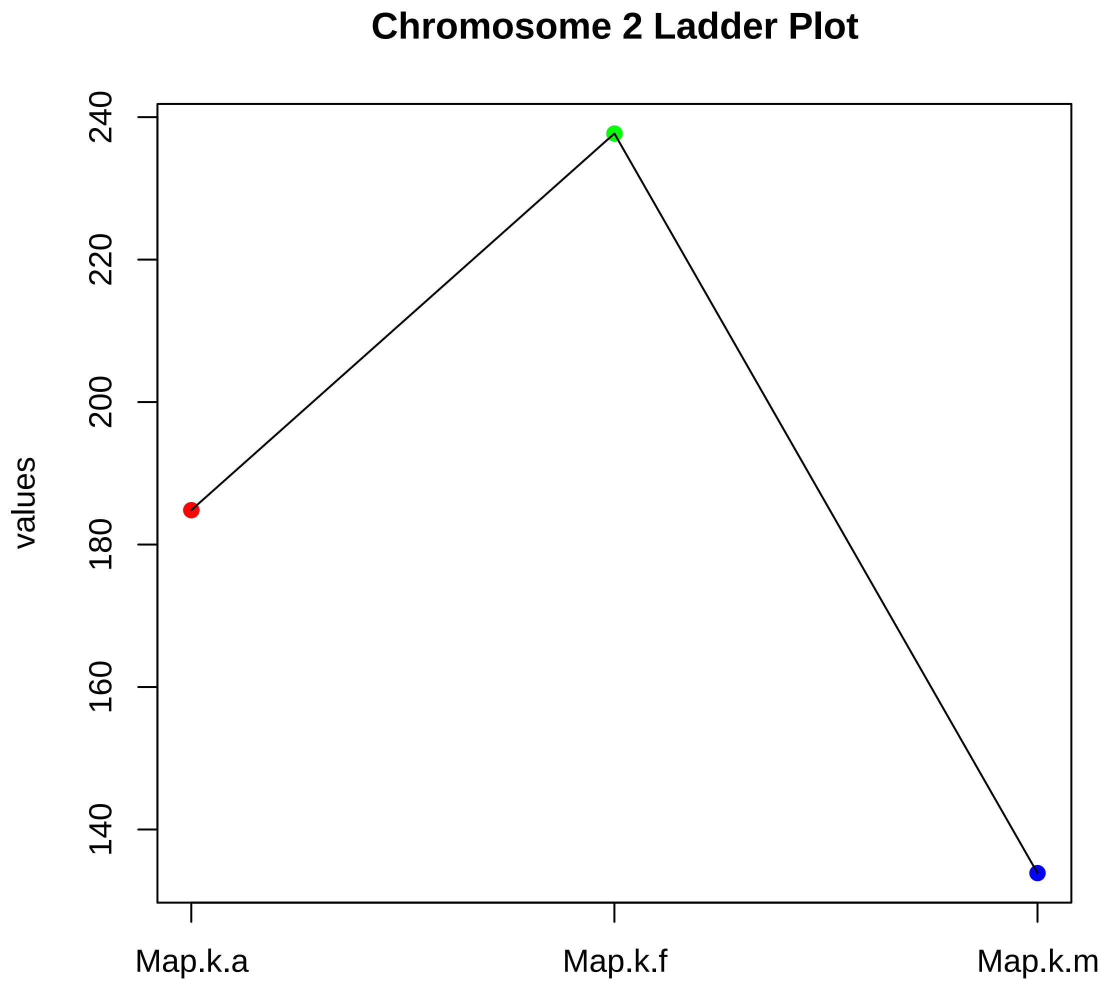
Chromosome 2 Ladder Plot
values
240
220
200
180
160
140
Map.k.a
Map.k.f
Map.k.m

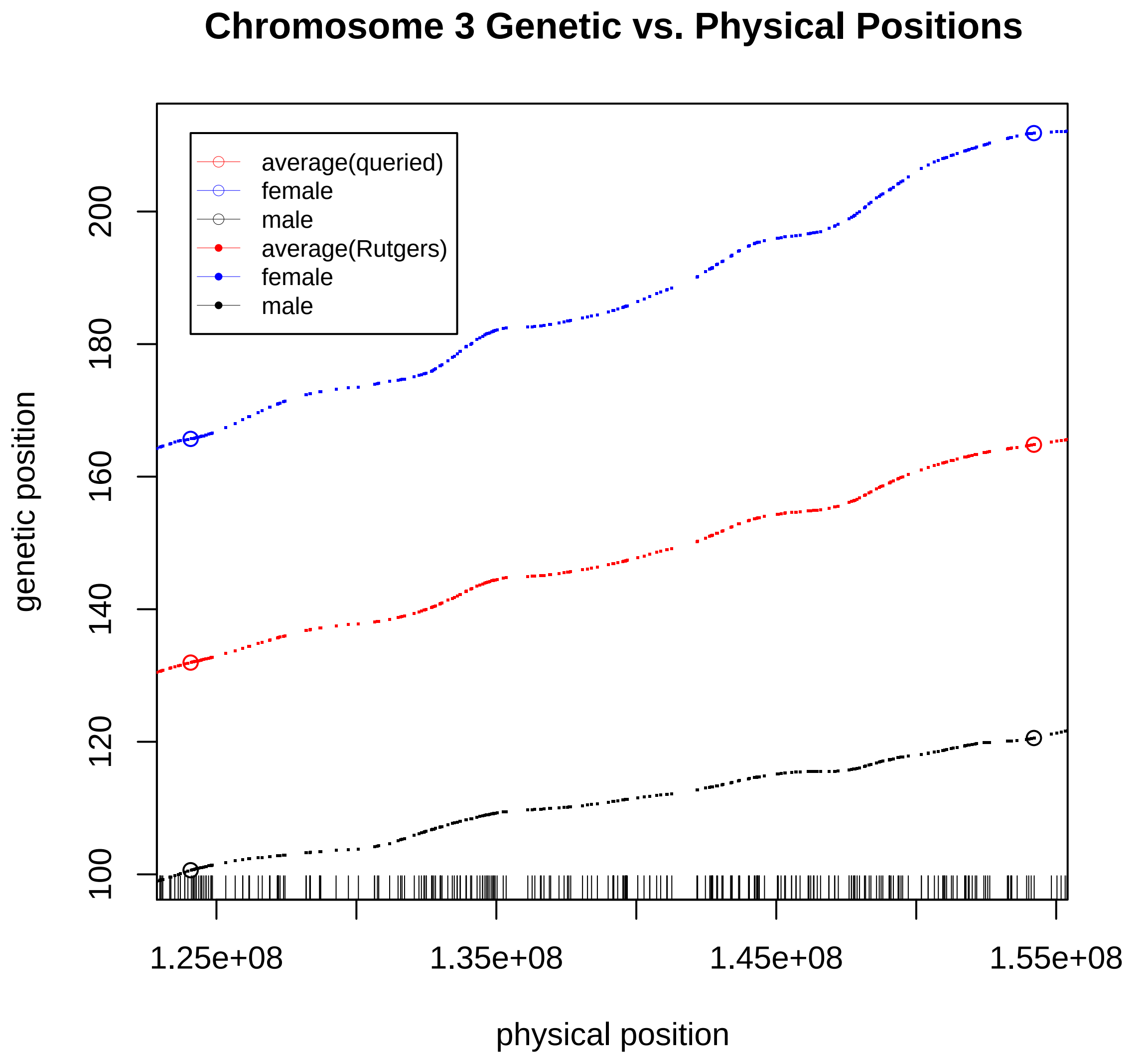

Chromosome 3 Genetic vs. Physical Positions
average(queried)
female
male
average(Rutgers)
female
male
genetic position
100
120
140
160
180
200
1.25e+08
1.35e+08
1.45e+08
1.55e+08
physical position

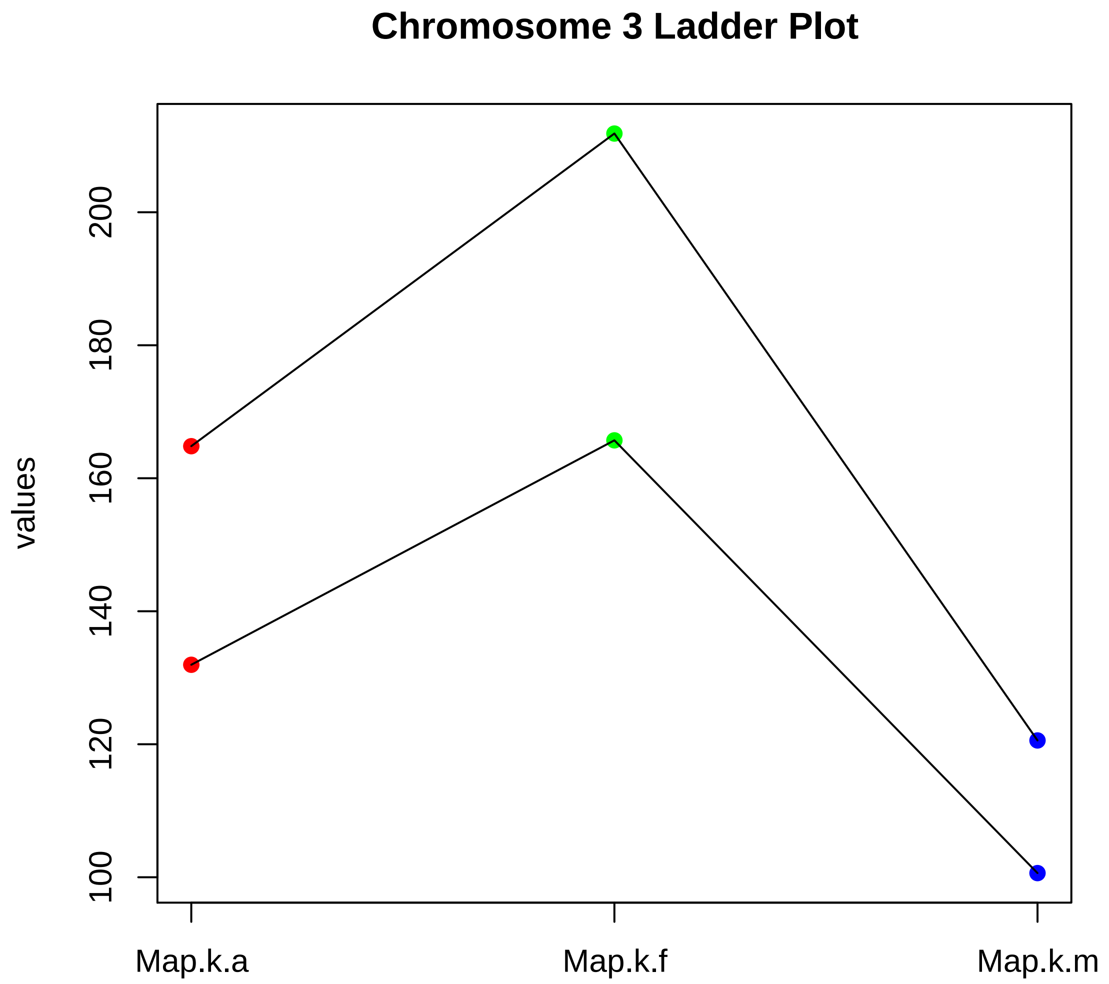

Chromosome 3 Ladder Plot
values
200
180
160
140
120
100
Map.k.a
Map.k.f
Map.k.m

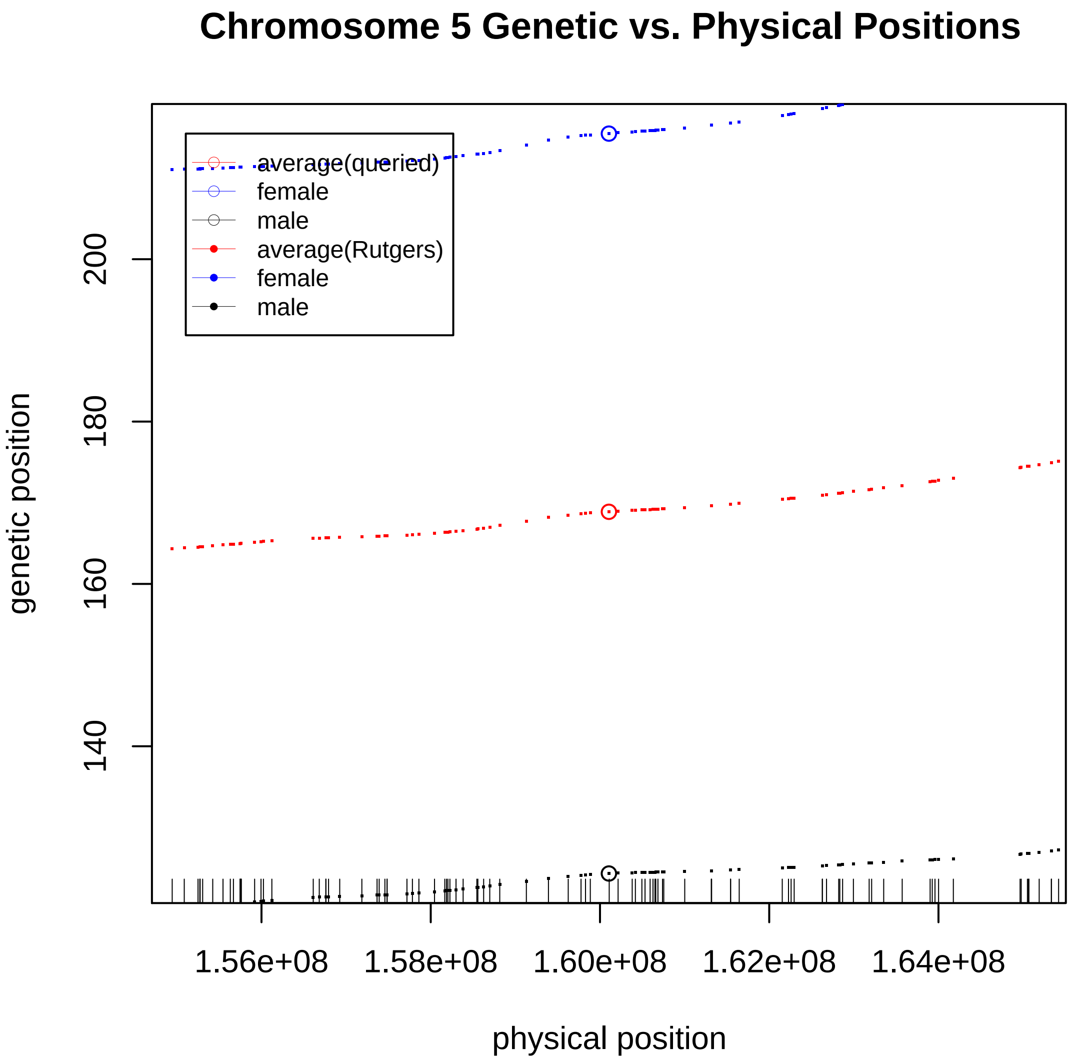
Chromosome 5 Genetic vs. Physical Positions
average(queried)
female
male
average(Rutgers)
female
male
genetic position
200
180
160
140
1.56e+08
1.58e+08
1.60e+08
1.62e+08
1.64e+08
physical position

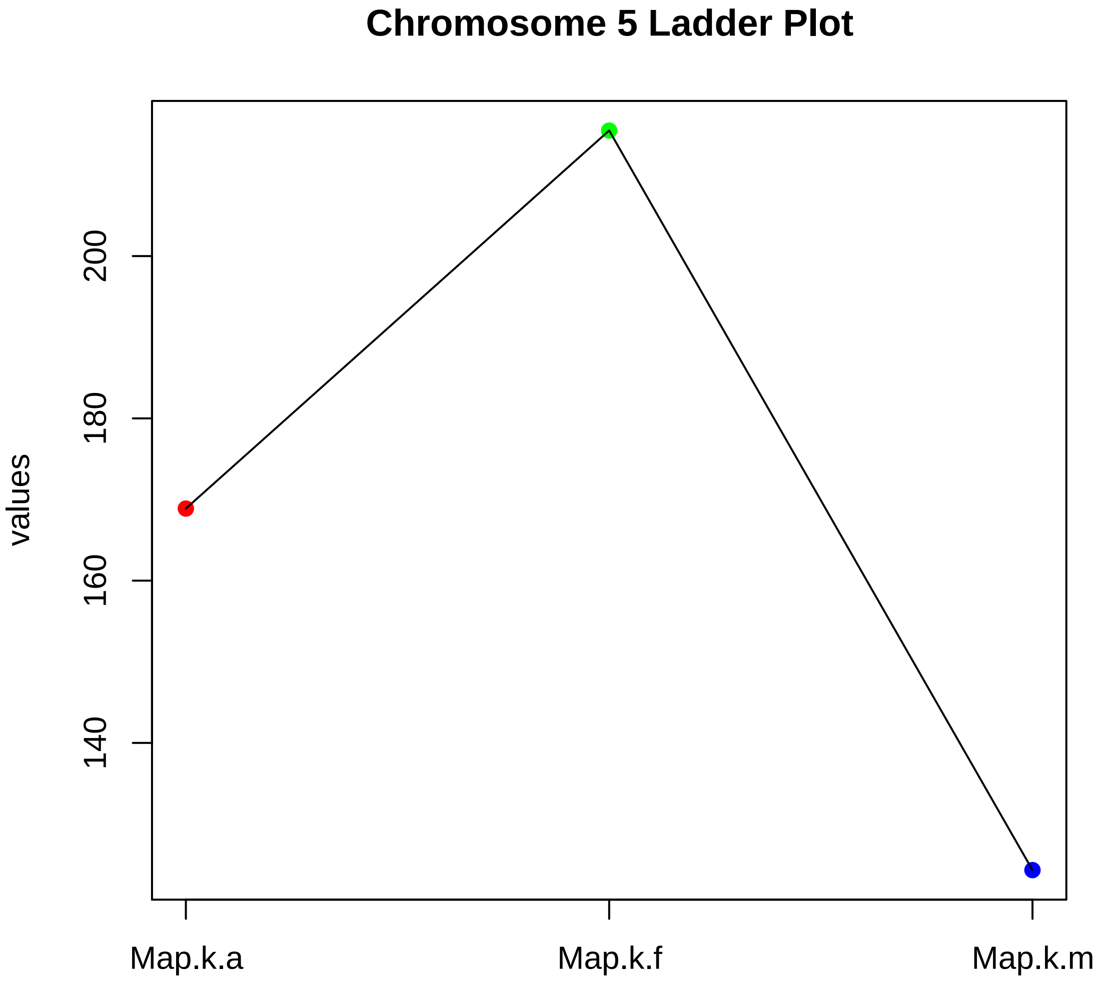
Chromosome 5 Ladder Plot
values
200
180
160
140
Map.k.a
Map.k.f
Map.k.m

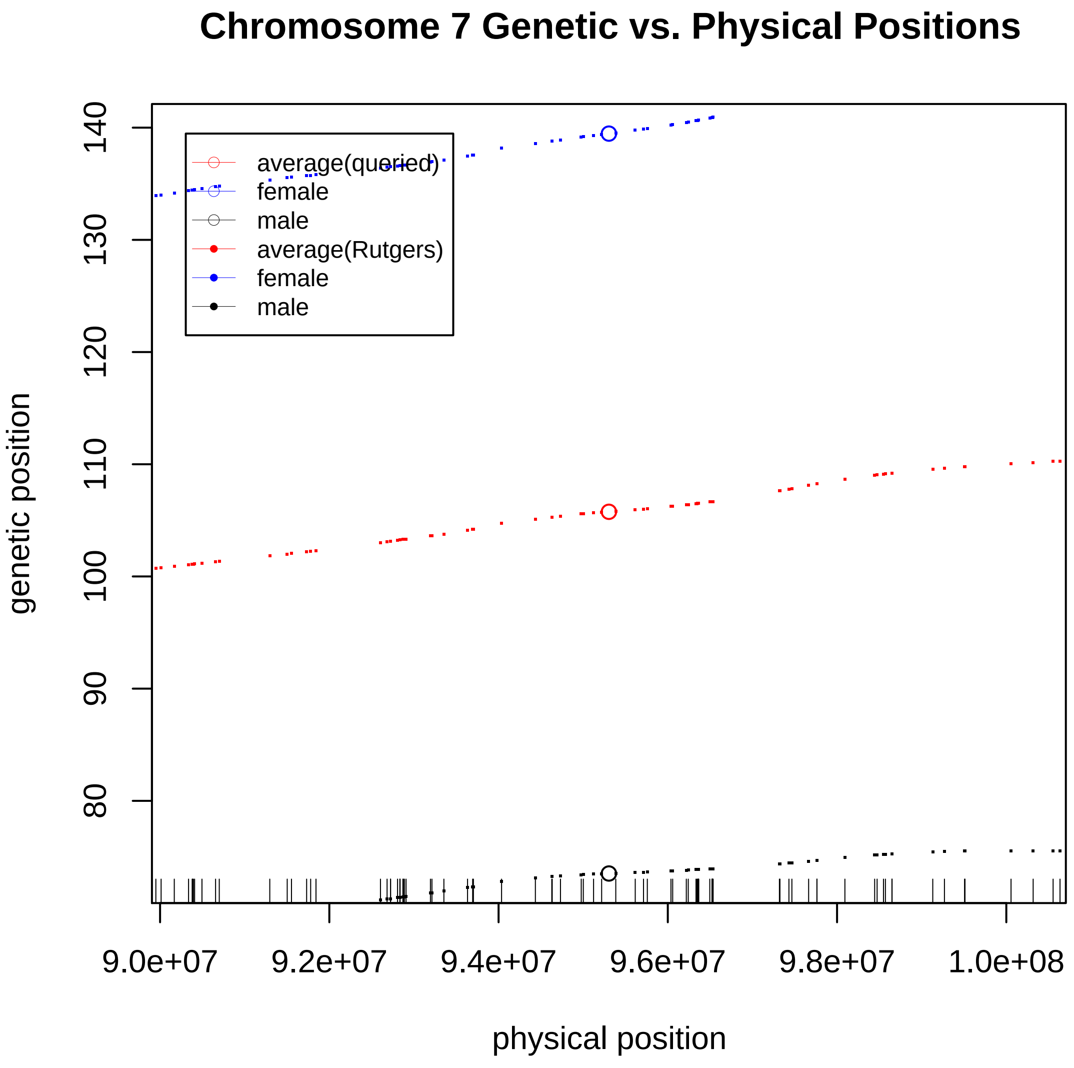
Chromosome 7 Genetic vs. Physical Positions
average(queried)
female
male
average(Rutgers)
female
male
genetic position
80
90
100
110
120
130
140
physical position
9.0e+07
9.2e+07
9.4e+07
9.6e+07
9.8e+07
1.0e+08

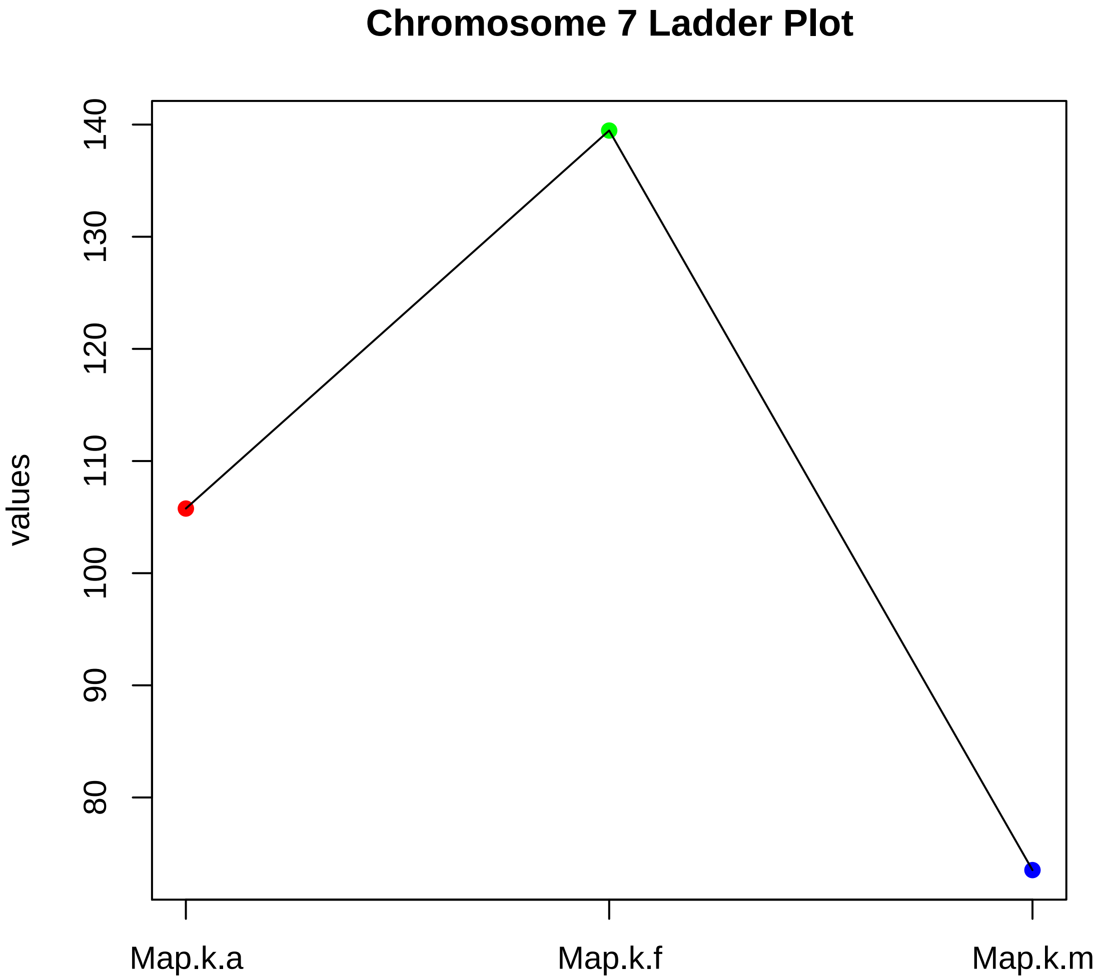

Chromosome 7 Ladder Plot
values
140
130
120
110
100
90
80
Map.k.a
Map.k.f
Map.k.m

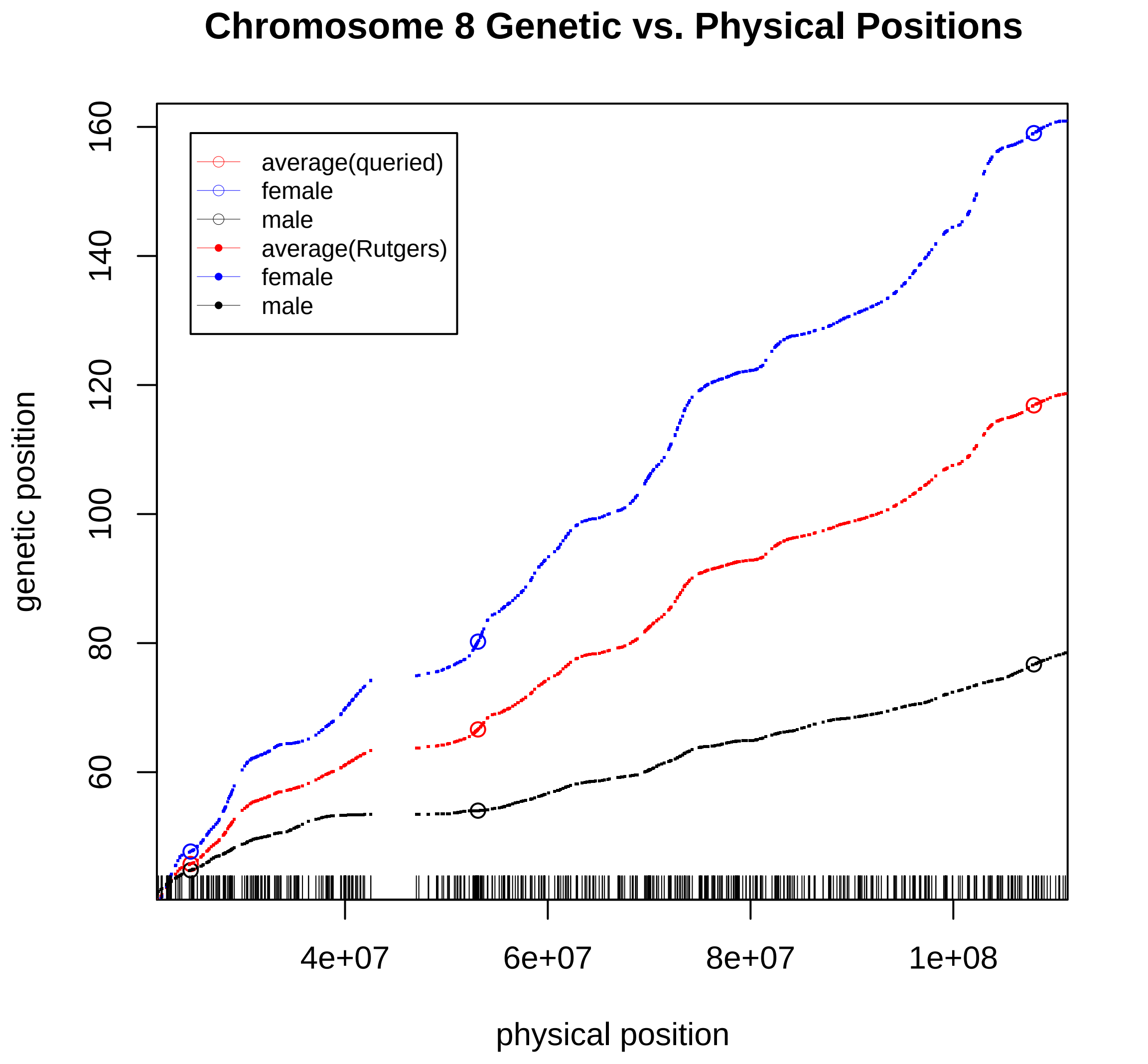
Chromosome 8 Genetic vs. Physical Positions
genetic position
physical position
160
140
120
100
80
60
4e+07
6e+07
8e+07
1e+08
average(queried)
female
male
average(Rutgers)
female
male

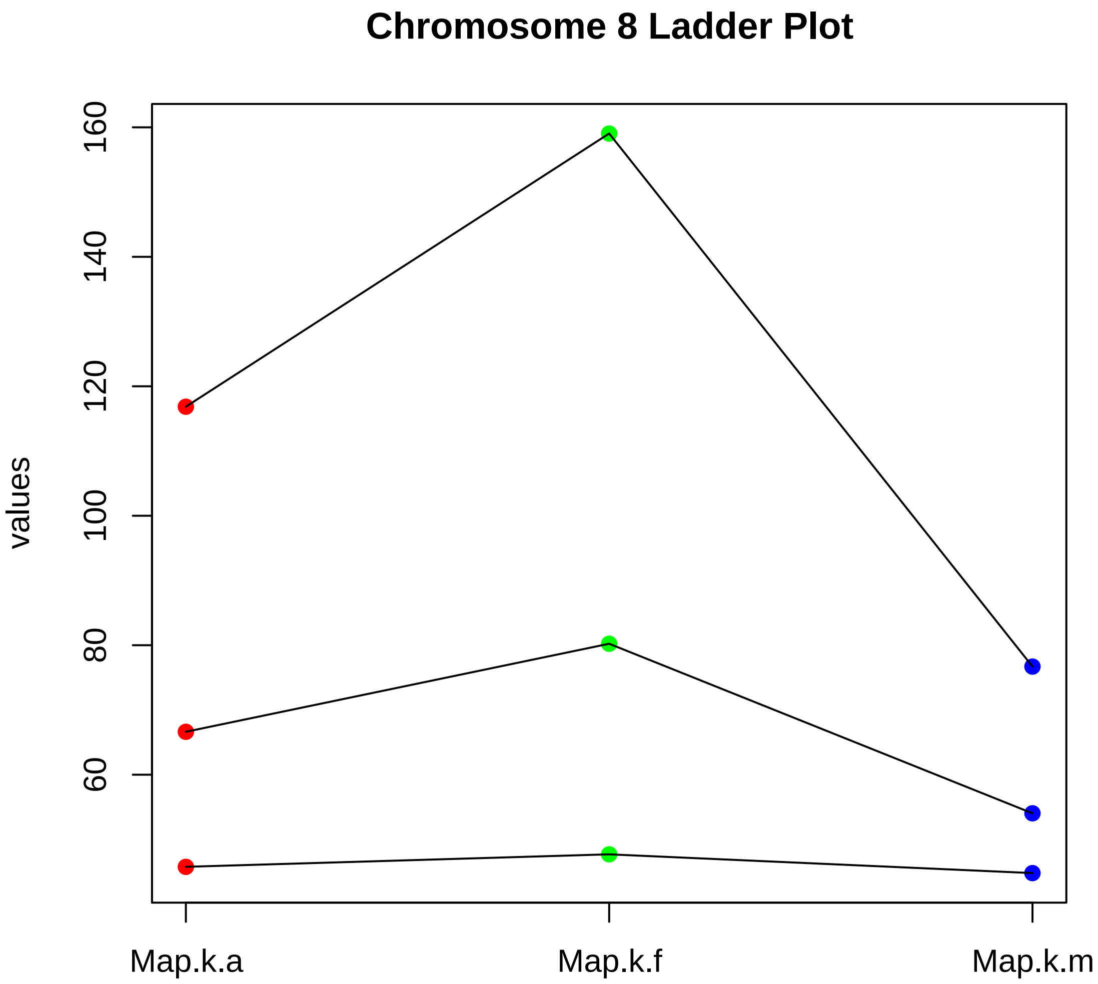
Chromosome 8 Ladder Plot
values
60
80
100
120
140
160
Map.k.a
Map.k.f
Map.k.m
